# 4-(Pyrazolyl)benzenesulfonamide Ureas as Carbonic Anhydrases Inhibitors and Hypoxia-Mediated Chemo-Sensitizing Agents in Colorectal Cancer Cells

**DOI:** 10.1021/acs.jmedchem.4c01894

**Published:** 2024-11-17

**Authors:** Wagdy M. Eldehna, Mohamed Fares, Alessandro Bonardi, Moscos Avgenikos, Fady Baselious, Matthias Schmidt, Tarfah Al-Warhi, Hatem A. Abdel-Aziz, Robert Rennert, Thomas S. Peat, Claudiu T. Supuran, Ludger A. Wessjohann, Hany S. Ibrahim

**Affiliations:** † Department of Pharmaceutical Chemistry, Faculty of Pharmacy, Kafrelsheikh University, Kafrelsheikh 33516, Egypt; ‡ Department of Pharmaceutical Chemistry, Faculty of Pharmacy, Pharos University in Alexandria, Canal El Mahmoudia Street, Alexandria 21648, Egypt; § Department of Pharmaceutical Chemistry, Faculty of Pharmacy, Egyptian Russian University, Badr City, Cairo 11829, Egypt; ∥ Sydney Pharmacy School, The University of Sydney, Sydney, New South Wales 2006, Australia; ⊥ Department NEUROFARBA—Pharmaceutical and Nutraceutical Section, University of Firenze, via Ugo Schiff 6, Sesto Fiorentino I-50019, Firenze, Italy; # Department of Bioorganic Chemistry, Leibniz Institute of Plant Biochemistry, Halle (Saale) D-06120, Germany; ¶ Department of Medicinal Chemistry, Institute of Pharmacy, Martin-Luther-University of Halle-Wittenberg, Halle (Saale) D-06120, Germany; ∇ Department of Chemistry, College of Science, Princess Nourah Bint Abdulrahman University, Riyadh 11564, Saudi Arabia; ○ Applied Organic Chemistry Department, National Research Center, Dokki, Giza 12622, Cairo, Egypt; ⧫ School of Biotechnology and Biomolecular Sciences, University of New South Wales, Sydney, New South Wales 2052, Australia

## Abstract

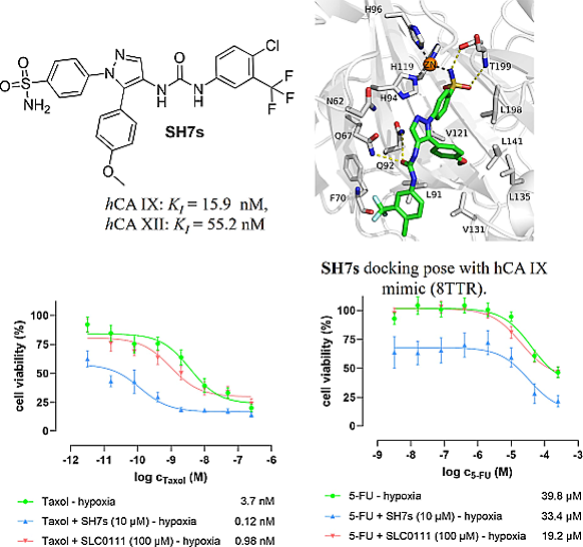

Hypoxia in tumors contributes to chemotherapy resistance, worsened by acidosis driven by carbonic anhydrases (*h*CA IX and XII). Targeting these enzymes can mitigate acidosis, thus enhancing tumor sensitivity to cytotoxic drugs. Herein, novel 4-(pyrazolyl)benzenesulfonamide ureas (**SH7a–t**) were developed and evaluated for their inhibitory activity against *h*CA IX and XII. They showed promising results (*h*CA IX: *K*_I_ = 15.9–67.6 nM, *h*CA XII: *K*_I_ = 16.7–65.7 nM). Particularly, **SH7s** demonstrated outstanding activity (*K*_I_s = 15.9 nM for *h*CA IX and 55.2 nM for *h*CA XII) and minimal off-target kinase inhibition over a panel of 258 kinases. In NCI anticancer screening, **SH7s** exhibited broad-spectrum activity with an effective growth inhibition full panel GI_50_ (MG-MID) value of 3.5 μM and a subpanel GI_50_ (MG-MID) range of 2.4–6.3 μM. Furthermore, **SH7s** enhanced the efficacy of Taxol and 5-fluorouracil in cotreatment regimens under hypoxic conditions in HCT-116 colorectal cancer cells, indicating its potential as a promising anticancer agent.

## 1 Introduction

Hypoxia in the tumor microenvironment (TME) is considered one of the main factors that aid solid tumors, such as colon cancer, to be resistant to either chemotherapy or radiotherapy.^1^ Hypoxia occurs in the TME due to the release of CO_2_ during the metabolism of glucose. As a result of CO_2_ release in hypoxia, an acidic environment will be established due to the catalytic activity of the tumor-associated human carbonic anhydrases *h*CA IX and *h*CA XII.^2^ This catalytic activity will lead to the accumulation of H^+^ outside cancer cells in the TME, which leads to an acidic pH 6.3–7. Hypoxia-related acidosis is considered a contributing factor for the inactivation of cytotoxic drugs.^3^ Targeting the tumor-associated carbonic anhydrase isoforms IX and XII by inhibition is a strategy to counteract the factors causing hypoxia-related acidosis, and hence a sensitization of hypoxic tumors toward cytotoxic drugs may take place.^4^

Different strategies for designing an inhibitor for tumor-associated carbonic anhydrases have been previously reported.^5^ As a result, **SLC-0111** has emerged as a selective *h*CA IX and *h*CA XII inhibitor in clinical trials for the cotreatment of advanced solid tumors in conjunction with the cytostatic drug gemcitabine.^6^

**Figure 1:**
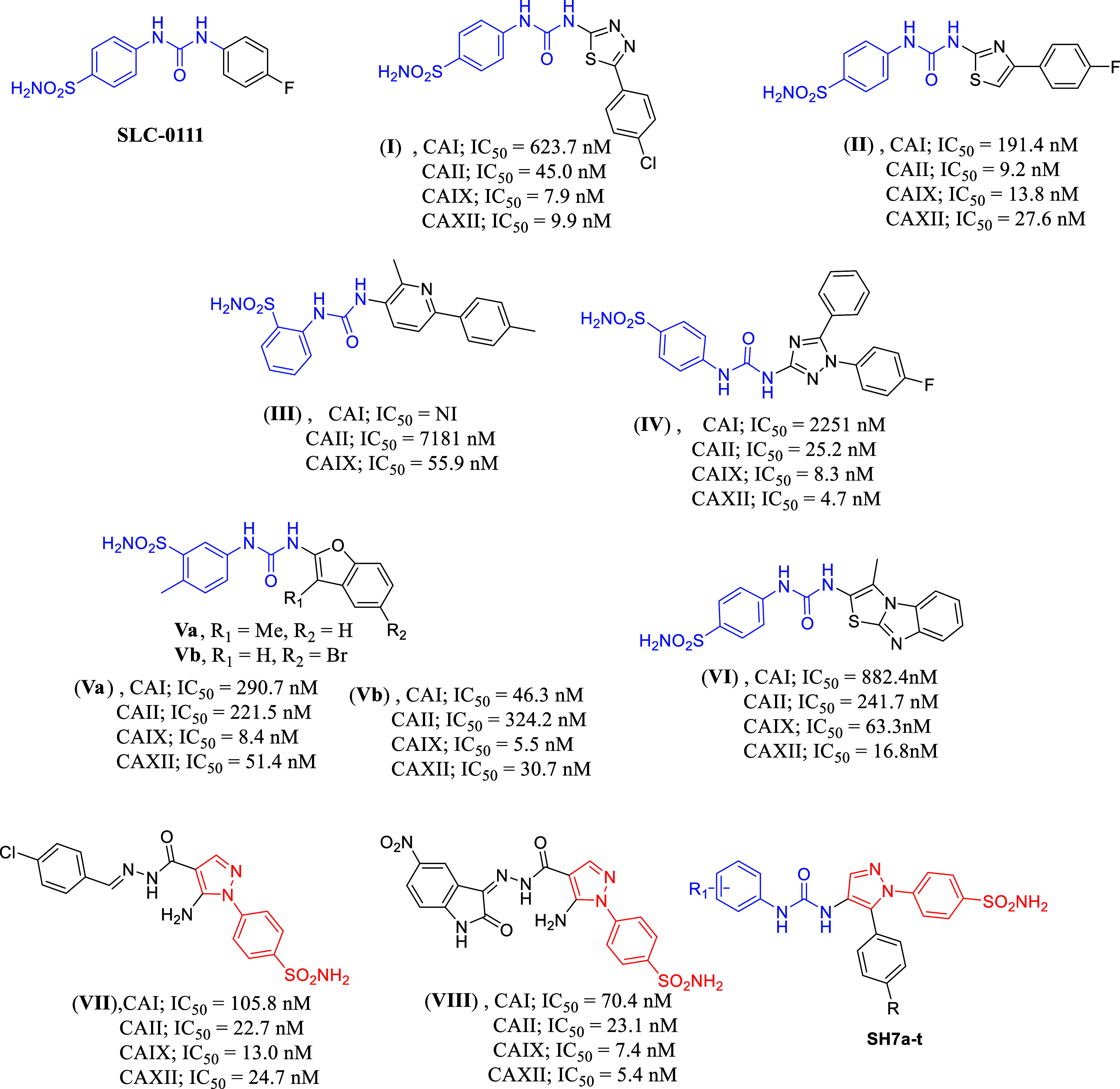
Previously reported compounds containing the urea moiety combined with a benzenesulfonamide moiety and their inhibitory effects against different *h*CA isoforms, and the formula of compounds **SH7a–t** discussed in this study.

After the emergence of **SLC-0111** as selective tumor-associated carbonic anhydrase inhibitor in clinical trials, our group conducted several trials to innovate a library of analogues leveraging bioisosterism at the terminal phenyl group, incorporating other heterocyclic scaffolds such as thiazole, thiadiazole, triazole and pyridine. The reported examples (**I**),^7^ (**II**),^7^ (**III**)^8^ and (**IV**)^9^ showed that the thiadiazole analogue (**I**) and triazole analogue (**III**) (Figure 1) can inhibit tumor-associated carbonic anhydrase isoforms in the very low nanomolar range, however, the selectivity index over the non-pathogenic *h*CA II was not promising. The other isosteric analogues displayed inhibitory activity for *h*CA IX and *h*CA XII in the low nanomolar range, and the pyridine analogue (**III**) exhibited a suitable selectivity index. Exchanging the phenyl tail in **SLC-0111** with a bicylic heterocyclic ring was a successful strategy to innovate potent inhibitors. For example, benzofuran in compounds **Va** and **Vb**(10) and a tricyclic heterocyclic such as thiazolo[3,2-*a*]benzimidazole (compound **VI**)^11^ yielded successful inhibitors with a promising selectivity index over CA II. Other *h*CA inhibitors contain an urea moiety and they were inspired from **SLC-0111** as reported in literature.^12−16^

Additionally, pyrazole benzenesulfonamides were synthesized to serve as carbonic anhydrase inhibitors, represented by the compounds **VII** and **VIII**, which displayed potent inhibitory activity against *h*CA IX and *h*CA XII with a low selectivity index.

In this research work, we have combined the phenyl urea scaffold with the pyrazole benzenesulfonamide as in the **SH7** series to make use of the promising activity of the previously reported compounds and to study the effect of this combination on the selectivity indices. Enzymatic SAR studies of this series laid the foundation for whole cell testing of the most promising compounds.

## 2 Results and Discussion

### 2.1 Chemistry

**Scheme 1:**
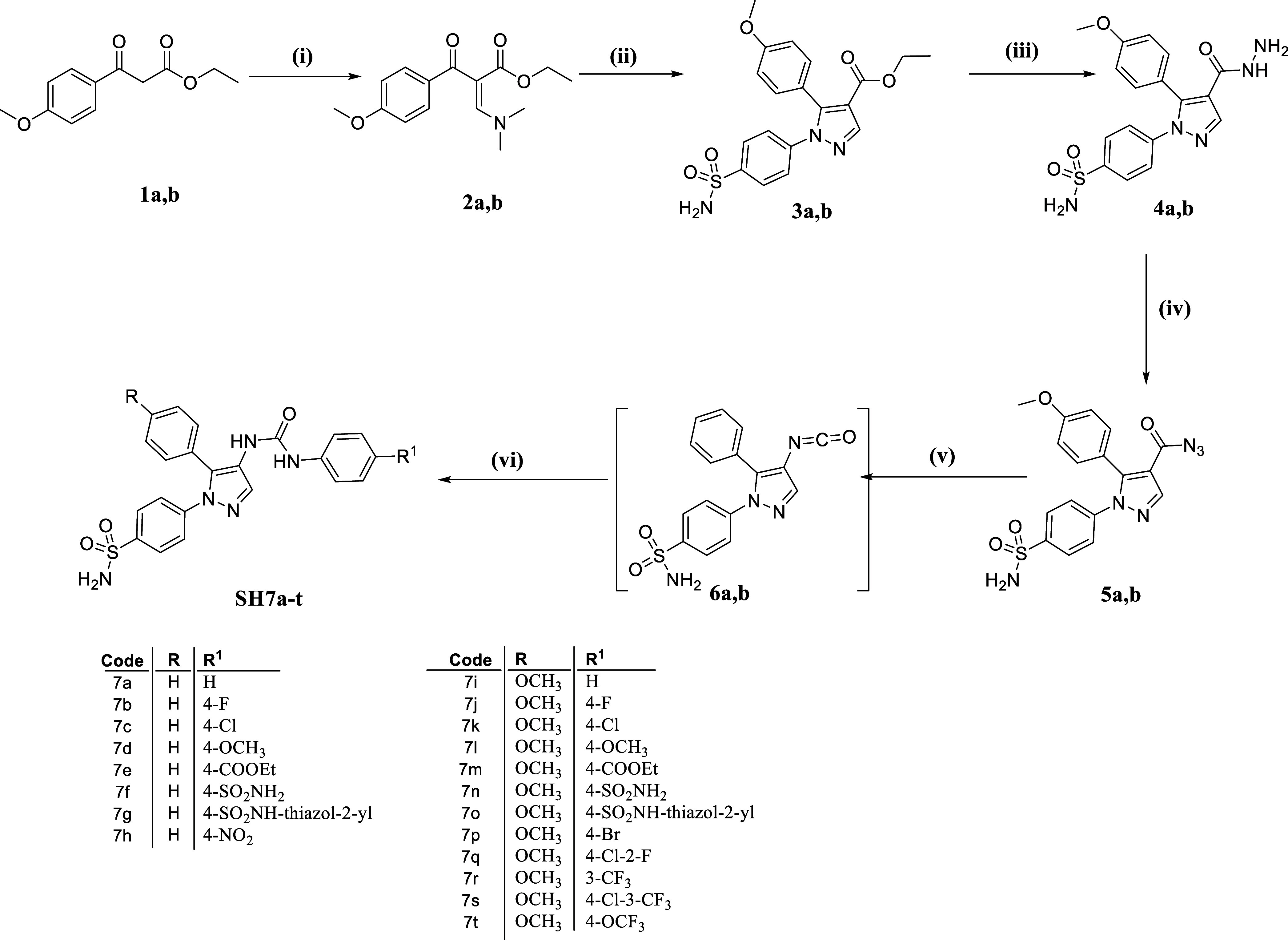
Syntheses of the Target Ureides **SH7a–t** Reagents and conditions: (i) DMF-DMA, reflux, 1 h; (ii) SO_2_NH_2_–C_6_H_4_–NHNH_2_·HCl, EtOH, reflux, 2 h; (iii) NH_2_NH_2_·H_2_O 95%, reflux 3 h; (iv) NaNO_2_, AcOH, 0–5 °C, 45 min; (v) toluene, reflux 1 h; (vi) R^1^–C_6_H_4_–NH_2_, toluene, reflux 2 h.

Scheme 1 outlines the sequence used to synthesize the target urea-incorporated pyrazoles **SH7a–t** of this study. Initially, ethyl 3-oxo-3-arylpropanoates **1a**–**b** were subjected to reflux with *N*,*N*-dimethylformamide dimethyl acetal (DMF-DMA), resulting in the formation of enaminone-based intermediates **2a**–**b**. These intermediates were then heated under reflux temperature with 4-hydrazinyl-benzenesulfonamide hydrochloride in absolute ethanol, where the dimethylamino group was substituted by the hydrazinyl group before a spontaneous cyclization occurred, yielding pyrazole-based ethyl esters **3a**–**b**. Subsequently, derivatives **3a**–**b** were subjected to hydrazinolysis through refluxing with hydrazine hydrate 95%, producing hydrazides **4a**–**b**. The hydrazides were then reacted with sodium nitrite in cold glacial acetic acid to form acyl azides **5a**–**b**. These azides underwent Curtius rearrangement upon heating in anhydrous toluene, forming the crucial intermediates 4-(4-isocyanato-5-aryl-1*H*-pyrazol-1-yl)benzenesulfonamides **6a**–**b**.^17^ Finally, the isocyanates mentioned reacted with the NH_2_ group of various aniline derivatives, resulting in the syntheses of 20 products with ureido linkers, namely **SH7a–t**.

The spectral and elemental analyses unequivocally confirmed the successful syntheses of the target derivatives **SH7a–t**. In the ^1^H NMR spectra, the two ureido linker’s NH protons of all 20 compounds were the most deshielded, appearing between 8.09 and 9.57 ppm. The NH proton of the secondary sulfamoyl group in compounds **SH7g** and **SH7o** was an exception, appearing beyond 12.60 ppm. Additionally, the protons of the primary sulfamoyl moiety in all compounds were presented as singlet signals, unless overlapping with other protons, showing an integration corresponding to two protons at a chemical shift between 7.38 and 7.40 ppm. All these protons disappeared from the spectra upon the addition of D_2_O due to H–D exchange. The aliphatic protons, whether from the methoxy group in compounds **7i**–**t** or any other aliphatic substitution (**SH7d**–**e** and **SH7l**–**m**), appeared in the aliphatic range of the chemical shift with the appropriate multiplicity and integration corresponding to each substituent.

Regarding the ^13^C NMR spectra, the exact number of signals for the equivalent carbon atoms in each compound, both aliphatic and aromatic, was observed within their respective ranges. High-resolution mass spectrometry (HRMS) further validated the successful syntheses of the compounds by displaying peaks corresponding to the positive or negative ions of each compound. Lastly, the more than 95% purity of all twenty compounds was verified using HPLC.

### 2.2 Biological Evaluations

#### 2.2.1 Carbonic Anhydrase Inhibition

The inhibitory activity of the compounds **SH7a–t** was investigated using a stopped-flow kinetic assay against the tumor-associated isoforms CA IX and XII.^18^ Since cytosolic CA I and II are widespread in erythrocytes and various other tissues, they are considered the primary off-target isoforms for anticancer applications of CA inhibitors and are included in this study.^19^ Thus, a structure–activity relationship can be determined using the inhibition constants (*K*_I_s) reported in Table 1, while the selectivity indices (SIs) are shown in Table 2.

(i) Generally, CA I was the least inhibited isoform (*K*_I_ = 791.7–89,140 nM), followed by CA II (*K*_I_ = 34.5–157.1 nM), while CA IX (*K*_I_ = 15.9–67.6 nM), and CA XII (*K*_I_ = 16.7–65.7 nM) exhibiting the highest inhibition. Notably, the derivatives **SH7b**, **SH7f**, **SH7j**, **SH7k**, and **SH7p–s** inhibited CA IX more potently than the standard, acetazolamide (**AAZ**).
(ii) The presence of a 4-methoxy group on the phenyl ring at position 5 of the pyrazole scaffold (**SH7i–t**) decreases the inhibition against off-targets CA I and II, with no significant effect on CA IX, while enhancing the activity toward CA XII compared to the unsubstituted derivatives (**SH7a–h**). Consequently, the subset of compounds **SH7i–t** (R = OCH_3_) is more selective for the tumor-associated isoforms CA IX and XII than derivatives **SH7a–h** (R = H), concerning the off-targets CA I and II.
(iii) Among the compounds that moderately inhibited the ubiquitous cytosolic CA I from 48.4 to 89.1 μM, **SH7f** and **SH7n** act in the high nanomolar range (*K*_I_ = 791.7 and 837.8 nM, respectively). Their better CA I activity is likely due to the zinc ion coordination of the more flexible ureido benzenesulfonamide pendants, overcoming the steric hindrance exerted by the isoform-specific residue combination His67, Phe91, and His^200^ in the narrow active site of this CA.
(iv) Except for the 4-sulfonamide substituent (**SH7f** and **SH7n**), which increased activity toward all isoforms investigated, polar substituents like 4-methoxy (**SH7d** and **SH7l**) or 4-carboxyethyl (**SH7e** and **SH7m**) reduced the activity against CA I and IX while increasing activity toward CA II and XII. This resulted in the most potent CA XII inhibitor, **SH7l** (*K*_I_ = 16.7 nM), with the highest CA XII selectivity (SI_CA I/XII_ = 4578.4; SI_CA II/XII_ = 6.2) compared to the unsubstituted derivatives **SH7a** and **SH7i**.
(v) The introduction of a fluorine atom (**SH7b** and **SH7j**) at position 4 on the ureido phenyl ring increased the inhibitory activity against all isoforms except of *h*CA XII. In contrast, substitution by chlorine (**SH7c** and **SH7k**) or bromine (**SH7p** and **SH7q**) decreased the activity against *h*CA I, II, and XII compared to the unsubstituted derivatives **SH7a** and **SH7i**. This trend was reversed for CA IX within the subset **SH7i–t** (R = OCH_3_), where lipophilic substituents enhanced the inhibition (4-Cl, 3-CF_3_ > 4-Br, 2-F > 4-Cl > 4-Br > 4-F > 3-CF_3_). This resulted in the four most potent and selective CA IX inhibitors **SH7s**: (*K*_I_ = 15.9 nM; SI_CA I/IX_ = 5053.5; SI_CA II/IX_ = 7.2), **SH7q** (*K*_I_ = 16.2 nM; SI_CA I/IX_ = 5127.2; SI_CA II/IX_ = 7.8), **SH7k** (*K*_I_ = 17.4 nM; SI_CA I/IX_ = 4674.7; SI_CA II/IX_ = 7.1), and **SH7p** (*K*_I_ = 19.3 nM; SI_CA I/IX_ = 4424.4; SI_CA II/IX_ = 8.1).

**Table 1:**
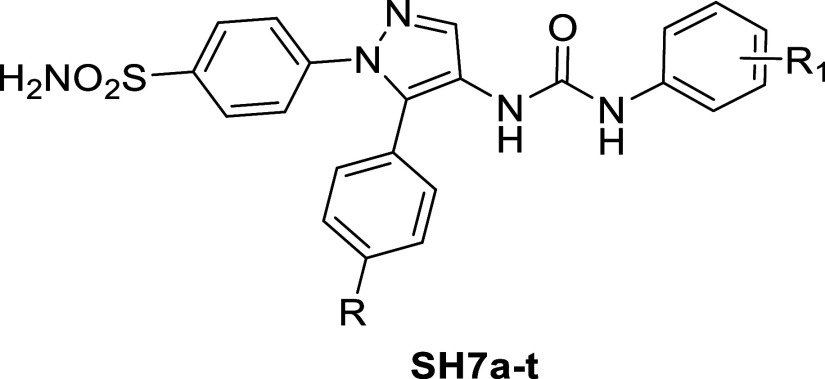
Inhibition Data of the Compounds **SH7a–t** and the Standard Sulfonamide Inhibitor Acetazolamide (**AAZ**) towards Human CA I, II, IX and XII, as Determined by Using a Stopped-Flow CO_2_ Hydrase Assay^18^

Cpd.	R	R_1_	*K*_I_ (nM)^a^			
			*h*CA I	*h*CA II	*h*CA IX	*h*CA XII
**SH7a**	H	H	51,420	61.3	33.4	42.5
**SH7b**	H	4-F	48,070	34.5	26.1	51.3
**SH7c**	H	4-Cl	57,520	65.8	39.8	59.6
**SH7d**	H	4-OCH_3_	54,180	37.6	44.5	22.2
**SH7e**	H	4-COOEt	63,810	46.2	63.2	27.5
**SH7f**	H	4-SO_2_NH_2_	791.7	54.7	24.3	39.3
**SH7g**	H	thiazol-2-yl	60,790	72.4	67.6	30.4
**SH7h**	H	4-NO_2_	52,270	38.1	37.4	49.7
**SH7i**	OCH_3_	H	73,810	96.9	34.9	35.9
**SH7j**	OCH_3_	4-F	69,580	87.3	22.1	43.4
**SH7k**	OCH_3_	4-Cl	81,340	123.4	17.4	48.8
**SH7l**	OCH_3_	4-OCH_3_	76,460	103.7	41.3	16.7
**SH7m**	OCH_3_	4-COOEt	72,770	93.5	55.7	26.9
**SH7n**	OCH_3_	4-SO_2_NH_2_	837.8	59.3	32.4	32.6
**SH7o**	OCH_3_	thiazol-2-yl	89,140	131.2	35.1	24.3
**SH7p**	OCH_3_	4-Br	85,390	157.1	19.3	57.1
**SH7q**	OCH_3_	4-Br,2-F	83,060	126.3	16.2	65.7
**SH7r**	OCH_3_	3-CF_3_	77,690	81.5	23.5	52.4
**SH7s**	OCH_3_	4-Cl,3-CF_3_	80,350	114.6	15.9	55.2
**SH7t**	OCH_3_	4-OCF_3_	74,170	86.3	29.6	19.0
**AAZ**			250.0	12.5	26.0	5.7

a Mean from three independent assays, measured by using a stopped-flow technique (errors were in the range of ±5–10% of the reported values).

**Table 2:** Selectivity Indices of the Compounds **SH7a–t**

Cpd.	SI			
	CA I/IX	CA II/IX	CA I/XII	CA II/XII
**SH7a**	1539.5	1.8	1209.9	1.4
**SH7b**	1841.8	1.3	937.0	0.7
**SH7c**	1445.2	1.7	965.1	1.1
**SH7d**	1217.5	0.8	2440.5	1.7
**SH7e**	1009.7	0.7	2320.4	1.7
**SH7f**	32.6	**2.3**	20.1	1.4
**SH7g**	899.3	1.1	1999.7	**2.4**
**SH7h**	1397.6	1.0	1051.7	0.8
**SH7i**	2114.9	**2.8**	2056.0	**2.7**
**SH7j**	3148.4	**4.0**	1603.2	**2.0**
**SH7k**	4674.7	**7.1**	1666.8	**2.5**
**SH7l**	1851.3	**2.5**	4578.4	**6.2**
**SH7m**	1306.5	1.7	2705.2	**3.5**
**SH7n**	25.9	1.8	25.7	1.8
**SH7o**	2539.6	**3.7**	3668.3	**5.4**
**SH7p**	4424.4	**8.1**	1495.4	**2.8**
**SH7q**	5127.2	**7.8**	1264.2	1.9
**SH7r**	3306.0	**3.5**	1482.6	1.6
**SH7s**	5053.5	**7.2**	1455.6	**2.1**
**SH7t**	2505.7	**2.9**	3903.7	**4.5**
**AAZ**	9.6	0.5	43.9	2.2

Interestingly, the 4-nitro group (**SH7h**) increased the activity only against CA II (*K*_I_ = 38.1 nM), while the 4-trifluoromethoxy substitution (**SH7t**) proved to be more effective than the 4-methoxy one (**SH7l**) across all investigated isoforms.

(vi) Compared to **SH7a** and **SH7i**, the presence of the thiazol-2-yl group increased the activity against CA XII, resulting in **SH7o** being the second most selective compound toward this isoform (SI_CA I/XII_ = 3668.3; SI_CA II/XII_ = 5.4).

It is worth mentioning that the adopted design approaches of this work yielded promising carbonic anhydrase inhibitors (CAIs), particularly **SH7s**, which demonstrated improved inhibitory activity against *h*CA IX compared to **SLC-0111**. Specifically, **SH7s** exhibited an approximately 3-fold higher potency against *h*CA IX than **SLC-0111**. This enhanced *h*CA IX inhibition was accompanied by increased activity against *h*CA II and decreased activity against *h*CA I compared to **SLC-0111**. As a result, **SH7s** showed lower *h*CA II/IX selectivity (SI_CA II/IX_ = 7.2) but higher *h*CA I/IX selectivity (SI_CA I/IX_ = 5053) compared to **SLC-0111**. Regarding *h*CA XII inhibition, **SH7s** displayed effective nanomolar inhibition, though not as potent as **SLC-0111**.

### 2.3 X-ray Crystallography

**Figure 2:**
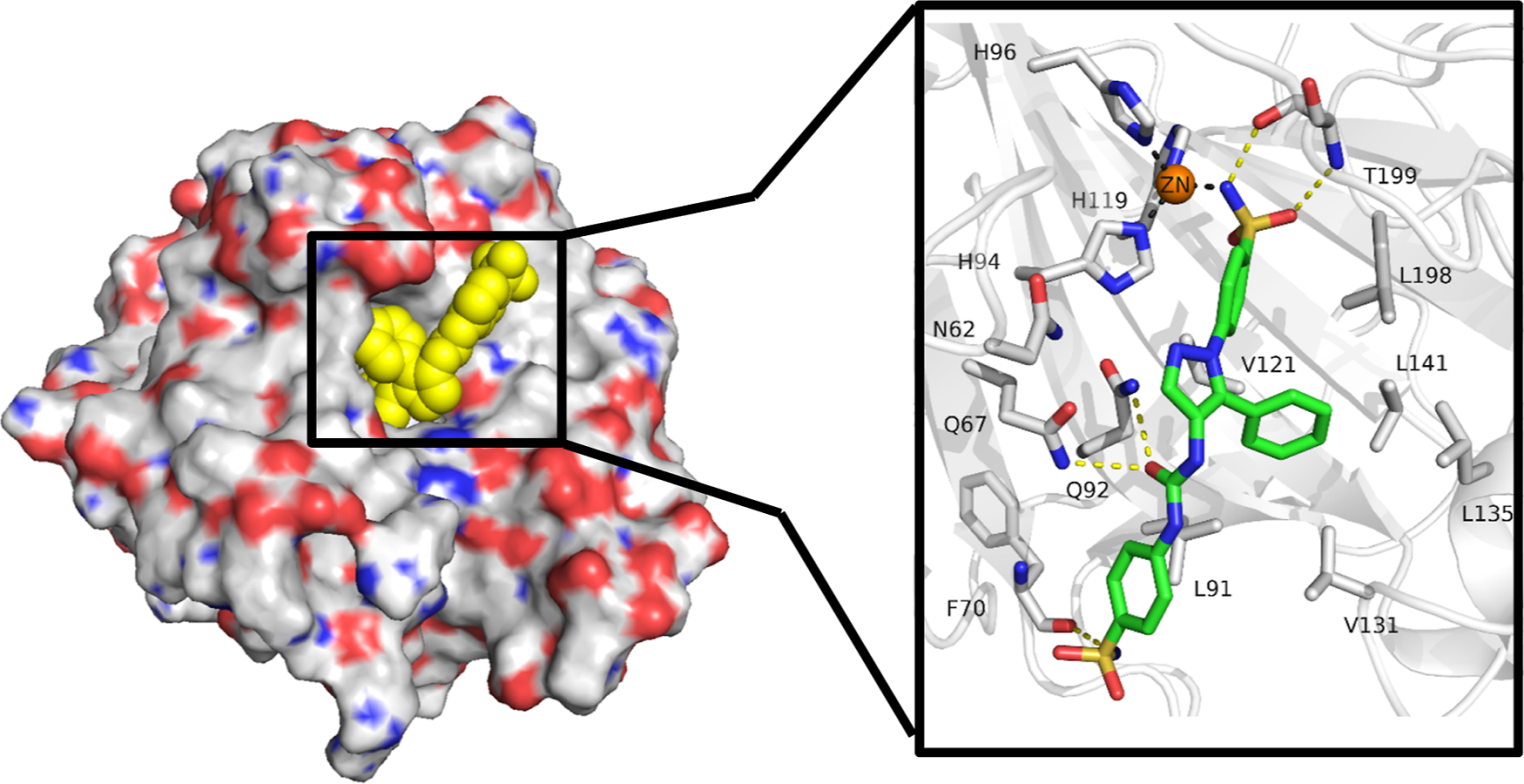
Binding mode of ligand **SH7f** as observed in the ligand–protein cocrystal of CA IX mimic (crystal structure PDB: 8TTR). The protein backbone is represented as gray cartoon, the zinc cofactor as orange sphere and the ligand as green sticks. Coordination bonds are represented as gray dashed lines and hydrogen bonds as yellow dashed lines.

In order to gain insight into the binding mode of this type of compounds, the X-ray crystal structure of compound **SH7f** cocrystallized with CA IX mimic was determined (Figure 2). The ligand shows the common interactions of a carbonic anhydrase sulfonamide inhibitor, in which the sulfonamide group is placed in the depth of the binding site, coordinating the zinc ion through the deprotonated nitrogen. A hydrogen bond between one oxygen atom of the coordinating sulfonamide group and NH of the Thr199 backbone was observed. Additionally, the NH of the sulfonamide group acts as hydrogen bond donor to the oxygen atom of Thr199 side chain. The carbonyl oxygen of the urea moiety lies in a proximity allowing it to accept hydrogen from either Gln67 or Gln92. Finally, the NH_2_ group of the terminal sulfonamide moiety forms a hydrogen bond with the backbone carbonyl oxygen atom of Phe70.

The phenyl ring bearing the coordinating sulfonamide group forms hydrophobic interactions with Val121 and Leu198, while the terminal phenyl ring shows hydrophobic interactions with Leu91. Moreover, the phenyl ring in position 5 of the pyrazole moiety is forming a hydrophobic interactions network with Leu91, Val131, Leu135, and Leu198.

### 2.4 Molecular Docking

**Figure 3:**
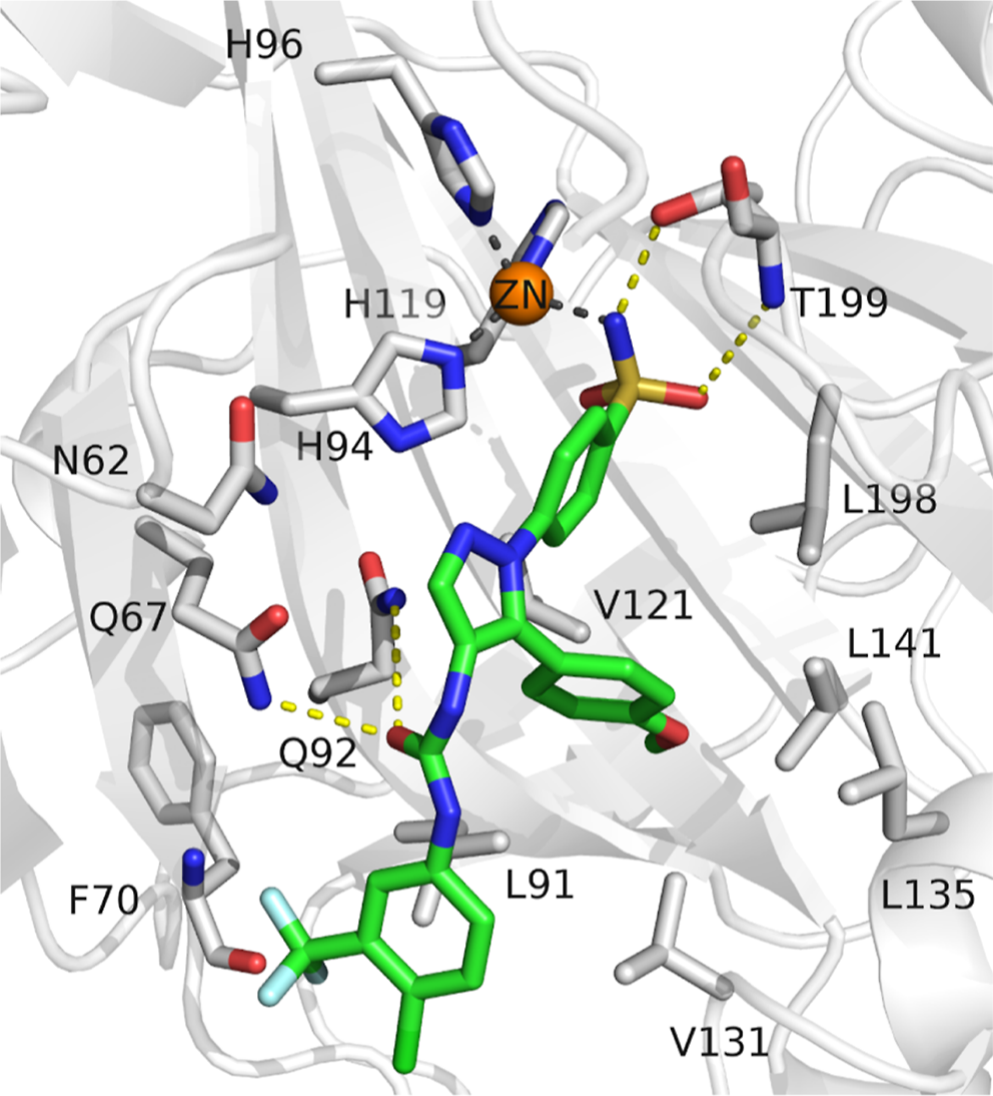
Docking pose of compound **SH7s** in the crystal structure of a CA IX mimic (crystal structure PDB: 8TTR). The protein backbone is represented as white cartoon, the zinc cofactor as orange sphere and the ligand by green sticks. Coordination bonds are represented as gray dashed lines and hydrogen bonds as yellow dashed lines.

The binding mode of the most active compound was studied by molecular docking in the CA IX mimic crystal structure (PDB: 8TTR). The obtained docking pose showed a comparable zinc chelation mode and similar interactions to that of the cocrystallized ligand, specifically the hydrogen bond formation with Thr199 and Gln67/Gln92. Additionally, the methoxy substitution on the lateral phenyl ring is deeply buried into the hydrophobic pocket, extending the hydrophobic interaction network (Figure 3).

### 2.5 In Vitro Anticancer Activity toward 60 Cancer Cell Lines (NCI, USA)

#### 2.5.1 Primary Single Dose (10^–5^ M) Screening

The ureido sulfonamides **SH7a–t** were submitted to the National Cancer Institute’s (NCI) Developmental Therapeutics Program for evaluation of their anticancer potential.^20−22^ The compounds underwent a screening against the NCI-60 Human Tumor Cell Lines panel, which consists of 60 diverse human cancer cell lines representing nine major cancer types: leukemia, lung, colon, central nervous system (CNS), melanoma, ovarian, renal, prostate, and breast cancers.

The antiproliferative assays were conducted using the sulforhodamine B (SRB) assay, a well-established method for determining cell growth and viability.^23^ In this screening, the selected compounds were tested at a single dose of 10 μM against all 60 cell lines. This comprehensive approach allows for the assessment of growth inhibitory potential across a wide range of cancer types, providing valuable insights into the compounds’ anticancer activities and potential selectivity.^23^

**Figure 4:**
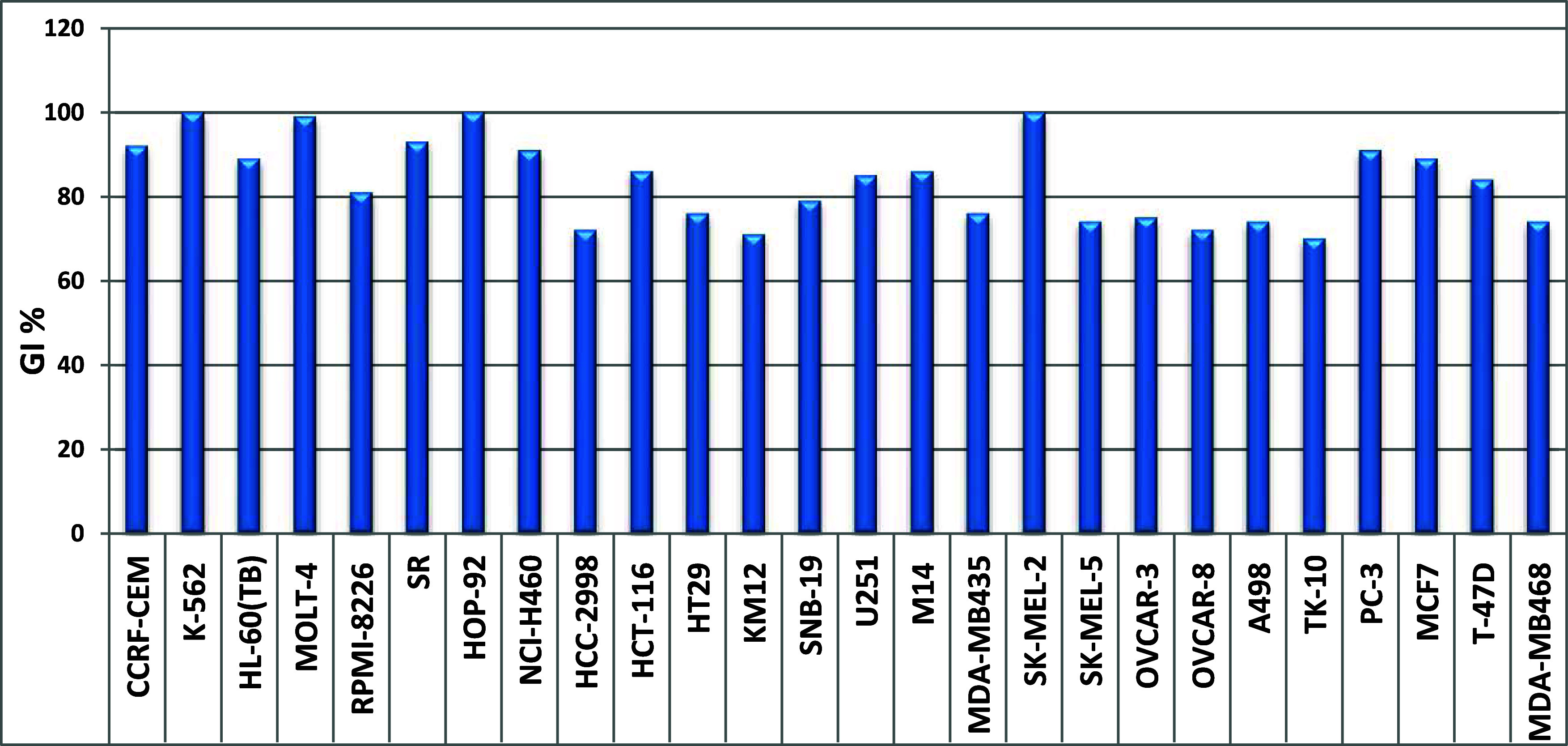
Cancer cell lines most susceptible to the impact of **SH7s** with GI values of more than 70%, after treatment with 10 μM for 48 hours.

Compound **SH7s** emerged as the most potent analogue in this assay, demonstrating broad-spectrum activity against a diverse range of cancer cell lines from various tumor subpanels. **SH7s** exhibited a mean growth inhibition value of 67% and effectively suppressed the proliferation of all examined cancer cell lines, with growth inhibition percentages ranging from 25% to 100% (Supporting Information, Figures S95 and S96). In particular, **SH7s** showed promising cell growth inhibitory activities (GI % > 85) against leukemia [CCRF-CEM, K-562, MOLT-4, HL-60(TB), RPMI-8226 and SR], non-small cell lung (NSCLC; HOP-92 and NCI-H460), colon (HCT-116), CNS (U251), melanoma (M14, SK-MEL-2), prostate (PC-3), and breast (MCF7) cancer cell lines (Figure 4).

**Figure 5:**
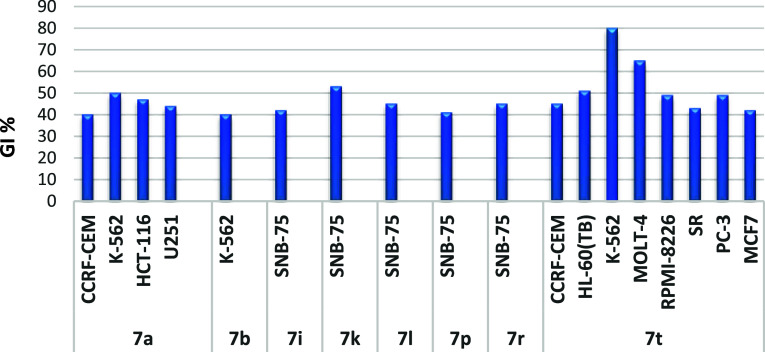
Cancer cell lines most susceptible to the impact of the target molecules (**SH7a**, **7b**, **7i**, **7k**, **7l**, **7p**, **7r**, and **7t**) with GI values of more than 40%, upon treatment with 10 μM for 48 h, with GI values of more than 40%.

On the other hand, eight compounds (**SH7a**, **7b**, **7i**, **7k**, **7l**, **7p**, **7r**, and **7t**) exhibited moderate but selective anticancer activity against specific cancer cell lines, with growth inhibition (GI) values exceeding 40%, as illustrated in Figure 5. Unfortunately, the remaining derivatives showed no significant activities in this NCI assay.

#### 2.5.2 In Vitro Five-Doses Full NCI 60 Cell Panel Screening

The preliminary screening results indicated that compound **SH7s** is the most active one in the current study, exhibiting effectiveness against various cell lines from different cancer subpanels (Tables 3 and 4). As a result, compound **SH7s** was selected for further evaluation using a five-doses screening (0.01–100 μM). The response parameters, including GI_50_, TGI, and LC_50_ against different cell lines, are summarized in Table 3. GI_50_ denotes the half-maximal growth inhibitory effect, TGI signifies cytostatic activity, and LC_50_ represents half-maximal cytotoxicity. Table 4 displays the subpanel and full panel mean graph midpoints (MG-MID) for the GI_50_ parameter, providing an average activity measure across individual subpanels and the full panel cell lines for compound **SH7s**.

**Table 3:** NCI In Vitro Testing Results Expressed as GI_50_, TGI and LC_50_ Values of Compound **SH7s**, as Determined in a Five-Doses Screening Assay

subpanel/cancer cell lines	compound **SH7s**		
	GI_50_ (μM)	TGI (μM)	LC_50_ (μM)
Leukemia			
CCRF-CEM	3.0	11.3	>100
HL-60(TB)	2.3	5.7	>100
K-562	3.1	16 0.9	>100
MOLT-4	2.0	6.0	>100
RPMI-8226	3.1	13.5	>100
SR	2.0	5.8	>100
Non-small Cell Lung Cancer (NSCLC)			
A549/ATCC	3.5	13.6	49.4
EKVX	3.8	16.4	61.7
HOP-62	4.1	16.1	43.3
HOP-92	2.0	7.3	37.5
NCI-H226	3.2	14.6	84.0
NCI-H23	3.1	12.4	43.0
NCI-H322M	3.9	14.3	41.2
NCI-H460	3.5	10.4	47.3
NCI-H522	3.0	12.3	46.1
Colon Cancer			
COLO 205	2.3	5.3	18.4
HCC-2998	3.1	8.9	32.4
HCT-116	2.0	4.2	8.7
HCT-15	3.6	14.4	42.3
HT29	3.6	10.9	43.9
KM12	3.0	11.6	57.9
SW-620	3.9	13.8	39.8
CNS Cancer			
SF-268	3.3	14.7	49.4
SF-295	2.7	10.9	36.4
SF-539	3.1	9.9	44.2
SNB-19	3.9	14.7	41.0
SNB-75	2.3	13.3	41.8
U251	3.3	12.3	39.3
Melanoma			
LOX IMVI	2.8	8.4	33.7
MALME-3M	2.5	7.7	34.0
M14	3.3	12.0	39.9
MDA-MB-435	2.8	9.6	55.4
SK-MEL-2	2.4	5.5	20.0
SK-MEL-28	3.7	13.7	55.1
SK-MEL-5	1.9	3.9	7.8
UACC-257	3.3	12.0	38.2
UACC-62	2.1	4.5	9.6
Ovarian Cancer			
IGROV1	3.7	21.3	>100
OVCAR-3	2.4	6.5	27.2
OVCAR-4	2.9	13.1	56.0
OVCAR-5	3.5	14.4	70.8
OVCAR-8	3.4	13.0	43.1
NCI/ADR-RES	24.7	>100	>100
SK-OV-3	3.7	14.0	42.9
Renal Cancer			
786-0	1.7	3.4	6.9
A498	2.8	8.3	29.3
ACHN	4.0	14.2	41.0
RXF 393	2.6	9.8	37.8
SN12C	3.6	13.1	48.9
TK-10	3.9	15.3	45.3
UO-31	3.4	15.1	41.8
Prostate Cancer			
PC-3	2.9	11.4	77.1
DU-145	3.5	12.1	35.6
Breast Cancer			
MCF7	2.7	9.2	49.1
MDA-MB-231/ATCC	2.0	4.7	13.4
HS 578T	3.4	16.9	>100
BT-549	1.8	4.4	16.1
T-47D	2.7	12.4	51.2
MDA-MB-468	2.1	6.9	32.3

**Table 4:** Median In Vitro Growth Inhibitory Concentrations (GI_50_, μM) of **SH7s** towards Cancer Cell Line Subpanels

subpanel/tumor cell lines	**SH7s**	
	MG-MID (μM)^a^	selectivity index
leukemia	2.6	1.35
non-small cell lung cancer	3.6	1.05
colon cancer	3.1	1.13
CNS cancer	3.1	1.13
melanoma	2.8	1.27
ovarian cancer	6.3	0.55
renal cancer	3.1	1.12
prostate cancer	3.2	1.11
breast cancer	2.4	1.43
full panel MG-MID^b^	3.5	

a Median value calculated according to the data obtained from NCI’s in vitro disease-oriented human tumor cell screen.

b GI_50_ (μM) full panel mean-graph midpoint (MG-MID) = the average sensitivity of all cell lines toward the test agents.

Compound **SH7s** exhibited potent growth inhibitory activity at low micromolar concentrations against almost the entire panel of cancer cell lines (GI_50_ range = 1.7–4.1 μM) except for NCI/ADR-RES (GI_50_ = 24.7 μM) (see Table 3). The highest activity was observed against renal cancer (786-0), breast cancer (BT-549), and melanoma (SK-MEL-5) cells with GI_50_ values of 1.7, 1.8, and 1.9 μM, respectively. Followed by colon cancer (HCT-116), leukemia (SR and MOLT-4), NSCLC (HOP-92), and breast cancer (MDA-MB-231) cells, all showing GI_50_ values of 2.0 μM. Compound **SH7s** exhibited a strong cytostatic effect against the majority of cell lines, with TGI ranging from 3.4 to 16.9 μM, except for IGROV1 (TGI > 21.3 μM) and NCI/ADR-RES (TGI > 100 μM) (see Table 3).

Overall, compound **SH7s** displayed a broad-spectrum antiproliferative effect throughout the entire NCI panel, with an effective growth inhibition full panel GI_50_ (MG-MID) value of 3.5 μM and a subpanel GI_50_ (MG-MID) range of 2.4–6.3 μM. Of the cancer subpanels, breast cancer and leukemia lines were most susceptible to the impact of **SH7s** [GI_50_ (MG-MID) = 2.4 and 2.6 μM, respectively] (see Table 4), followed by the melanoma, colon cancer, CNS cancer, and renal cancer subpanels [GI_50_ (MG-MID) = 3.1 μM].

The selectivity index, which is a calculated measure of a compound’s selectivity comparing two targets or cell lines (subpanels), was determined by dividing the full panel MG-MID (μM) of the compounds by their individual subpanel MG-MID (μM). Compound **SH7s** demonstrated a broad-spectrum anticancer activity against all tumor subpanels tested at the GI_50_ level, hence, with relatively low selectivity indices ranging from 0.55 to 1.43 throughout the cancers panel (see Table 4).

Moreover, compound **SH7s** exhibited a non-lethal, but cytostatic effect in the leukemia subpanel, and on the ovarian cancer cells IGROV1 and NCI/ADR-RES, and the breast cancer cells HS 578T, where LC_50_ values were >100 μM. Most of the cell lines showed LC_50_ values >10 times their GI_50_, indicating the relative non-lethal effect of **SH7s** against most of them.

#### 2.5.3 Anticancer Activity of **SH7s** Alone and as Adjuvant in Cotreatment with **Taxol** and **5-FU**

The impact of one of the most promising derivatives of this study, namely **SH7s**, on the viability and proliferation of human cancer cells was investigated in more detail by using the colorectal cancer cell line HCT-116 and conducting a fluorometric resazurin-based cell viability assay. Alongside **SH7s**, the established CA inhibitor **SLC-0111**, was tested in parallel as a reference.^24^ Initially, both compounds were tested as single treatments for 48 h, pointing out an appreciably higher cytotoxic effect of **SH7s** compared to **SLC-0111**, indicated by their relative IC_50_ values of 13.1 and >100 μM, as shown in (Supporting Information, Figure S92).

Subsequently, both compounds were tested for their potency as adjuvants for drug efficacy-boosting cotreatments of the two clinically approved anticancer drugs Taxol (paclitaxel) and 5-fluorouracil (5-FU), under both normoxic and hypoxic cell cultivation conditions. For that purpose, and again under both cultivation conditions, dose–response curves of Taxol and 5-FU as single-treatments and as cotreatments with fixed concentrations of **SH7s** (10 μM) and **SLC-0111** (100 μM) were measured, and finally the resulting IC_50_ values of the single- and cotreatments were compared. The fixed cotreatment concentrations of both CA inhibitors were selected based on the aforementioned dose–response curves of their single-treatments, whereby the 10 and 100 μM represent approximately the IC_20_ values of **SH7s** and **SLC-0111**, respectively, toward the HCT-116 cancer cells. Ensuring an observable, but not excessive antiproliferative effect of the CA inhibitors themselves, we will detect their adjuvant impact on both drug treatments by leaving a measuring window large enough to unequivocal detection.

**Figure 6:**
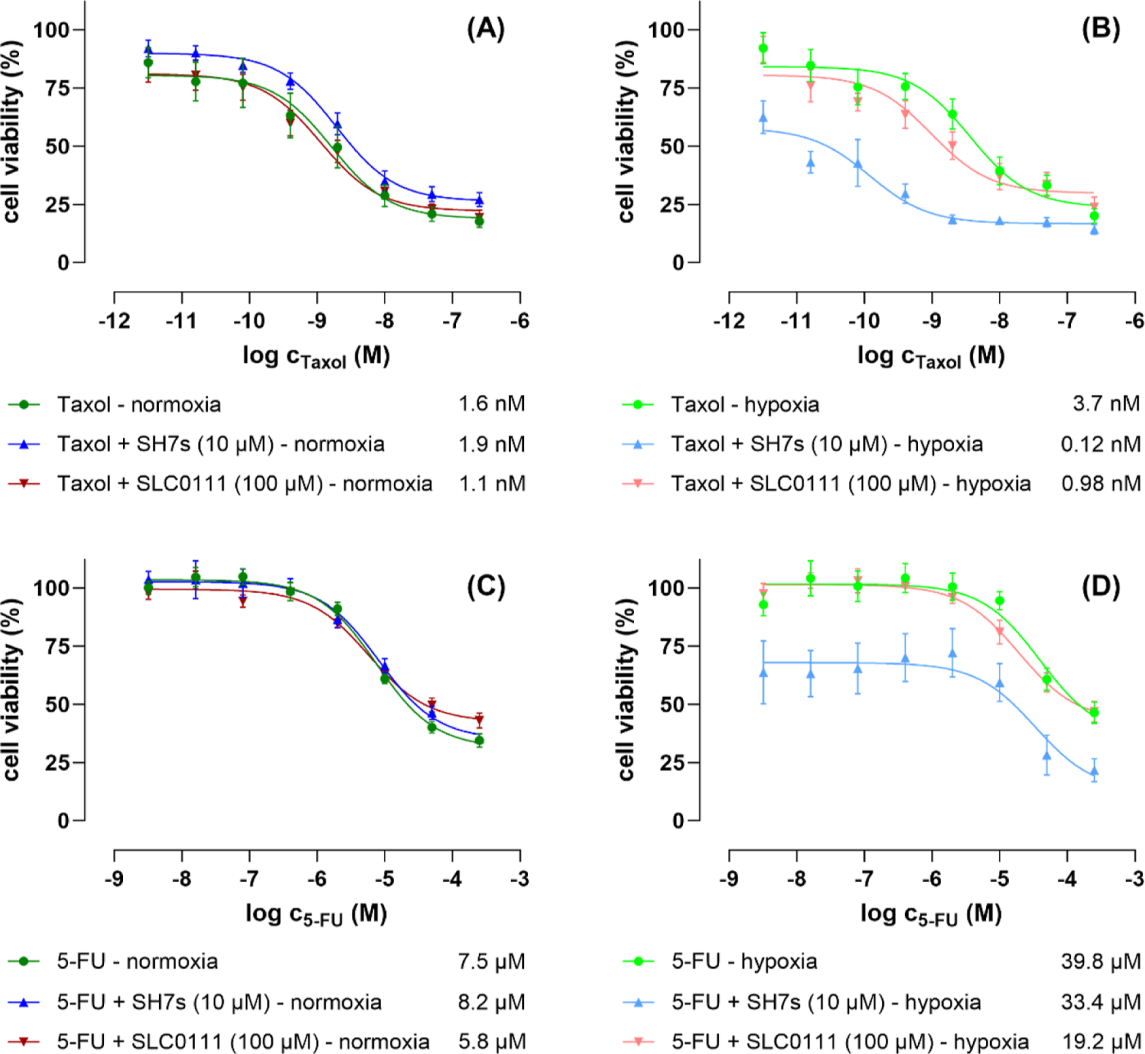
Dose–response curves of the antiproliferative/cytotoxic effects of the clinically approved anticancer drugs Taxol (paclitaxel) (A,B) and 5-fluorouracil (5-FU) (C,D) on human HCT-116 colorectal cancer cells, measured after 48 h single treatment and cotreatments with **SH7s** (fixed concentration 10 μM) and **SLC-0111** (fixed concentration 100 μM), under both normoxic (A,C) and hypoxic (B,D) cell culture conditions, by conducting fluorometric resazurin-based cell viability assays. The data represent three biological replicates each comprising technical triplicates. The given values are relative IC_50_ values, the standard errors are expressed as ±SEM. Data analysis was done by using GraphPad Prism 10.1 software.

Thus, the following outcomes can be summarized, as illustrated in Figure 6:

(i) Taxol as a single-treatment was more than 2-times less effective under hypoxic conditions (IC_50_ = 3.7 nM) compared to normoxic conditions (IC_50_ = 1.6 nM).
(ii) Whereas under normoxic conditions, neither the derivative **SH7s** (with 10 μM) nor the reference CA inhibitor **SLC-0111** (with 100 μM) significantly influenced the effect of Taxol (Figure 6A). Both compounds though improved the effect of Taxol under hypoxic conditions. With the drug as single-treatment having an IC_50_ = 3.7 nM, the cotreatment with **SH7s** improved the activity 30-fold (IC_50_ = 0.12 nM), and **SLC-0111** at least ∼4-fold (IC_50_ = 0.98 nM). Furthermore, contrary to **SLC-0111**, the derivative **SH7s** shifted the IC_50_ curve of Taxol to the left, improving the IC_50_ value, and also strongly reducing the number of viable cancer cells even at lower Taxol concentrations (Figure 6B).
(iii) 5-FU as a single-treatment was ∼8-times less effective under hypoxic conditions (IC_50_ = 39.8 μM) compared to normoxic conditions (IC_50_ = 7.5 μM).
(iv) Under normoxic conditions, neither the derivative **SH7s** (at 10 μM) nor the reference CA inhibitor **SLC-0111** (at 100 μM) significantly affected 5-FU (Figure 6C). **SLC-0111** also had little effect under hypoxic conditions, whereas the derivative **SH7s** at 10 μM improved the effect of 5-FU under hypoxic conditions. 5-FU as single treatment had an IC_50_ = 39.8 nM, and in cotreatment with **SH7s** a very similar IC_50_ = 33.4 nM. The major impact of **SH7s** cotreatment with 5-FU, however, resulted in a strongly reduced number of viable cancer cells over the whole range of 5-FU concentrations (Figure 6D).

Taken together, the novel CA inhibitor derivative **SH7s**, in contrast to the published CA reference inhibitor **SLC-0111**, is substantially more active toward HCT-116 colorectal cancer cells (IC_50_ = 13.1 μM) even as a single-treatment. These compounds, when coapplied at their ∼IC_20_ concentration (10 μM), also adjuvantly enhanced the anticancer effects of 5-FU but especially Taxol under hypoxic conditions, which are of far more relevant for solid tumors than normoxic conditions.

### 2.6 Kinase Profiling

**Figure 7:**
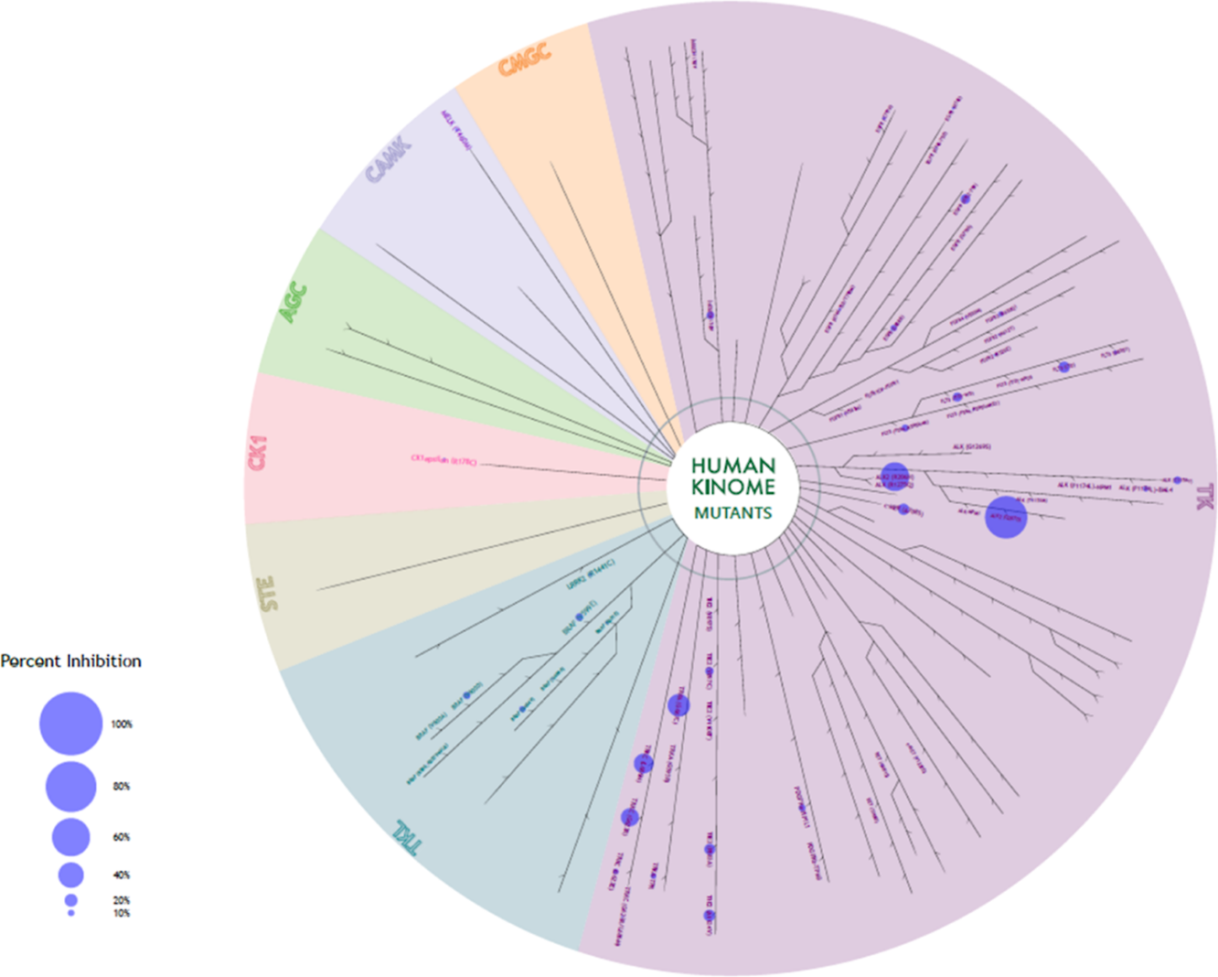
Human kinase array results for compound **SH7s**.

To exclude the off-targets of the best candidate (**SH7s**), the compound was submitted for kinase profiling against 258 kinases (mutant strains) conducted by Reaction Biology (Freiburg, Germany). The screening assay was done at a concentration of 20 μM, and the results are expressed by mean % inhibition (more details in the Supporting Information, Table S1). The compound was inactive against the entire kinase panel except for two mutated forms of the activin receptor-like kinase 2 (ALK2), namely ALK2 (R1275Q), with 67% inhibition and fALK2(R206H) with 45% (Figure 7, Supporting Information, Table S1). Based on these results, we can exclude most of these kinases as potential off-targets of **SH7s**.

## 3 Conclusions

A series of 4-(pyrazol-1-yl)benzenesulfonamide ureas (**SH7a–t**) was synthesized to be investigated as inhibitors of tumor-associated carbonic anhydrases. All compounds displayed a low nM inhibition toward CA IX and CA XII. X-ray crystallography of **SH7f**, as a representative from this series, with a CA IX mimic was done to explore its interaction in the active site and to confirm the concept. Most of the derivatives were subjected to cancer cell viability screenings against a panel of 60 cell lines at NCI-USA. Among them, compound **SH7s** turned out as the most promising candidate, hence, it was investigated in more detail under both normoxic and hypoxic conditions after excluding off-targets through a kinase assay profiling against 256 kinases. The coadministration of **SH7s** with Taxol against a colorectal cancer cell line (HCT-116), as a representative of solid tumors which are affected by hypoxic conditions, showed a significant sensitizing effect of **SH7s**, enhancing the Taxol effect up to 30-fold, an effect that promises positive effects also *in vivo*.^25^ Accordingly, **SH7s** can be considered a very promising candidate for chemo-sensitization of colorectal cancers.

## 4 Experimental Section

### 4.1 Chemistry

#### 4.1.1 General

Melting points were determined utilizing the Electrothermal IA-9000 apparatus and have been reported without correction. The ^1^H NMR and ^13^C NMR spectra were recorded by Bruker 400 MHz spectrometer (400 MHz for ^1^H and 101 MHz for ^13^C NMR). Deuterated dimethyl sulfoxide (DMSO-*d*_6_) was used as a solvent in all samples. The reaction progression was observed via TLC using Merck’s silica gel on aluminum sheets 60 F254. The HPLC consists of an XTerra RP18 column (3.5 μm, 3.9 mm × 100 mm) from the manufacturer Waters (Milford, MA, USA) and two LC-10AD pumps, a SPD-M10A VP PDA detector, and a SIL-HT autosampler, all from the manufacturer Shimadzu (Kyoto, Japan). Mass spectrometry analyses were performed with a Finnigan MAT710C (Thermo Separation Products, San Jose, CA, USA) for the ESIMS spectra and with a LTQ (linear ion trap) Orbitrap XL hybrid mass spectrometer (Thermo Fisher Scientific, Bremen, Germany) for the HRMS-ESI (high resolution mass spectrometry) spectra. All the approved final compounds had a purity ≥95%.

#### 4.1.2 Synthesis of Ethyl 2-Benzoyl-3-(dimethylamino)acrylate Derivatives (**2a**–**b**)

Ethyl 3-oxo-3-arylpropanoates **1a**–**b** (17.5 mmol) were heated to reflux with DMF-DMA (8 mL) for 1 h. After that, the reaction mixture was subjected to evaporation under vacuum, leaving a residue that was washed with diethyl ether (3 × 3 mL), yielding the enaminones **2a**–**b**.^26^

##### 4.1.2.1 Ethyl 2-Benzoyl-3-(dimethylamino)acrylate

White crystals, yield 83%, mp 155–157 °C (reported mp 154–156 °C).^26^

##### 4.1.2.2 Ethyl 3-(Dimethylamino)-2-(4-methoxybenzoyl)acrylate

White crystals, yield 80%, bp 82–83 °C (reported bp 79–80 °C).^27^

#### 4.1.3 Synthesis of Ethyl 5-Aryl-1-(4-sulfamoylphenyl)-1*H*-pyrazole-4-carboxylates (**3a**–**b**)

The enaminones **2a**–**b** (10.5 mmol) were dissolved in 10 mL of absolute ethanol, and 4-hydrazinylbenzenesulfonamide hydrochloride (10.5 mmol, 4.70 g) was added before this mixture being refluxed for 2 h. Upon cooling to room temperature, the solid formed was filtered and washed with *n*-hexane (3 × 3 mL), resulting in the desired intermediates, **3a**–**b**.^26^

##### 4.1.3.1 Ethyl 5-Phenyl-1-(4-sulfamoylphenyl)-1*H*-pyrazole-4-carboxylate

White crystals, yield 78%, mp 184–185 °C (reported mp 186–187 °C).^28^

##### 4.1.3.2 Ethyl 5-(4-Methoxyphenyl)-1-(4-sulfamoylphenyl)-1*H*-pyrazole-4-carboxylate

White crystals, yield 81%, mp 156–158 °C (reported mp 158–159 °C).^28^

#### 4.1.4 Synthesis of 4-(4-(Hydrazinecarbonyl)-5-aryl-1*H*-pyrazol-1-yl)benzenesulfonamides (**4a**–**b**)

Esters **3a**–**b** (7 mmol) were refluxed for 3 h in hydrazine hydrate 95% (8 mL), with the reaction progress monitored by thin-layer chromatography (TLC). Once the reaction was complete, the mixture was poured over ice with continuous stirring, and a small amount of acetic acid was added. The resulting precipitate was filtered, washed with ethanol (2 × 2 mL) and diethyl ether (2 × 3 mL), and dried to yield hydrazides **4a**–**b** that were used in the next step without further purification.^29^

#### 4.1.5 Synthesis of 5-Aryl-1-(4-sulfamoylphenyl)-1*H*-pyrazole-4-carbonyl Azides (**5a**–**b**)

An ice bath maintained at 0–5 °C was used in this reaction. In this bath, a solution of sodium nitrite (0.55 g, 8 mmol) in water was added to a solution of a chosen hydrazide **4a**–**b** (3.7 mmol) in glacial acetic acid (3 mL). The mixture was stirred for 45 min before the precipitate formed was filtered, washed with cold water (3 × 5 mL) and *n*-hexane (3 × 2 mL), and air-dried. The resulting azides **5a**–**b** were then used in the subsequent reaction without any additional purification.^30^

#### 4.1.6 Synthesis of 4-(5-Aryl-4-(3-arylureido)-1*H*-pyrazolyl)benzenesulfonamides (**SH7a–t**)

A chosen azide derivative **5a**–**b** (0.22 mmol) was heated to reflux in dry toluene (5 mL) for 45 min to produce the matching isocyanate **6a**–**b**. Subsequently, a suitable aniline derivative (0.22 mmol) was introduced into the produced isocyanate solution, followed by refluxing the mixture for a duration of 2 h. The solid that was generated was separated by filtration, then washed with hot toluene (2 × 3 mL) and *n*-hexane (3 × 2 mL), dried, and finally crystallized from ethanol to obtain the matching urea-tethered products **SH7a–t**.^9^

##### 4.1.6.1 4-(5-Phenyl-4-(3-phenylureido)-1*H*-pyrazolyl)benzenesulfonamide (**SH7a**)

White crystals (yield 80%); mp 205–207 °C; HPLC purity: 98.9%; ^1^H NMR (500 MHz, DMSO-*d*_6_): δ ppm 6.91 (t, 1H, H-4 of C_6_H_5_, J = 7.3 Hz), 7.22 (t, 2H, H-3 and H-5 of C_6_H_5_, J = 7.7 Hz), 7.28 (d, 2H, H-2 and H-6 of C_6_H_5_, J = 7.3 Hz), 7.32 (d, 2H, H-3 and H-5 of 4-SO_2_NH_2_–C_6_H_4_, J = 8.5 Hz), 7.38 (m, 4H, H-2 and H-6 of C_6_H_5_ and SO_2_NH_2_ (D_2_O-exchangeable)), 7.42–7.51 (m, 3H, H-3, H-4, and H-5 of C_6_H_5_), 7.73 (d, 2H, H-2 and H-6 of 4-SO_2_NH_2_–C_6_H_4_, J = 8.4 Hz), 7.92 (s, 1H, H-3 of C_3_H_1_N_2_), 8.15 (s, 1H, NH (D_2_O-exchangeable)), 8.86 (s, 1H, NH (D_2_O-exchangeable)); ^13^C NMR (101 MHz, DMSO-*d*_6_): δ ppm 118.41, 122.30, 122.84, 124.66, 127.08, 128.82, 129.37, 129.51, 129.70, 130.37, 131.94, 135.97, 140.24, 142.50, 142.59, 153.42 (C=O); Anal. Calcd for C_22_H_19_N_5_O_3_S (433.49 g/mol): C, 60.96; H, 4.42; N, 16.16; found C, 61.04; H, 61.04; N, 16.12; HRMS (ESI) for C_22_H_20_N_5_O_3_S, calcd 434.1281, found 434.1285 [M + H]^+^, for C_22_H_19_N_5_NaO_3_S, calcd 456.1101, found 456.1105 [M + Na]^+^, for C_22_H_18_N_5_O_3_S, calcd 432.1136, found 432.1134 [M – H]^−^, and for C_22_H_19_ClN_5_O_3_S, calcd 468.0903, found 468.0899 [M + Cl]^−^.

##### 4.1.6.2 4-(4-(3-(4-Fluorophenyl)ureido)-5-phenyl-1*H*-pyrazolyl)benzenesulfonamide (**SH7b**)

White crystals (yield 78%); mp 217–218 °C; HPLC purity: 97.9%; ^1^H NMR (400 MHz, DMSO-*d*_6_): δ ppm 7.08 (t, 2H, H-3 and H-5 of 4-F–C_6_H_4_, J = 8.8 Hz), 7.29 (dd, 2H, H-2 and H-6 of 4-F–C_6_H_4_, J = 8.0, 1.4 Hz), 7.34 (d, 2H, H-3 and H-5 of 4-SO_2_NH_2_–C_6_H_4_, J = 8.8 Hz), 7.39 (s, 2H, SO_2_NH_2_ (D_2_O-exchangeable)), 7.40–7.50 (m, 5H, C_6_H_5_), 7.75 (d, 2H, H-2 and H-6 of 4-SO_2_NH_2_–C_6_H_4_, J = 8.8 Hz), 7.89 (s, 1H, H-3 of C_3_H_1_N_2_), 8.13 (s, 1H, NH (D_2_O-exchangeable)), 8.87 (s, 1H, NH (D_2_O-exchangeable)); ^13^C NMR (101 MHz, DMSO-*d*_6_): δ ppm 115.62, 115.84, 120.04, 120.11, 122.64, 124.57, 126.98, 128.74, 129.41, 129.58, 130.26, 132.20, 136.04, 136.49, 136.51, 142.41, 142.55, 153.45, 156.52, 158.80; Anal. Calcd for C_22_H_18_FN_5_O_3_S (451.48 g/mol): C, 58.53; H, 4.02; N, 15.51; found C, 58.64; H, 4.01; N, 15.49; HRMS (ESI) for C_22_H_19_FN_5_O_3_S, calcd 452.1187, found 452.1186 [M + H]^+^, and for C_22_H_18_FN_5_NaO_3_S, calcd 474.1007, found 474.1010 [M + Na]^+^, for C_22_H_17_FN_5_O_3_S, calcd 450.1042, found 450.1039 [M – H]^−^, and for C_22_H_18_ClFN_5_O_3_S, calcd 486.0808, found 486.0801 [M + Cl]^−^.

##### 4.1.6.3 4-(4-(3-(4-Chlorophenyl)ureido)-5-phenyl-1*H*-pyrazolyl)benzenesulfonamide (**SH7c**)

White crystals (yield 83%); mp 231–233 °C; HPLC purity: 99.2%; ^1^H NMR (500 MHz, DMSO-*d*_6_): δ ppm 7.24–7.30 (m, 4H, 4-Cl–C_6_H_4_), 7.32 (d, 2H, H-3 and H-5 of 4-SO_2_NH_2_–C_6_H_4_, J = 8.6 Hz), 7.40 (s, 2H, SO_2_NH_2_ (D_2_O-exchangeable)), 7.40–7.48 (m, 5H, C_6_H_5_), 7.73 (d, 2H, H-2 and H-6 of 4-SO_2_NH_2_–C_6_H_4_, J = 8.6 Hz), 7.95 (s, 1H, H-3 of C_3_H_1_N_2_), 8.13 (s, 1H, NH (D_2_O-exchangeable)), 8.98 (s, 1H, NH (D_2_O-exchangeable)); ^13^C NMR (101 MHz, DMSO-*d*_6_): δ ppm 119.96, 122.60, 124.69, 125.77, 127.09, 128.78, 129.19, 129.54, 129.70, 130.36, 132.24, 136.08, 139.25, 142.47, 142.64, 153.35 (C=O); Anal. Calcd for C_22_H_18_ClN_5_O_3_S (467.93 g/mol): C, 56.47; H, 3.88; N, 14.97; found C, 56.34; H, 3.9; N, 15.01; HRMS (ESI) for C_22_H_19_ClN_5_O_3_S, calcd 468.0892, found 468.0889 [M + H]^+^, and for C_22_H_18_ClN_5_NaO_3_S, calcd 490.0711, found 490.0708 [M + Na]^+^, for C_22_H_17_ClN_5_O_3_S, calcd 466.0746, found 466.0742 [M – H]^−^, and for C_22_H_18_Cl_2_N_5_O_3_S, calcd 502.0513, found 502.0509 [M + Cl]^−^.

##### 4.1.6.4 4-(4-(3-(4-Methoxyphenyl)ureido)-5-phenyl-1*H*-pyrazolyl)benzenesulfonamide (**SH7d**)

White crystals (yield 73%); mp 224–225 °C; HPLC purity: 98.9%; ^1^H NMR (400 MHz, DMSO-*d*_6_): δ ppm 3.69 (s, 3H, OCH_3_), 6.83 (d, 2H, H-3 and H-5 of 4-OCH_3_–C_6_H_4_, J = 7.2 Hz), 7.28–7.32 (m, 5H, C_6_H_5_), 7.33 (d, 2H, H-3 and H-5 of 4-SO_2_NH_2_–C_6_H_4_, J = 8.8 Hz), 7.38 (s, 2H, SO_2_NH_2_ (D_2_O-exchangeable)), 7.47 (d, 2H, H-2 and H-6 of 4-OCH_3_–C_6_H_4_, J = 7.6 Hz), 7.74 (d, 2H, H-2 and H-6 of 4-SO_2_NH_2_–C_6_H_4_, J = 8.8 Hz), 7.81 (s, 1H, H-3 of C_3_H_1_N_2_), 8.14 (s, 1H, NH (D_2_O-exchangeable)), 8.66 (s, 1H, NH (D_2_O-exchangeable)); ^13^C NMR (101 MHz, DMSO-*d*_6_): δ ppm 55.61 (OCH_3_), 114.45, 120.01, 122.93, 124.50, 126.97, 128.81, 129.36, 129.58, 130.26, 132.00, 133.24, 135.94, 142.44, 142.49, 153.51, 154.82; Anal. Calcd for C_23_H_21_N_5_O_4_S (463.51 g/mol): C, 59.60; H, 4.57; N, 15.11; found C, 59.42; H, 4.59; N, 15.17; HRMS (ESI) for C_23_H_22_N_5_O_4_S, calcd 464.1387, found 464.1389 [M + H]^+^, and for C_23_H_21_N_5_NaO_4_S, calcd 486.1206, found 486.1210 [M + Na]^+^.

##### 4.1.6.5 Ethyl 4-(3-(5-Phenyl-1-(4-sulfamoylphenyl)-1*H*-pyrazol-4-yl)ureido)benzoate (**SH7e**)

White crystals (yield 80%); 195–197 °C; HPLC purity: 98.9%; ^1^H NMR (400 MHz, DMSO-*d*_6_): δ ppm 1.28 (t, 3H, O–CH_2_–CH_3_, J = 7.2 Hz), 4.25 (q, 2H, O–CH_2_–CH_3_, J = 7.2 Hz), 7.30 (d, 2H, H-2 and H-6 of C_6_H_5_, J = 7.6 Hz), 7.35 (d, 2H, H-3 and H-5 of 4-SO_2_NH_2_–C_6_H_4_, J = 8.8 Hz), 7.39 (s, 2H, SO_2_NH_2_ (D_2_O-exchangeable)), 7.45–7.50 (m, 3H, H-3, H-4, and H-5 of C_6_H_5_), 7.53 (d, 2H, H-2 and H-6 of 4-CO_2_C_2_H_5_–C_6_H_4_, J = 8.8 Hz), 7.75 (d, 2H, H-2 and H-6 of 4-SO_2_NH_2_–C_6_H_4_, J = 8.8 Hz), 7.85 (d, 2H, H-3 and H-5 of 4-CO_2_C_2_H_5_–C_6_H_4_, J = 8.8 Hz), 8.05 (s, 1H, H-3 of C_3_H_1_N_2_), 8.17 (s, 1H, NH (D_2_O-exchangeable)), 9.27 (s, 1H, NH (D_2_O-exchangeable)); ^13^C NMR (101 MHz, DMSO-*d*_6_): δ ppm 14.69 (O–CH_2_–CH_3_), 60.72 (O–CH_2_–CH_3_), 117.50, 122.34, 123.12, 124.61, 126.99, 128.63, 129.48, 129.61, 130.29, 130.85, 132.20, 135.88, 142.36, 142.62, 144.72, 152.98, 165.86 (C=O); Anal. Calcd for C_25_H_23_N_5_O_5_S (505.55 g/mol): C, 59.40; H, 4.59; N, 13.85; found C, 59.41; H, 4.60; N, 13.81; HRMS (ESI) for C_25_H_24_N_5_O_5_S, calcd 506.1493, found 506.1491 [M + H]^+^, and for C_25_H_23_N_5_NaO_5_S, calcd 528.1312, found 528.1311 [M + Na]^+^, for C_25_H_22_N_5_O_5_S, calcd 504.1347, found 504.1345 [M – H]^−^, and for C_25_H_23_ClN_5_O_5_S, calcd 540.1114, found 540.1110 [M + Cl]^−^.

##### 4.1.6.6 4-(3-(5-Phenyl-1-(4-sulfamoylphenyl)-1*H*-pyrazol-4-yl)ureido)benzenesulfonamide (**SH7f**)

White crystals (yield 79%); mp 273–275 °C; HPLC purity: 97.1%; ^1^H NMR (500 MHz, DMSO-*d*_6_): δ ppm 7.18 (s, 2H, SO_2_NH_2_ (D_2_O-exchangeable)), 7.28 (d, 2H, H-2 and H-6 of C_6_H_5_, J = 7.5 Hz), 7.33 (d, 2H, H-3 and H-5 of 4-SO_2_NH_2_–C_6_H_4_, J = 8.4 Hz), 7.39 (s, 2H, SO_2_NH_2_ (D_2_O-exchangeable)), 7.42–7.49 (m, 3H, H-3, H-4, H-5 of C_6_H_5_), 7.54 (d, 2H, H-3′ and H-5′ of 4′-SO_2_NH_2_–C_6_H_4_, J = 8.6 Hz), 7.67 (d, 2H, H-2′ and H-6′ of 4′-SO_2_NH_2_–C_6_H_4_, J = 8.5 Hz), 7.73 (d, 2H, H-2 and H-6 of 4-SO_2_NH_2_–C_6_H_4_, J = 8.4 Hz), 8.06 (s, 1H, H-3 of C_3_H_1_N_2_), 8.15 (s, 1H, NH (D_2_O-exchangeable)), 9.24 (s, 1H, NH (D_2_O-exchangeable)); ^13^C NMR (101 MHz, DMSO-*d*_6_): δ ppm 117.73, 122.44, 124.71, 127.10, 127.40, 128.70, 129.59, 129.72, 130.38, 135.97, 137.29, 142.44, 142.68, 143.33, 153.16 (C=O); Anal. Calcd for C_22_H_20_N_6_O_5_S_2_ (512.56 g/mol): C, 51.55; H, 3.93; N, 16.40; found C, 51.56; H, 3.95; N, 16.43; HRMS (ESI) for C_22_H_21_N_6_O_5_S_2_, calcd 513.1009, found 513.1009 [M + H]^+^, and for C_22_H_20_N_6_NaO_5_S_2_, calcd 535.0829, found 535.0829 [M + Na]^+^, for C_22_H_19_N_6_O_5_S_2_, calcd 511.0864, found 511.0857 [M – H]^−^, and for C_22_H_20_ClN_6_O_5_S_2_, calcd 547.0631, found 547.0622 [M + Cl]^−^.

##### 4.1.6.7 4-(3-(5-Phenyl-1-(4-sulfamoylphenyl)-1*H*-pyrazol-4-yl)ureido)-*N*-(thiazol-2-yl)benzenesulfonamide (**SH7g**)

White crystals (yield 78%); mp 261–262 °C; HPLC purity: 96.0%; ^1^H NMR (400 MHz, DMSO-*d*_6_): δ ppm 6.79 (d, 1H, H-5 of C_3_H_2_NS, J = 4.6 Hz), 7.22 (d, 1H, H-4 of C_3_H_2_NS, J = 4.6 Hz), 7.27–7.32 (d, 2H, H-2 and H-6 of C_6_H_5_, J = 7.4 Hz), 7.34 (d, 2H, H-3 and H-5 of 4-SO_2_NH_2_–C_6_H_4_, J = 8.7 Hz), 7.39 (s, 2H, SO_2_NH_2_ (D_2_O-exchangeable)), 7.43–7.50 (m, 3H, H-3, H-4 and H-5 of C_6_H_5_), 7.53 (d, 2H, H-2 and H-6 of 4-SO_2_NH(C_3_H_2_NS)–C_6_H_4_, J = 8.8 Hz), 7.68 (d, 2H, H-3 and H-5 of 4-SO_2_NH(C_3_H_2_NS)–C_6_H_4_, J = 8.8 Hz), 7.75 (d, 2H, H-2 and H-6 of 4-SO_2_NH_2_–C_6_H_4_, J = 8.7 Hz), 8.04 (s, 1H, H-3 of C_3_H_1_N_2_), 8.16 (s, 1H, NH (D_2_O-exchangeable)), 9.24 (s, 1H, NH (D_2_O-exchangeable)), 12.62 (s, 1H, NH (D_2_O-exchangeable)); Anal. Calcd for C_25_H_21_N_7_O_5_S_3_ (595.67 g/mol): C, 50.41; H, 3.55; N, 16.46; found C, 50.32; H, 3.54; N, 16.47; HRMS (ESI) for C_25_H_22_N_7_O_5_S_3_, calcd 596.0839, found 596.0851 [M + H]^+^, and for C_25_H_21_N_7_NaO_5_S_3_, calcd 618.0658, found 618.0669 [M + Na]^+^, and for C_25_H_20_N_7_O_5_S_3_, calcd 594.0694, found 594.0688 [M – H]^−^.

##### 4.1.6.8 4-(4-(3-(4-Nitrophenyl)ureido)-5-phenyl-1*H*-pyrazolyl)benzenesulfonamide (**SH7h**)

White crystals (yield 74%); mp 239–241 °C; HPLC purity: 95.7%; ^1^H NMR (400 MHz, DMSO-*d*_6_): δ ppm 7.31 (d, 2H, H-2 and H-6 of C_6_H_5_, J = 7.5 Hz), 7.36 (d, 2H, H-3 and H-5 of 4-SO_2_NH_2_–C_6_H_4_, J = 8.6 Hz), 7.40 (s, 2H, SO_2_NH_2_ (D_2_O-exchangeable)), 7.43–7.56 (m, 3H, H-3, H-4 and H-5 of C_6_H_5_), 7.64 (d, 2H, H-3 and H-5 of 4-NO_2_–C_6_H_4_, J = 9.1 Hz), 7.76 (d, 2H, H-2 and H-6 of 4-SO_2_NH_2_–C_6_H_4_, J = 8.6 Hz), 8.08–8.26 (m, 4H, H-2 and H-6 of 4-NO_2_–C_6_H_4_, H-3 of C_3_H_1_N_2_ and NH (D_2_O-exchangeable)), 9.57 (s, 1H, NH (D_2_O-exchangeable)); Anal. Calcd for C_22_H_18_N_6_O_5_S (478.48 g/mol): C, 55.22; H, 3.79; N, 17.56; found C, 55.38; H, 3.79; N, 17.49; HRMS (ESI) for C_22_H_19_N_6_O_5_S, calcd 479.1132, found 479.1136 [M + H]^+^, and for C_22_H_18_N_6_NaO_5_S, calcd 501.0952, found 501.0957 [M + Na]^+^.

##### 4.1.6.9 4-(5-(4-Methoxyphenyl)-4-(3-phenylureido)-1*H*-pyrazolyl)benzenesulfonamide (**SH7i**)

White crystals (yield 81%); mp 226–227 °C; HPLC purity: 95.9%; ^1^H NMR (400 MHz, DMSO-*d*_6_): δ ppm 3.79 (s, 3H, OCH_3_), 6.93 (t, 1H, H-4 of C_6_H_5_, J = 7.3 Hz), 7.04 (d, 2H, H-3 and H-5 of 4-OCH_3_–C_6_H_4_, J = 8.7 Hz), 7.22 (d, 2H, H-2 and H-6 of 4-OCH_3_–C_6_H_4_, J = 8.8 Hz), 7.24 (t, 2H, H-3 and H-5 of C_6_H_5_, J = 7.9 Hz), 7.36 (d, 2H, H-3 and H-5 of 4-SO_2_NH_2_–C_6_H_4_, J = 8.7 Hz), 7.38 (s, 2H, SO_2_NH_2_ (D_2_O-exchangeable)), 7.40 (d, 2H, H-2 and H-6 of C_6_H_5_, J = 7.8 Hz), 7.76 (d, 2H, H-2 and H-6 of 4-SO_2_NH_2_–C_6_H_4_, J = 8.7 Hz), 7.84 (s, 1H, H-3 of C_3_H_1_N_2_), 8.13 (s, 1H, NH (D_2_O-exchangeable)), 8.83 (s, 1H, NH (D_2_O-exchangeable)); ^13^C NMR (101 MHz, DMSO-*d*_6_): δ ppm 55.73 (OCH_3_), 115.09, 118.31, 120.70, 122.14, 122.61, 124.39, 126.98, 129.25, 131.72, 131.78, 135.72, 140.21, 142.38, 142.53, 153.32, 160.09; Anal. Calcd for C_23_H_21_N_5_O_4_S (463.51 g/mol): C, 59.60; H, 4.57; N, 15.11; found C, 59.76; H, 4.55; N, 15.05; HRMS (ESI) for C_23_H_22_N_5_O_4_S, calcd 464.1387, found 464.1391 [M + H]^+^, and for C_23_H_21_N_5_NaO_4_S, calcd 486.1206, found 486.1212 [M + Na]^+^, for C_22_H_17_FN_5_O_3_S, calcd 462.1241, found 462.1239 [M – H]^−^, and for C_22_H_18_ClFN_5_O_3_S, calcd 498.1008, found 498.1004 [M + Cl]^−^.

##### 4.1.6.10 4-(4-(3-(4-Fluorophenyl)ureido)-5-(4-methoxyphenyl)-1*H*-pyrazolyl)benzenesulfonamide (**SH7j**)

White crystals (yield 85%); mp 233–234 °C; HPLC purity: 97.5%; ^1^H NMR (400 MHz, DMSO-*d*_6_): δ ppm 3.78 (s, 3H, OCH_3_), 7.04 (d, 2H, H-3 and H-5 of 4-OCH_3_–C_6_H_4_, J = 8.8 Hz), 7.08 (t, 2H, H-3 and H-5 of 4-F–C_6_H_4_, J = 8.9 Hz), 7.22 (d, 2H, H-2 and H-6 of 4-OCH_3_–C_6_H_4_, J = 8.8 Hz), 7.36 (d, 2H, H-3 and H-5 of 4-SO_2_NH_2_–C_6_H_4_, J = 8.7 Hz), 7.38 (s, 2H, SO_2_NH_2_ (D_2_O-exchangeable)), 7.41 (dd, 2H, H-2 and H-6 of 4-F–C_6_H_4_, J = 9.2, 5.0 Hz), 7.76 (d, 2H, H-2 and H-6 of 4-SO_2_NH_2_–C_6_H_4_, J = 8.7 Hz), 7.83 (s, 1H, H-3 of C_3_H_1_N_2_), 8.11 (s, 1H, NH (D_2_O-exchangeable)), 8.85 (s, 1H, NH (D_2_O-exchangeable)); Anal. Calcd for C_23_H_20_FN_5_O_4_S (481.50 g/mol): C, 57.37; H, 4.19; N, 14.55; found C, 57.32; H, 4.21; N, 14.60; HRMS (ESI) for C_23_H_21_FN_5_O_4_S, calcd 482.1293, found 482.1296 [M + H]^+^, and for C_23_H_20_FN_5_NaO_4_S, calcd 504.1112, found 504.1117 [M + Na]^+^.

##### 4.1.6.11 4-(4-(3-(4-Chlorophenyl)ureido)-5-(4-methoxyphenyl)-1*H*-pyrazolyl)benzenesulfonamide (**SH7k**)

White crystals (yield 79%); mp 240–241 °C; HPLC purity: 97.5%; ^1^H NMR (400 MHz, DMSO-*d*_6_): δ ppm 3.76 (s, 3H, OCH_3_), 7.01 (d, 2H, H-3 and H-5 of 4-OCH_3_–C_6_H_4_, J = 8.8 Hz), 7.18 (d, 2H, H-3 and H-5 of 4-Cl–C_6_H_4_, J = 8.7 Hz), 7.28 (d, 2H, H-2 and H-6 of 4-OCH_3_–C_6_H_4_, J = 8.9 Hz), 7.36 (d, 2H, H-3 and H-5 of 4-SO_2_NH_2_–C_6_H_4_, J = 8.7 Hz), 7.38 (s, 2H, SO_2_NH_2_ (D_2_O-exchangeable)), 7.40 (d, 2H, H-2 and H-6 of 4-Cl–C_6_H_4_, J = 8.9 Hz), 7.77 (d, 2H, H-2 and H-6 of 4-SO_2_NH_2_–C_6_H_4_, J = 8.7 Hz), 8.06 (s, 1H, H-3 of C_3_H_1_N_2_), 8.12 (s, 1H, NH (D_2_O-exchangeable)), 8.87 (s, 1H, NH (D_2_O-exchangeable)); Anal. Calcd for C_23_H_20_ClN_5_O_4_S (497.95 g/mol): C, 55.48; H, 4.05; N, 14.06; found C, 55.51; H, 4.04; N, 14.09; HRMS (ESI) for C_23_H_21_ClN_5_O_4_S, calcd 498.0997, found 498.1001 [M + H]^+^, and for C_23_H_20_ClN_5_NaO_4_S, calcd 520.0817, found 520.0822 [M + Na]^+^, for C_23_H_19_ClN_5_O_4_S, calcd 496.0852, found 496.0845 [M – H]^−^, and for C_23_H_20_Cl_2_N_5_O_4_S, calcd 532.0619, found 532.0613 [M + Cl]^−^.

##### 4.1.6.12 4-(5-(4-Methoxyphenyl)-4-(3-(4-methoxyphenyl)ureido)-1*H*-pyrazolyl)benzenesulfonamide (**SH7l**)

White crystals (yield 86%); mp 232–234 °C; HPLC purity: 98.6%; ^1^H NMR (400 MHz, DMSO-*d*_6_): δ ppm 3.69 (s, 3H, OCH_3_), 3.79 (s, 3H, OCH_3_), 6.83 (d, 2H, H-3′ and H-5′ of 4′-OCH_3_–C_6_H_4_, J = 9.0 Hz), 7.04 (d, 2H, H-3 and H-5 of 4-OCH_3_–C_6_H_4_, J = 8.8 Hz), 7.22 (d, 2H, H-2 and H-6 of 4-OCH_3_–C_6_H_4_, J = 8.8 Hz), 7.30 (d, 2H, H-2′ and H-6′ of 4′-OCH_3_–C_6_H_4_, J = 9.0 Hz), 7.36 (d, 2H, H-3 and H-5 of 4-SO_2_NH_2_–C_6_H_4_, J = 8.7 Hz), 7.38 (s, 2H, SO_2_NH_2_ (D_2_O-exchangeable)), 7.71–7.81 (m, 3H, H-2 and H-6 of 4-SO_2_NH_2_–C_6_H_4_ and H-3 of C_3_H_1_N_2_), 8.11 (s, 1H, NH (D_2_O-exchangeable)), 8.65 (s, 1H, NH (D_2_O-exchangeable)); Anal. Calcd for C_24_H_23_N_5_O_5_S (493.54 g/mol): C, 58.41; H, 4.70; N, 14.19; found C, 58.57; H, 4.68; N, 14.13; HRMS (ESI) for C_24_H_24_N_5_O_5_S, calcd 494.1493, found 494.1494 [M + H]^+^, and for C_24_H_23_N_5_NaO_5_S, calcd 516.1312, found 516.1317 [M + Na]^+^.

##### 4.1.6.13 Ethyl 4-(3-(5-(4-Methoxyphenyl)-1-(4-sulfamoylphenyl)-1*H*-pyrazol-4-yl)ureido)benzoate (**SH7m**)

White crystals (yield 82%); mp 219–221 °C; HPLC purity: 96.0%; ^1^H NMR (400 MHz, DMSO-*d*_6_): δ ppm 1.29 (t, 3H, O–CH_2_–CH_3_, J = 7.1 Hz), 3.79 (s, 3H, OCH_3_), 4.25 (q, 2H, O–CH_2_-CH_3_, J = 7.1 Hz), 7.04 (d, 2H, H-3 and H-5 of 4-OCH_3_–C_6_H_4_, J = 8.8 Hz), 7.23 (d, 2H, H-2 and H-6 of 4-OCH_3_–C_6_H_4_, J = 8.8 Hz), 7.36 (d, 2H, H-3 and H-5 of 4-SO_2_NH_2_–C_6_H_4_, J = 8.7 Hz), 7.39 (s, 2H, SO_2_NH_2_ (D_2_O-exchangeable)), 7.53 (d, 2H, H-2 and H-6 of 4-CO_2_C_2_H_5_–C_6_H_4_, J = 8.8 Hz), 7.76 (d, 2H, H-2 and H-6 of 4-SO_2_NH_2_–C_6_H_4_, J = 8.7 Hz), 7.85 (d, 2H, H-3 and H-5 of 4-CO_2_C_2_H_5_–C_6_H_4_, J = 8.8 Hz), 7.99 (s, 1H, H-3 of C_3_H_1_N_2_), 8.14 (s, 1H, NH (D_2_O-exchangeable)), 9.25 (s, 1H, NH (D_2_O-exchangeable)); Anal. Calcd for C_26_H_25_N_5_O_6_S (535.58 g/mol): C, 58.31; H, 4.71; N, 13.08; found C, 58.16; H, 4.71; N, 13.13; HRMS (ESI) for C_26_H_26_N_5_O_6_S, calcd 536.1598, found 536.1603 [M + H]^+^, and for C_26_H_25_N_5_NaO_6_S, calcd 558.1418, found 558.1424 [M + Na]^+^, for C_26_H_24_N_5_O_6_S, calcd 534.1453, found 534.1443 [M – H]^−^, and for C_26_H_25_ClN_5_O_6_S, calcd 570.1220, found 570.1210 [M + Cl]^−^.

##### 4.1.6.14 4-(3-(5-(4-Methoxyphenyl)-1-(4-sulfamoylphenyl)-1*H*-pyrazol-4-yl)ureido)benzenesulfonamide (**SH7n**)

White crystals (yield 78%); mp 288–290 °C; HPLC purity: 99.4%; ^1^H NMR (400 MHz, DMSO-*d*_6_): δ ppm 3.79 (s, 3H, OCH_3_), 7.04 (d, 2H, H-3 and H-5 of 4-OCH_3_–C_6_H_4_, J = 8.7 Hz), 7.17 (s, 2H, SO_2_NH_2_ (D_2_O-exchangeable)), 7.23 (d, 2H, H-2 and H-6 of 4-OCH_3_–C_6_H_4_, J = 8.7 Hz), 7.36 (d, 2H, H-3 and H-5 of 4-SO_2_NH_2_–C_6_H_4_, J = 8.7 Hz), 7.39 (s, 2H, SO_2_NH_2_ (D_2_O-exchangeable)), 7.56 (d, 2H, H-3′ and H-5′ of 4′-SO_2_NH_2_–C_6_H_4_, J = 8.8 Hz), 7.69 (d, 2H, H-2′ and H-6′ of 4′-SO_2_NH_2_–C_6_H_4_, J = 8.8 Hz), 7.76 (d, 2H, H-2 and H-6 of 4-SO_2_NH_2_–C_6_H_4_, J = 8.7 Hz), 7.99 (s, 1H, H-3 of C_3_H_1_N_2_), 8.13 (s, 1H, NH (D_2_O-exchangeable)), 9.22 (s, 1H, NH (D_2_O-exchangeable)); ^13^C NMR (101 MHz, DMSO-*d*_6_): δ ppm 55.74 (OCH_3_), 115.11, 117.62, 120.57, 122.21, 124.46, 127.00, 127.30, 131.73, 132.15, 135.71, 137.21, 138.22, 142.47, 143.28, 153.06, 160.14; Anal. Calcd for C_23_H_22_N_6_O_6_S_2_ (542.59 g/mol): C, 50.91; H, 4.09; N, 15.49; found C, 50.95; H, 4.09; N, 15.52; HRMS (ESI) for C_23_H_23_N_6_O_6_S_2_, calcd 543.1115, found 543.1120 [M + H]^+^, and for C_23_H_22_N_6_NaO_6_S_2_, calcd 565.0934, found 565.0940 [M + Na]^+^, for C_23_H_21_N_6_O_6_S_2_, calcd 541.0969, found 541.0966 [M – H]^−^, and for C_23_H_22_ClN_6_O_6_S_2_, calcd 577.0736, found 577.0733 [M + Cl]^−^.

##### 4.1.6.15 4-(3-(5-(4-Methoxyphenyl)-1-(4-sulfamoylphenyl)-1*H*-pyrazol-4-yl)ureido)-*N*-(thiazol-2-yl)benzenesulfonamide (**SH7o**)

White crystals (yield 77%); mp 271–273 °C; HPLC purity: 97.5%; ^1^H NMR (400 MHz, DMSO-*d*_6_): δ ppm 3.78 (s, 3H, OCH_3_), 6.78 (d, 1H, H-5 of C_3_H_2_NS, J = 4.6 Hz), 7.04 (d, 2H, H-3 and H-5 of 4-OCH_3_–C_6_H_4_, J = 8.8 Hz), 7.21 (d, 2H, H-2 and H-6 of 4-OCH_3_–C_6_H_4_, J = 8.8 Hz), 7.22 (d, 1H, H-4 of C_3_H_2_NS, J = 4.5 Hz), 7.36 (d, 2H, H-3 and H-5 of 4-SO_2_NH_2_–C_6_H_4_, J = 8.7 Hz), 7.38 (s, 2H, SO_2_NH_2_ (D_2_O-exchangeable)), 7.53 (d, 2H, H-2 and H-6 of 4-SO_2_NH(C_3_H_2_NS)–C_6_H_4_, J = 8.8 Hz), 7.67 (d, 2H, H-3 and H-5 of 4-SO_2_NH(C_3_H_2_NS)–C_6_H_4_, J = 8.8 Hz), 7.76 (d, 2H, H-2 and H-6 of 4-SO_2_NH_2_–C_6_H_4_, J = 8.7 Hz), 7.97 (s, 1H, H-3 of C_3_H_1_N_2_), 8.12 (s, 1H, NH (D_2_O-exchangeable)), 9.21 (s, 1H, NH (D_2_O-exchangeable)), 12.61 (br s, 1H, NH (D_2_O-exchangeable)); Anal. Calcd for C_26_H_23_N_7_O_6_S_3_ (625.69 g/mol): C, 49.91; H, 3.71; N, 15.67; found C, 49.92; H, 3.70; N, 15.65; HRMS (ESI) for C_26_H_24_N_7_O_6_S_3_, calcd 626.0945, found 626.0950 [M + H]^+^, and for C_26_H_23_N_7_NaO_6_S_3_, calcd 648.0764, found 648.0767 [M + Na]^+^, and for C_26_H_22_N_7_O_6_S_3_, calcd 624.0799, found 624.0794 [M – H]^−^.

##### 4.1.6.16 4-(4-(3-(4-Bromophenyl)ureido)-5-(4-methoxyphenyl)-1*H*-pyrazolyl)benzenesulfonamide (**SH7p**)

White crystals (yield 83%); mp 254–255 °C; ^1^H NMR (400 MHz, DMSO-*d*_6_): δ ppm 3.79 (s, 3H, OCH_3_), 7.04 (d, 2H, H-3 and H-5 of 4-OCH_3_–C_6_H_4_, J = 8.7 Hz), 7.22 (d, 2H, H-3 and H-5 of 4–Br-C_6_H_4_, J = 8.7 Hz), 7.31–7.44 (m, 8H, H-2 and H-6 of 4-OCH_3_–C_6_H_4_, H-3 and H-5 of 4-SO_2_NH_2_–C_6_H_4_, SO_2_NH_2_ (D_2_O-exchangeable), and H-2 and H-6 of 4-Br–C_6_H_4_), 7.78 (d, 2H, H-2 and H-6 of 4-SO_2_NH_2_–C_6_H_4_, J = 8.7 Hz), 8.11 (s, 1H, H-3 of C_3_H_1_N_2_), 8.17 (s, 1H, NH (D_2_O-exchangeable)), 8.81 (s, 1H, NH (D_2_O-exchangeable)); Anal. Calcd for C_23_H_20_BrN_5_O_4_S (542.41 g/mol): C, 50.93; H, 3.72; N, 12.91; found C, 50.86; H, 3.73; N, 12.93; HRMS (ESI) for C_23_H_21_BrN_5_O_4_S, calcd 542.0492, found 542.0497 [M + H]^+^, and for C_23_H_20_BrN_5_NaO_4_S, calcd 564.0312, found 564.0317 [M + Na]^+^, and for C_23_H_19_BrN_5_O_4_S, calcd 540.0347, found 542.0351 [M – H]^−^.

##### 4.1.6.17 4-(4-(3-(4-Chloro-2-fluorophenyl)ureido)-5-(4-methoxyphenyl)-1*H*-pyrazolyl)benzenesulfonamide (**SH7q**)

White crystals (yield 87%); mp 247–249 °C; ^1^H NMR (400 MHz, DMSO-*d*_6_): δ ppm 3.78 (s, 3H, OCH_3_), 7.03 (d, 2H, H-3 and H-5 of 4-OCH_3_–C_6_H_4_, J = 8.8 Hz), 7.18–7.24 (m, 3H, H-2 and H-6 of 4-OCH_3_–C_6_H_4_ and H-5 of 4-Cl–2-F–C_6_H_3_), 7.29 (t, 1H, H-6 of 4-Cl–2-F–C_6_H_3_, J = 9.0 Hz), 7.36 (d, 2H, H-3 and H-5 of 4-SO_2_NH_2_–C_6_H_4_, J = 8.7 Hz), 7.38 (s, 2H, SO_2_NH_2_ (D_2_O-exchangeable)), 7.76 (d, 2H, H-2 and H-6 of 4-SO_2_NH_2_–C_6_H_4_, J = 8.7 Hz), 7.79 (dd, 1H, H-3 of 4-Cl–2-F–C_6_H_3_, J = 6.7, 2.7 Hz), 7.92 (s, 1H, H-3 of C_3_H_1_N_2_), 8.09 (s, 1H, NH (D_2_O-exchangeable)), 9.01 (s, 1H, NH (D_2_O-exchangeable)); Anal. Calcd for C_23_H_19_ClFN_5_O_4_S (515.94 g/mol): C, 53.54; H, 3.71; N, 13.57; found C, 53.59; H, 3.72; N, 13.52; HRMS (ESI) for C_23_H_20_ClFN_5_O_4_S, calcd 516.0903, found 516.0909 [M + H]^+^, and for C_23_H_19_ClFN_5_NaO_4_S, calcd 538.0723, found 538.0729 [M + Na]^+^, for C_23_H_18_ClFN_5_O_4_S, calcd 514.0758, found 514.0766 [M – H]^−^, and for C_23_H_19_Cl_2_FN_5_O_4_S, calcd 550.0524, found 550.0521 [M + Cl]^−^.

##### 4.1.6.18 4-(5-(4-Methoxyphenyl)-4-(3-(3-(trifluoromethyl)phenyl)ureido)-1*H*-pyrazolyl)benzenesulfonamide (**SH7r**)

White crystals (yield 80%); mp 234–235 °C; ^1^H NMR (400 MHz, DMSO-*d*_6_): δ ppm 3.78 (s, 3H, OCH_3_), 7.04 (d, 2H, H-3 and H-5 of 4-OCH_3_–C_6_H_4_, J = 8.8 Hz), 7.23 (d, 2H, H-2 and H-6 of 4-OCH_3_–C_6_H_4_, J = 8.8 Hz), 7.27 (d, 1H, H-6 of 3-OCF_3_–C_6_H_4_, J = 6.3 Hz),7.37 (d, 2H, H-3 and H-5 of 4-SO_2_NH_2_–C_6_H_4_, J = 8.8 Hz), 7.38 (s, 2H, SO_2_NH_2_ (D_2_O-exchangeable)), 7.44–7.51 (m, 2H, H-4 and H-5 of 3-OCF_3_–C_6_H_4_), 7.76 (d, 2H, H-2 and H-6 of 4-SO_2_NH_2_–C_6_H_4_, J = 8.7 Hz), 7.96 (s, 1H, H-2 of 3-OCF_3_–C_6_H_4_), 7.99 (s, 1H, H-3 of C_3_H_1_N_2_), 8.13 (s, 1H, NH (D_2_O-exchangeable)), 9.19 (s, 1H, NH (D_2_O-exchangeable)); Anal. Calcd for C_24_H_20_F_3_N_5_O_4_S (531.51 g/mol): C, 54.23; H, 3.79; N, 13.18; found C, 54.22; H, 3.80; N, 13.13; HRMS (ESI) for C_24_H_21_F_3_N_5_O_4_S, calcd 532.1261, found 532.1268 [M + H]^+^, and for C_24_H_20_F_3_N_5_NaO_4_S, calcd 554.1080, found 554.1088 [M + Na]^+^, and for C_24_H_19_F_3_N_5_O_4_S, calcd 530.1115, found 530.1115 [M – H]^−^.

##### 4.1.6.19 4-(4-(3-(4-Chloro-3-(trifluoromethyl)phenyl)ureido)-5-(4-methoxyphenyl)-1*H*-pyrazolyl)benzenesulfonamide (**SH7s**)

White crystals (yield 75%); mp 245–247 °C; HPLC purity: 98.7%; ^1^H NMR (400 MHz, DMSO-*d*_6_): δ ppm 3.78 (s, 3H, OCH_3_), 7.03 (d, 2H, H-3 and H-5 of 4-OCH_3_–C_6_H_4_, J = 8.8 Hz), 7.22 (d, 2H, H-2 and H-6 of 4-OCH_3_–C_6_H_4_, J = 8.7 Hz), 7.37 (d, 2H, H-3 and H-5 of 4-SO_2_NH_2_–C_6_H_4_, J = 8.7 Hz), 7.39 (s, 2H, SO_2_NH_2_ (D_2_O-exchangeable)), 7.54–7.59 (m, 2H, H-5 and H-6 of 4-Cl–3-CF_3_–C_6_H_3_), 7.76 (d, 2H, H-2 and H-6 of 4-SO_2_NH_2_–C_6_H_4_, J = 8.7 Hz), 8.00 (s, 1H, H-2 of 4-Cl–3-CF_3_–C_6_H_3_), 8.05 (s, 1H, H-3 of C_3_H_1_N_2_), 8.10 (s, 1H, NH (D_2_O-exchangeable)), 9.28 (s, 1H, NH (D_2_O-exchangeable)); ^13^C NMR (101 MHz, DMSO-*d*_6_): δ ppm 55.72 (OCH_3_), 115.07, 116.85, 116.91, 120.55, 121.91, 122.58, 123.13, 124.49, 124.61, 127.00, 127.30, 131.67, 132.46, 132.75, 136.11, 139.81, 142.47, 142.51, 153.29, 160.13; Anal. Calcd for C_24_H_19_ClF_3_N_5_O_4_S (565.95 g/mol): C, 50.93; H, 3.38; N, 12.37; found C, 50.90; H, 3.37; N, 12.40; HRMS (ESI) for C_24_H_20_ClF_3_N_5_O_4_S, calcd 566.0871, found 566.0878 [M + H]^+^, and for C_24_H_19_ClF_3_N_5_NaO_4_S, calcd 588.0691, found 588.070 [M + Na]^+^.

##### 4.1.6.20 4-(5-(4-Methoxyphenyl)-4-(3-(4-(trifluoromethoxy)phenyl)ureido)-1*H*-pyrazolyl)benzenesulfonamide (**SH7t**)

White crystals (yield 78%); mp 241–242 °C; HPLC purity: 99.1%; ^1^H NMR (400 MHz, DMSO-*d*_6_): δ ppm 3.78 (s, 3H, OCH_3_), 7.03 (d, 2H, H-3 and H-5 of 4-OCH_3_–C_6_H_4_, J = 8.8 Hz), 7.22 (d, 2H, H-2 and H-6 of 4-OCH_3_–C_6_H_4_, J = 8.8 Hz), 7.24 (d, 2H, H-3 and H-5 of 4-OCF_3_–C_6_H_4_, J = 9.0 Hz), 7.36 (d, 2H, H-3 and H-5 of 4-SO_2_NH_2_–C_6_H_4_, J = 8.7 Hz), 7.39 (s, 2H, SO_2_NH_2_ (D_2_O-exchangeable)), 7.50 (d, 2H, H-2 and H-6 of 4-OCF_3_–C_6_H_4_, J = 9.1 Hz), 7.76 (d, 2H, H-2 and H-6 of 4-SO_2_NH_2_–C_6_H_4_, J = 8.7 Hz), 7.89 (s, 1H, H-3 of C_3_H_1_N_2_), 8.11 (s, 1H, NH (D_2_O-exchangeable)), 9.03 (s, 1H, NH (D_2_O-exchangeable)); ^13^C NMR (101 MHz, DMSO-*d*_6_): δ ppm 55.72 (OCH_3_), 115.08, 119.36, 119.52, 120.61, 121.90, 122.18, 122.30, 124.45, 126.99, 131.70, 132.24, 135.87, 139.47, 142.43, 142.50, 142.94, 142.96, 153.33, 160.11; Anal. Calcd for C_24_H_20_F_3_N_5_O_5_S (547.51 g/mol): C, 52.65; H, 3.68; N, 12.79; found C, 52.73; H, 3.67; N, 12.77; HRMS (ESI) for C_24_H_21_F_3_N_5_O_5_S, calcd 548.1210, found 548.1218 [M + H]^+^, and for C_24_H_20_F_3_N_5_NaO_5_S, calcd 570.1029, found 570.1038 [M + Na]^+^.

### 4.2 Biological Evaluations

#### 4.2.1 CA Inhibitory Assay

An Applied photophysics stopped-flow instrument has been used for assaying the CA-catalyzed CO_2_ hydration activity.^18^ Phenol red (at a concentration of 0.2 mM) has been used as an indicator, working at the absorbance maximum of 557 nm, with 20 mM Hepes (pH 7.5) as a buffer and 20 mM Na_2_SO_4_ (for maintaining constant the ionic strength), following the initial rates of the CA-catalyzed CO_2_ hydration reaction for a period of 10–100 s. The CO_2_ concentrations ranged from 1.7 to 17 mM for the determination of the kinetic parameters and inhibition constants. For each inhibitor, at least six traces of the initial 5–10% of the reaction have been used for determining the initial velocity. The uncatalyzed rates were determined in the same manner and subtracted from the total observed rates. Stock solutions of inhibitor (0.1 mM) were prepared in distilled–deionized water and dilutions up to 0.01 nM were done thereafter with the assay buffer. Inhibitor and enzyme solutions were preincubated together for 15 min at room temperature before assay to allow for the formation of the E–I complex. The inhibition constants were obtained by nonlinear least-squares methods using PRISM 3 and the Cheng–Prusoff equation, as reported earlier,^31^ and represent the mean from at least three different determinations. Enzyme concentrations were in the range of 5–18 nM. All CA isoforms were recombinant ones, obtained in-house as reported earlier.^31^

#### 4.2.2 X-ray Crystallography

The CA IX protein mimic was expressed, purified and crystallized as previously reported.^32^ Briefly, the protein was expressed in *Escherichia coli* BL21-DE3 cells and induced with 0.5 mM IPTG at mid log phase. The cells were grown for another 4 h, centrifuged and the cell pellets were stored at −20 °C. The cells were later disrupted using a cell crusher (Avestin), the resulting slurry was centrifuged to remove cell debris, and the supernatant was loaded onto a His-Trap 1 mL FF column, washed with 20 column volumes of buffer, and the protein was eluted with 5 column volumes of buffer plus 250 mM imidazole. The peak fractions were pooled, and a gel filtration column (S200 16/60) was used to further purify the protein. The protein was then concentrated to about 11 mg/mL for storage.

The protein was crystallized in sitting drop plates using 150 nL of protein at 5.5 mg/mL with 120 nL reservoir solution with 30 nL microcrystalline seeds over 50 μL reservoirs at both 8° and 20 °C. Plate-like crystals grew at both temperatures in optimized conditions of 2.6–2.8 M ammonium sulfate, 100 mM tris buffer at pH 8.0–9.0. Ligand(s) were soaked into preformed crystals by adding compound directly and resealing the plate. Crystals were harvested using loops with the addition of glycerol (20% final concentration) and cryo-cooled in liquid nitrogen for transport and data collection at the Australian Synchrotron.

360 deg of data were collected from each crystal at the MX1 beamline, indexed using XDS^33^ and scaled using Aimless.^34^ Molecular replacement was used (Phaser^35^). The model was refined manually with Coot^36^ and further refined using REFMAC.^37^ The structure coordinates and structure factors have been deposited in the PDB with accession code 8TTR.

#### 4.2.3 Molecular Docking

Molecular docking was performed using Schrodinger 19-1 package. The protein crystal structure (PDB: 8TTR) was prepared using the Protein Preparation Wizard^38,39^ by adding hydrogen atoms and assigning bond orders along with creating zero -order bonds to metals. Water molecules 5 Å away from the ligands or making less than two hydrogen bonds with nonwaters were deleted. Filling in missing side chains was performed using Prime.^40−42^ Ionization states of the ligands were generated using Epik^43−45^ at pH 7.0 ± 2.0. Hydrogen bonds were optimized using PROPKA at pH 7.0. The prepared protein structure was subjected to restrained minimization.

The ligand **SH7s** was prepared utilizing the LigPrep^46^ panel with generating possible states, including metal binding states, at pH 7.0 ± 2.0. The prepared ligand structure with deprotonated sulfonamide nitrogen was used for docking. Receptor Grid Generation panel was used to generate the grids by utilizing the centroid of the ligand as the center of the grid. Docking was performed using Glide^47−50^ with flexible ligand sampling and standard precision. Five poses were subjected to post-docking minimization and the top-scored pose was reported.

#### 4.2.4 In Vitro Anticancer Activity toward 60 Cancer Cell Lines (NCI-60)

The anticancer assays were conducted following the protocol established by the Drug Evaluation Branch of the NCI in Bethesda, MD utilizing the sulforhodamine B (SRB) protein assay, as reported previously.^51,52^

#### 4.2.5 Anticancer Activity of **SH7s** Alone and as Adjuvant Cotreatment with Taxol and 5-FU

The impact of a single treatment of the most promising derivative **SH7s** or its proposed drug efficacy-boosting, adjuvant effect in a cotreatment regime with the approved anticancer drugs Taxol and 5-FU on the *in vitro* viability and proliferation of human cancer cells was investigated by using the human colorectal cancer cell line HCT-116 in cell viability assays. The cells were cultured in their specific growth medium composed of McCoy’s 5A basal medium supplemented with 10% (v/v) heat-inactivated fetal calf serum (FCS), 2 mM l-glutamine and 0.1% (v/v) penicillin/streptomycin (Pen/Strep). For that purpose, cell culture and assay reagents and consumables were used and purchased as follows: McCoy’s 5A basal medium, FCS, l-glutamine (200 mM), phosphate buffered saline (PBS), 0.05% trypsin–EDTA and Pen/Strep (100×) were purchased from Capricorn Scientific GmbH (Ebsdorfergrund, Germany). Trypan blue in the context of counter-staining for viable cell counting was used from Invitrogen (Waltham, MA, USA). Resazurin and digitonin were purchased from Merck Sigma-Aldrich (Taufkirchen, Germany), DMSO from Duchefa Biochemie (Haarlem, The Netherlands). Cell culture and other lab plastics have been purchased from TPP (Trasadingen, Switzerland), Greiner Bio-One (Frickenhausen, Germany) and Sarstedt (Nümbrecht, Germany). For the hypoxic cell cultivation, AgelessEye oxygen indicator was purchased from Mitsubishi Gas Chemical Company (Tokyo, Japan), Image-iT Green Hypoxia Reagent from Fisher Scientific (Schwerte, Germany), O2frepak 100CC oxygen absorber (O2frepak, Guangdong, China), and MINI-Vertical plastic spacers 0.75 mm from Carl Roth (Karlsruhe, Germany).

Cell cultivation was routinely done in an Eppendorf CellXpert C170i CO_2_ incubator using T-75 flasks to reach subconfluency (∼70–80%) prior to subculturing or assay usage, when the adherent cells were rinsed with PBS and detached by using trypsin–EDTA (0.05% in PBS) prior to cell passaging and seeding. The cell viability assays were conducted in parallel under normoxic standard growth conditions (humidified atmosphere with 5% CO_2_ at 37 °C) and hypoxic conditions. For that purpose, the HCT-116 cells were seeded in 96-well plates with a density of 7000 cells/80 μL/well, ensuring that after 48 h of incubation the untreated controls reached a reproducible cell confluency of 80–90% in both normoxic and hypoxic conditions. After seeding, the adherent cells were initially allowed to attach to the well plates’ bottom. Afterward, the cells were treated (standard growth medium supplemented with the test items) for 48 h in single treatments with the most promising derivative of this study, **SH7s**, an already known CA inhibitor **SLC-0111**(24) (both with concentrations ranging from 100 μM to 45.7 nM in 3-fold dilutions), as well as the approved anticancer drugs paclitaxel (Taxol; concentration range 250 nM to 3.2 pM in 5-fold dilutions) and 5-fluorouracil (5-FU; concentration range 250 μM to 3.2 nM in 5-fold dilutions), and in cotreatments of both anticancer drugs (dose–response curves covering the same, aforementioned concentration ranges) with fixed concentrations of **SH7s** (10 μM; i.e. approximately the IC_20_ of the single treatment) and **SLC-0111** (100 μM; i.e. approximately the IC_20_ of the single treatment). Cells treated in parallel with blank standard culture medium were used as negative control (for data normalization set to 100% cell viability), and those treated with 100 μM of the cytotoxic saponin digitonin as positive control (for data normalization set to 0% cell viability). For usage under hypoxic conditions, all media used were degassed in advance by sonication for 15 min. Further, hypoxic assay conditions were established by adapting the hypoxic cell culture methodology suitable for standard incubators recently described by Matthiesen et al.^53^ In brief, plates to be treated under hypoxic culture conditions were equipped with sterile MINI-vertical plastic spacers between the lid of the plate and the wells. The plates were then placed inside vacuum bags and O2frepak 100CC oxygen absorber sachets were added in equal spacing surrounding the plates, while four sachets were used per plate with no more than four plates per bag. To monitor the oxygen content, i.e. hypoxia (<0.5%), in the well plates, two different types of oxygen indicators were used. The reversible resazurin/resorufin-based AgelessEye oxygen indicator (1 indicator per two plates), indicating deep hypoxia by a blue-to-pink color shift, and Image-iT Green Hypoxia Reagent, applied in one well per plate in accordance to the manufacturer’s guidelines, whereby green fluorescence after incubation additionally confirmed the achievement of hypoxic conditions. Finally, the vacuum bags were evacuated from ambient air and sealed under mild vacuum using a vacuum sealing machine. Subsequently, the sealed hypoxic cell assay plates were placed in the standard cell culture incubator and incubated, like the corresponding plates under normoxic conditions, for 48 h. After the incubation period, the vacuum bags were cut open and the assay plates were removed to be further processed similarly to those assay plates treated under normoxic conditions. That means, the incubation media were discarded, and the cells were rinsed once with PBS. The cells’ remaining viability and proliferation was measured by conducting a fluorometric resazurin-based cell viability assay using a well-established protocol.^54^ Resazurin solution (50 μM) in basal medium was prepared freshly prior use and added to the cells with 100 μL/well. Subsequently, the cells were incubated under normoxic standard growth conditions for further 2 h. Finally, the resazurin-resofurin conversion by remaining viable cells was fluorometrically measured (λ_exc._ 540 nm/λ_em._ 590 nm) by using a SpectraMax iD5 multiwell plate reader (Molecular Devices, San Jose, USA). Data were determined in three biological replicates, each with technical triplicates. IC_50_ curves and values were calculated by using the nonlinear regression package of GraphPad Prism v10.1 software (San Diego, CA, USA).

#### 4.2.6 Kinase Profiling

**SH7s** were tested against 258 kinases according to Reaction Biology protocol as reported.^55^ Compound was tested in single dose duplicate mode at a concentration of 20 μM. Control compound staurosporine was tested in 10-dose IC_50_ mode with 4-fold serial dilution starting at 20 or 100 μM. Alternate control compounds were tested in 10-dose IC_50_ mode with 3 or 4-fold serial dilution starting at 10, 20, 50, or 100 μM. Reactions were carried out at 10 μM ATP. Curve fits were performed where the enzyme activities at the highest concentration of compounds were less than 65%.

## Data Availability

Data will be made available on request; X-ray coordinates and structure factors are available from the PDB with accession number 8TTR.

## Supporting Information Available

Information of the final compounds **SH7a–t** spectral data (^1^H NMR, ^13^C NMR, HRMS and HPLC purity); dose–response curves of the antiproliferative/cytotoxic effects of the derivative **SH7s** and the CA reference inhibitor **SLC-0111** on human HCT-116 colorectal cancer cells; and kinase profiling assay results of **SH7s** and NCI charts for cellular assay for **SH7t** and **SH7s** (PDF)
Molecular formula strings of compounds **SLC-0111**, **1**-**VIII**, **1a**,**b**, **2a**,**b**, **3a**,**b**, **4a**,**b**, **5a**,**b**, **6a**,**b**, **SH7a–t** (CSV)
Co-crystallized ligand (**SH7f**) in CA IX mimic (PDB: 8TTR) (PDB)
Docking pose of compound (**SH7s**) in CA IX mimic (PDB: 8TTR) (PDB)
PDB validation report for CA IX mimic (PDB: 8TTR) (XLSX)

## Supplementary Material

jm4c01894_si_002.csv

jm4c01894_si_003.pdb

jm4c01894_si_004.pdb

## Abbreviations

AAZ: acetazolamide
ACN: acetonitrile
CA: carbonic anhydrase
CAI: CA inhibitor
SI: selectivity index
GI %: % growth inhibition
ZBGs: zinc binding groups
5-FU: 5-fluorouracil
