# Supplementary material for: 4-(Pyrazolyl)benzenesulfonamide Ureas as Carbonic Anhydrases Inhibitors and Hypoxia-Mediated Chemo-Sensitizing Agents in Colorectal Cancer Cells
Source: J Med Chem. 2024 Nov 17;67(22):20438–54. doi: 10.1021/acs.jmedchem.4c01894 (PMC11613454; doi:10.1021/acs.jmedchem.4c01894)
Supplement: Supplementary file 1 — jm4c01894_si_001.pdf [file jm4c01894_si_001.pdf]

Supporting information

4-(Pyrazolyl)benzenesulfonamide ureas as carbonic anhydrases inhibitors and hypoxia-mediated chemo-sensitizing agents in colorectal cancer cells

Wagdy M. Eldehna,[1,2*] Mohamed Fares,[3,4] Alessandro Bonardi,[5] Moscos Avgenikos,[6] Fady Baselious,[7] Matthias Schmidt,[7] Tarfah Al-Warhi,[8] Hatem A. Abdel-Aziz,[9] Robert Rennert,[6] Thomas S. Peat,[10] Claudiu T. Supuran, [5] Ludger A. Wessjohann,[6,*] and Hany S. Ibrahim,[3,6,7,*]

[1] *Department of Pharmaceutical Chemistry, Faculty of Pharmacy, Kafrelsheikh University, Kafrelsheikh 33516, Egypt.*

[2] *Department of Pharmaceutical Chemistry, Faculty of Pharmacy, Pharos University in Alexandria; Canal El Mahmoudia St., Alexandria 21648, Egypt*

[3] *Department of Pharmaceutical Chemistry, Faculty of Pharmacy, Egyptian Russian University, Badr City, Cairo 11829, Egypt*

[4] *Sydney Pharmacy School, the University of Sydney, NSW 2006, Australia*

[5] *Università degli Studi di Firenze, Department NEUROFARBA – Pharmaceutical and Nutraceutical section, University of Firenze, via Ugo Schiff 6, I-50019 Sesto Fiorentino, Firenze, Italy.*

[6] *Leibniz Institute of Plant Biochemistry, Department of Bioorganic Chemistry, D-06120 Halle (Saale), Germany*

[7] *Department of Medicinal Chemistry, Institute of Pharmacy, Martin-Luther-University of Halle-Wittenberg, D-06120 Halle (Saale), Germany*

[8] *Department of Chemistry, College of Science, Princess Nourah bint Abdulrahman University, Riyadh, Saudi Arabia*

[9] *Applied Organic Chemistry Department, National Research Center, Dokki 12622, Cairo, Egypt*

[10] *School of Biotechnology and Biomolecular Sciences, University of New South Wales, Sydney, NSW 2052, Australia.*

Corresponding Authors

wagdy2000@gmail.com (W.M. Eldehna), Ludger.Wessjohann@ipb-halle.de (L. Wessjohann), and hany.ibrahim@pharmazie.uni-halle.de (H.S. Ibrahim).

	Topic	Page
**1**	**^{1}H and ^{13}C NMR charts of final compounds**	**S6**
	**Figure S1.** ^{1}H NMR spectrum of compound **7a**	**S6**
	**Figure S2.** ^{1}H NMR spectrum of compound **7c**	**S7**
	**Figure S3.** ^{1}H NMR spectrum of compound **7f**	**S8**
	**Figure S4.** ^{1}H NMR spectrum of compound **7g** (D_2O)	**S9**
	**Figure S5.** ^{1}H NMR spectrum of compound **7h**	**S10**
	**Figure S6.** ^{1}H NMR spectrum of compound **7h** (D_2O)	**S11**
	**Figure S7.** ^{1}H NMR spectrum of compound **7i**	**S12**
	**Figure S8.** ^{1}H NMR spectrum of compound **7i** (D_2O)	**S13**
	**Figure S9.** ^{13}C NMR spectrum of compound **7i**	**S14**
	**Figure S10.** ^{1}H NMR spectrum of compound **7j**	**S15**
	**Figure S11.** ^{1}H NMR spectrum of compound **7j** (D_2O)	**S16**
	**Figure S12.** ^{1}H NMR spectrum of compound **7k** (D_2O)	**S17**
	**Figure S13.** ^{1}H NMR spectrum of compound **7l**	**S18**
	**Figure S14.** ^{1}H NMR spectrum of compound **7l** (D_2O)	**S19**
	**Figure S15.** ^{1}H NMR spectrum of compound **7m**	**S20**
	**Figure S16.** ^{1}H NMR spectrum of compound **7m** (D_2O)	**S21**
	**Figure S17.** ^{1}H NMR spectrum of compound **7n**	**S22**
	**Figure S18.** ^{1}H NMR spectrum of compound **7n** (D_2O)	**S23**
	**Figure S19.** ^{13}C NMR spectrum of compound **7n**	**S24**
	**Figure S20.** ^{1}H NMR spectrum of compound **7o**	**S25**
	**Figure S21.** ^{1}H NMR spectrum of compound **7o** (D_2O)	**S26**
	**Figure S22.** ^{1}H NMR spectrum of compound **7p** (D_2O)	**S27**
	**Figure S23.** ^{1}H NMR spectrum of compound **7q**	**S28**
	**Figure S24.** ^{1}H NMR spectrum of compound **7q** (D_2O)	**S29**
	**Figure S24.** ^{1}H NMR spectrum of compound **7r**	**S30**
	**Figure S26.** ^{1}H NMR spectrum of compound **7r** (D_2O)	**S31**
	**Figure S27.** ^{1}H NMR spectrum of compound **7s**	**S32**
	**Figure S28.** ^{1}H NMR spectrum of compound **7s** (D_2O)	**S33**

	**Figure S29.** ^{13}C NMR spectrum of compound **7s**	**S34**
	**Figure S30.** ^{1}H NMR spectrum of compound **7t**	**S35**
	**Figure S31.** ^{1}H NMR spectrum of compound **7t** (D_2O)	**S36**
	**Figure S32.** ^{13}C NMR spectrum of compound **7t**	**S37**
**2**	**HRMS spectra of final compounds**	**S38**
	**Figure S33.** HRMS spectrum of compound **7a.**	**S38**
	**Figure S34.** HRMS spectrum of compound **7a**	**S39**
	**Figure S35.** HRMS spectrum of compound **7b.**	**S40**
	**Figure S36.** HRMS spectrum of compound **7c.**	**S41**
	**Figure S37.** HRMS spectrum of compound **7c**	**S42**
	**Figure S38.** HRMS spectrum of compound **7c**	**S43**
	**Figure S39.** HRMS spectrum of compound **7d**	**S44**
	**Figure S40.** HRMS spectrum of compound **7d**	**S45**
	**Figure S41.** HRMS spectrum of compound **7d**	**S46**
	**Figure S42.** HRMS spectrum of compound **7e**	**S47**
	**Figure S43.** HRMS spectrum of compound **7e**	**S48**
	**Figure S44.** HRMS spectrum of compound **7e**	**S49**
	**Figure S45.** HRMS spectrum of compound **7f**	**S50**
	**Figure S46.** HRMS spectrum of compound **7f**	**S51**
	**Figure S47.** HRMS spectrum of compound **7g**	**S52**
	**Figure S48.** HRMS spectrum of compound **7g**	**S53**
	**Figure S49.** HRMS spectrum of compound **7h**	**S54**
	**Figure S50.** HRMS spectrum of compound **7i**	**S55**
	**Figure S51.** HRMS spectrum of compound **7i**	**S56**
	**Figure S52.** HRMS spectrum of compound **7i**	**S57**
	**Figure S53.** HRMS spectrum of compound **7k**	**S58**
	**Figure S54.** HRMS spectrum of compound **7k**	**S59**
	**Figure S55.** HRMS spectrum of compound **7l**	**S60**
	**Figure S56.** HRMS spectrum of compound **7l**	**S61**
	**Figure S57.** HRMS spectrum of compound **7m**	**S62**

	**Figure S58.** HRMS spectrum of compound **7m**	**S63**
	**Figure S59.** HRMS spectrum of compound **7n**	**S64**
	**Figure S60.** HRMS spectrum of compound **7n**	**S65**
	**Figure S61.** HRMS spectrum of compound **7n**	**S66**
	**Figure S62.** HRMS spectrum of compound **7o**	**S67**
	**Figure S63.** HRMS spectrum of compound **7o**	**S68**
	**Figure S64.** HRMS spectrum of compound **7p**	**S69**
	**Figure S65.** HRMS spectrum of compound **7p**	**S70**
	**Figure S66.** HRMS spectrum of compound **7q**	**S71**
	**Figure S67.** HRMS spectrum of compound **7q**	**S72**
	**Figure S68.** HRMS spectrum of compound **7q**	**S73**
	**Figure S69.** HRMS spectrum of compound **7r**	**S74**
	**Figure S70.** HRMS spectrum of compound **7r**	**S75**
	**Figure S71.** HRMS spectrum of compound **7r**	**S76**
	**Figure S72.** HRMS spectrum of compound **7s**	**S77**
	**Figure S73.** HRMS spectrum of compound **7s**	**S78**
	**Figure S74.** HRMS spectrum of compound **7t**	**S79**
**3**	**HPLC Purity charts**	**S80**
	**Figure S76.** HPLC chromatogram of compound **7a**	**S80**
	**Figure S77.** HPLC chromatogram of compound **7b**	**S81**
	**Figure S78.** HPLC chromatogram of compound **7c**	**S82**
	**Figure S79.** HPLC chromatogram of compound **7d**	**S83**
	**Figure S80.** HPLC chromatogram of compound **7e**	**S84**
	**Figure S81.** HPLC chromatogram of compound **7f**	**S85**
	**Figure S82.** HPLC chromatogram of compound **7g**	**S86**
	**Figure S83.** HPLC chromatogram of compound **7h**	**S87**
	**Figure S84.** HPLC chromatogram of compound **7i**	**S88**
	**Figure S85.** HPLC chromatogram of compound **7j**	**S89**
	**Figure S86.** HPLC chromatogram of compound **7k**	**S90**
	**Figure S87.** HPLC chromatogram of compound **7l**	**S91**

	**Figure S88.** HPLC chromatogram of compound **7n**	**S92**
	**Figure S89.** HPLC chromatogram of compound **7o**	**S93**
	**Figure S90.** HPLC chromatogram of compound **7s**	**S94**
	**Figure S91.** HPLC chromatogram of compound **7t**	**S95**
**4**	**Figure S92.** Dose-response curves of the anti-proliferative/cytotoxic effects of the derivative SH7s and the CA reference inhibitor SLC-0111 on human HCT-116 colorectal cancer cells	**S96**
**5**	**Table S1**. Kinase Assay profiling results for compound **SH7s**	**S97**
**٦**	**NCI charts for cellular assay**	**S103**
	**Figure S93.** Single dose assay of compound **7t** a panel of 60 cancer cell lines**.**	**S103**
	**Figure S94.** Single dose assay of compound **7s** a panel of 60 cancer cell lines	**S104**
	**Figure S95.** Five dose assay of compound **7s** a panel of 60 cancer cell lines	**S105**
	**Figure S96.** Five dose assay of compound **7s** a panel of 60 cancer cell lines	**S106**

^{1}H and ^{13}C NMR charts of the final compounds

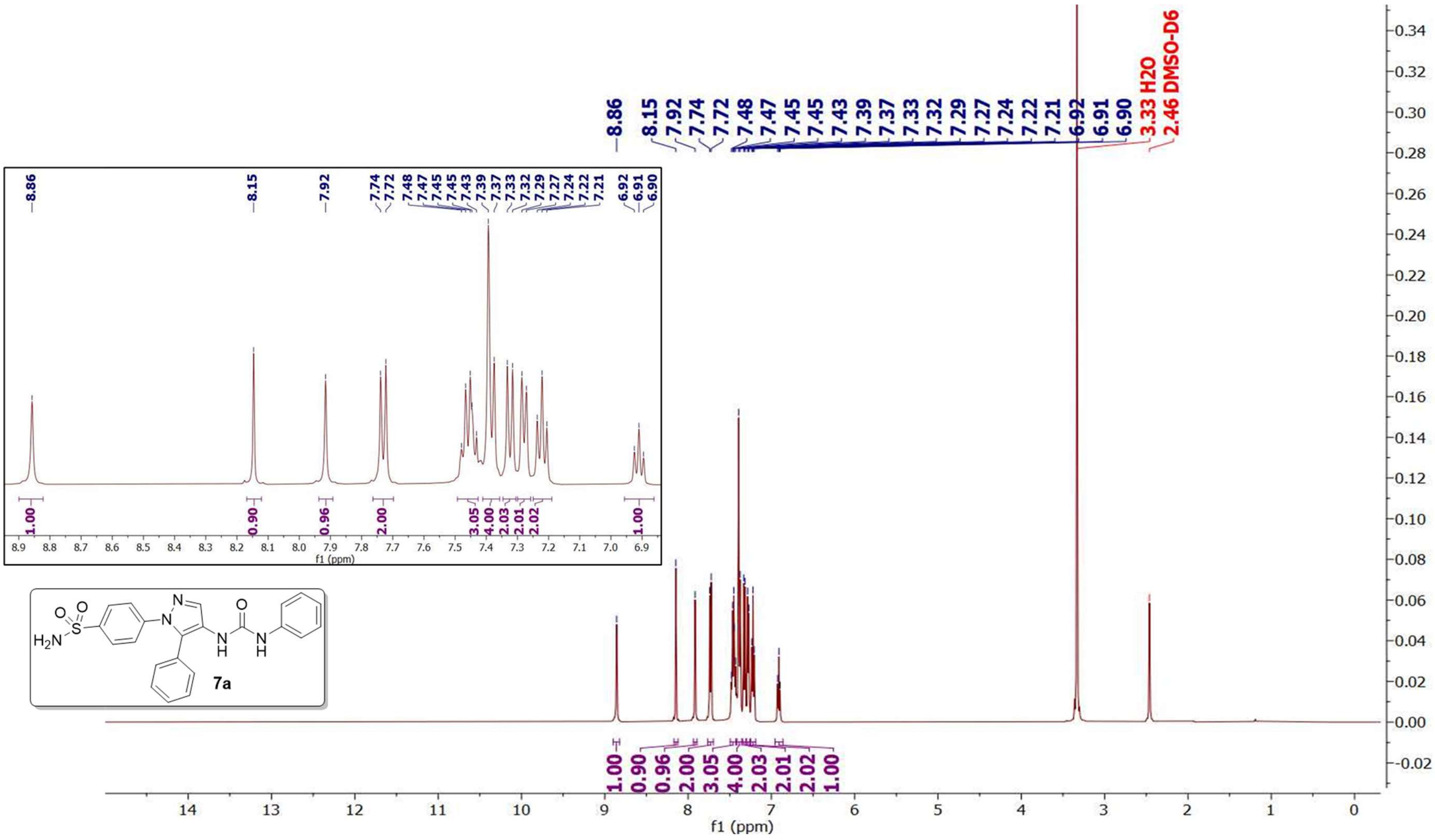

Figure S1. ^{1}H NMR spectrum of compound **7a.**

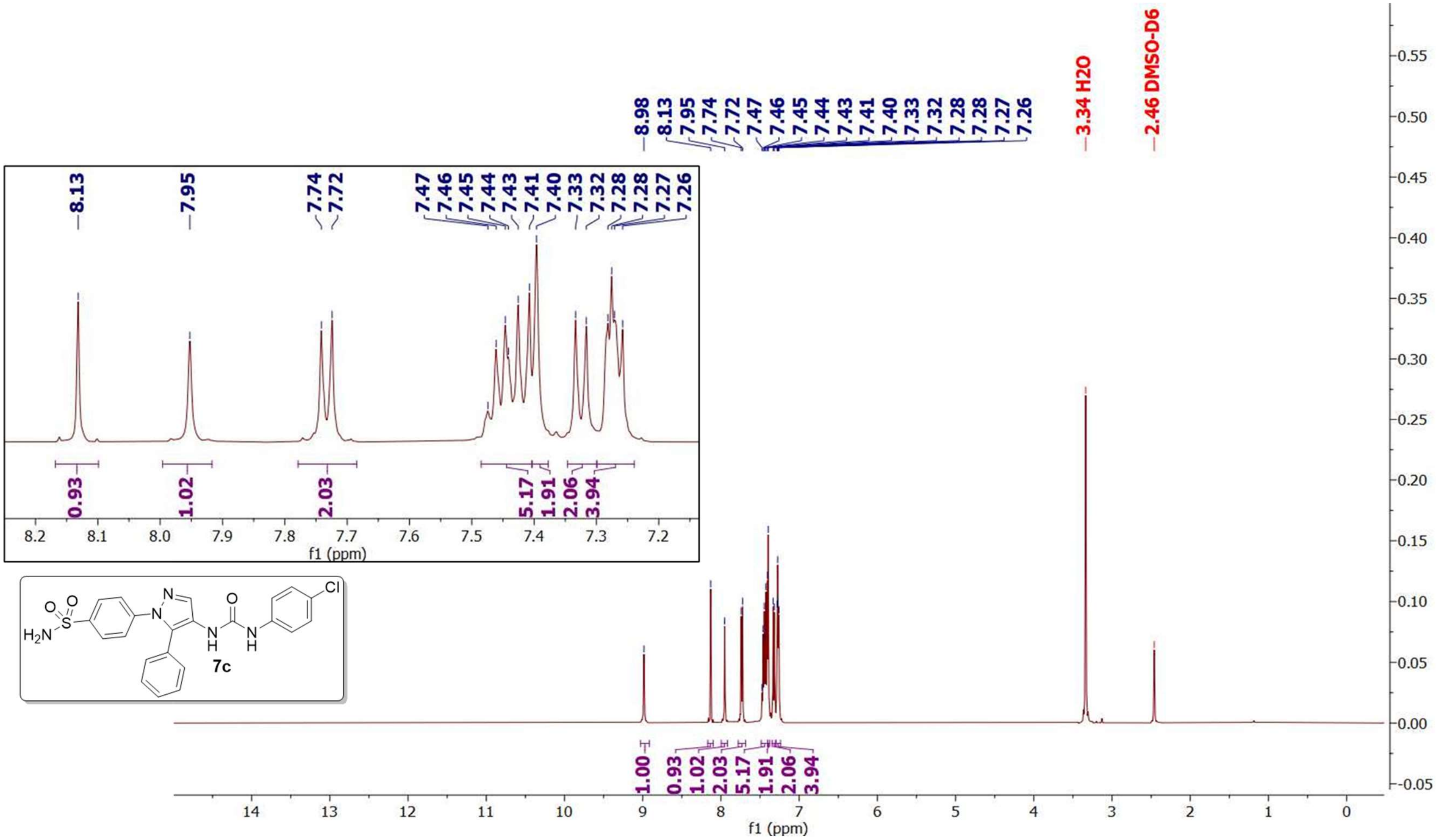

Figure S2. ^{1}H NMR spectrum of compound **7c.**

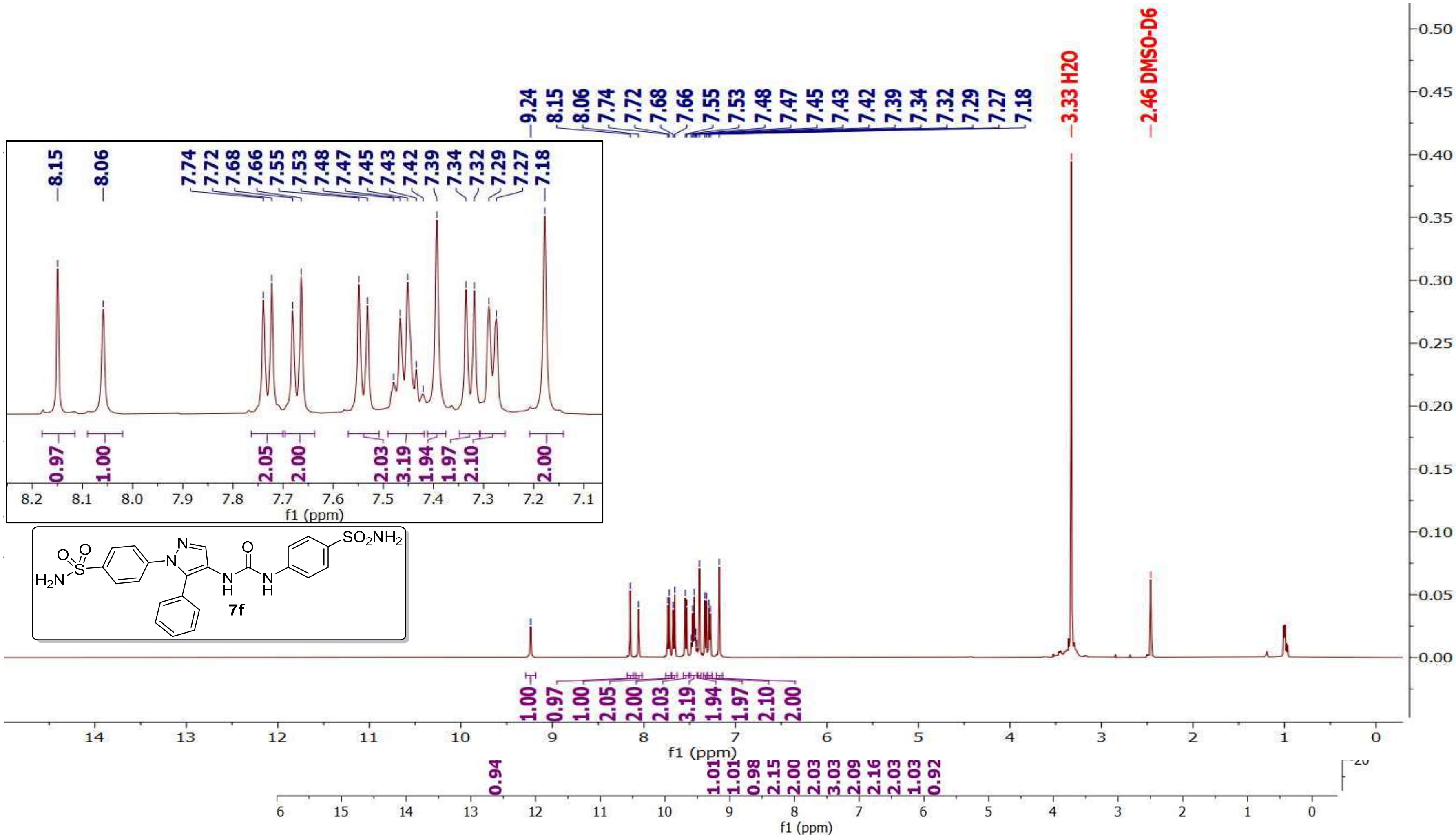

Figure S3. ^{1}H NMR spectrum of compound **7f.**

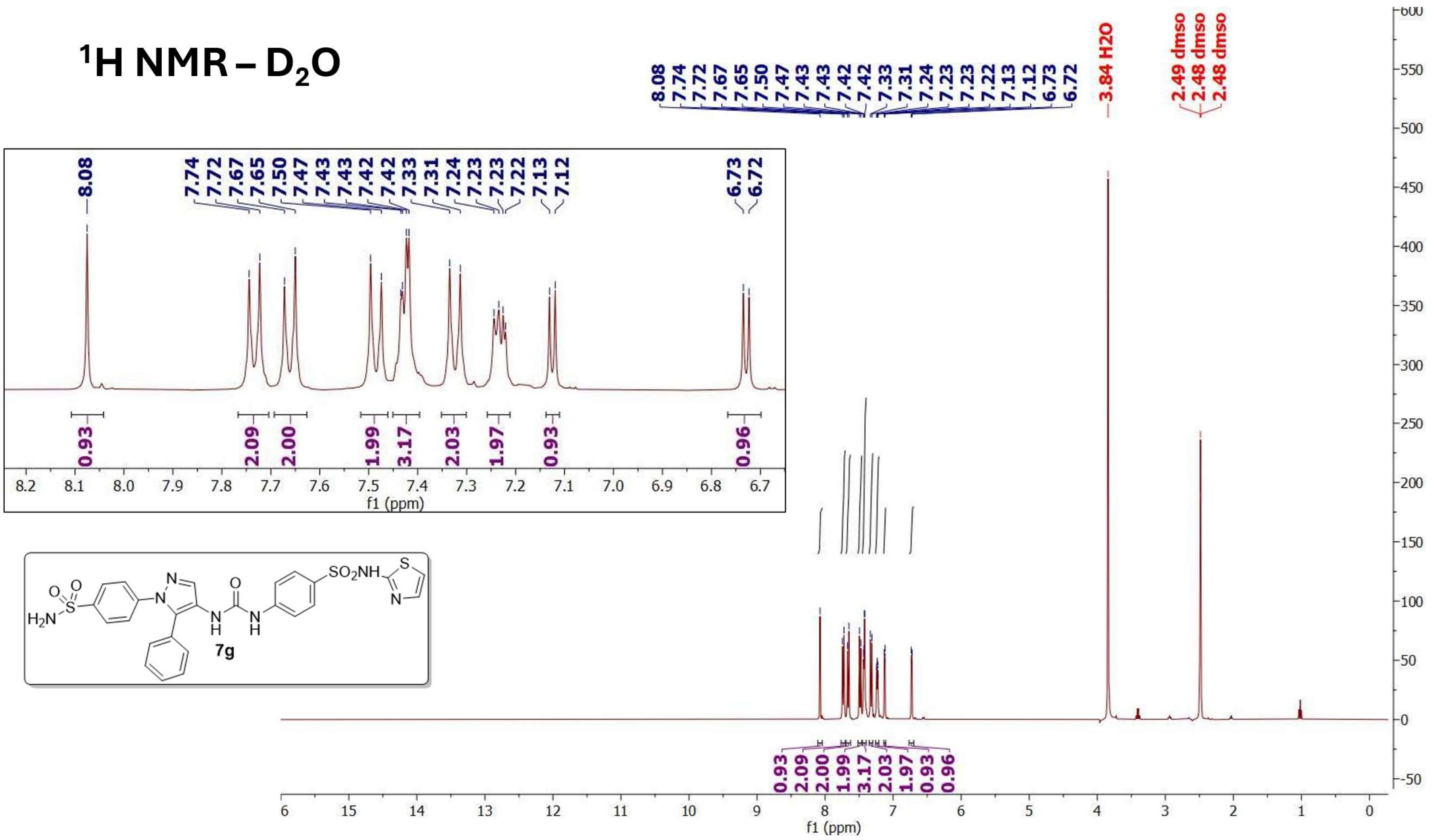

Figure S4. ^{1}H NMR spectrum of compound **7g** (D_2O).

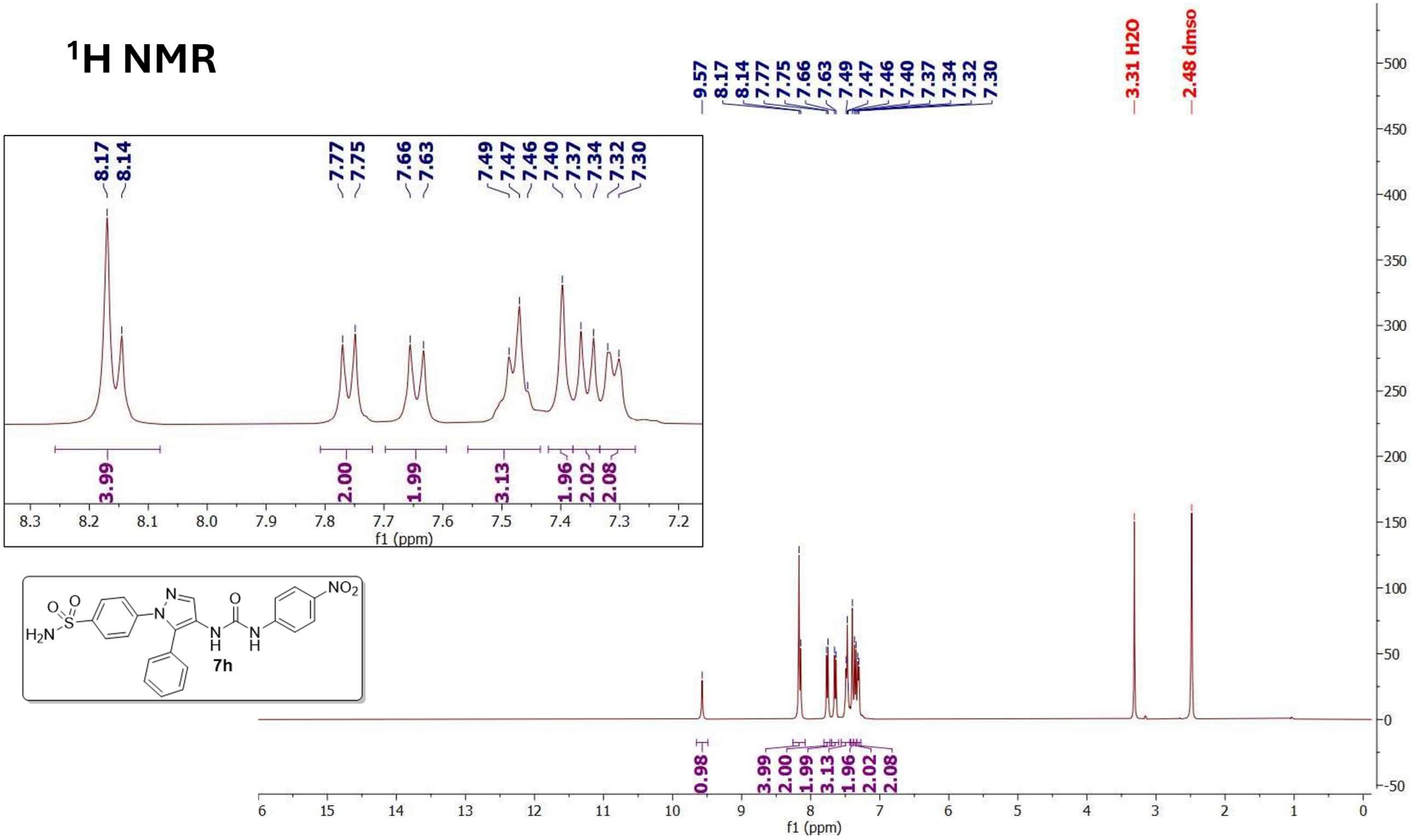

Figure S5. ¹H NMR spectrum of compound **7h.**

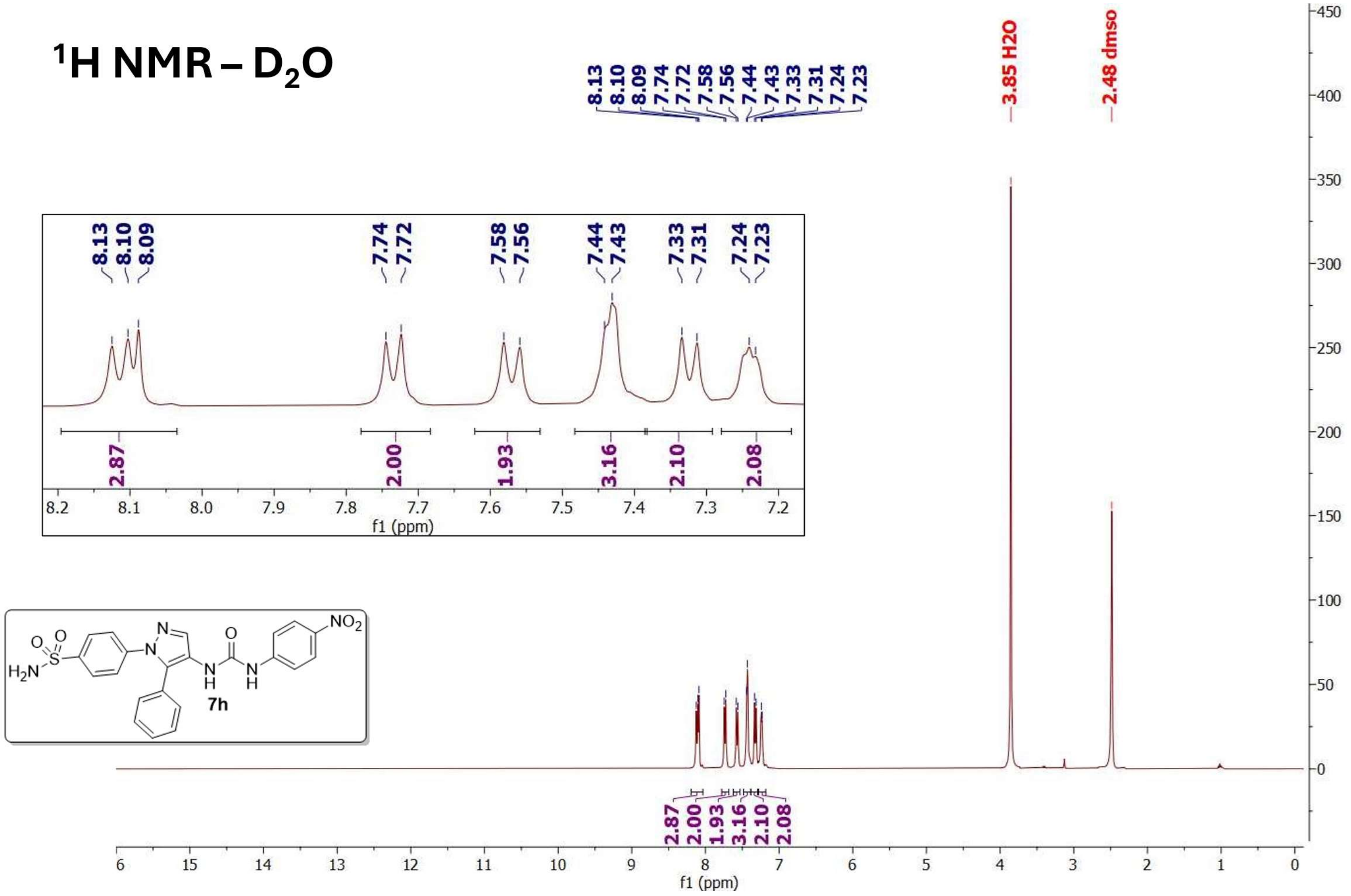

Figure S6. ^{1}H NMR spectrum of compound **7h** (D_2O).

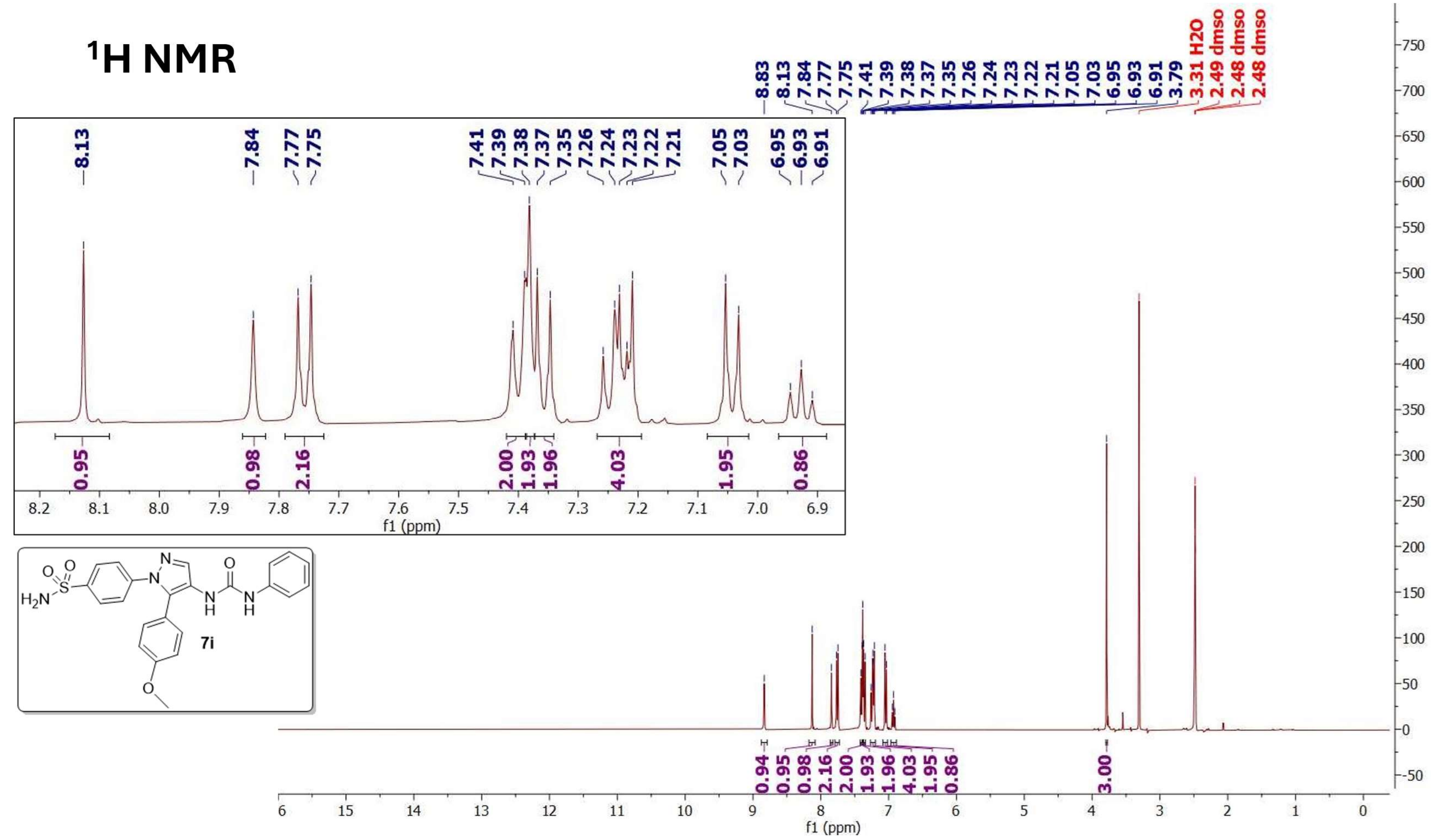

Figure S7. ^{1}H NMR spectrum of compound **7i.**

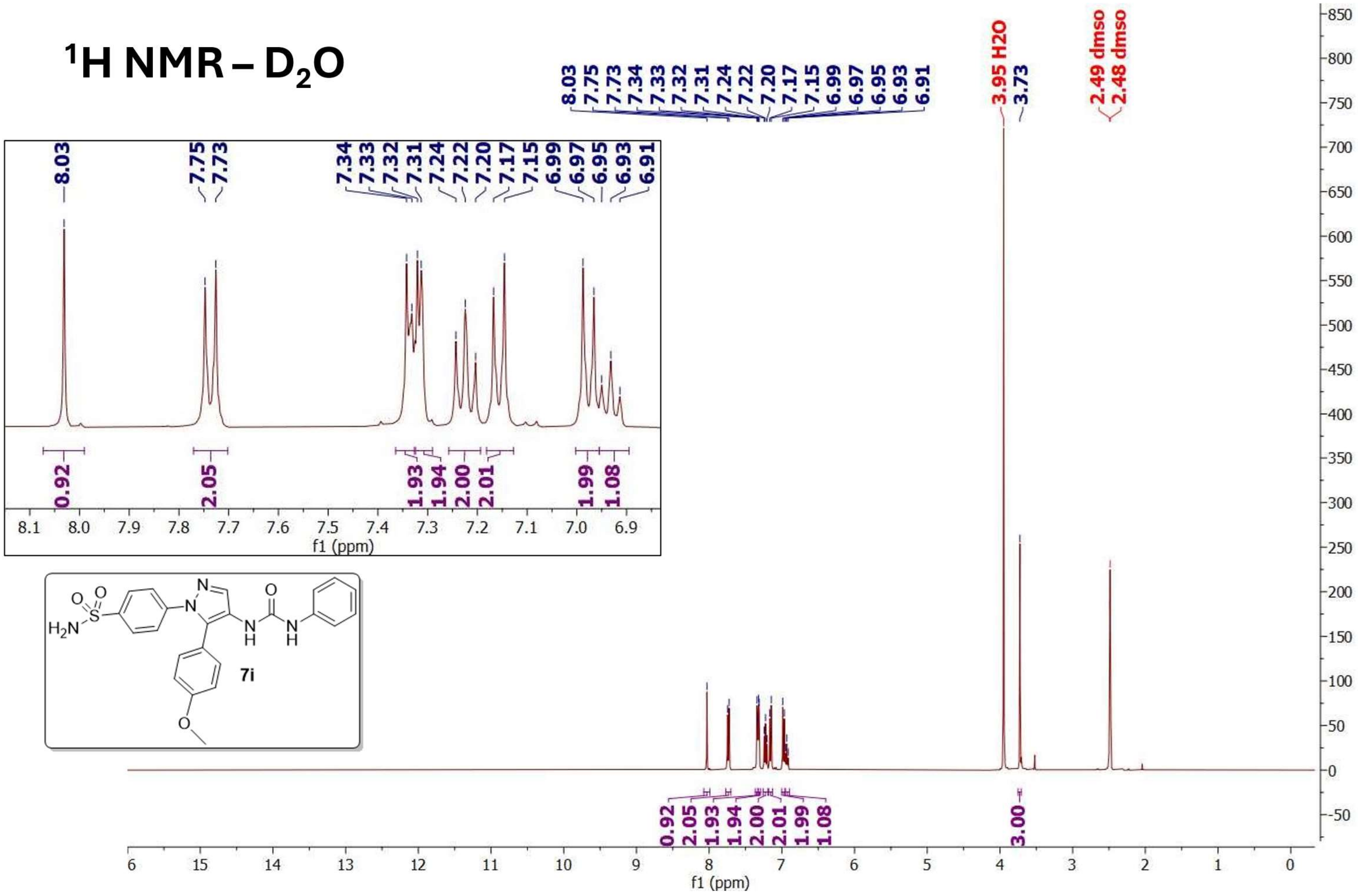

Figure S8. ^{1}H NMR spectrum of compound **7i** (D_2O).

^{13}C NMR

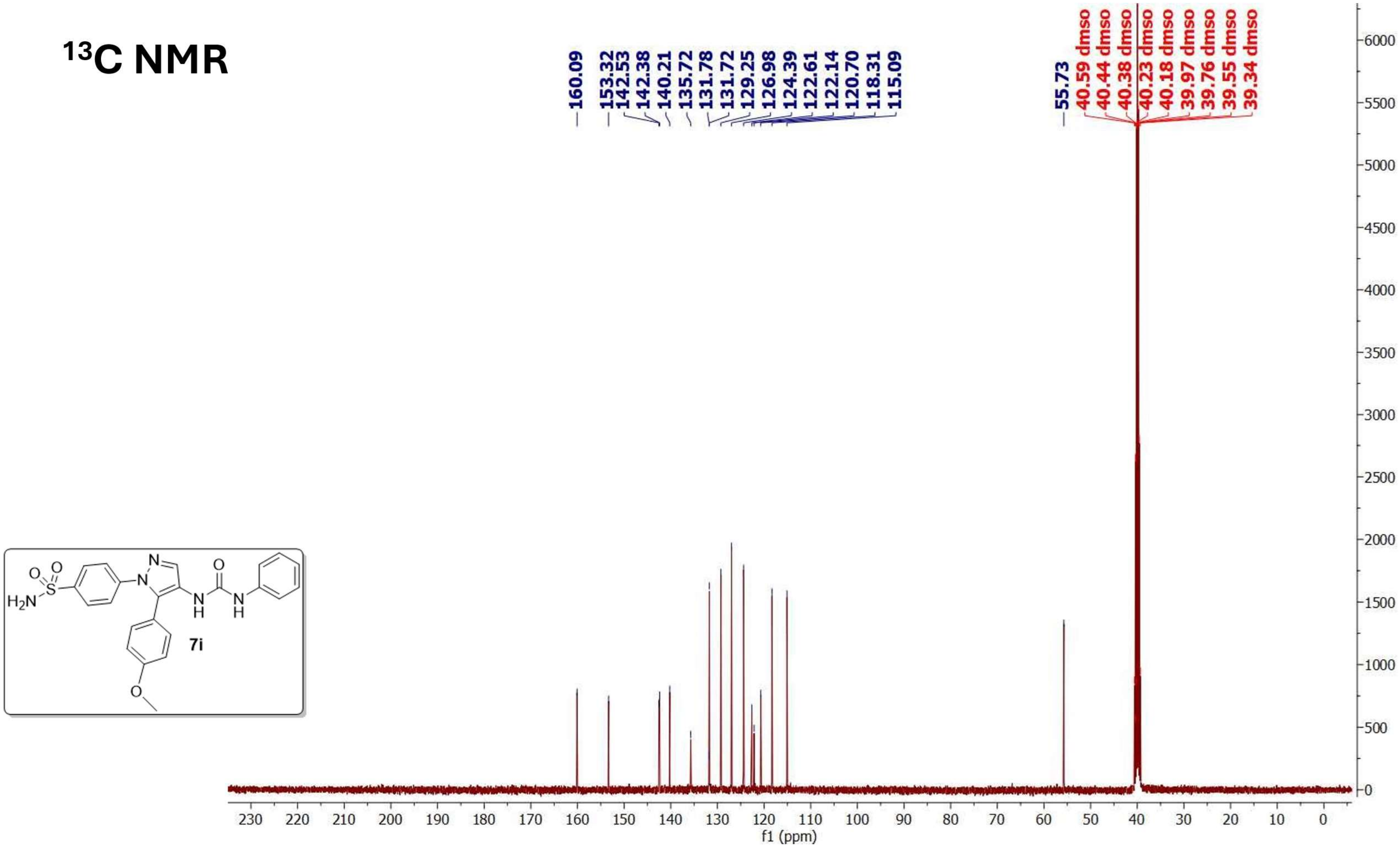

Figure S9. ^{13}C NMR spectrum of compound **7i.**

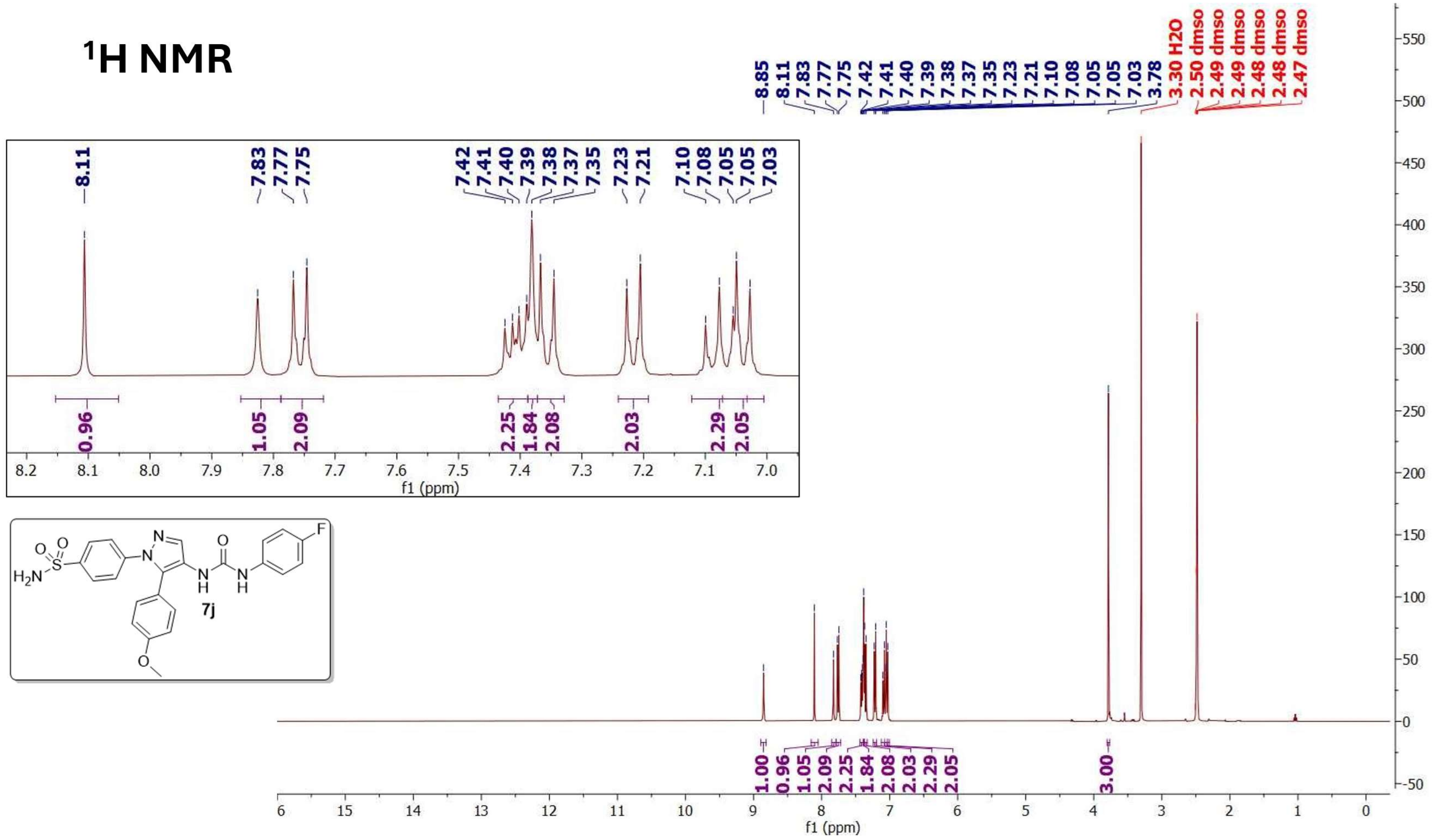

Figure S10. ^{1}H NMR spectrum of compound **7j.**

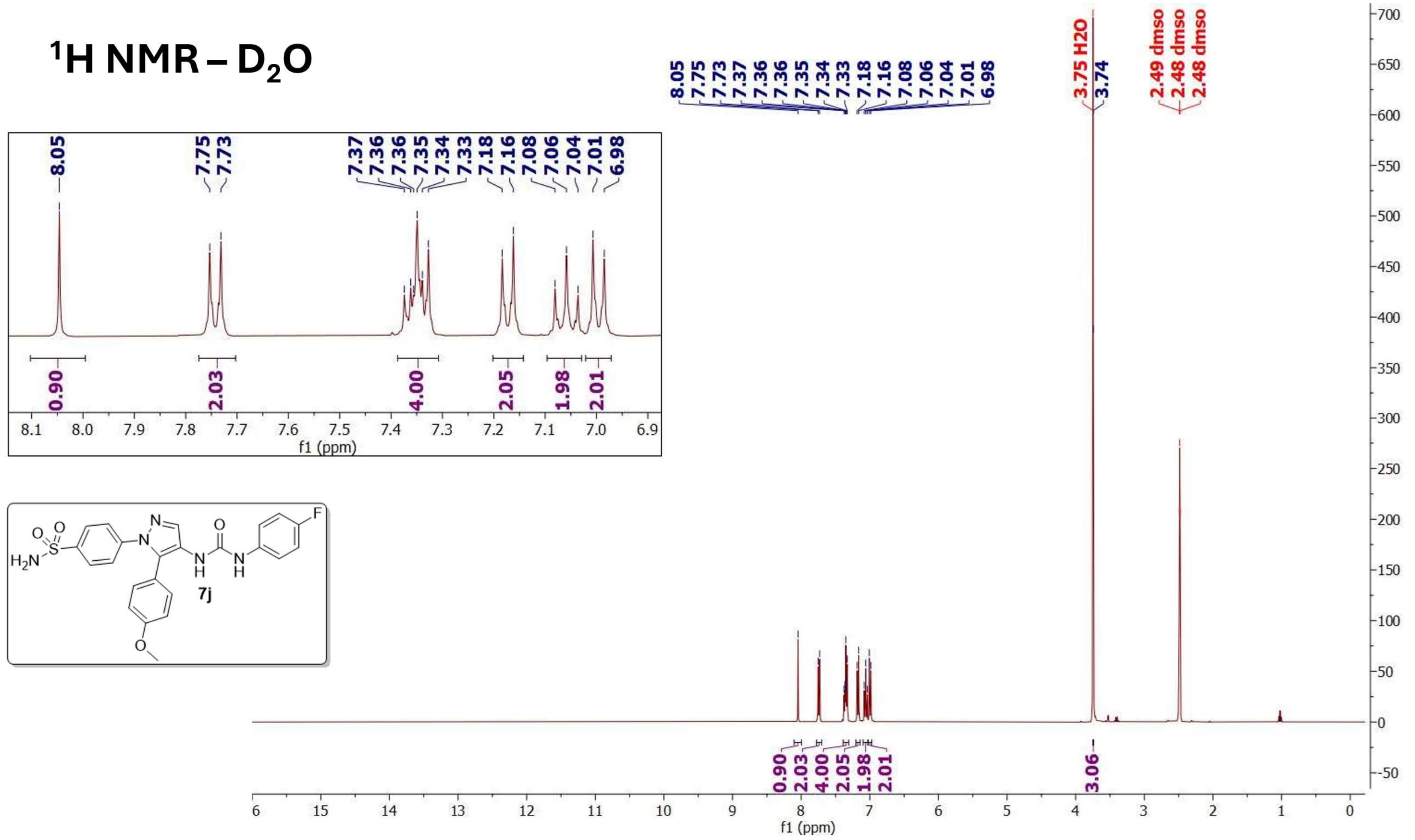

Figure S11. ¹H NMR spectrum of compound **7j** (D_2O).

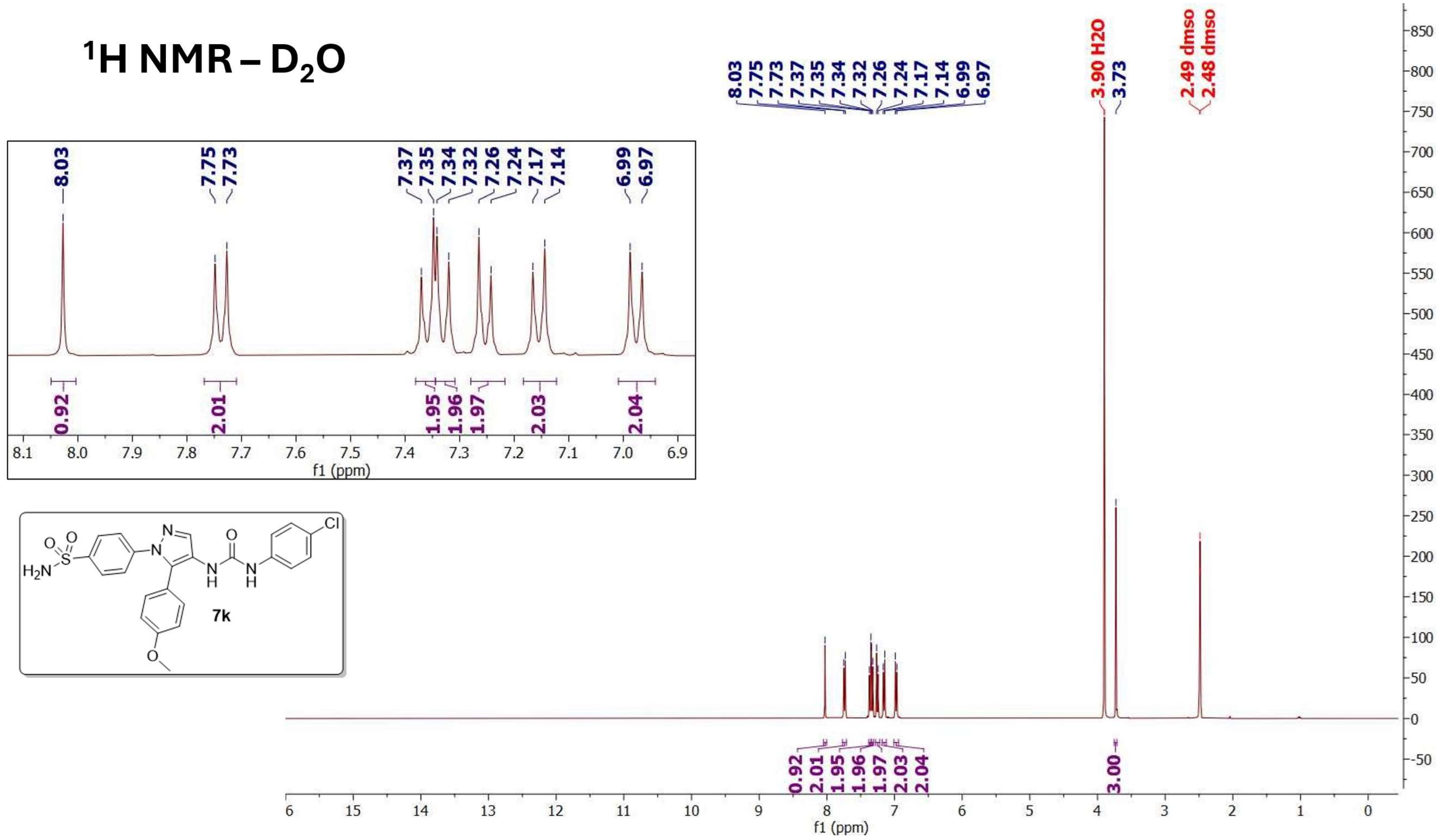

Figure S12. ^{1}H NMR spectrum of compound **7k** (D_2O).

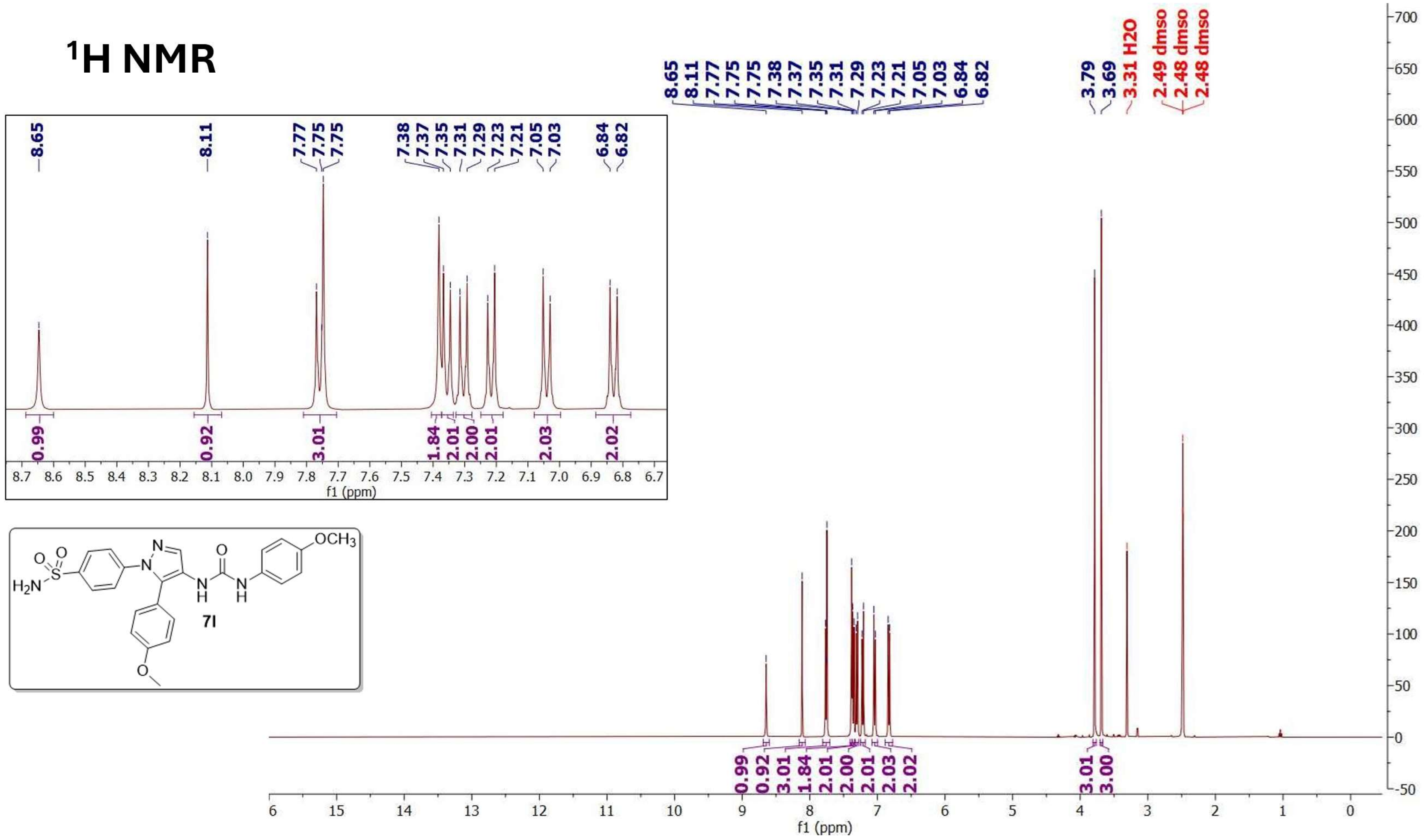

Figure S13. ^{1}H NMR spectrum of compound **7l.**

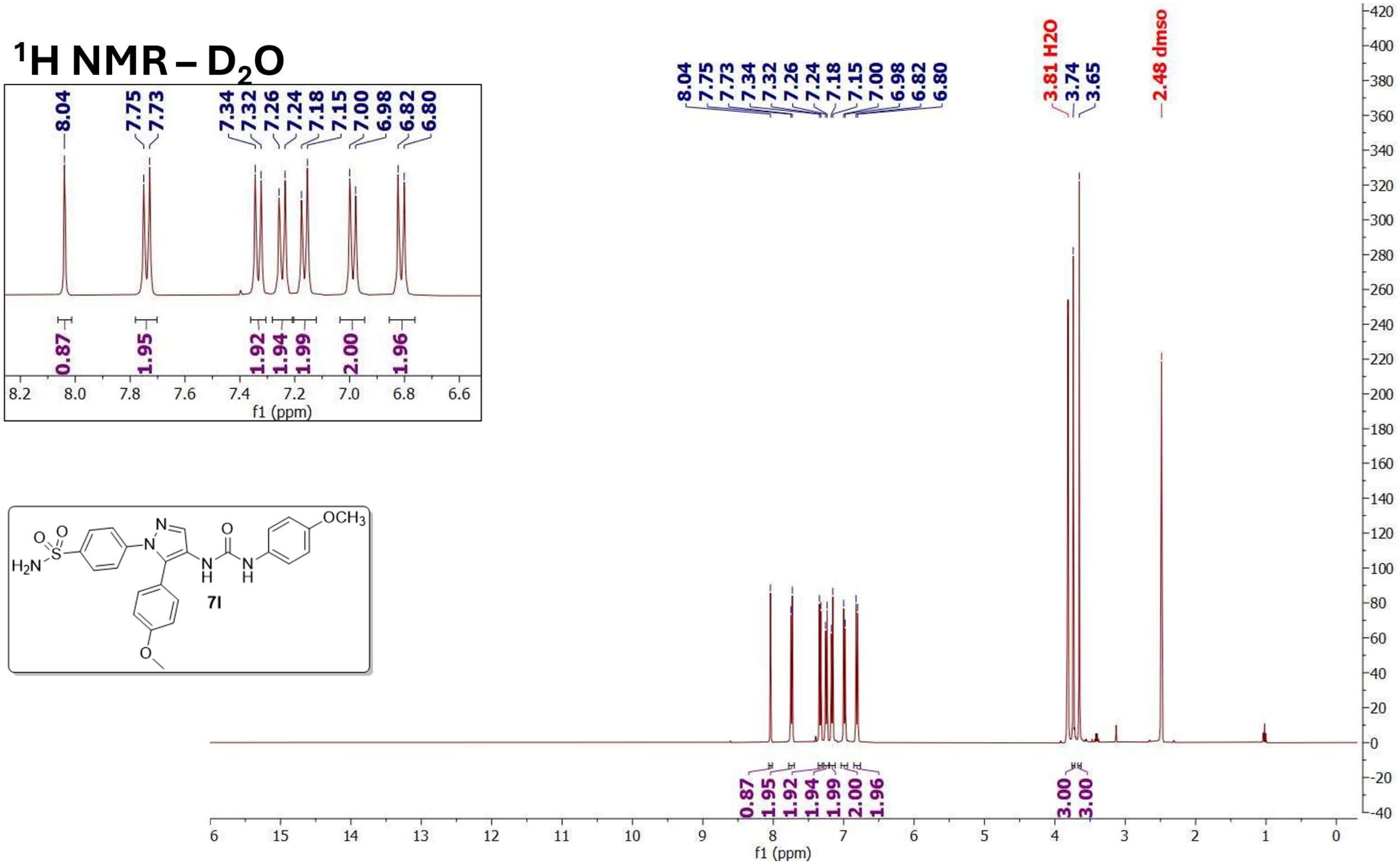

Figure S14. ^{1}H NMR spectrum of compound **7l** (D_2O).

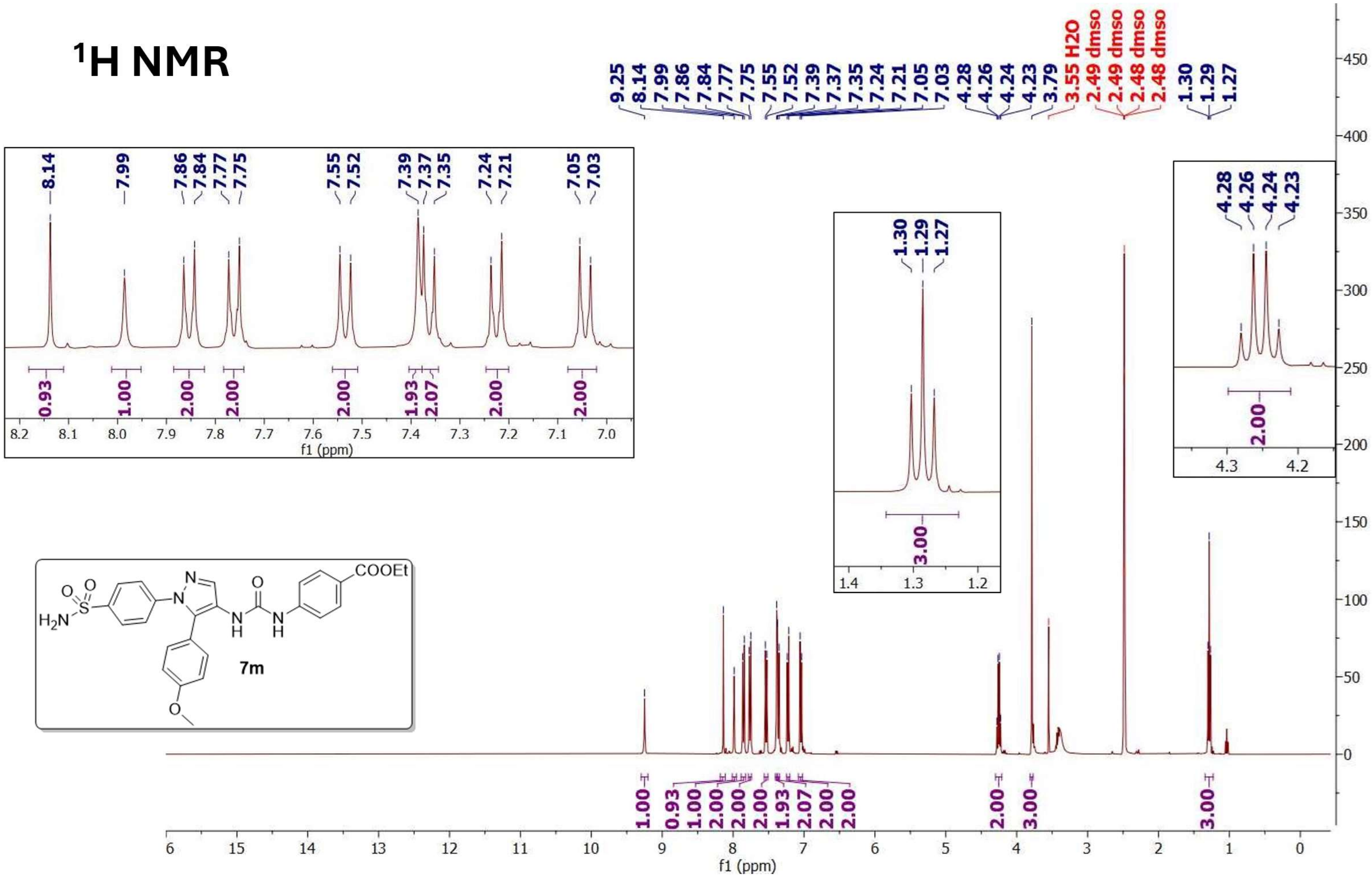

Figure S15. ^{1}H NMR spectrum of compound **7m.**

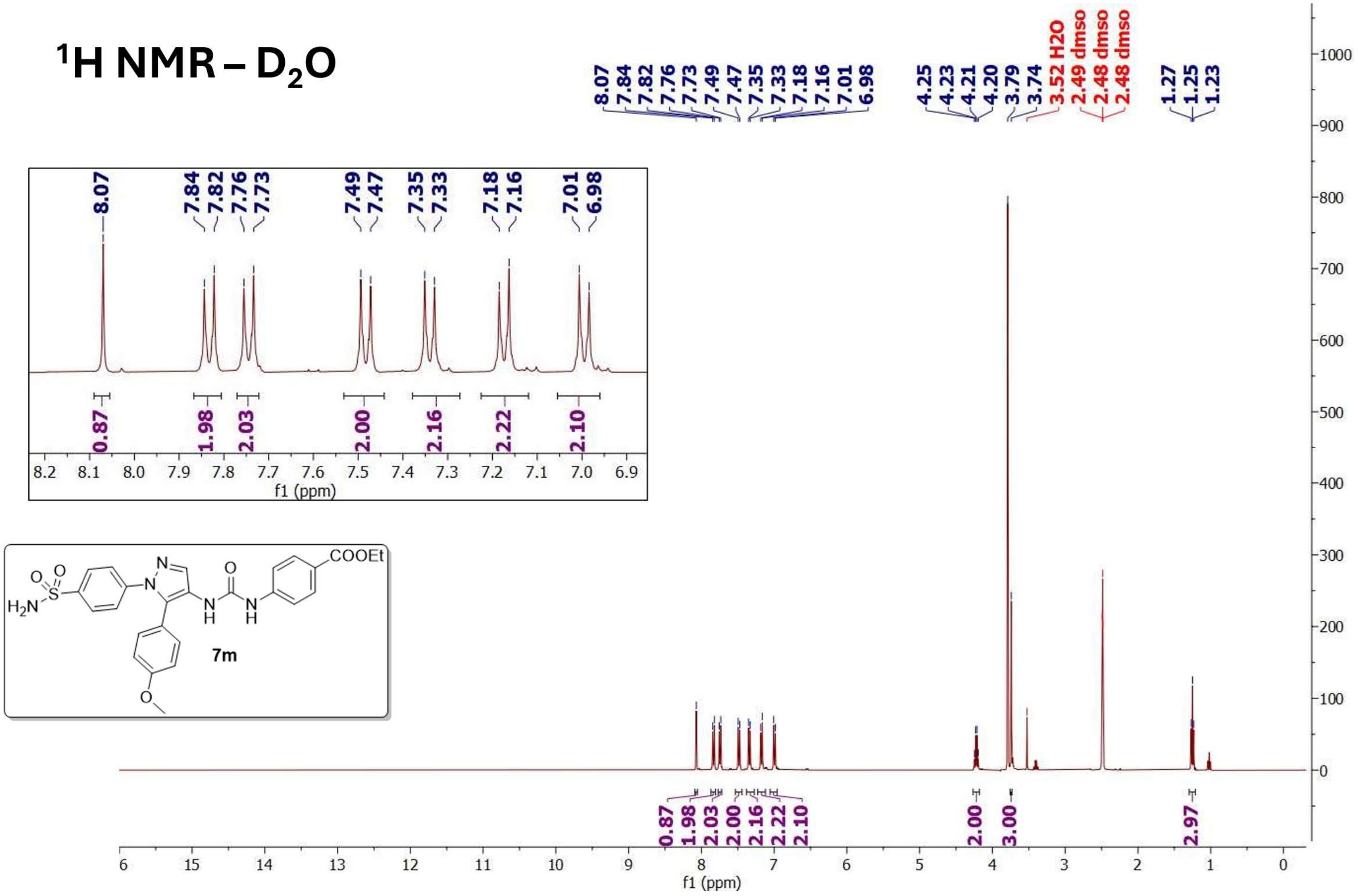

Figure S16. ^{1}H NMR spectrum of compound **7m** (D_2O).

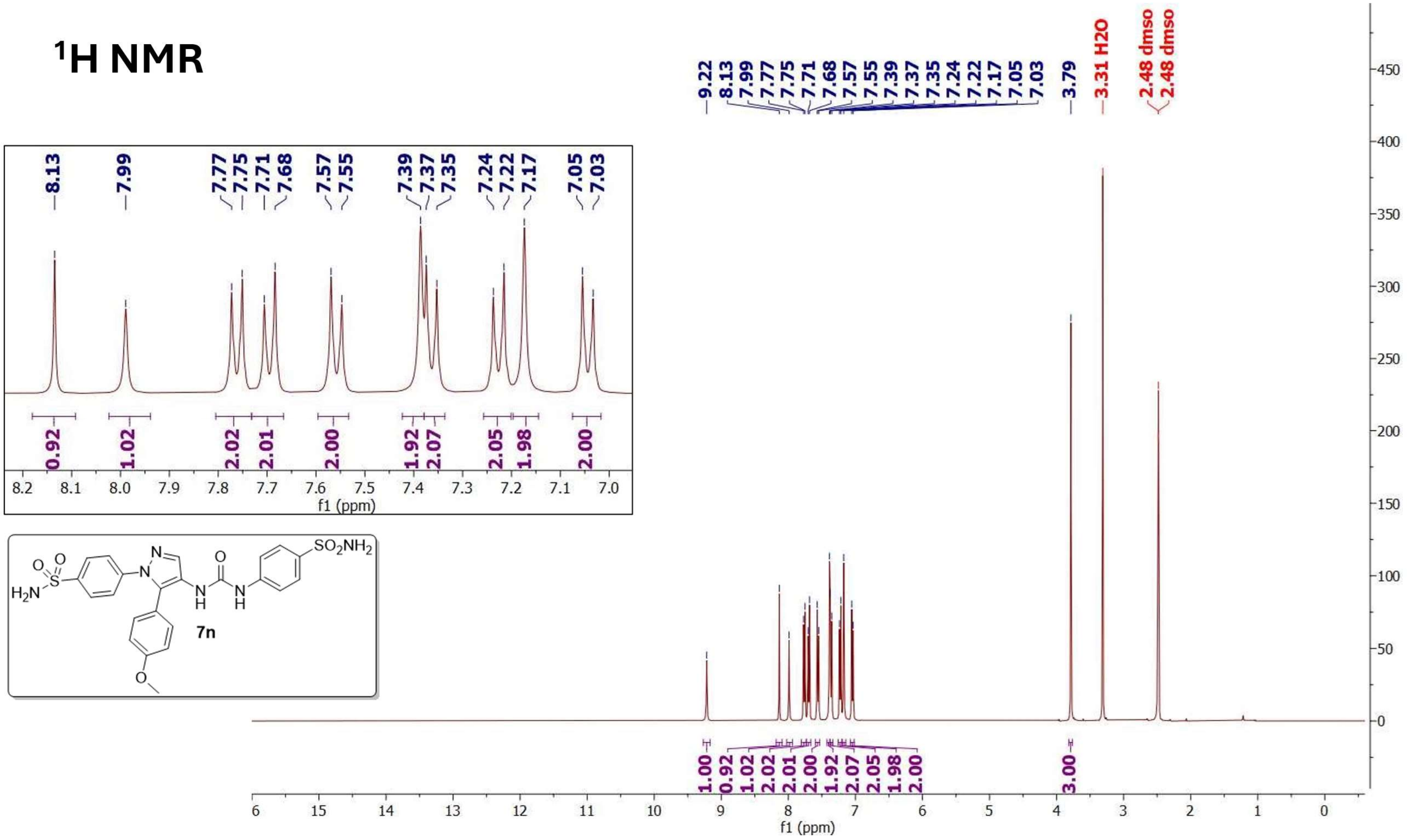

Figure S17. ^{1}H NMR spectrum of compound **7n.**

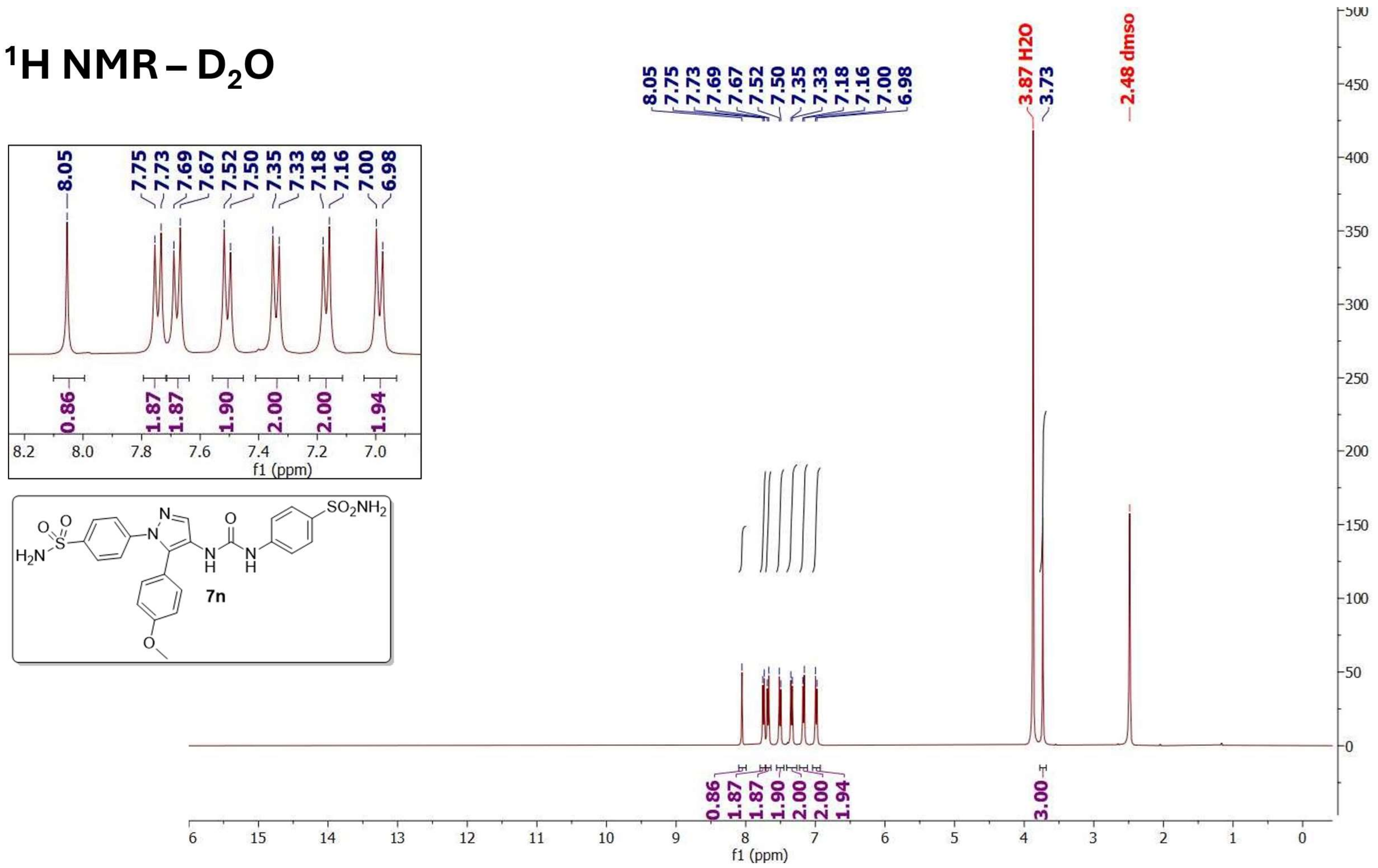

Figure S18. ^{1}H NMR spectrum of compound **7n** (D_2O).

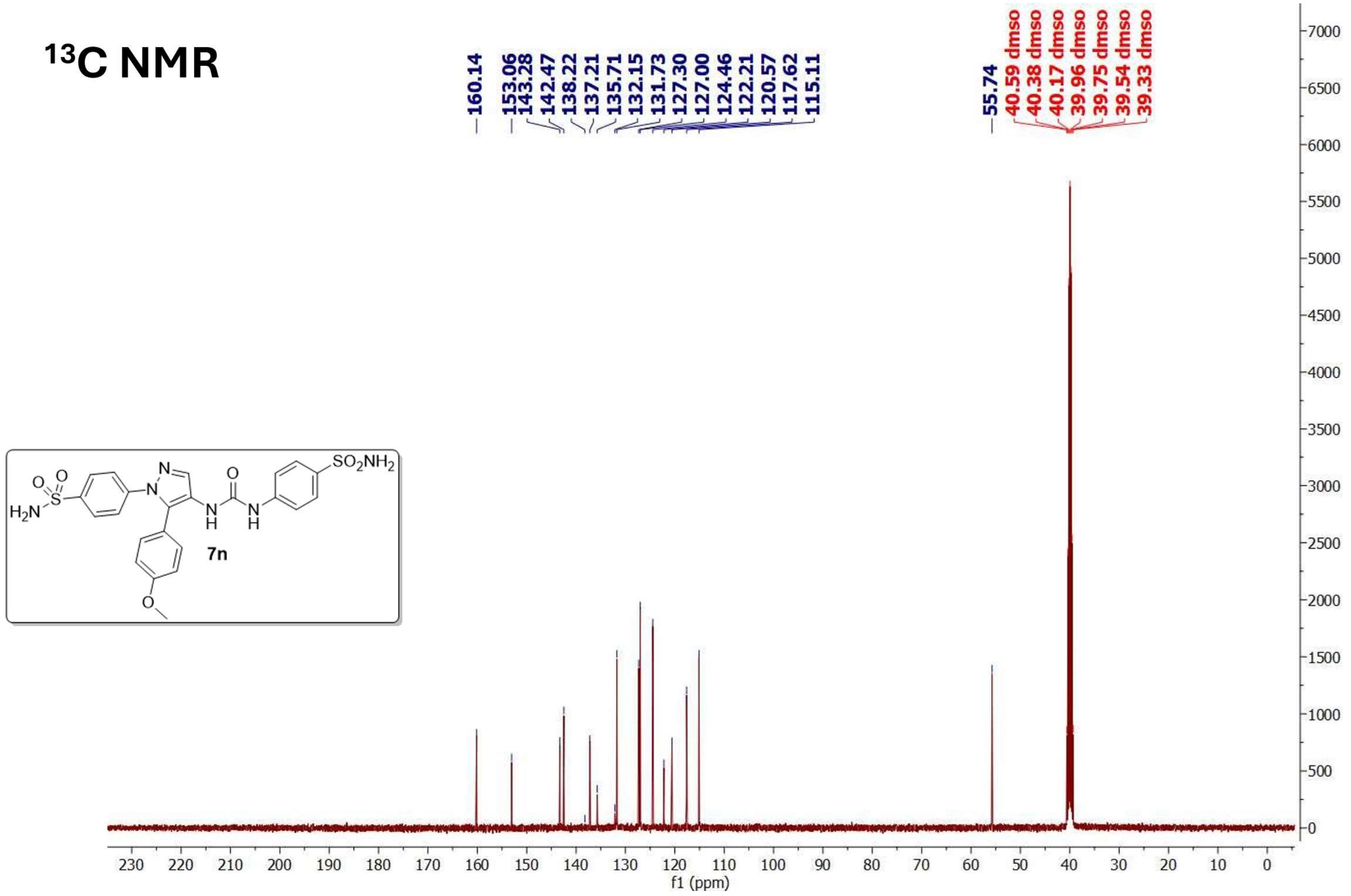

Figure S19. ^{13}C NMR spectrum of compound **7n.**

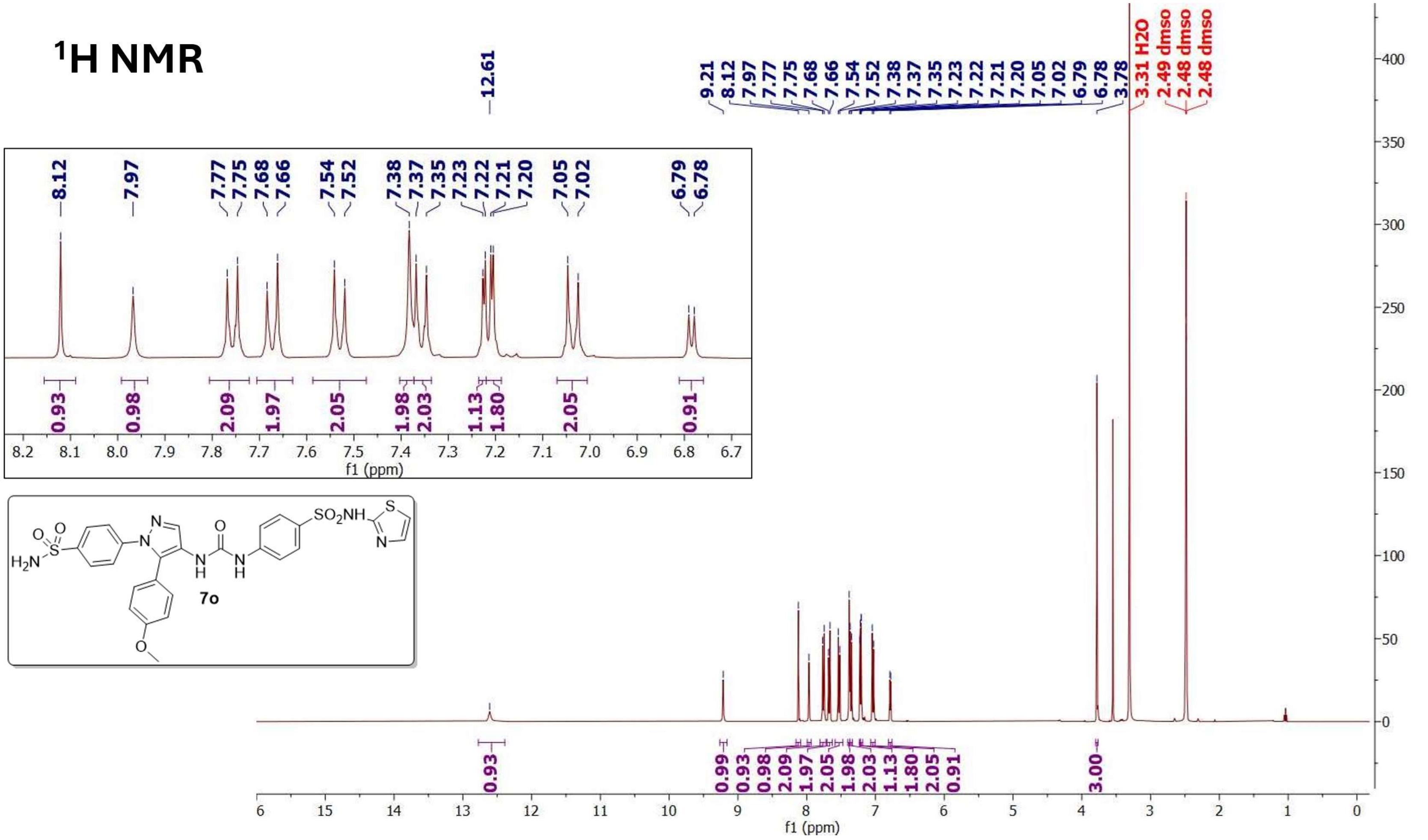

Figure S20. ^{1}H NMR spectrum of compound **7o.**

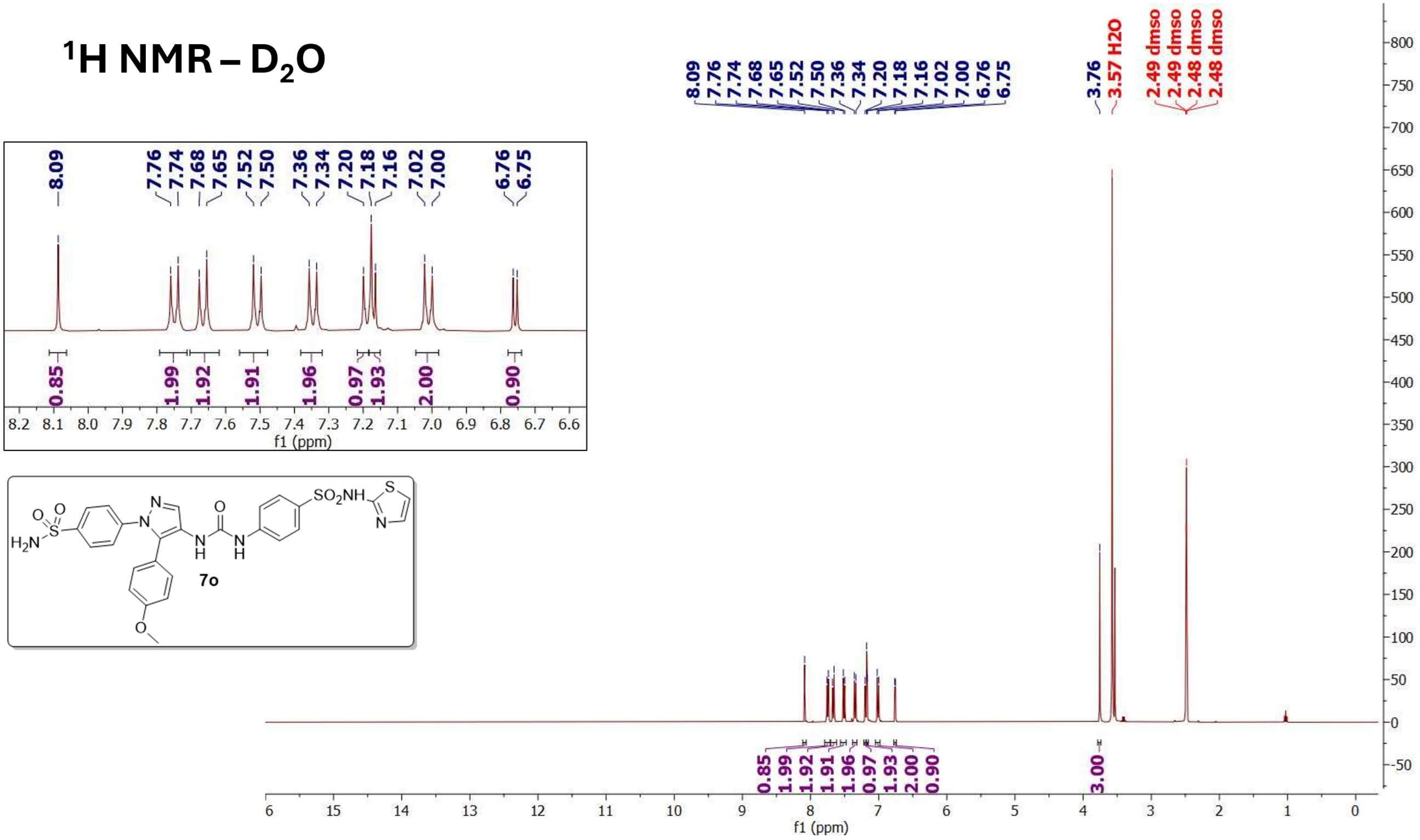

Figure S21. ^{1}H NMR spectrum of compound **7o** (D_2O).

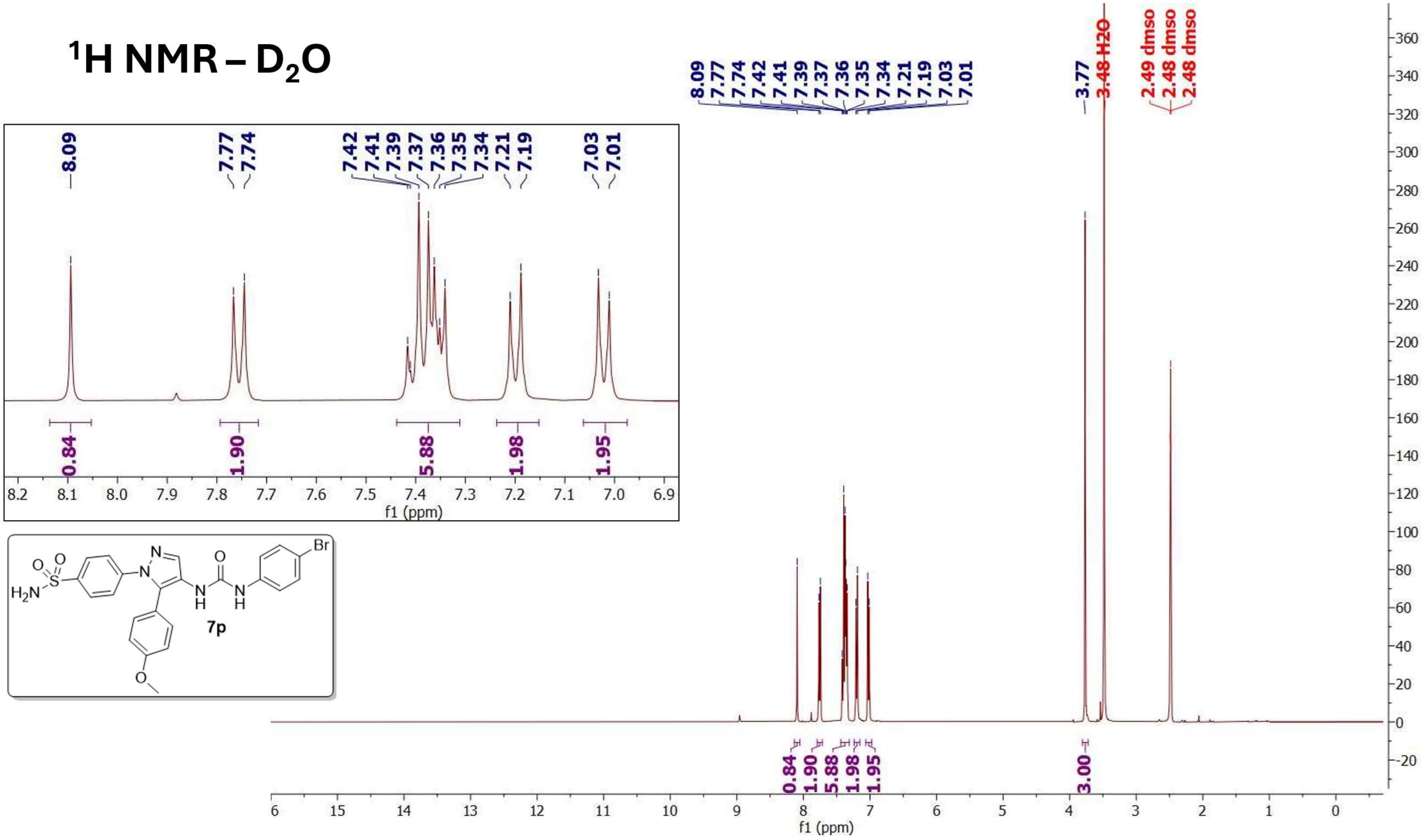

Figure S22. ¹H NMR spectrum of compound **7p** (D_2O).

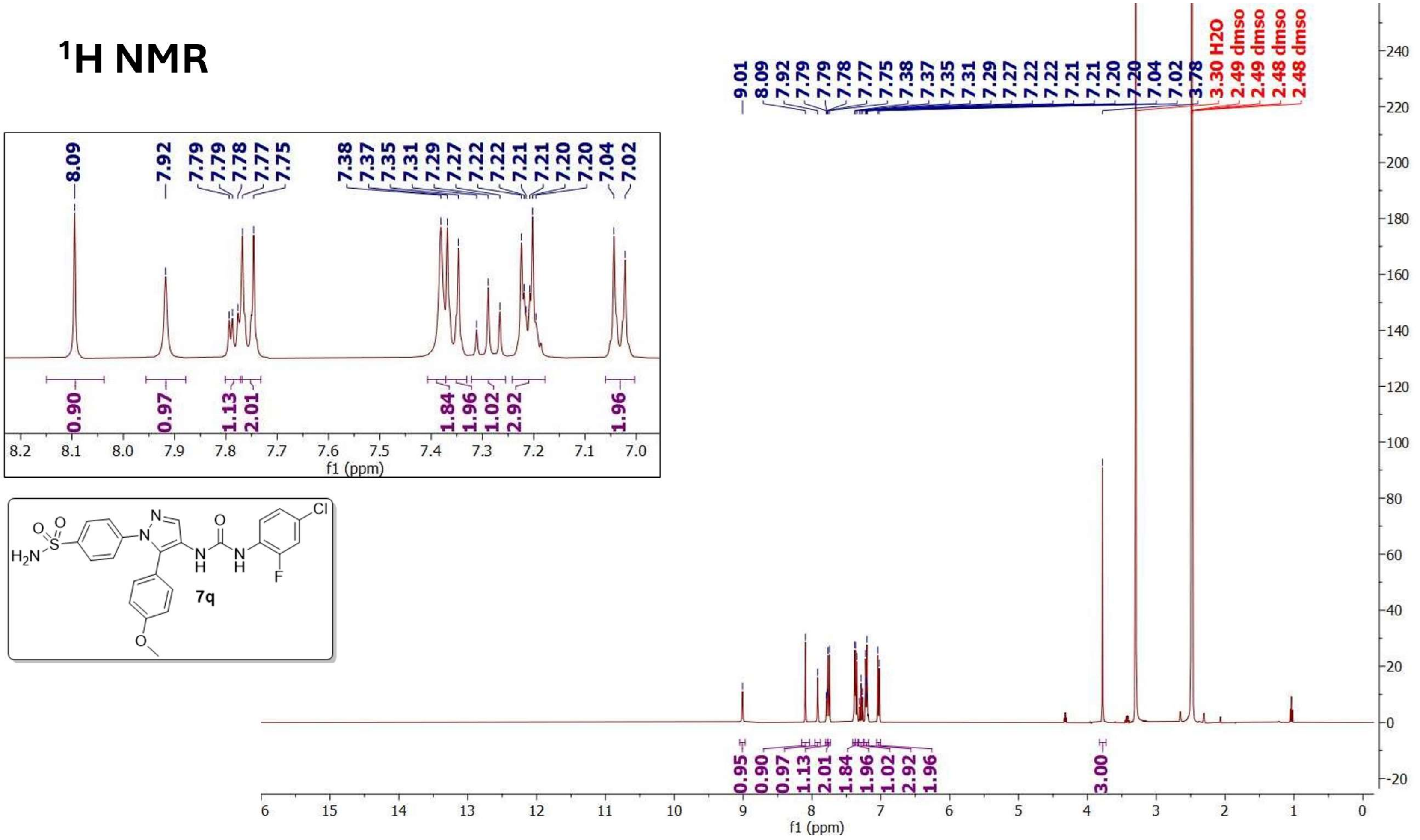

Figure S23. ^{1}H NMR spectrum of compound **7q.**

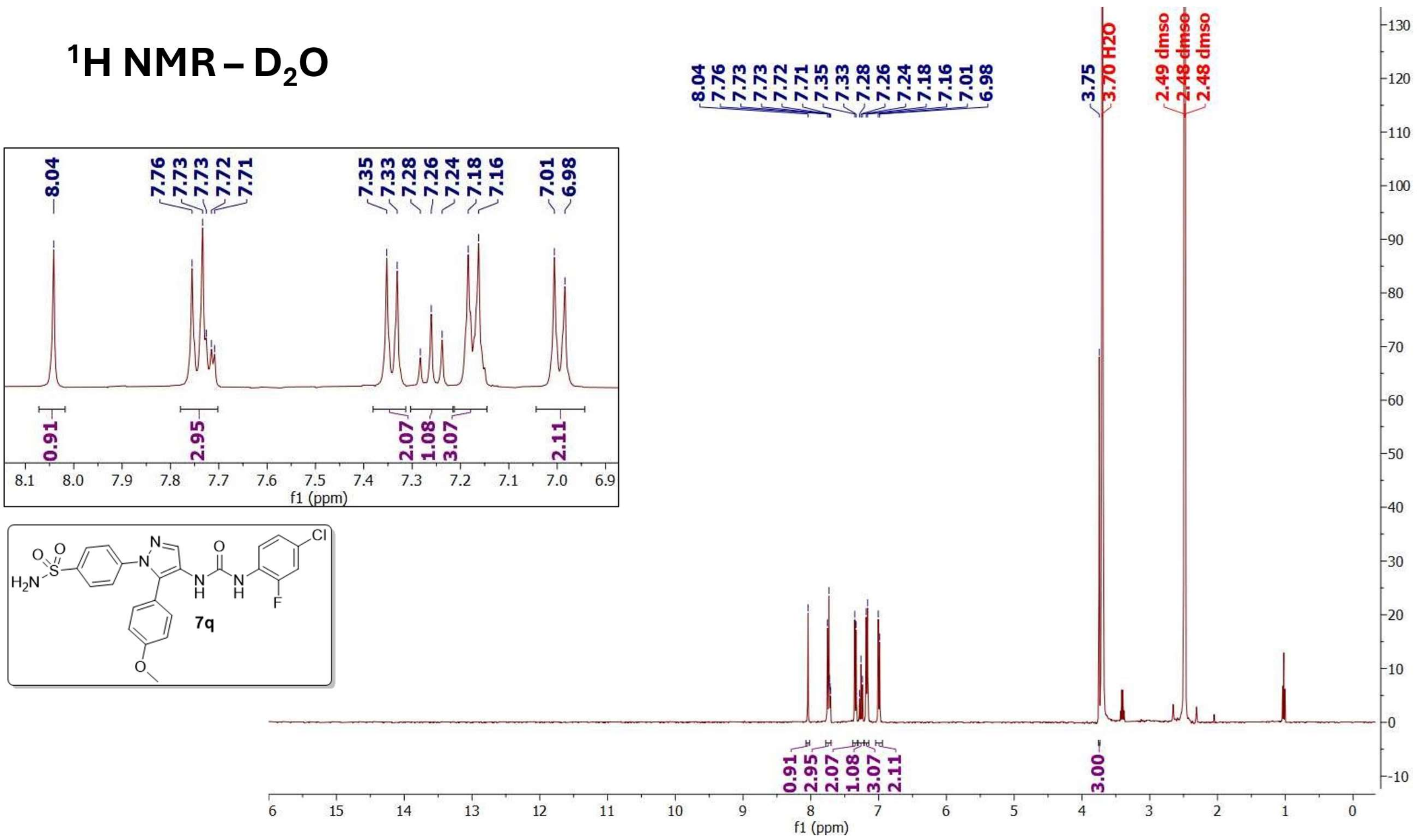

Figure S24. ^{1}H NMR spectrum of compound **7q.**

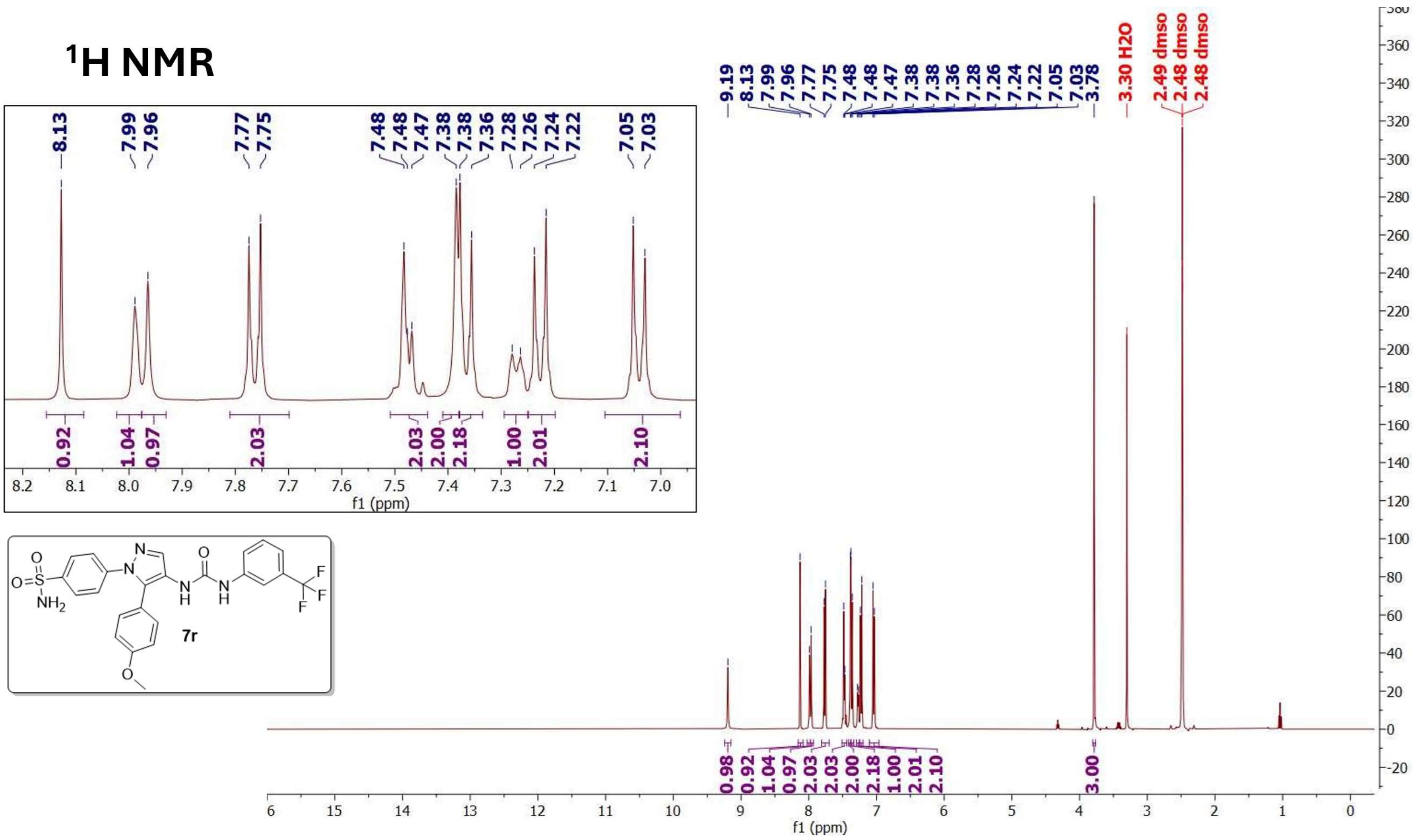

Figure S24. ^{1}H NMR spectrum of compound **7r.**

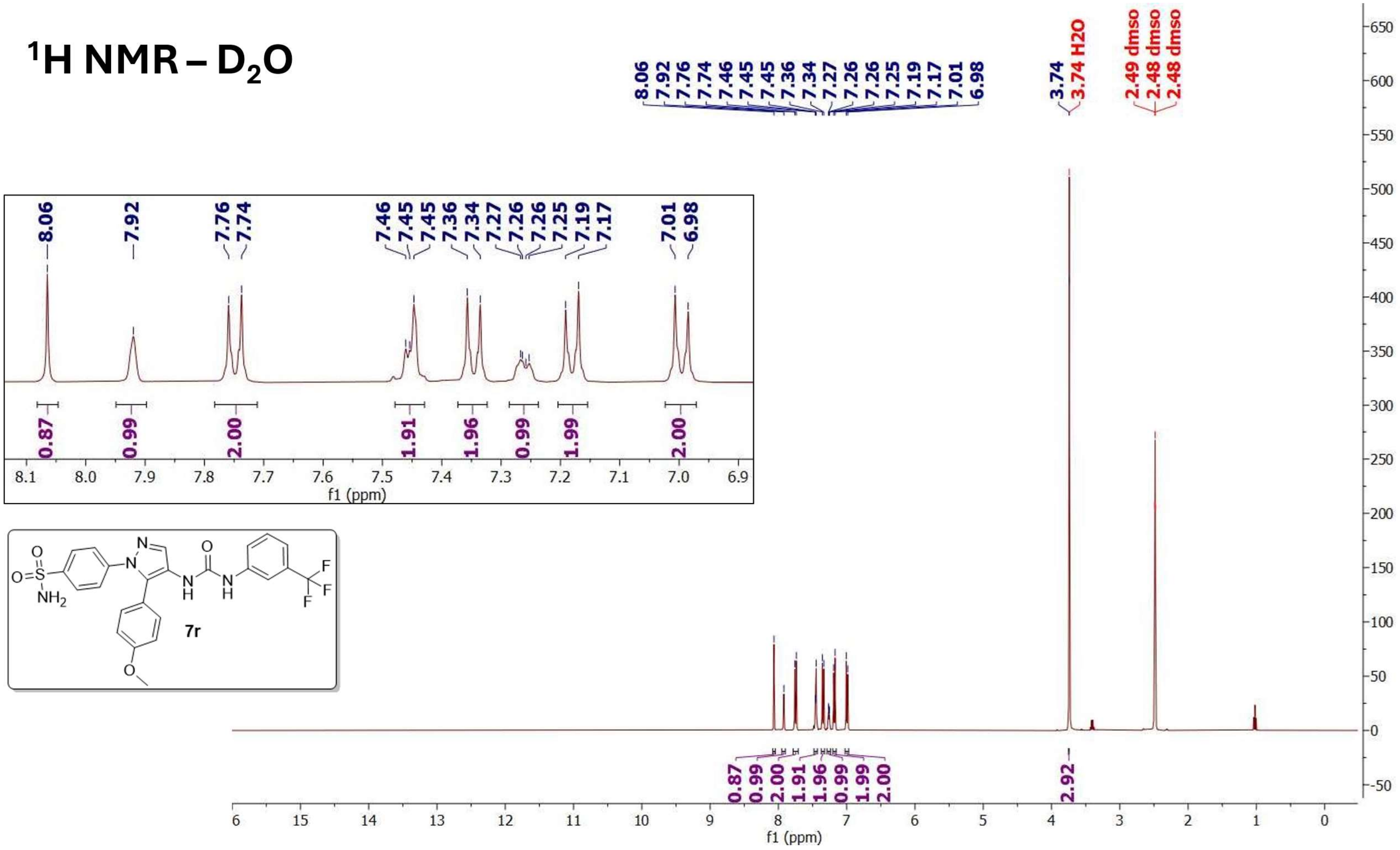

Figure S26. ^{1}H NMR spectrum of compound **7r** (D_2O).

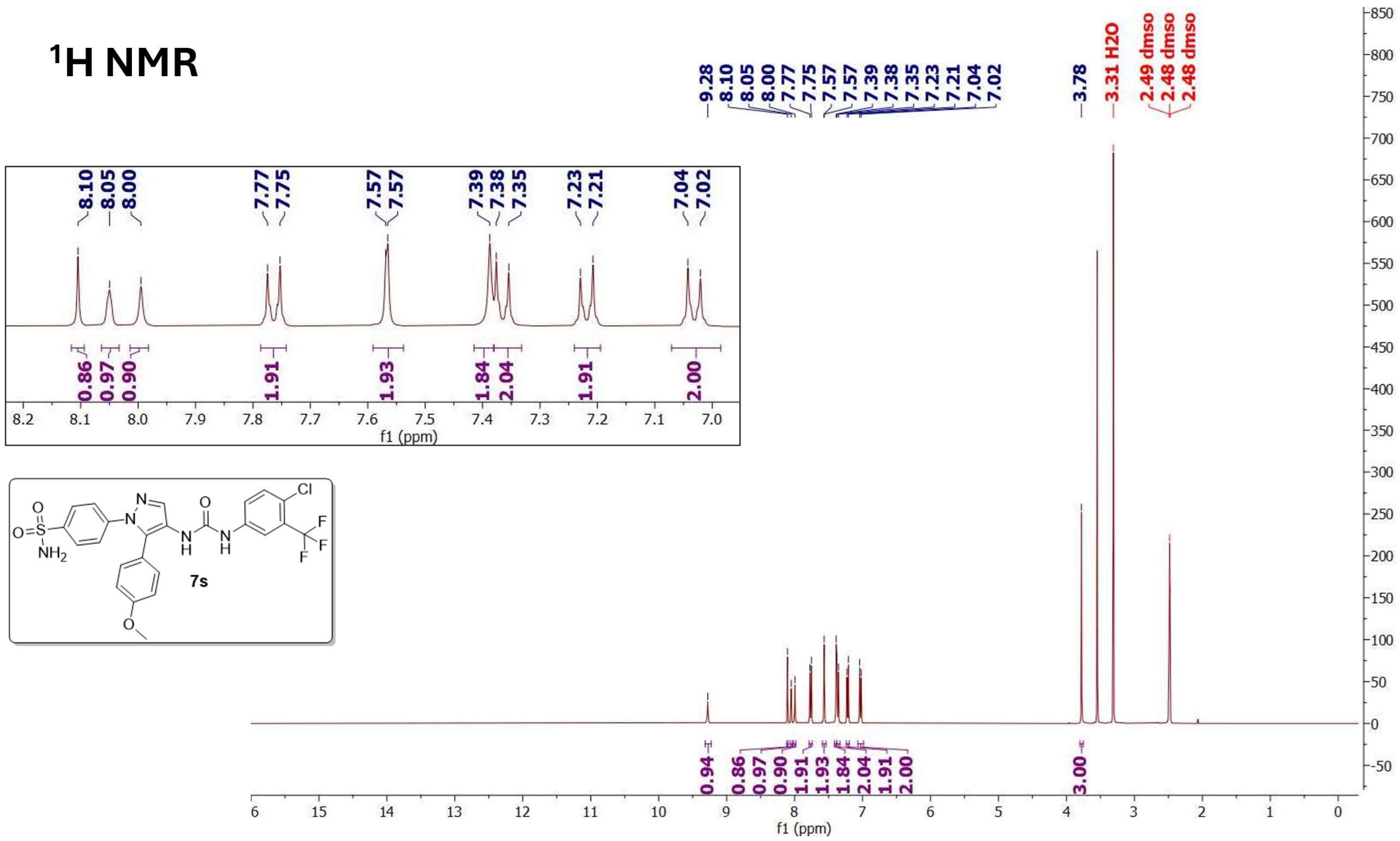

Figure S27. ^{1}H NMR spectrum of compound **7s.**

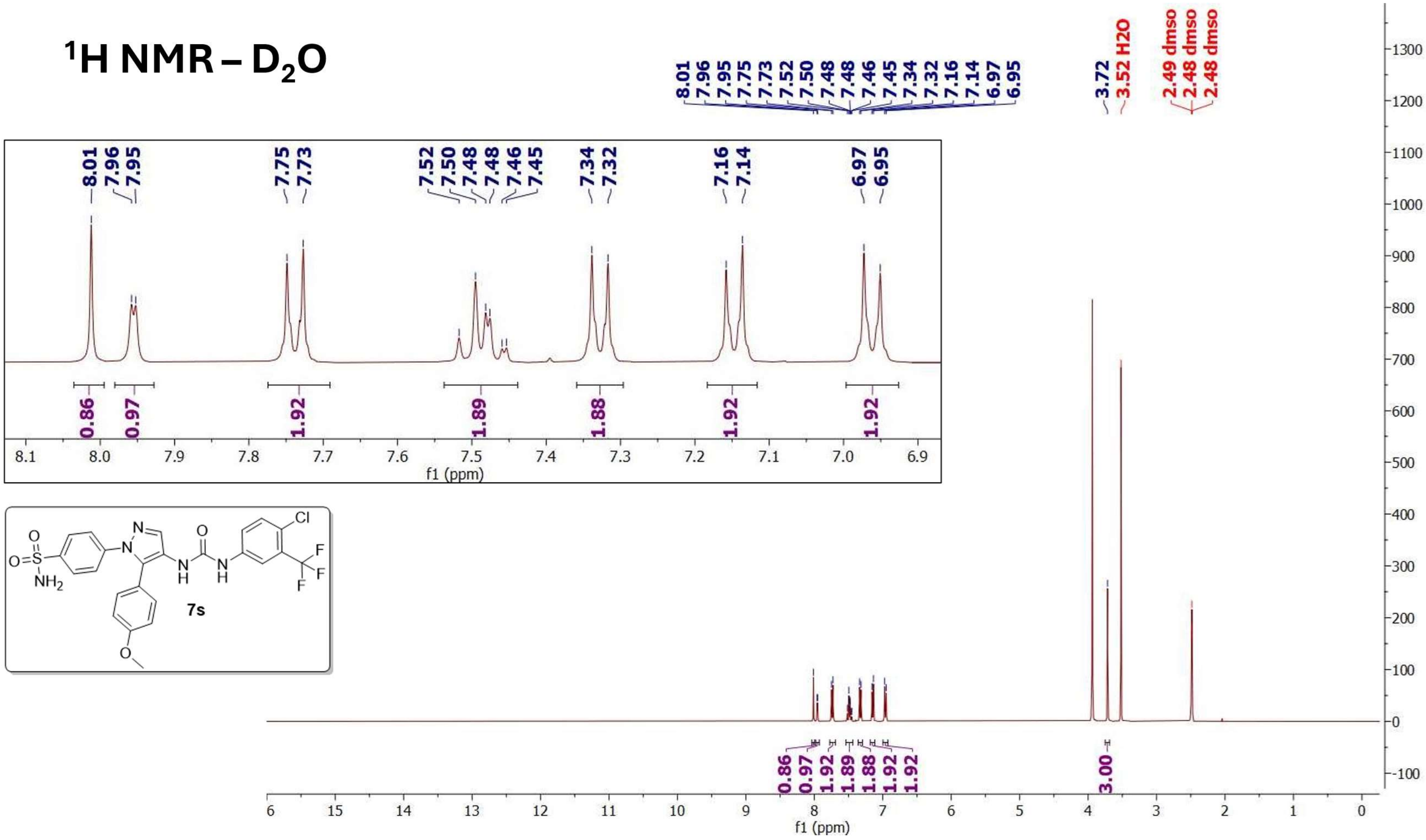

Figure S28. ^{1}H NMR spectrum of compound **7s** (D_2O).

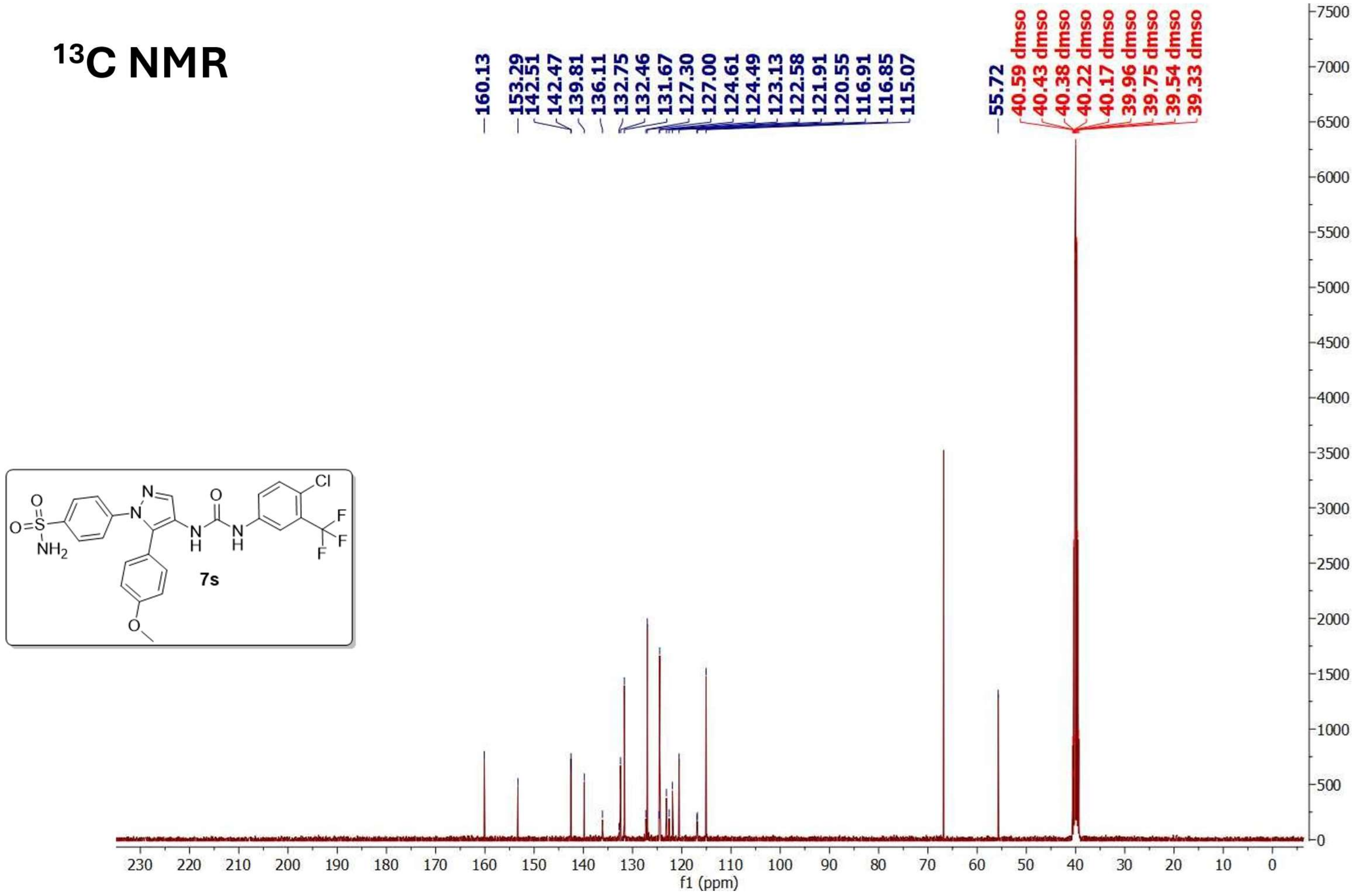

Figure S29. ^{13}C NMR spectrum of compound **7s.**

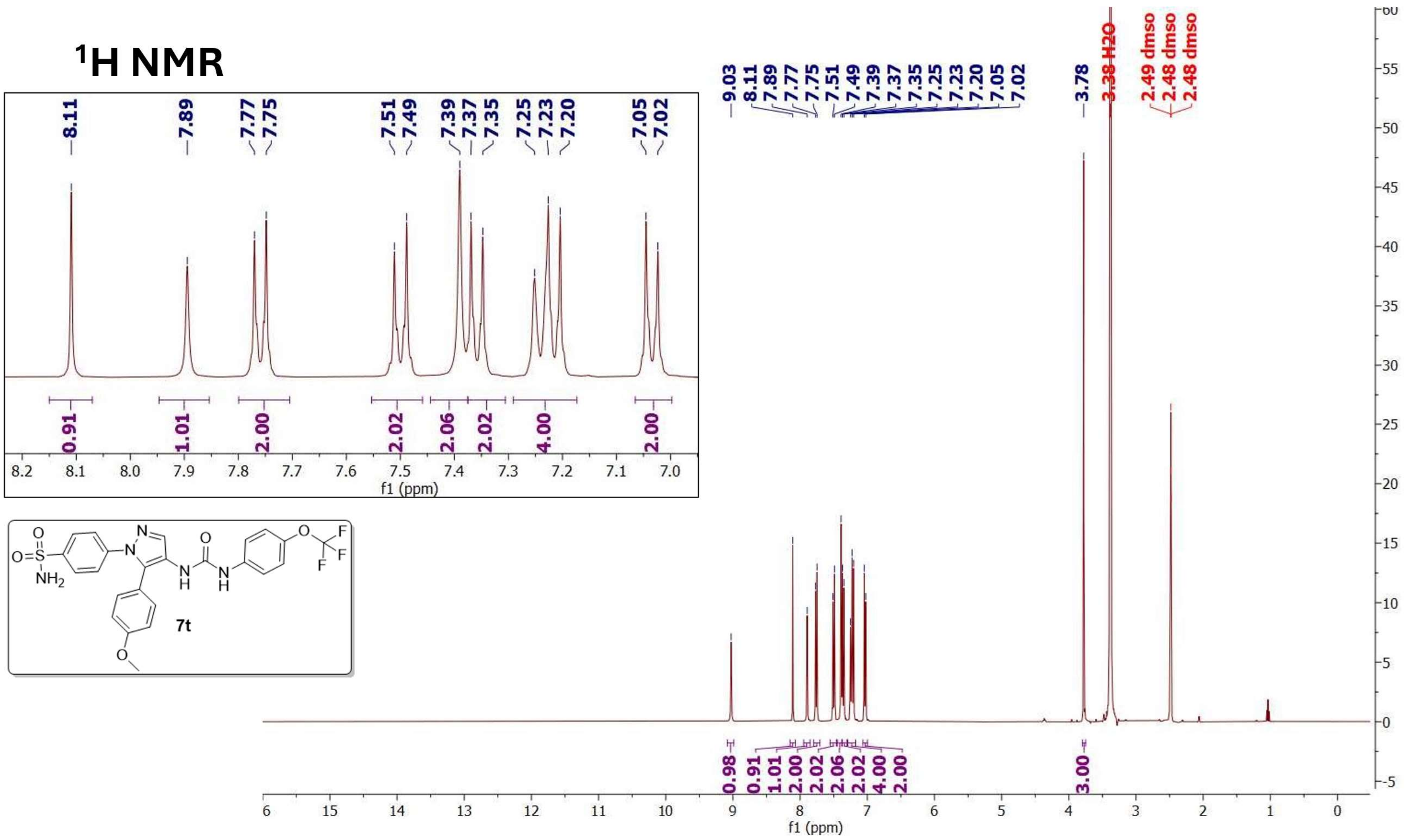

Figure S30. ^{1}H NMR spectrum of compound **7t.**

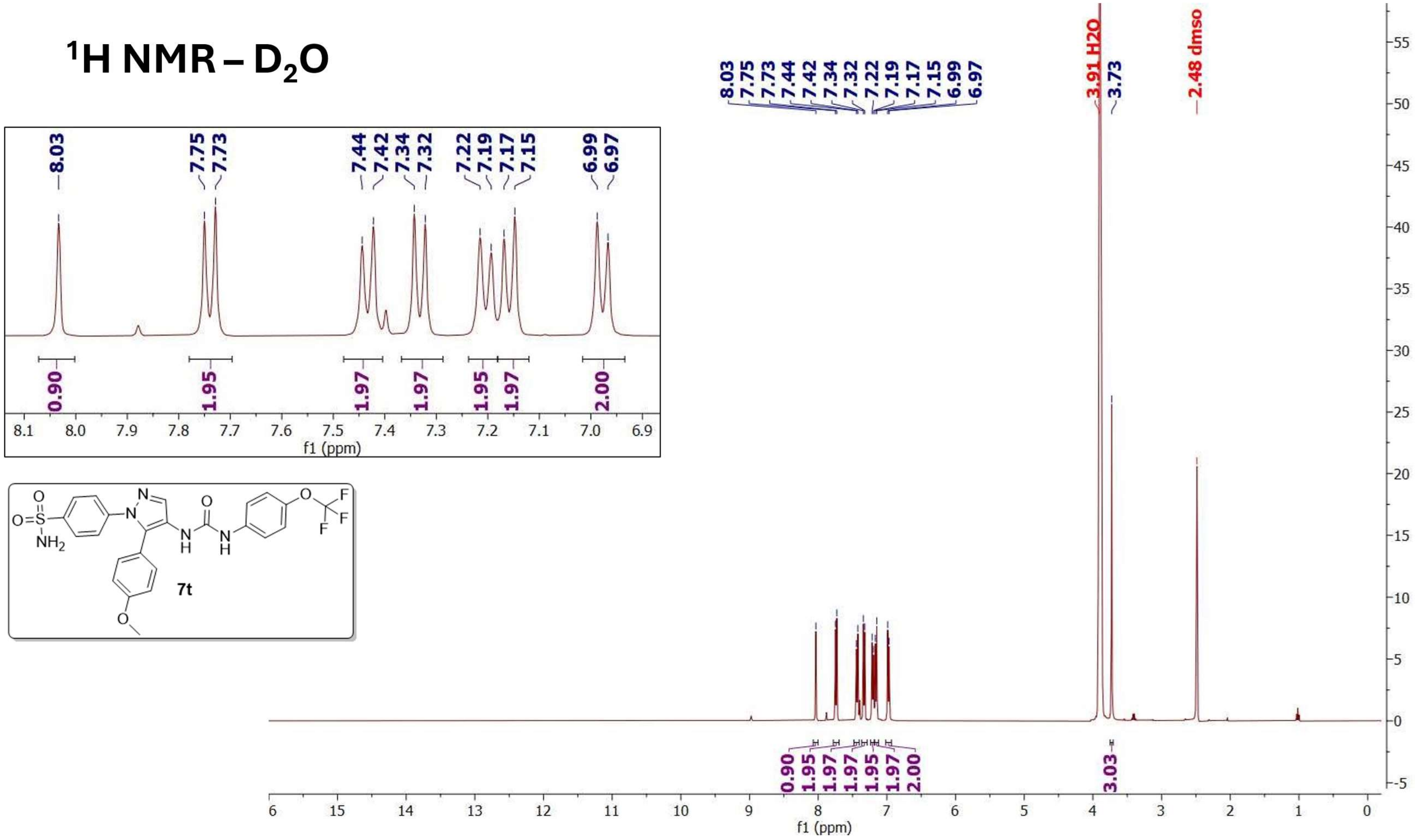

Figure S31. ^{1}H NMR spectrum of compound **7t** (D_2O).

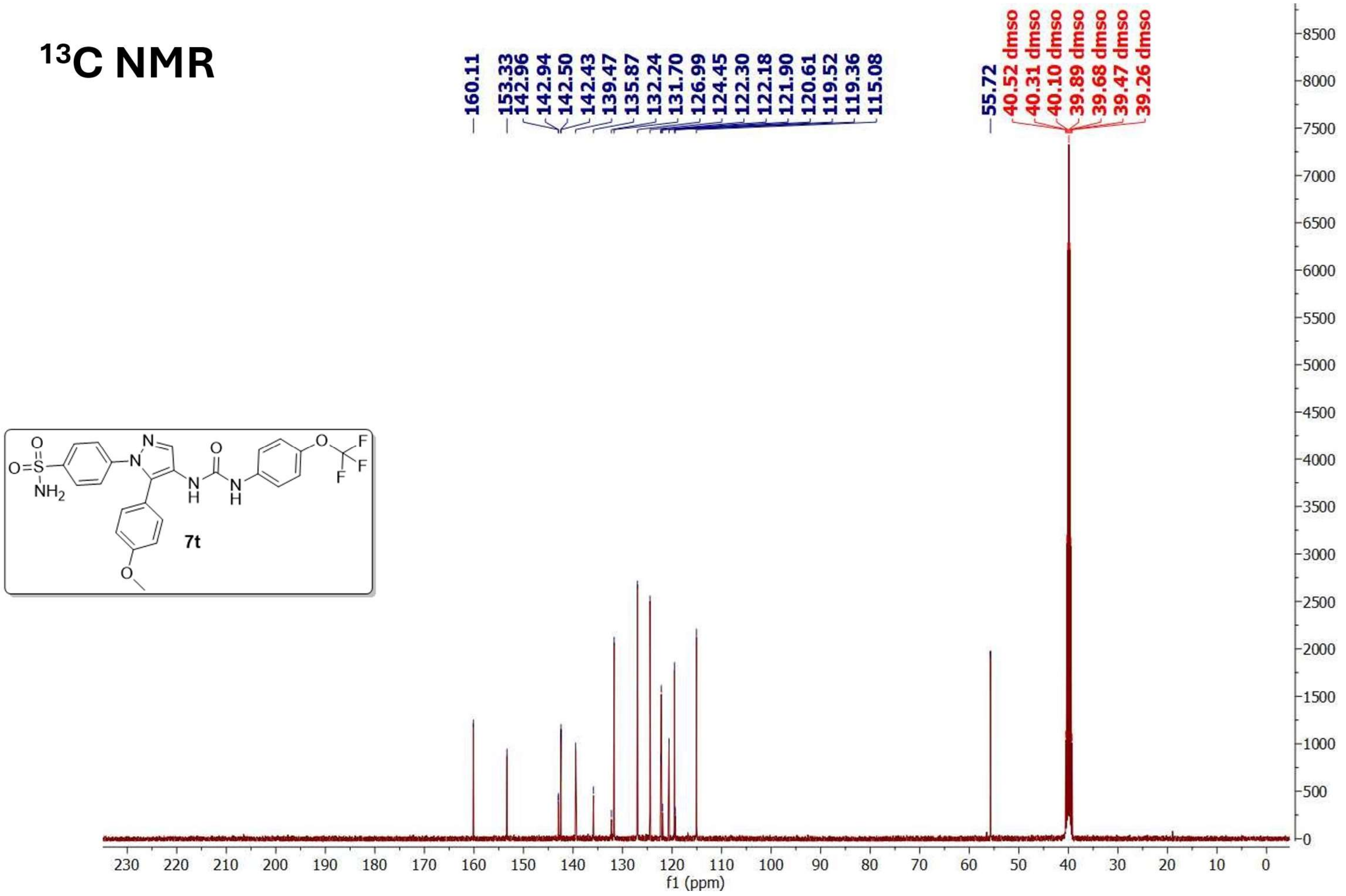

Figure S32. ^{13}C NMR spectrum of compound **7t.**

HRMS spectra of the final compounds

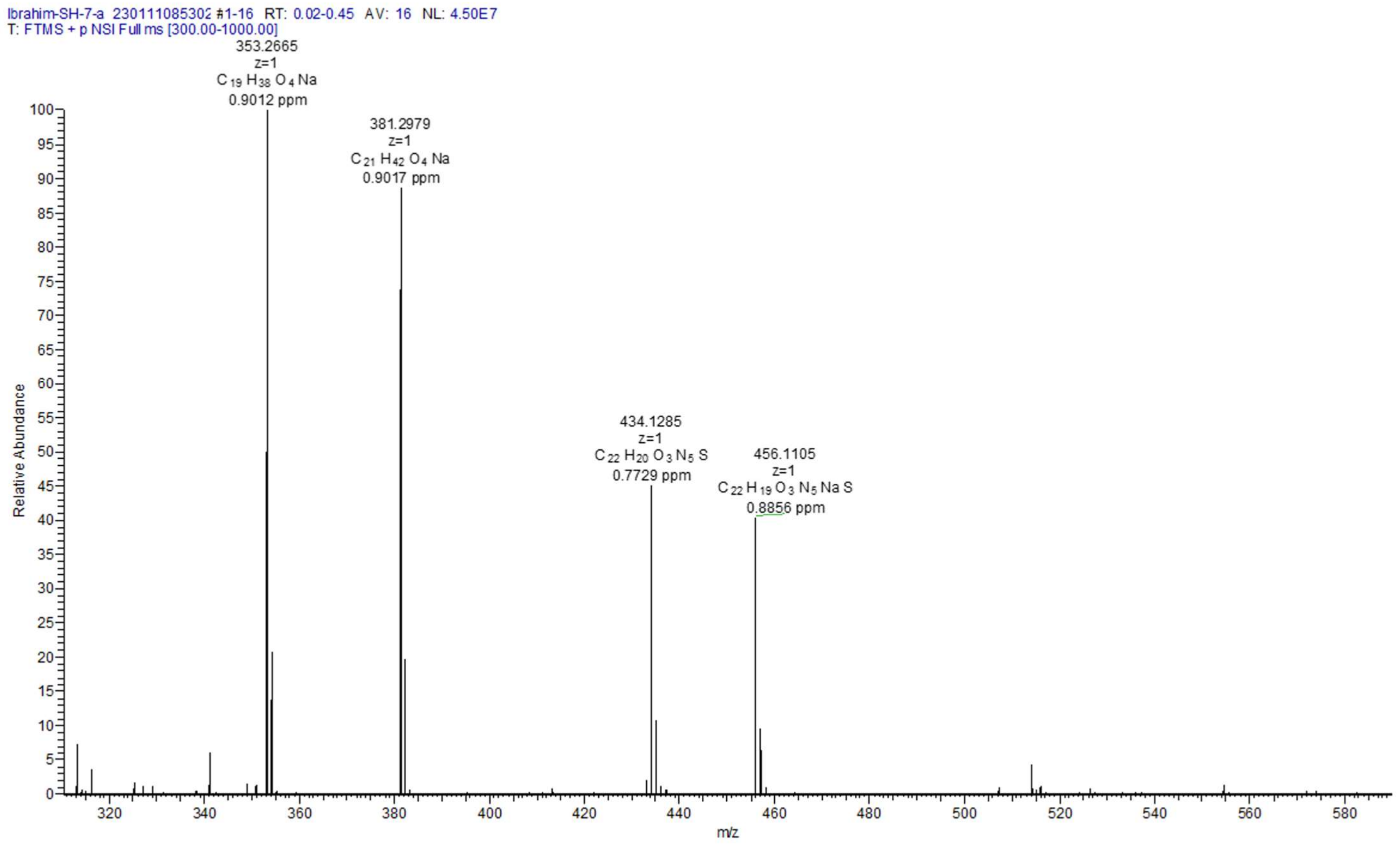

Figure S33. HRMS spectrum of compound **7a.**

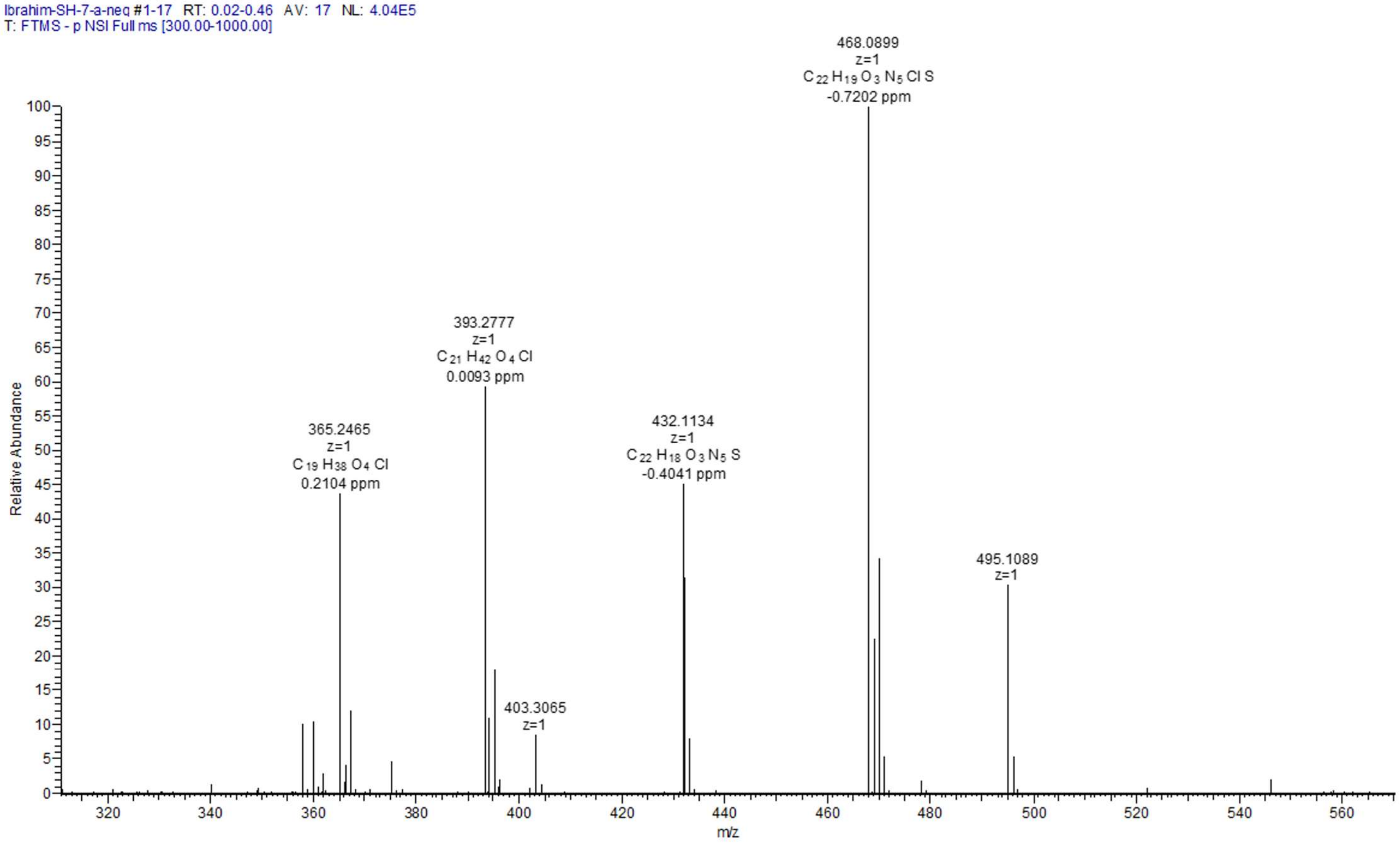

Figure S34. HRMS spectrum of compound **7a.**

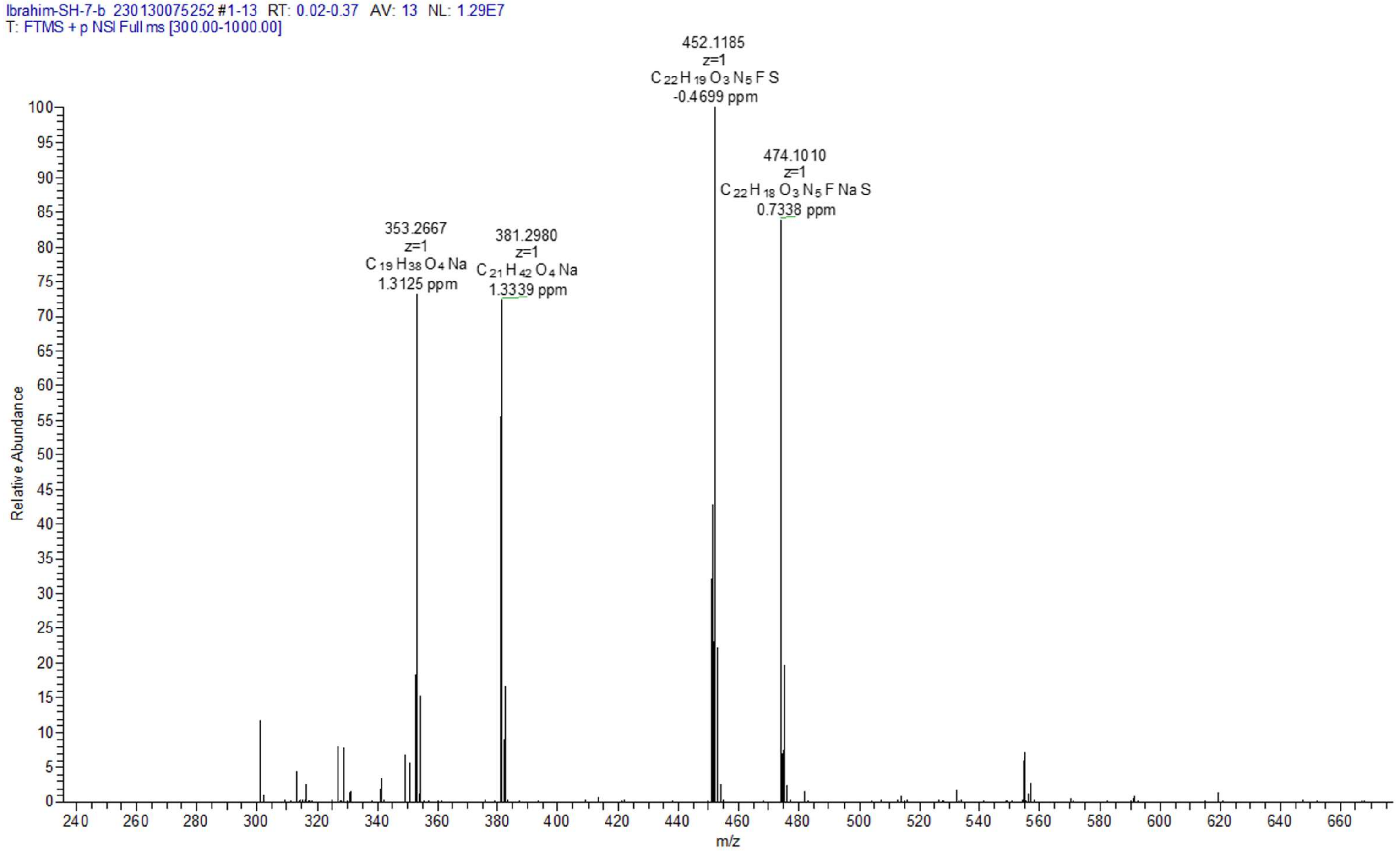

Figure S35. HRMS spectrum of compound **7b.**

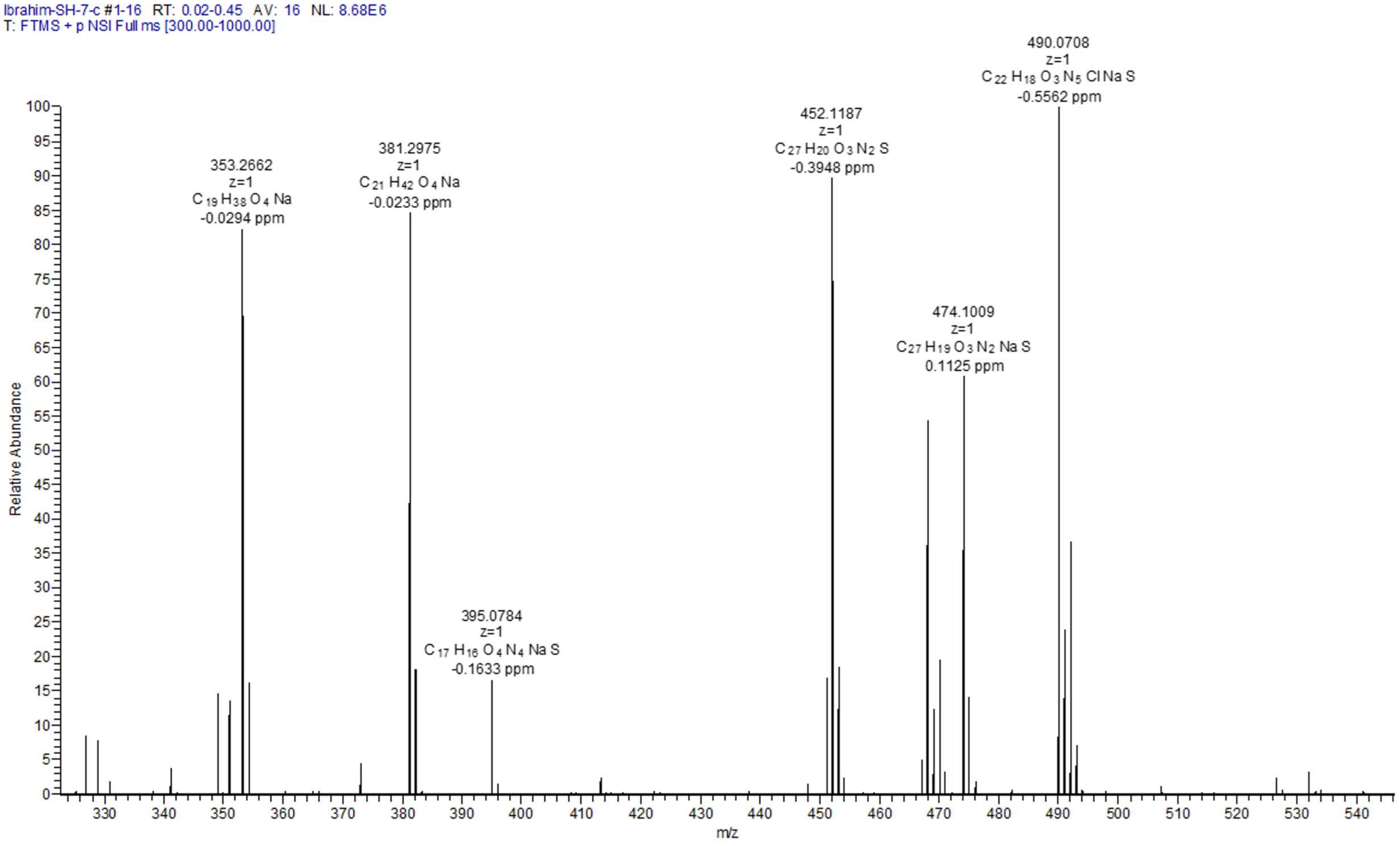

Figure S36. HRMS spectrum of compound **7c.**

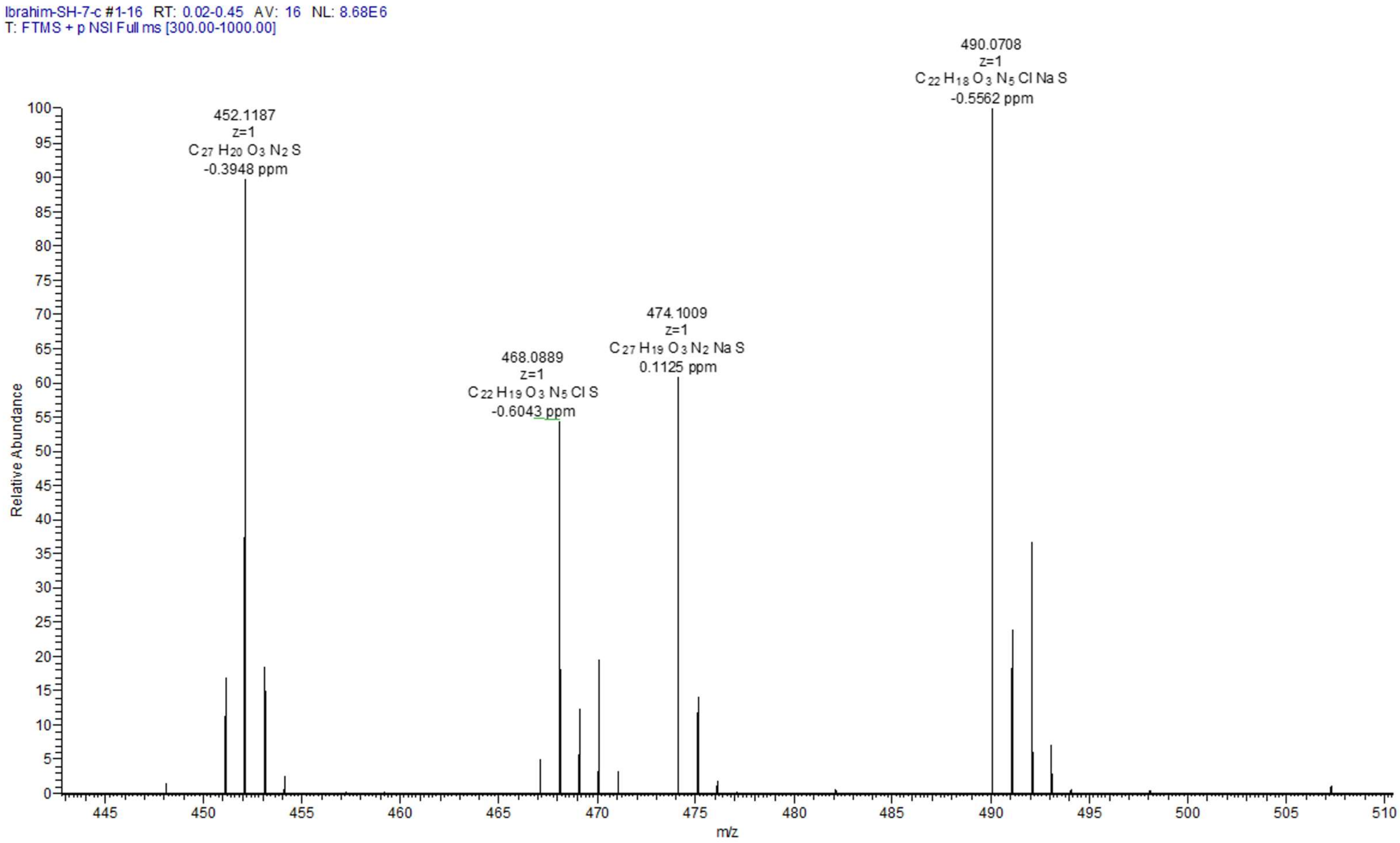

Figure S37. HRMS spectrum of compound **7c.**

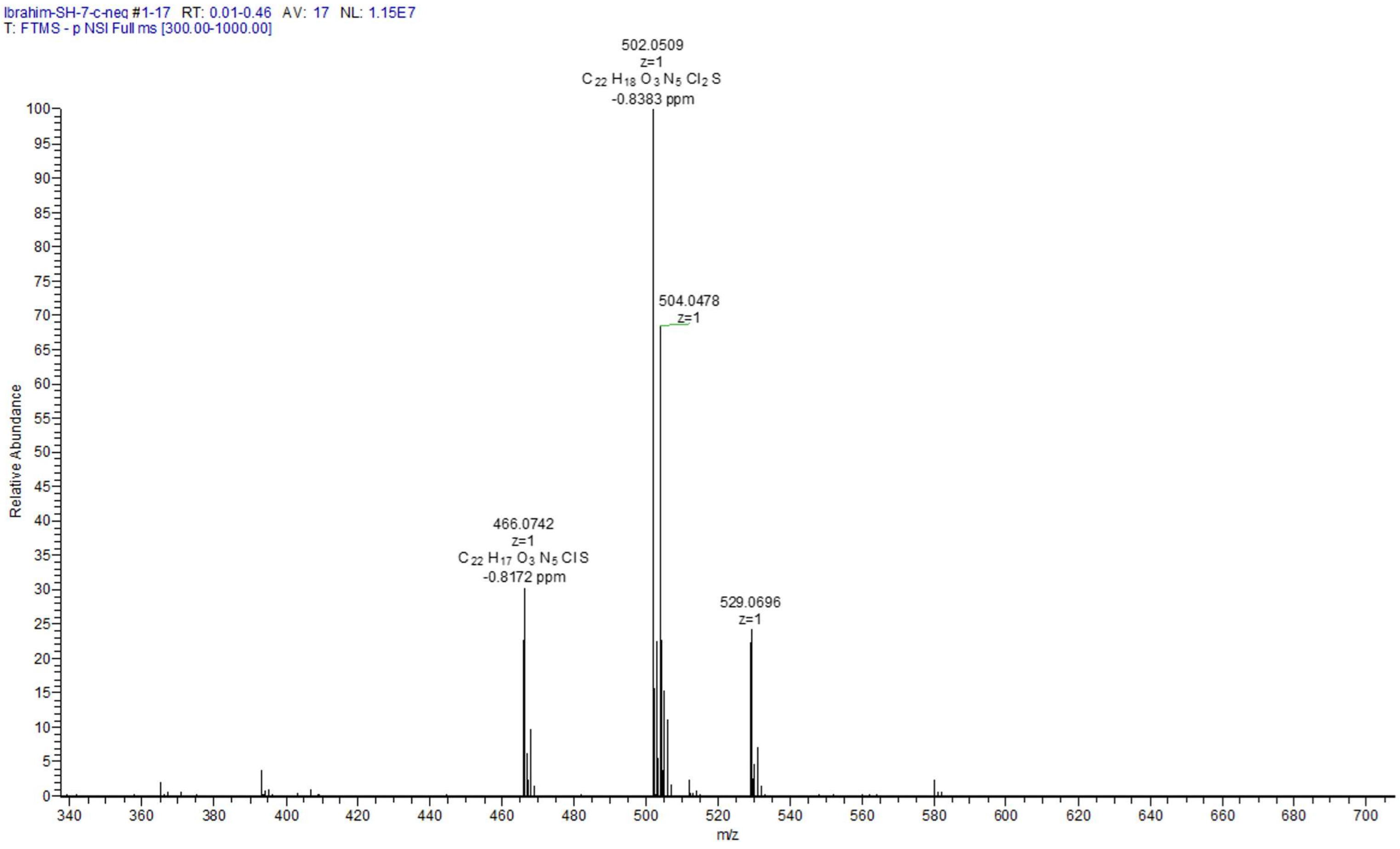

Figure S38. HRMS spectrum of compound **7c.**

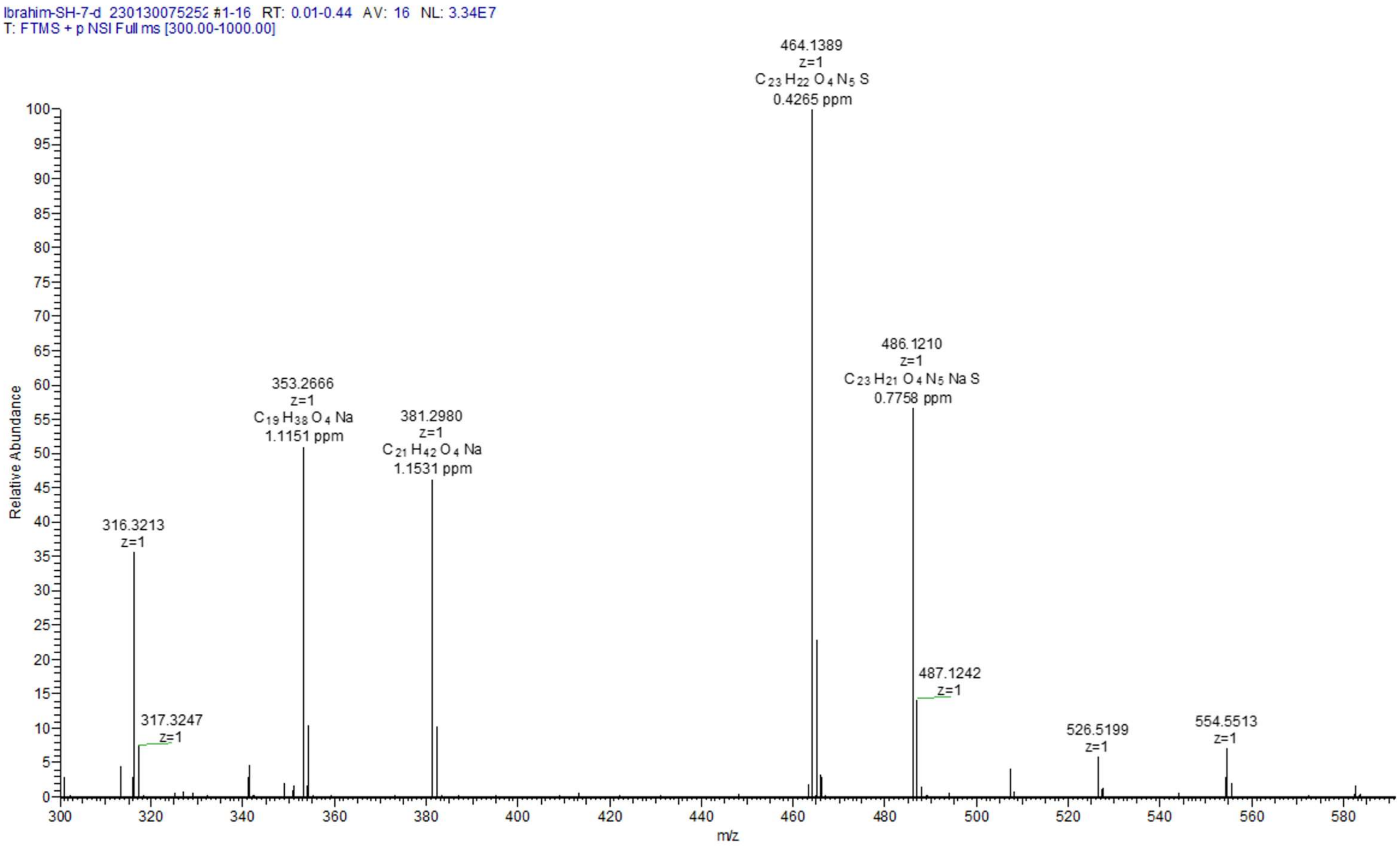

Figure S39. HRMS spectrum of compound **7d.**

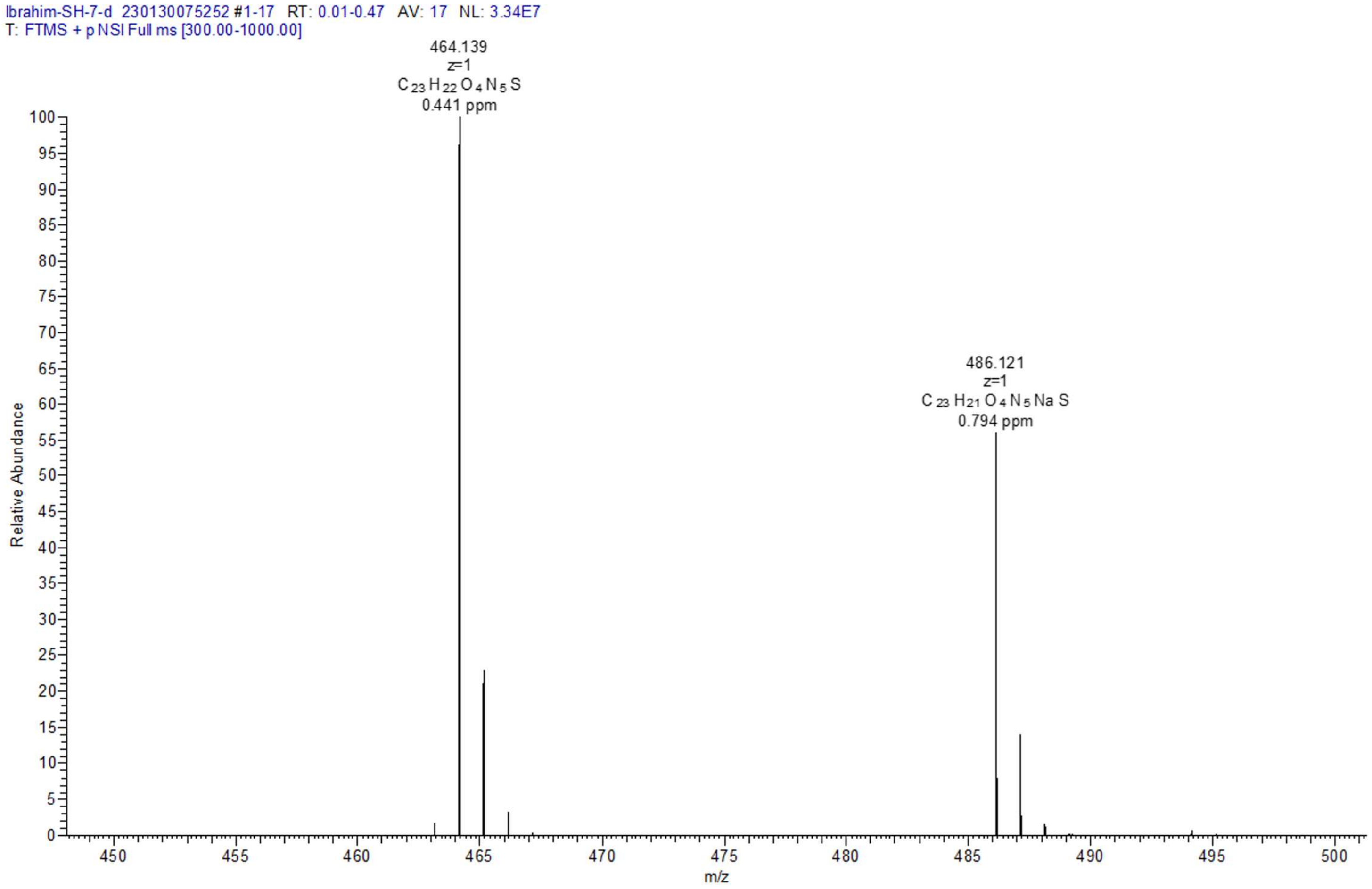

Figure S40. HRMS spectrum of compound **7d.**

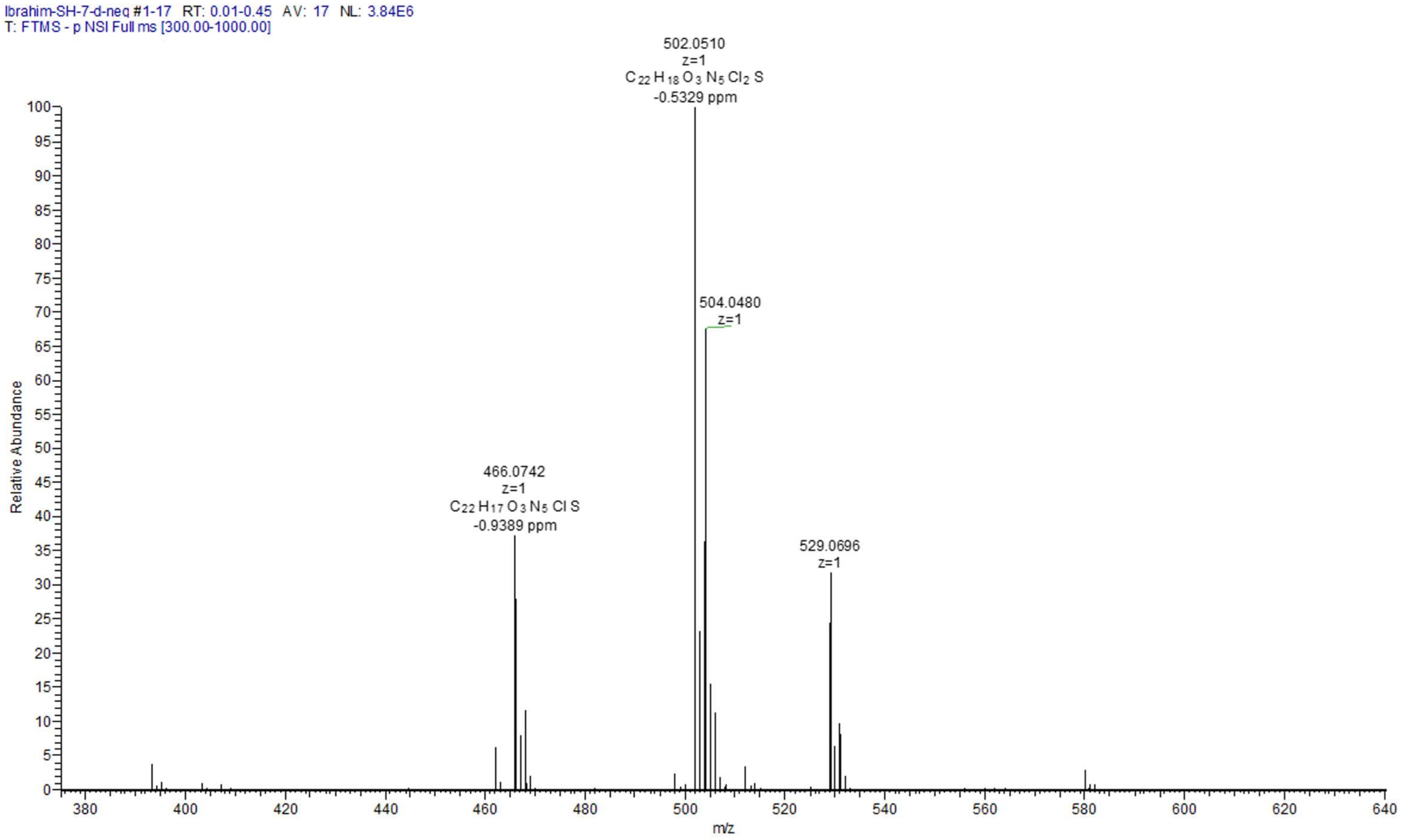

Figure S41. HRMS spectrum of compound **7d.**

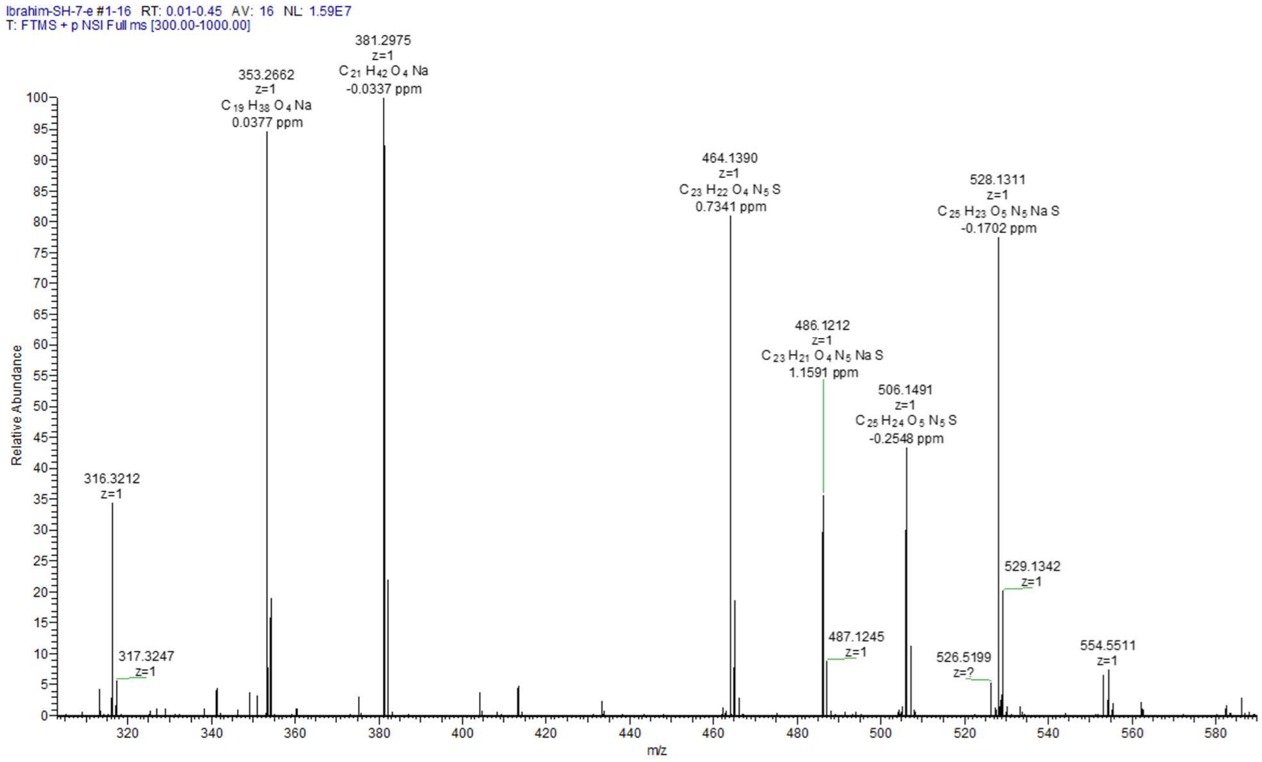

Figure S42. HRMS spectrum of compound **7e**.

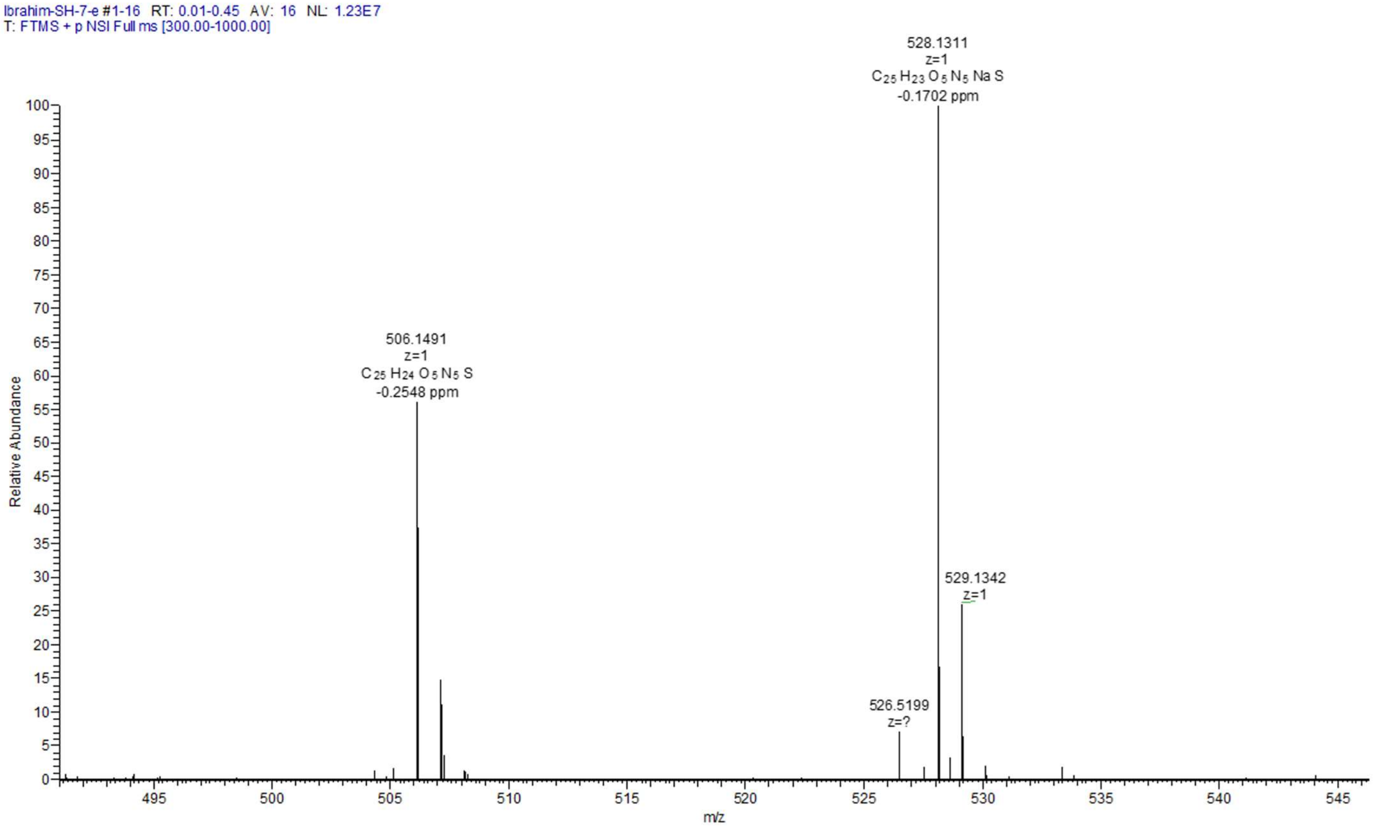

Figure S43. HRMS spectrum of compound **7e.**

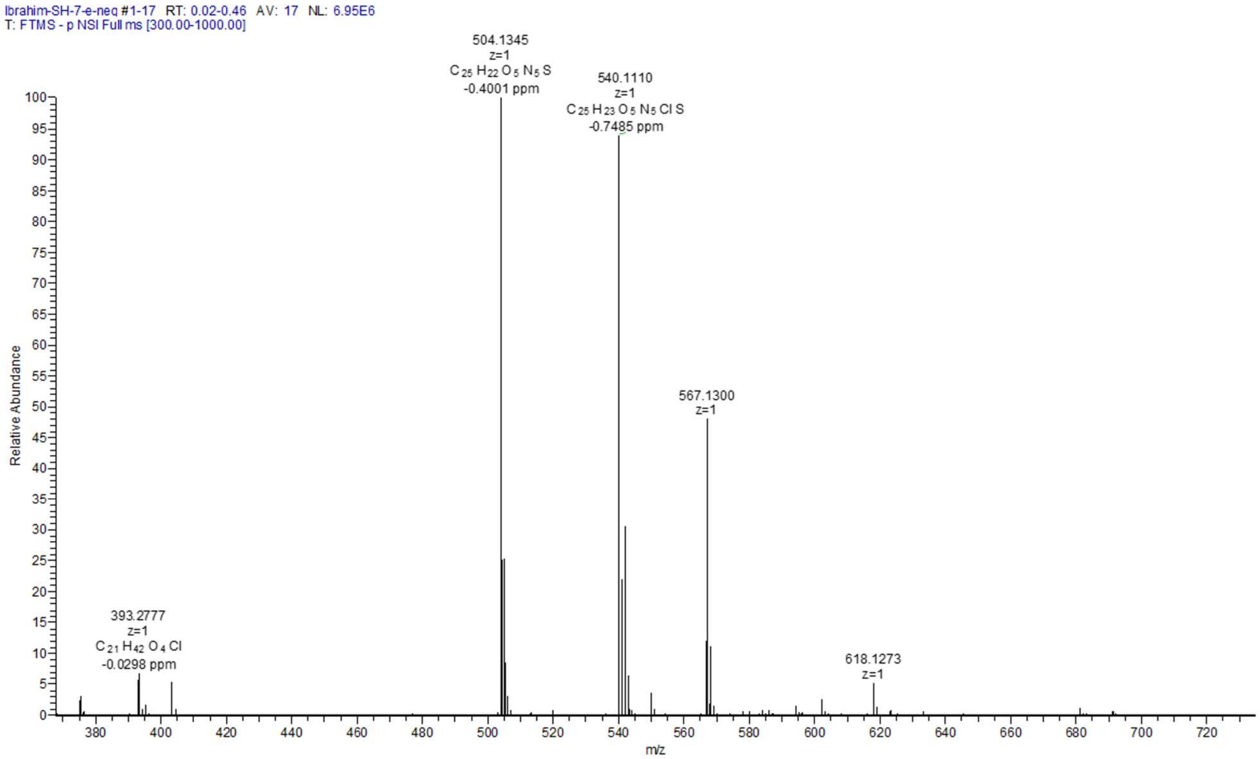

Figure S44. HRMS spectrum of compound **7e.**

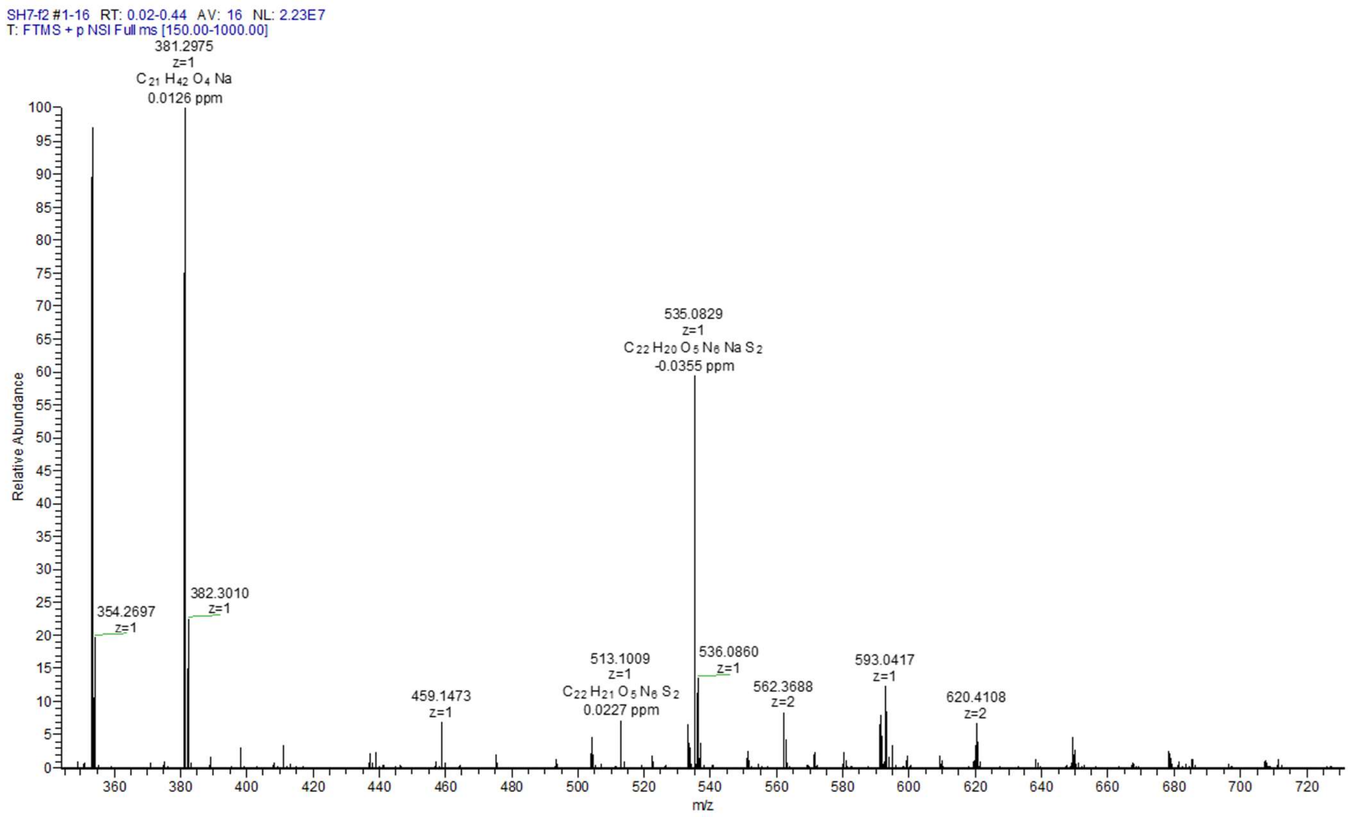

Figure S45. HRMS spectrum of compound **7f.**

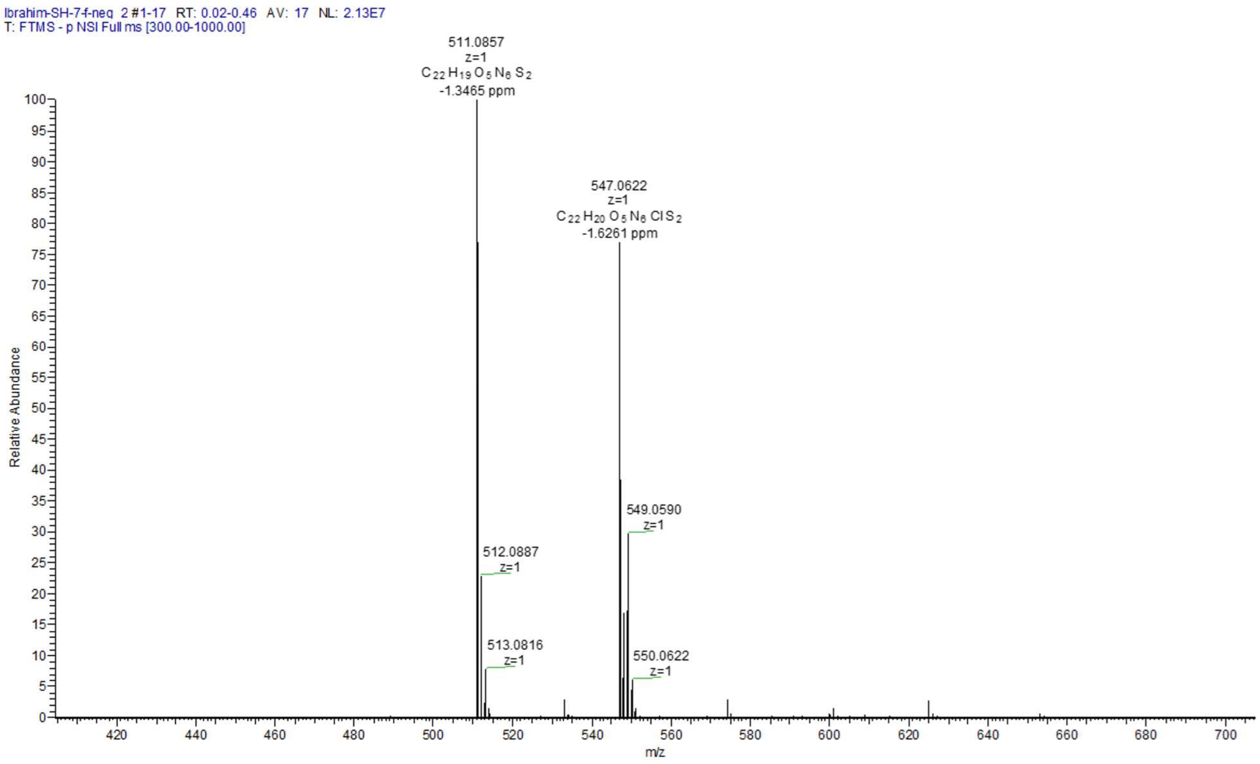

Figure S46. HRMS spectrum of compound **7f.**

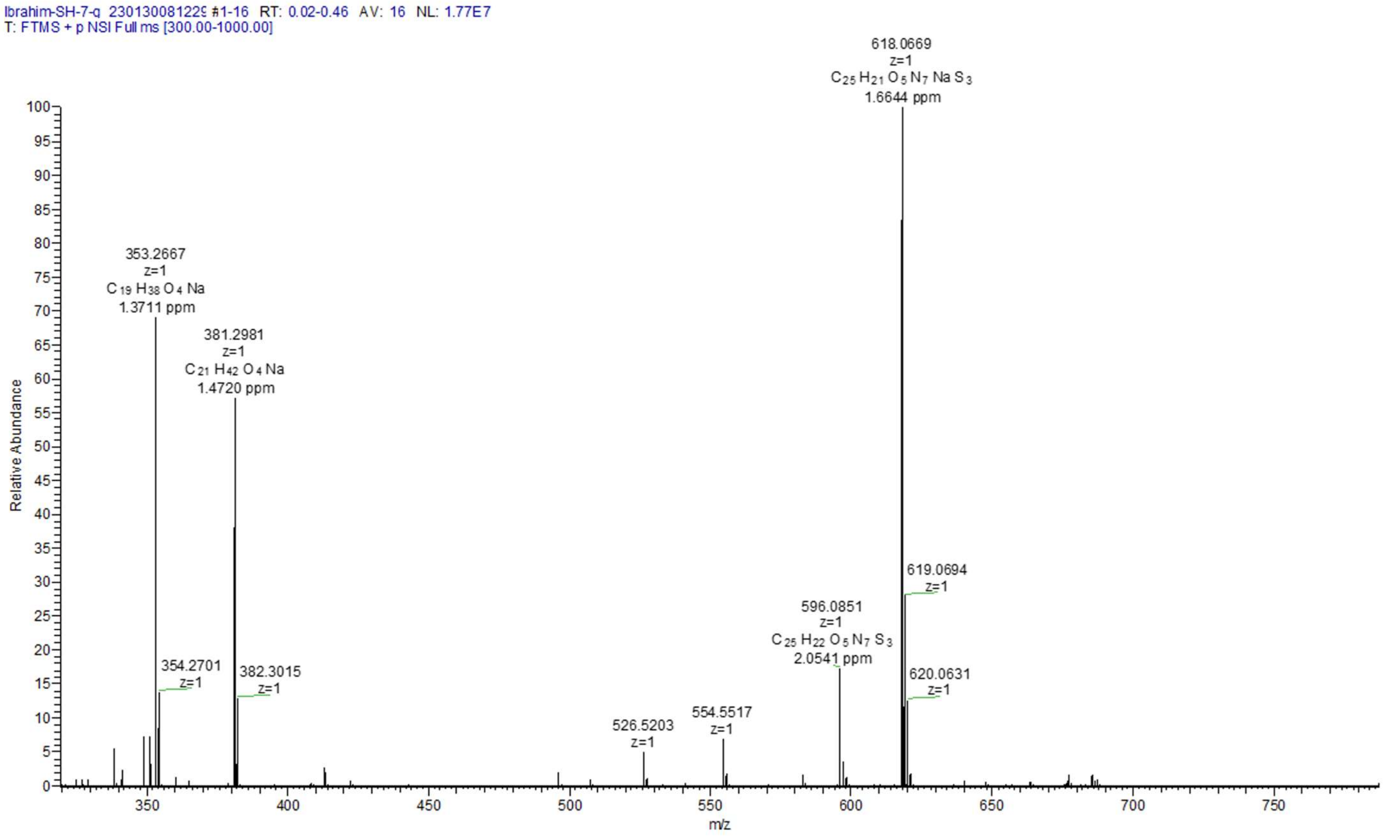

Figure S47. HRMS spectrum of compound **7g.**

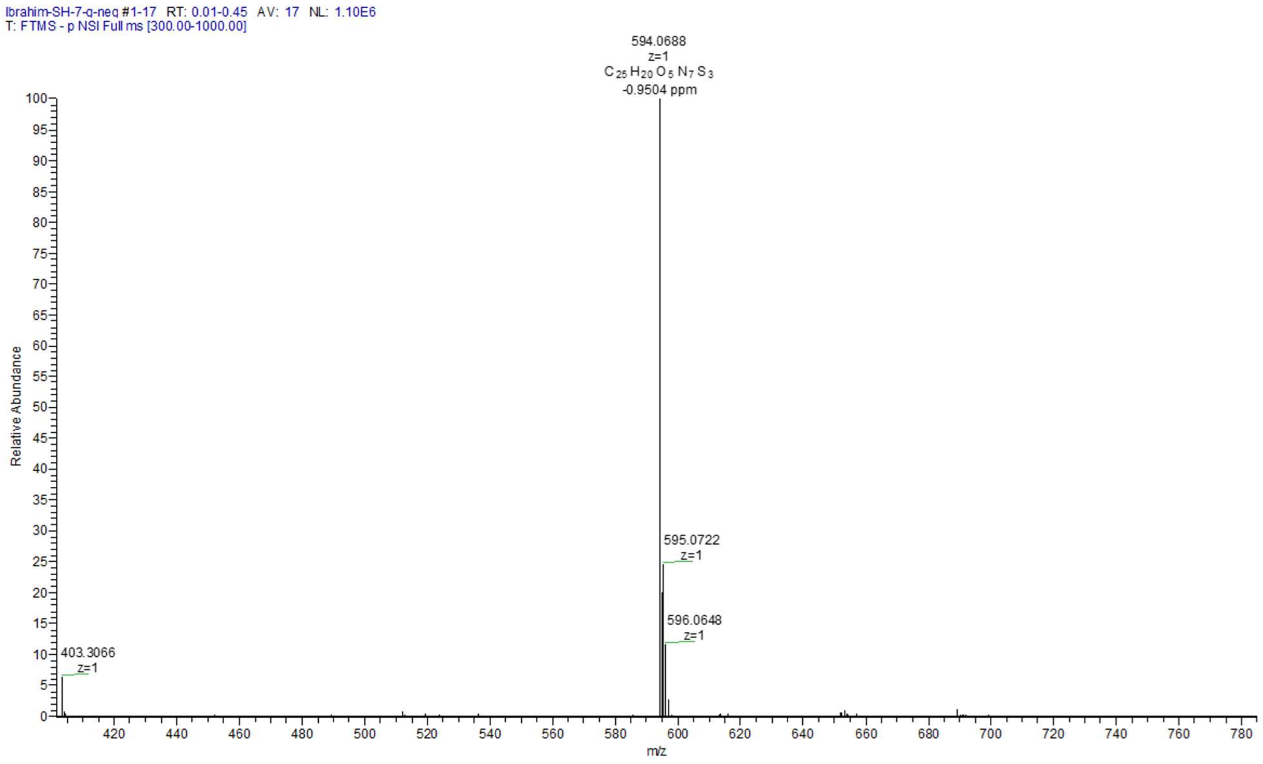

Figure S48. HRMS spectrum of compound **7g.**

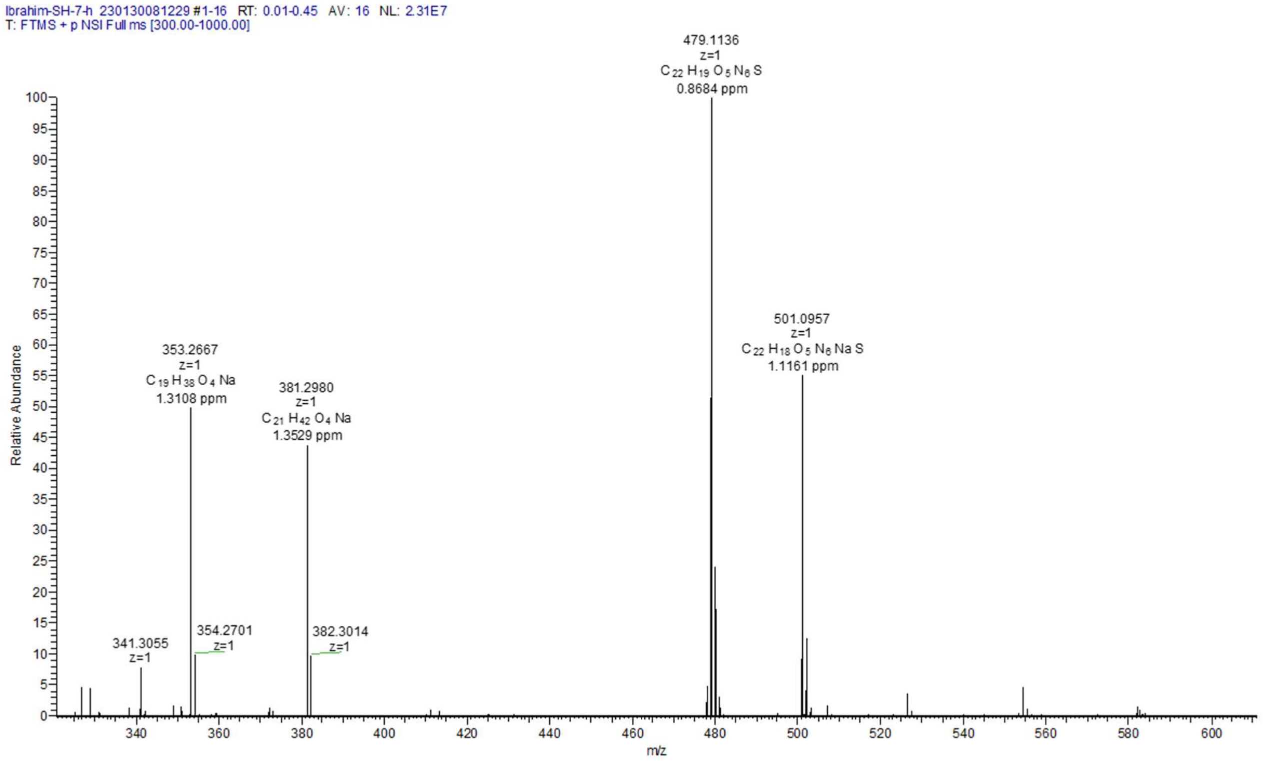

Figure S49. HRMS spectrum of compound **7h.**

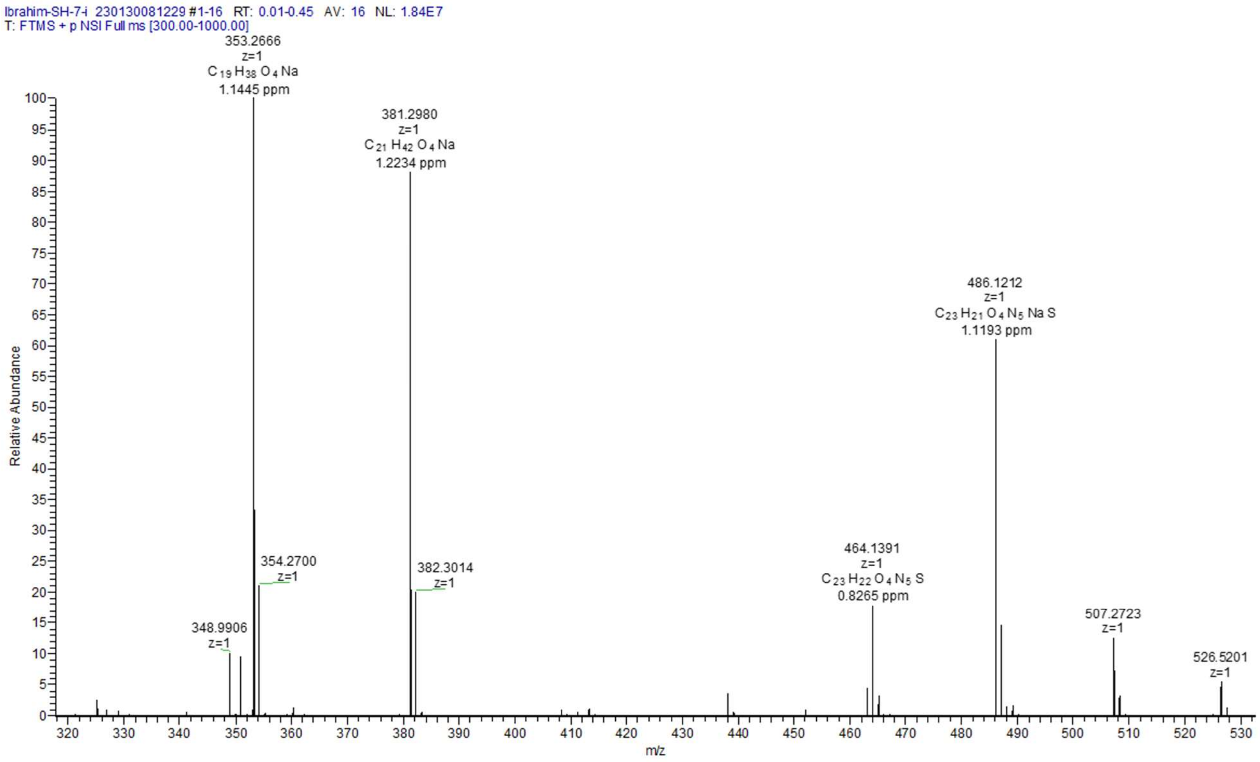

Figure S50. HRMS spectrum of compound **7i.**

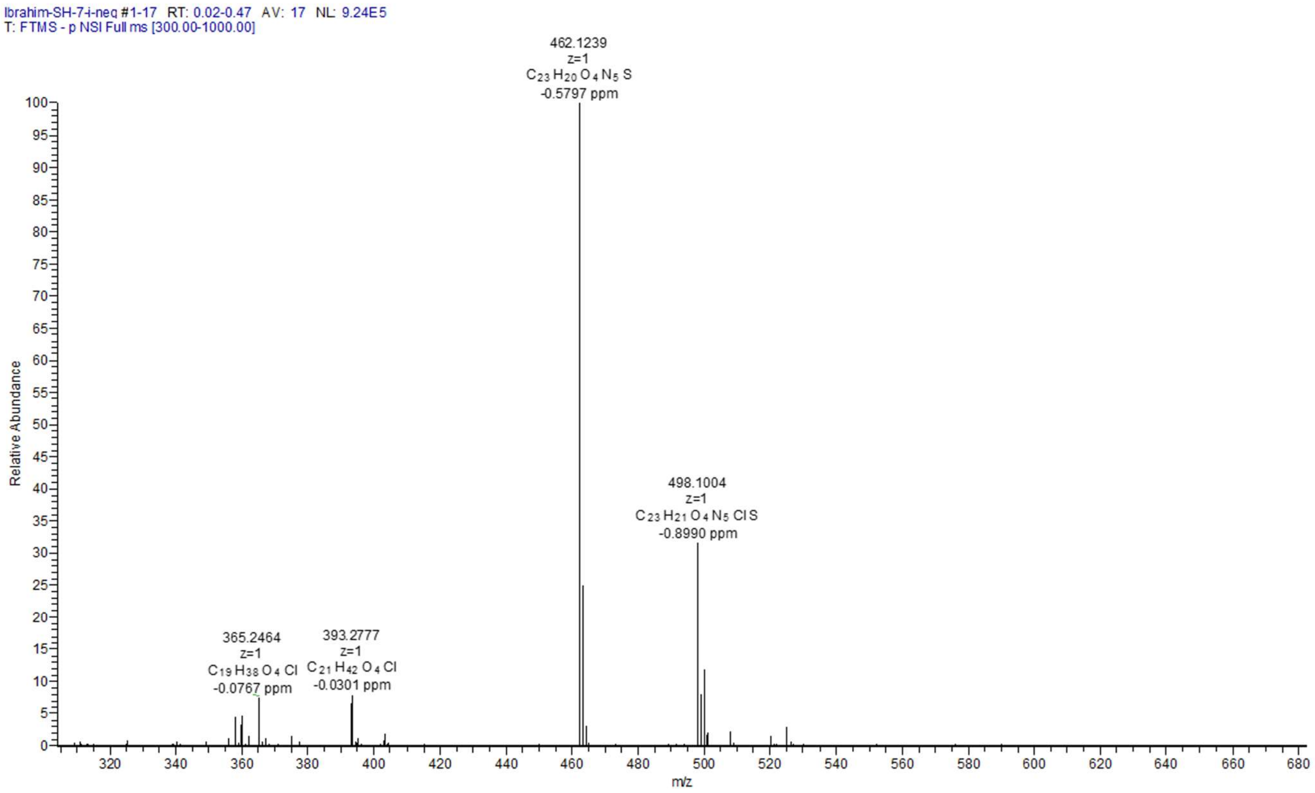

Figure S51. HRMS spectrum of compound **7i.**

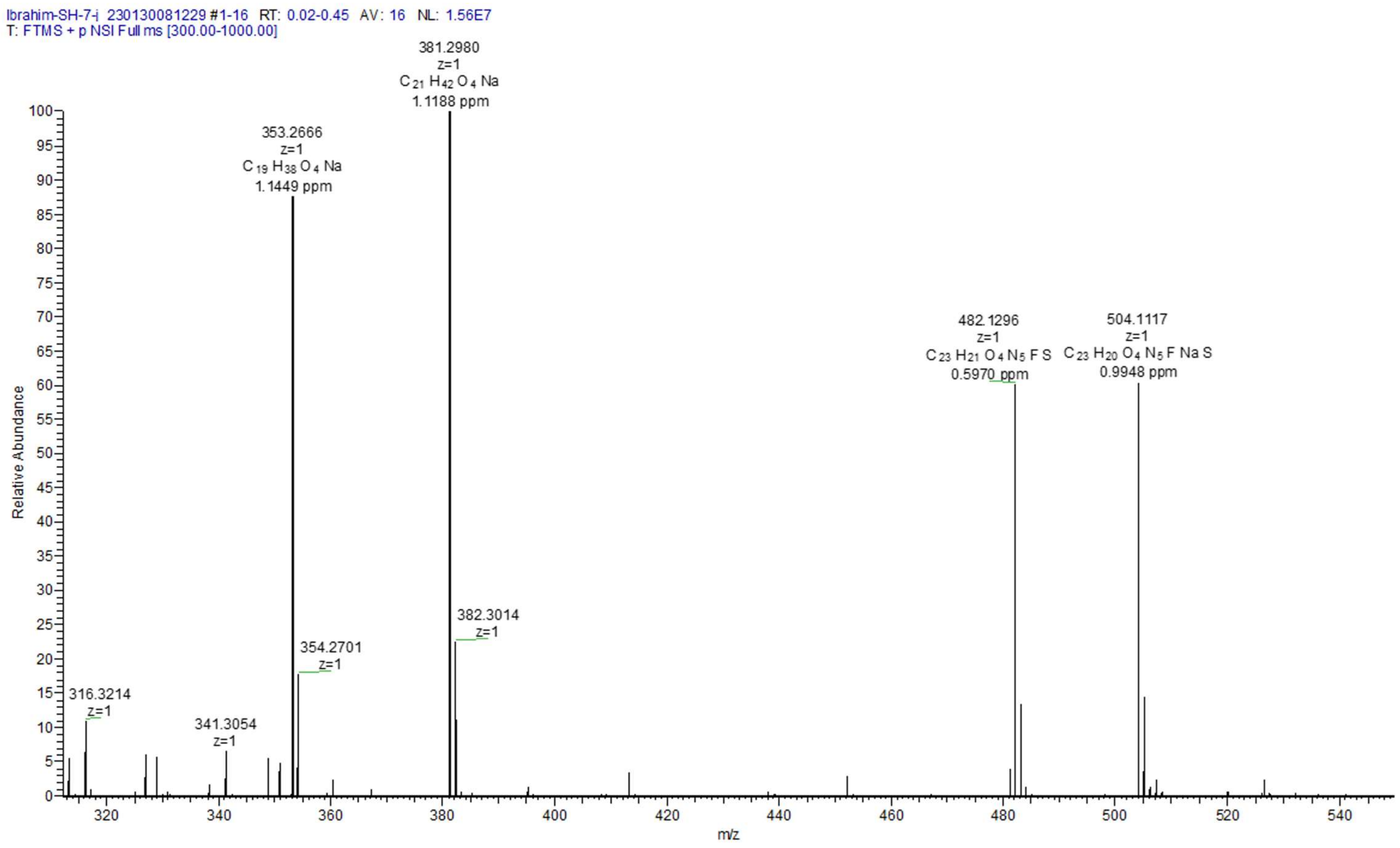

Figure S52. HRMS spectrum of compound **7i.**

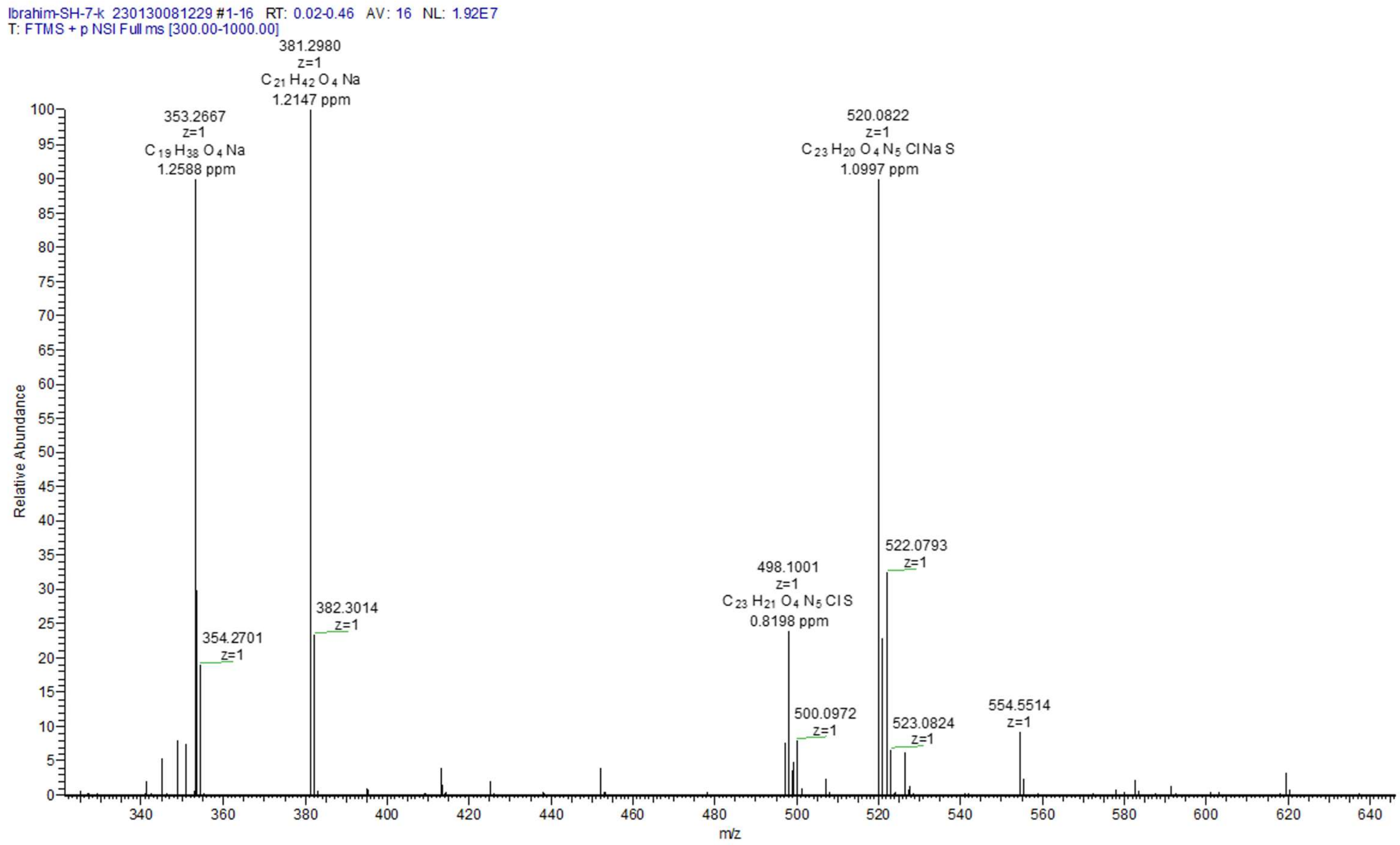

Figure S53. HRMS spectrum of compound **7k.**

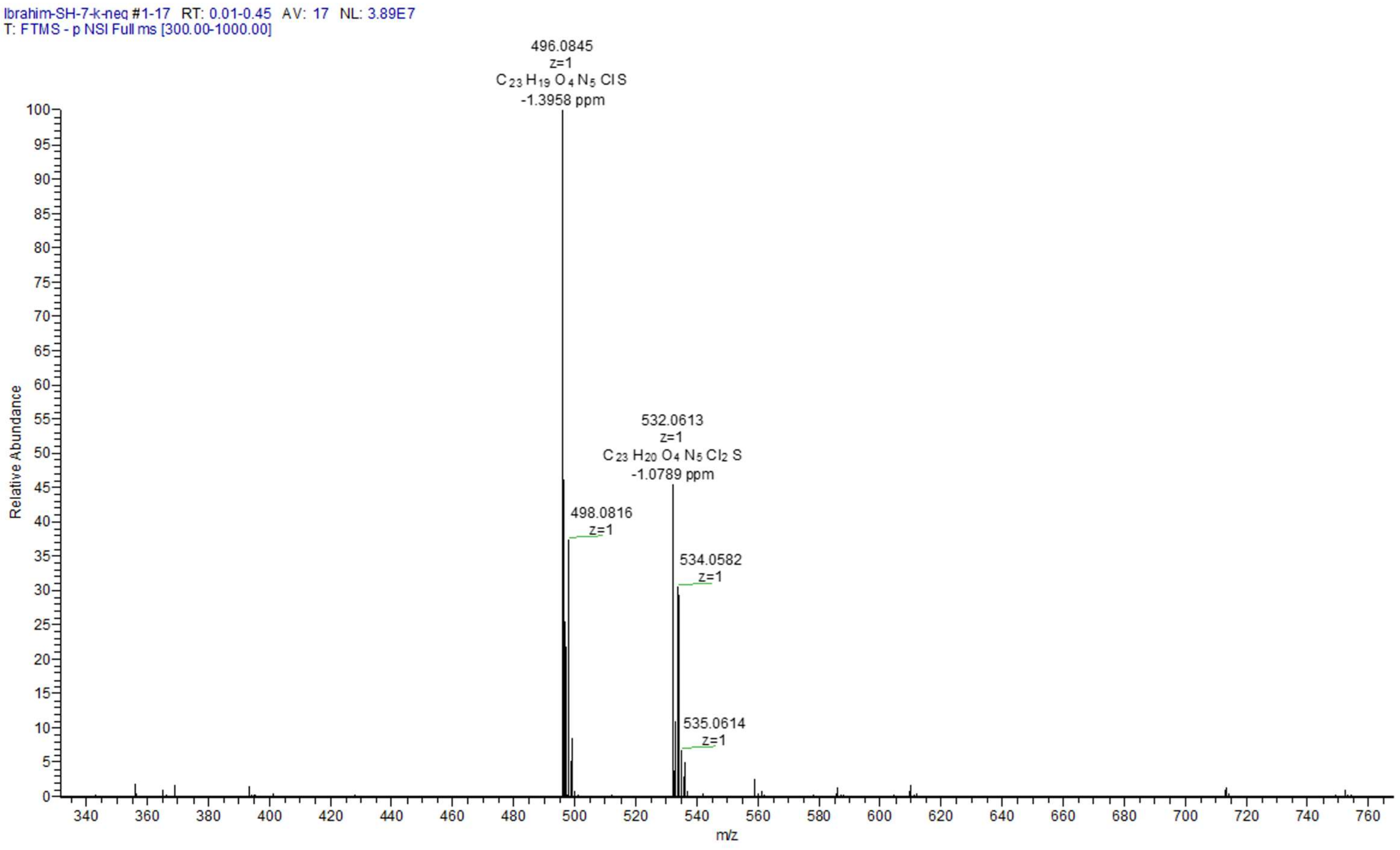

Figure S54. HRMS spectrum of compound **7k.**

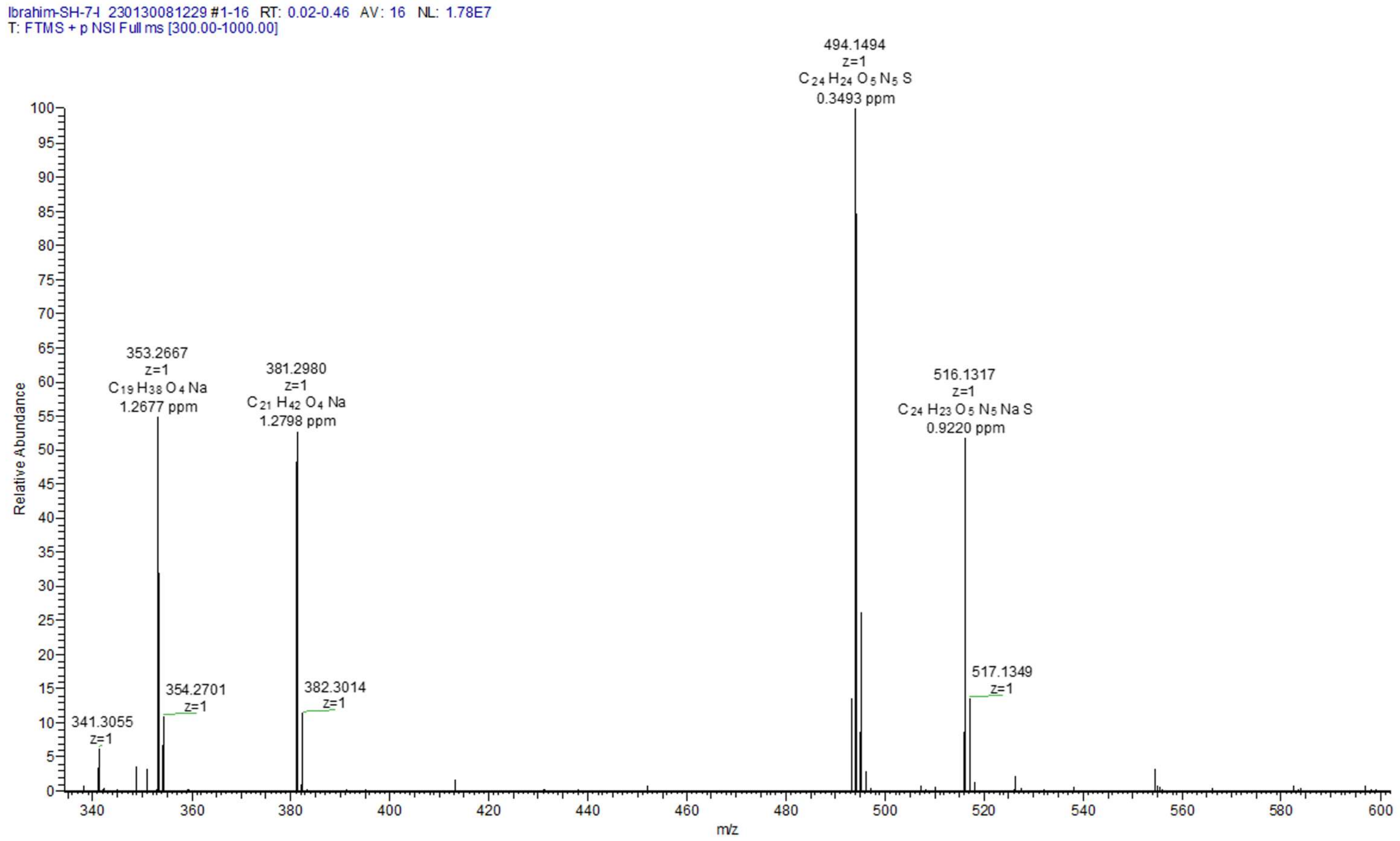

Figure S55. HRMS spectrum of compound **7l.**

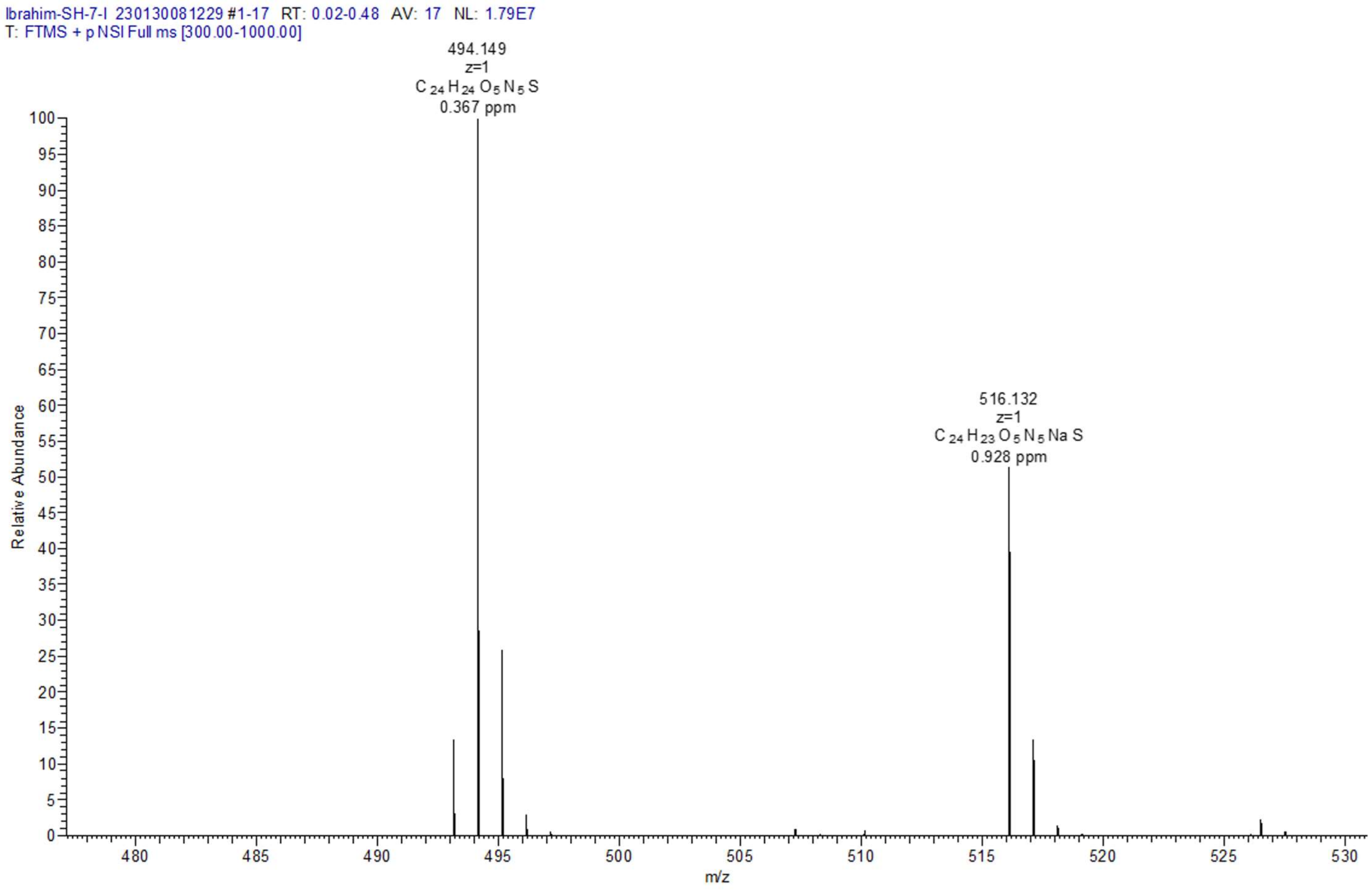

Figure S56. HRMS spectrum of compound **7l.**

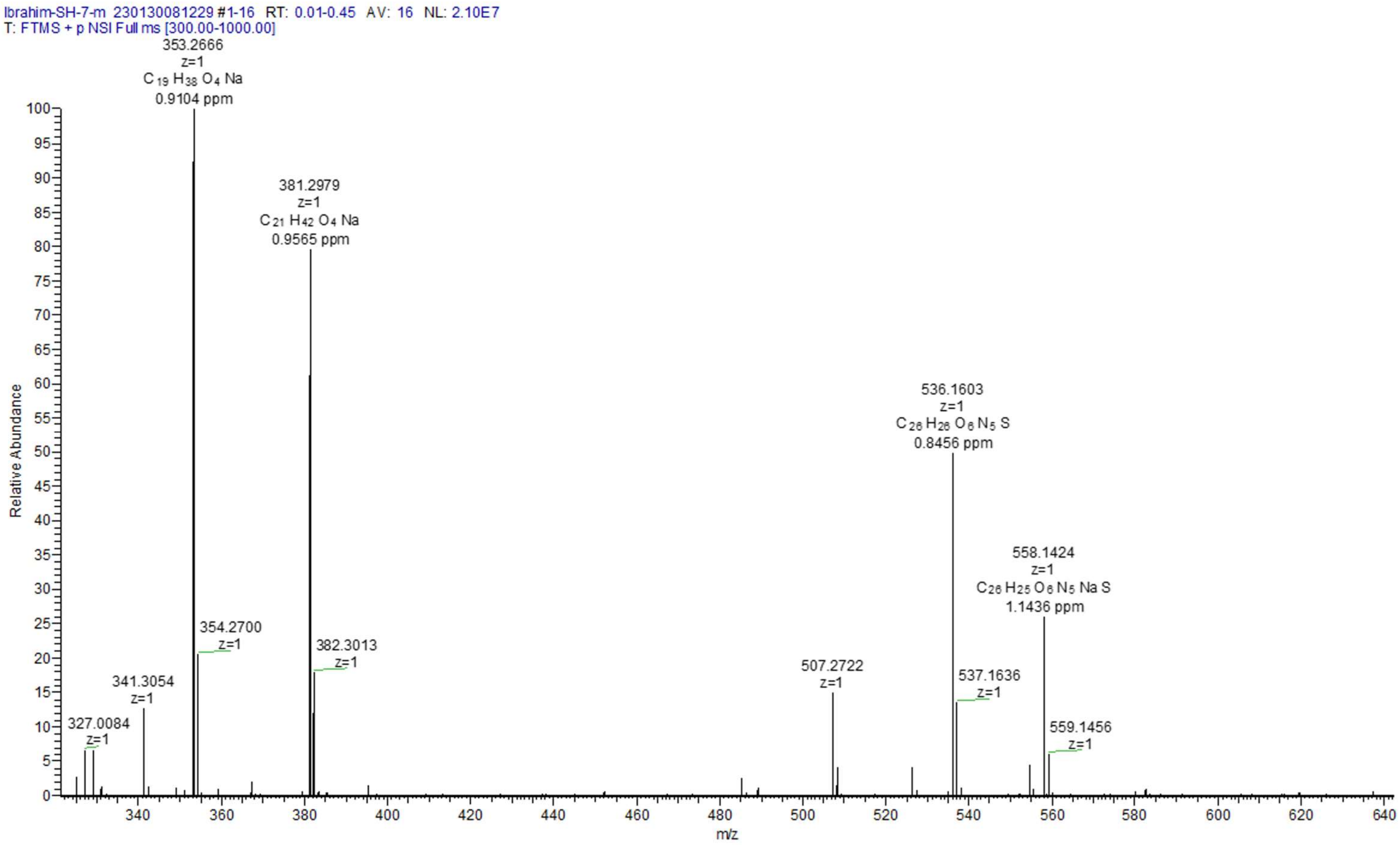

Figure S57. HRMS spectrum of compound **7m.**

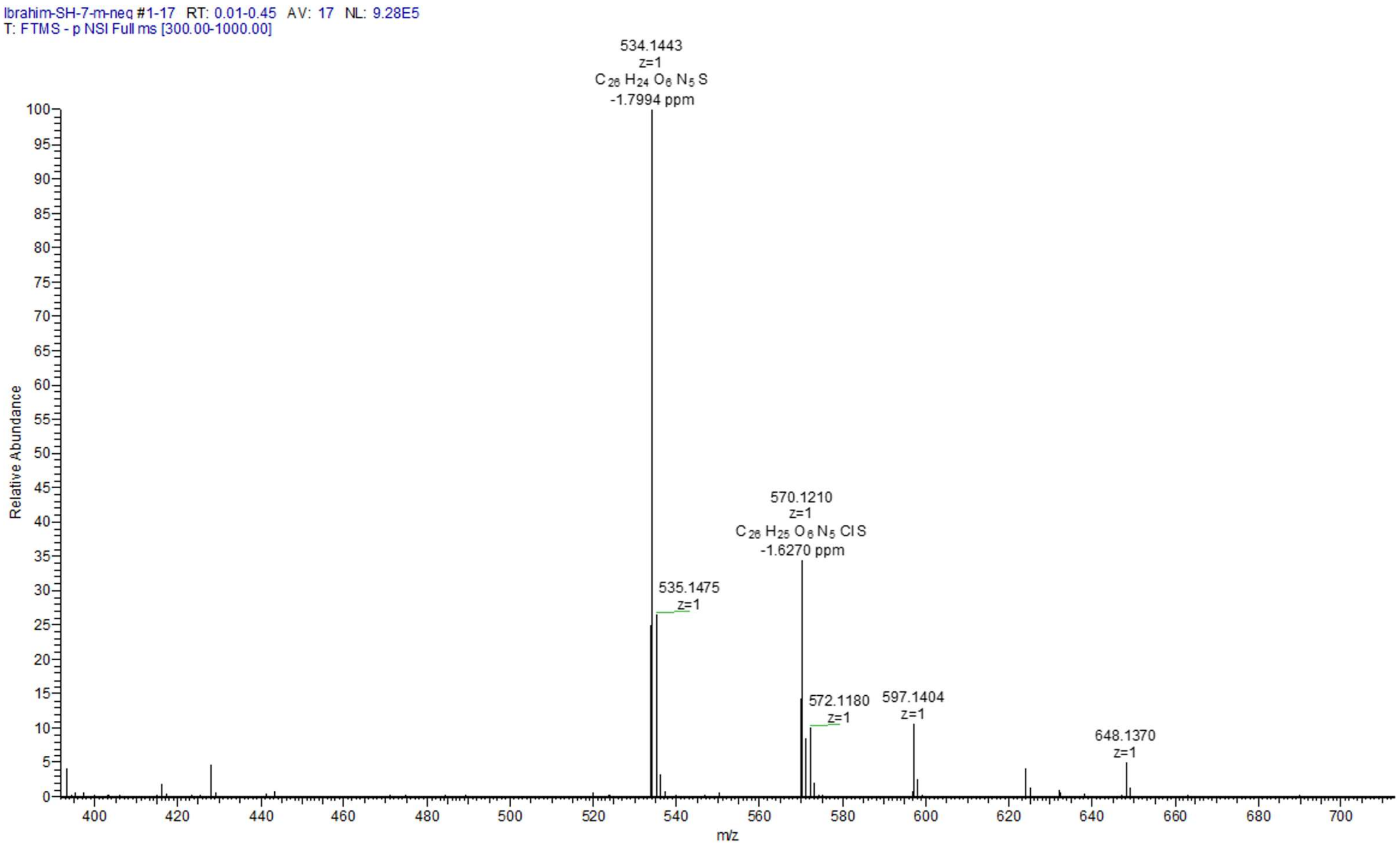

Figure S58. HRMS spectrum of compound **7m.**

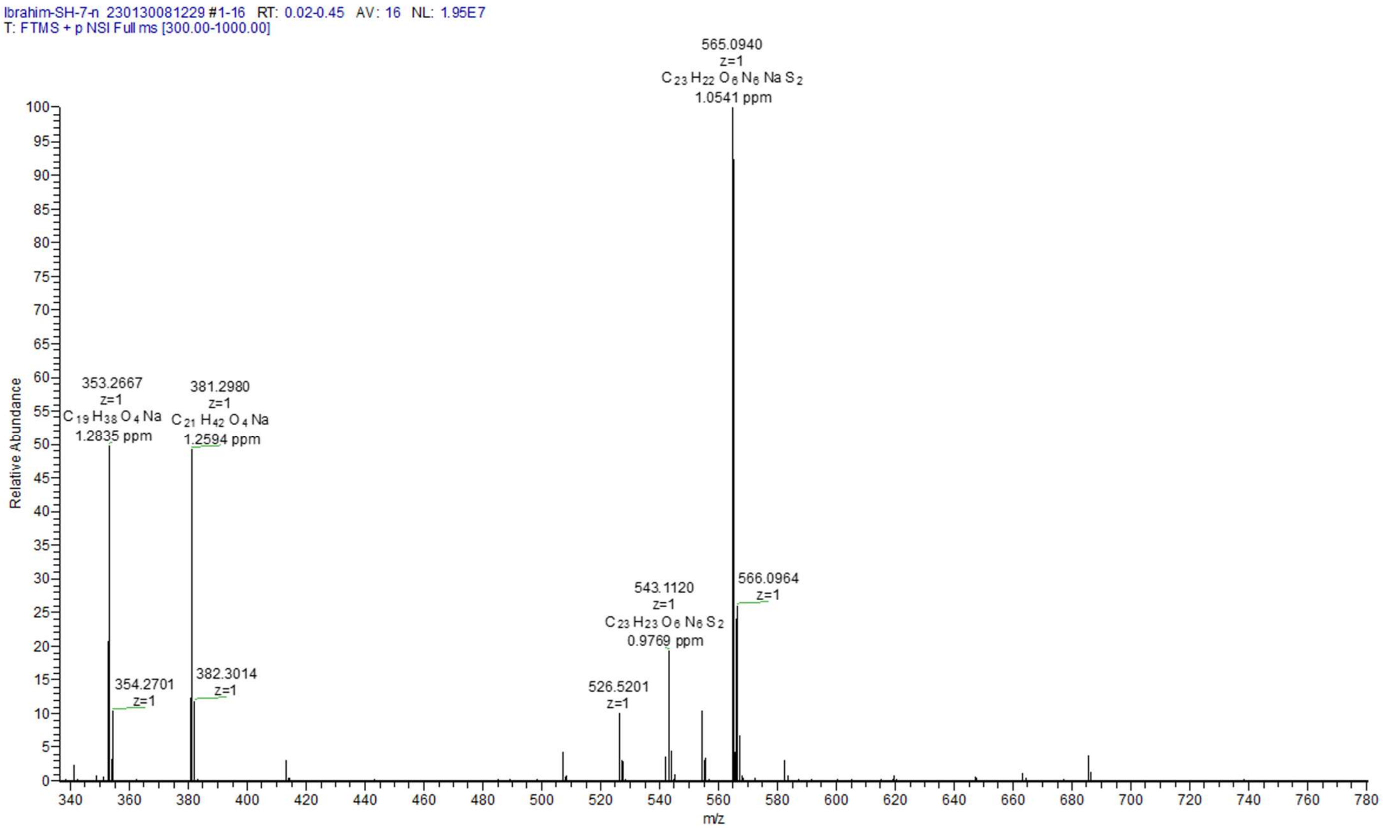

Figure S59. HRMS spectrum of compound **7n.**

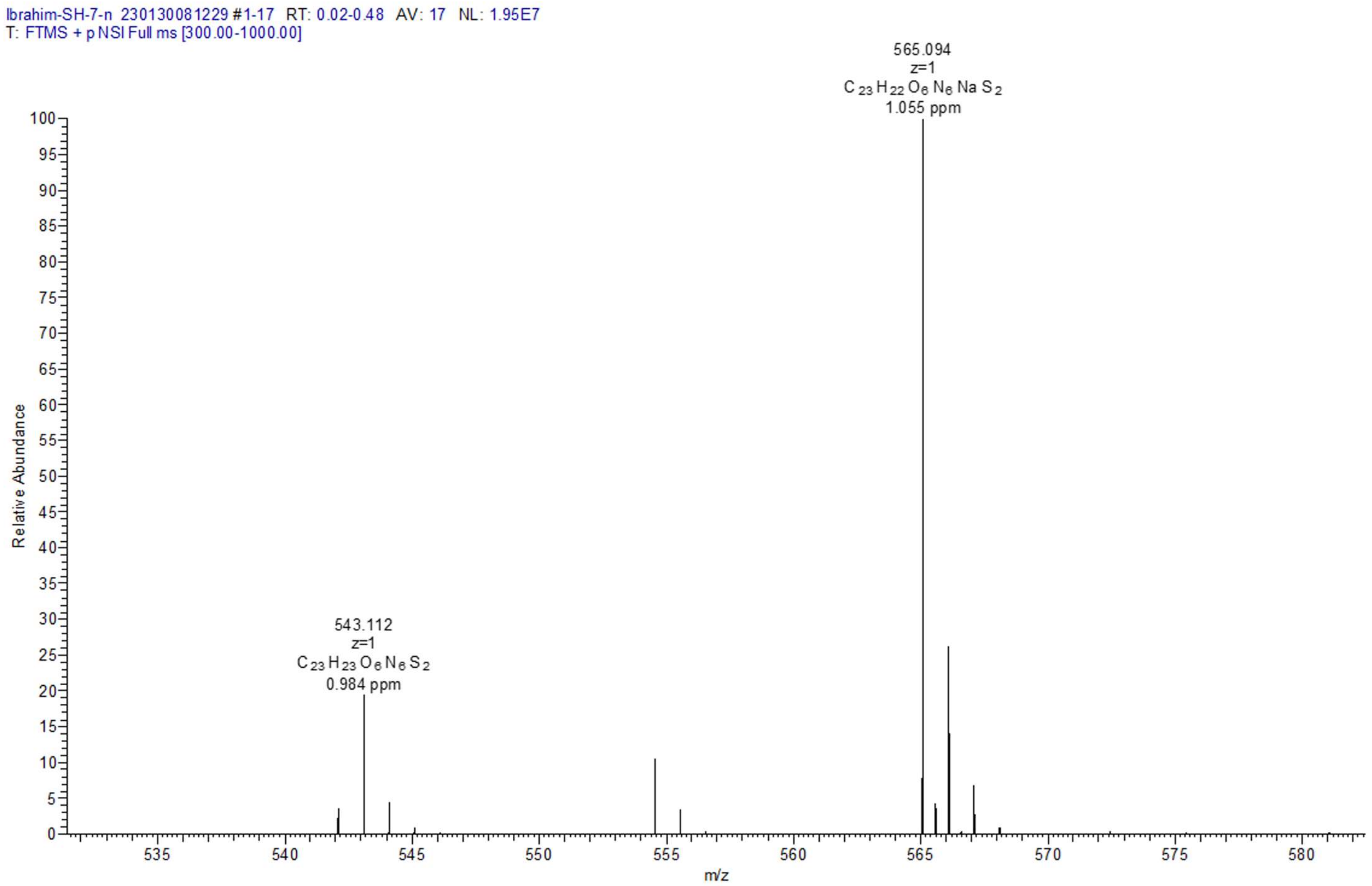

Figure S60. HRMS spectrum of compound **7n.**

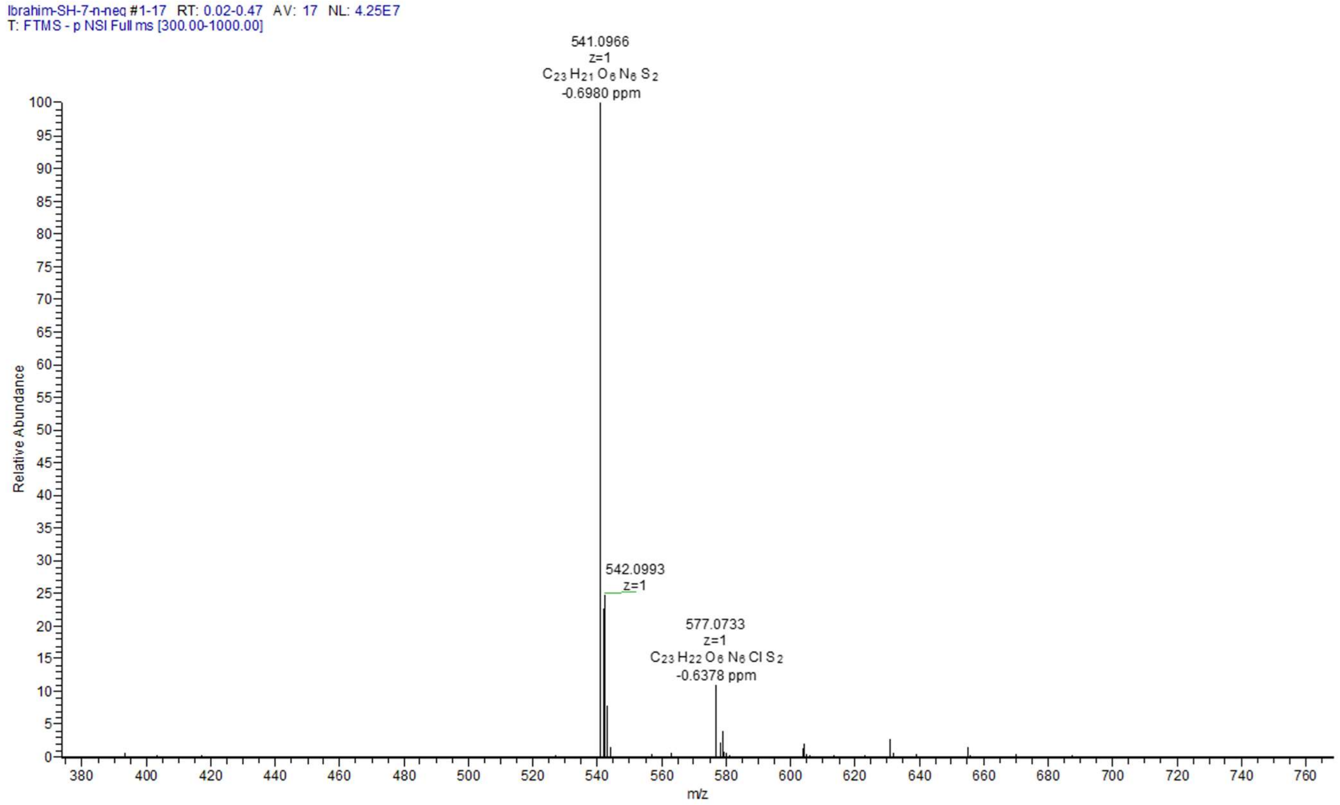

Figure S61. HRMS spectrum of compound **7n.**

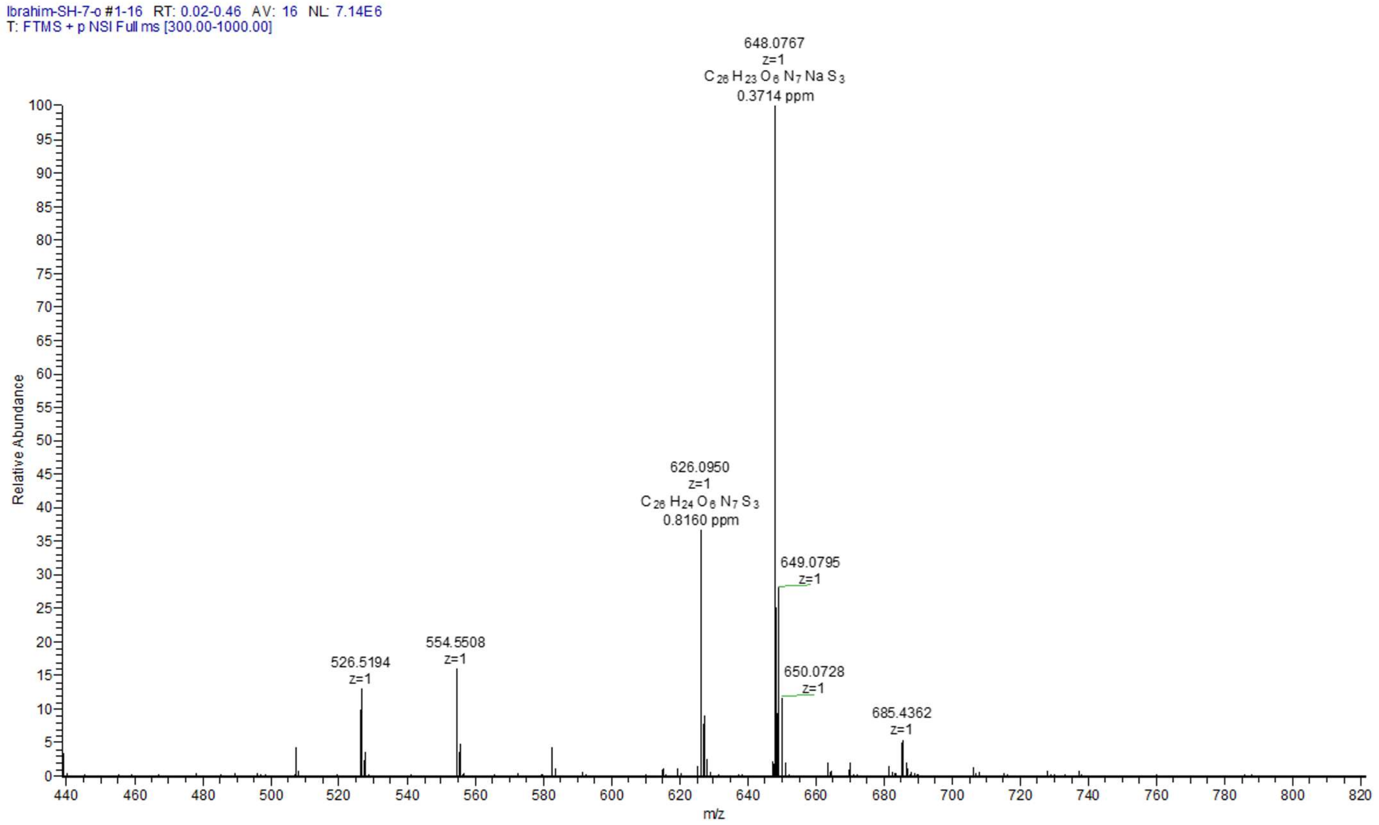

Figure S62. HRMS spectrum of compound **7o.**

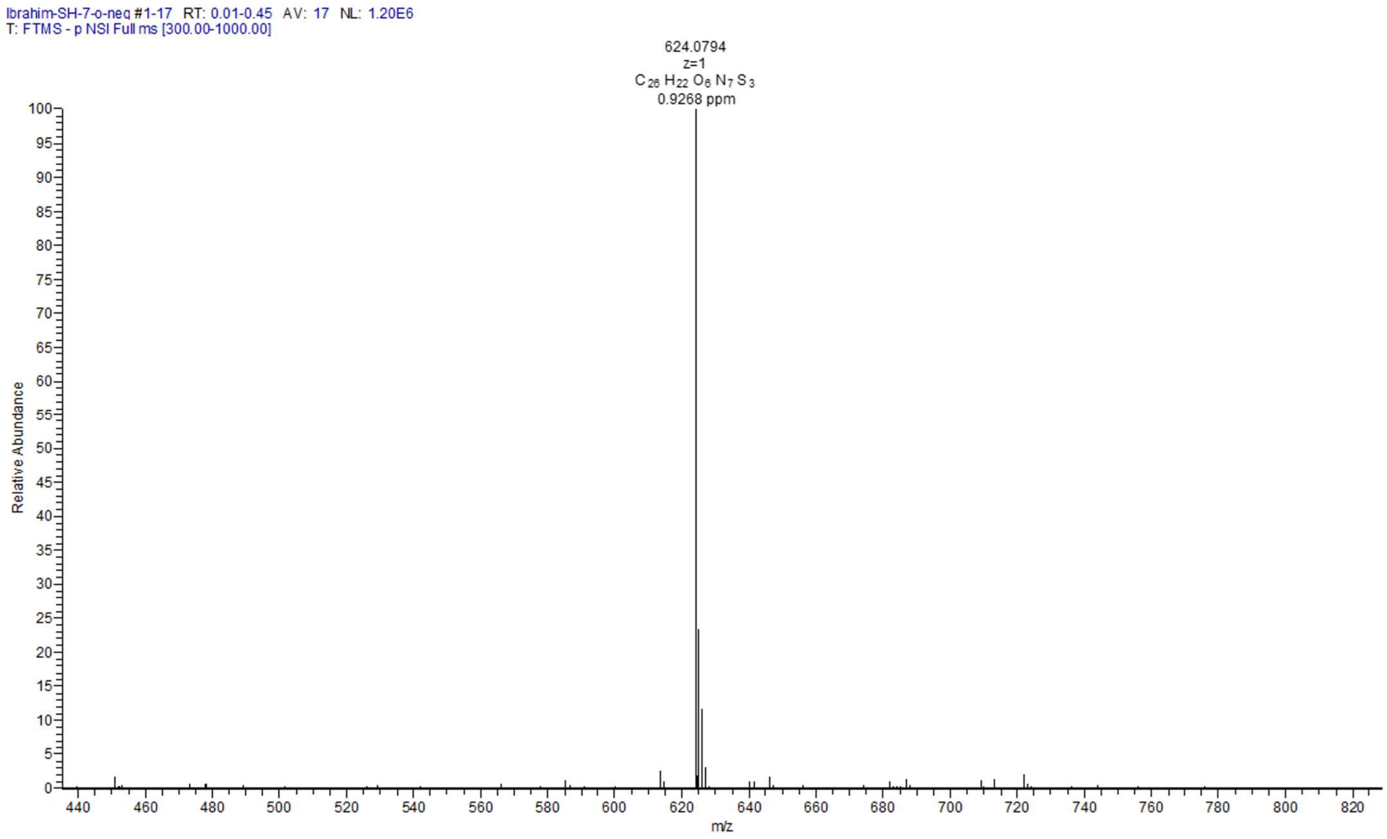

Figure S63. HRMS spectrum of compound **7o.**

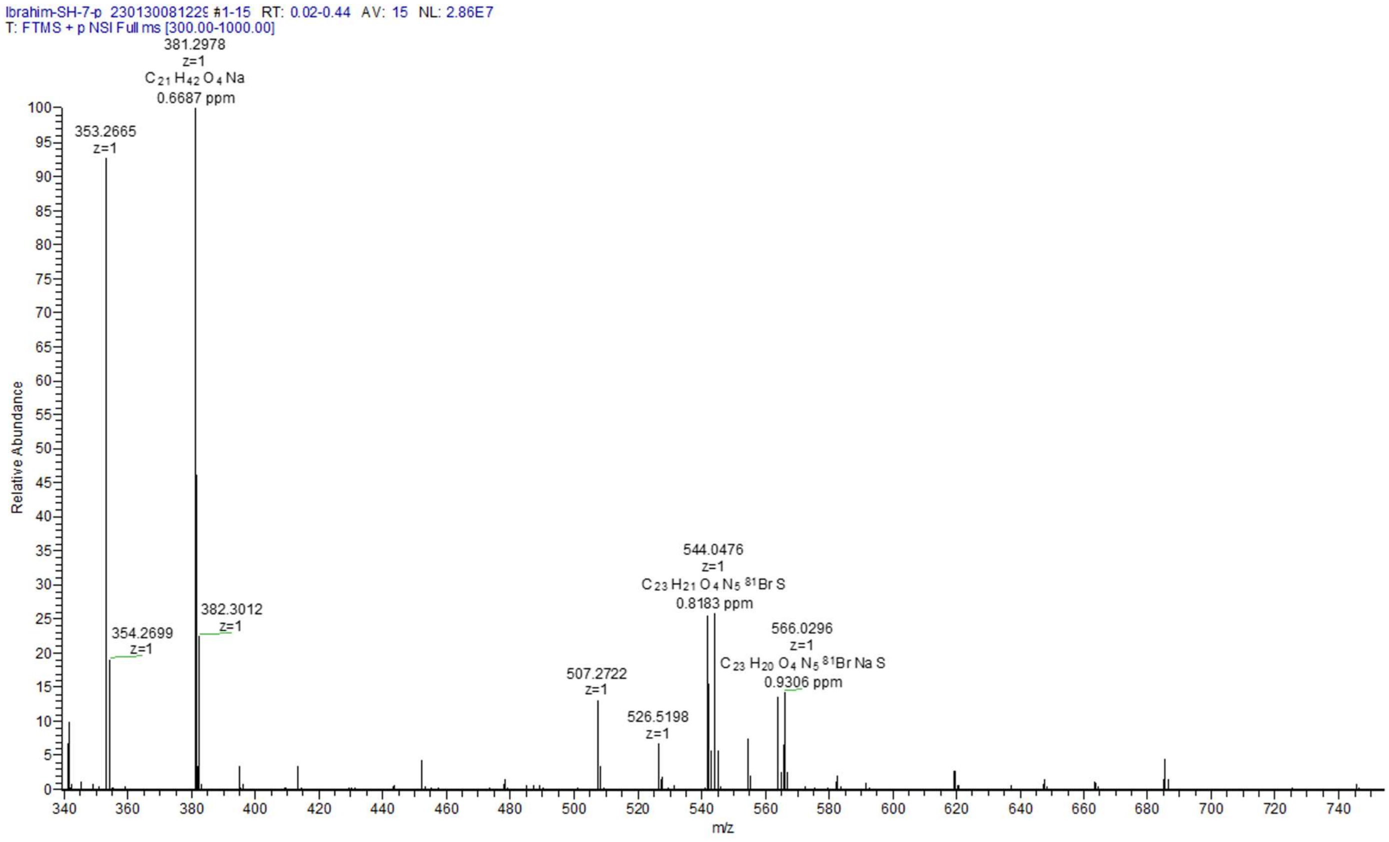

Figure S64. HRMS spectrum of compound **7p.**

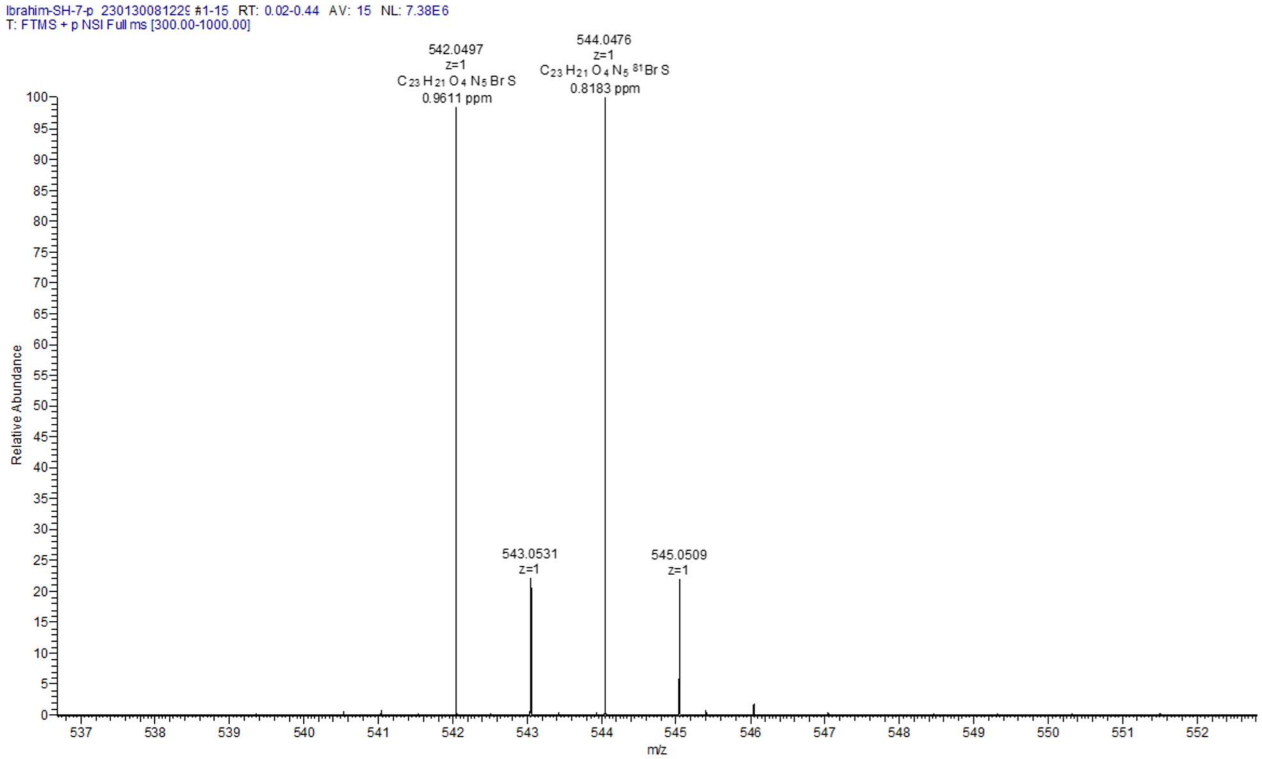

Figure S65. HRMS spectrum of compound **7p.**

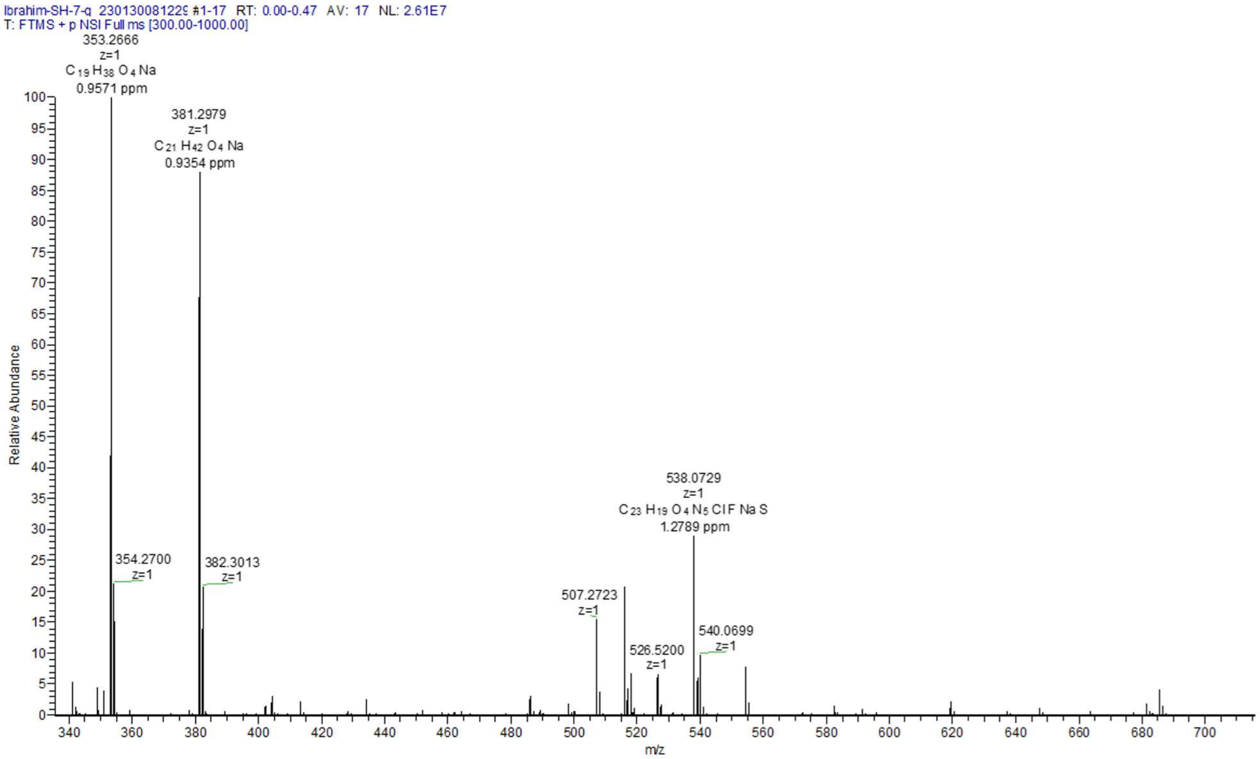

Figure S66. HRMS spectrum of compound **7q.**

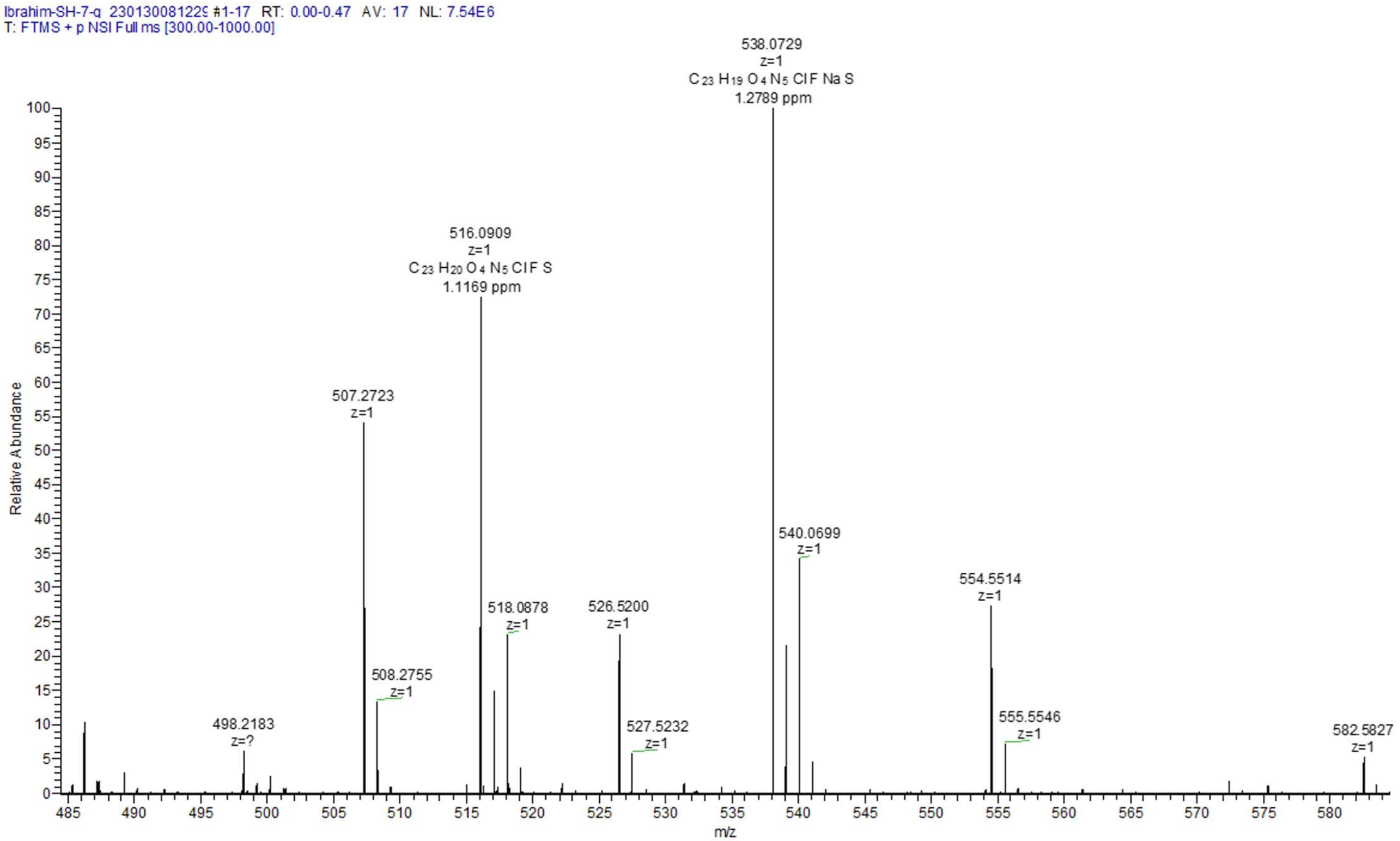

Figure S67. HRMS spectrum of compound **7q.**

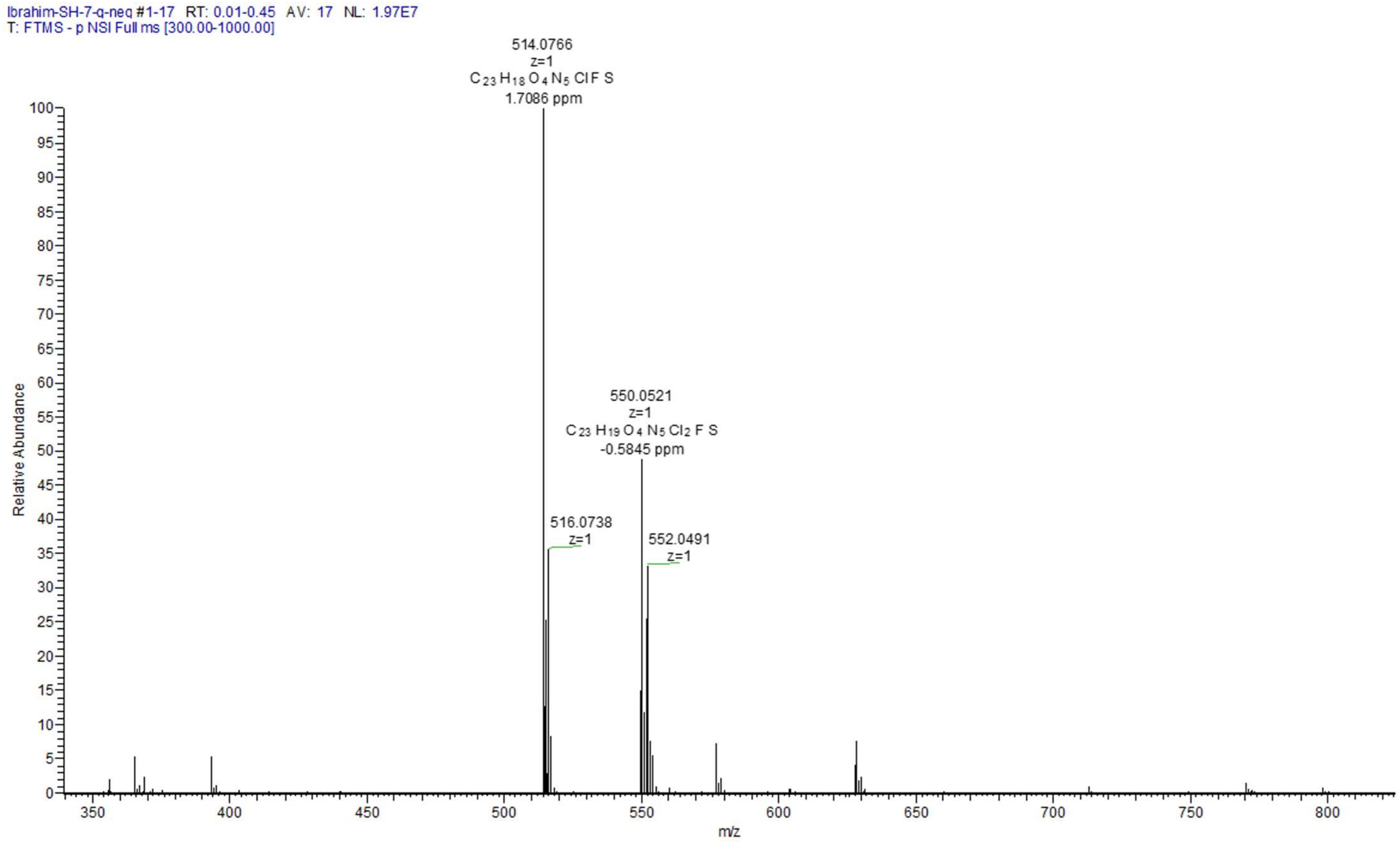

Figure S68. HRMS spectrum of compound **7q.**

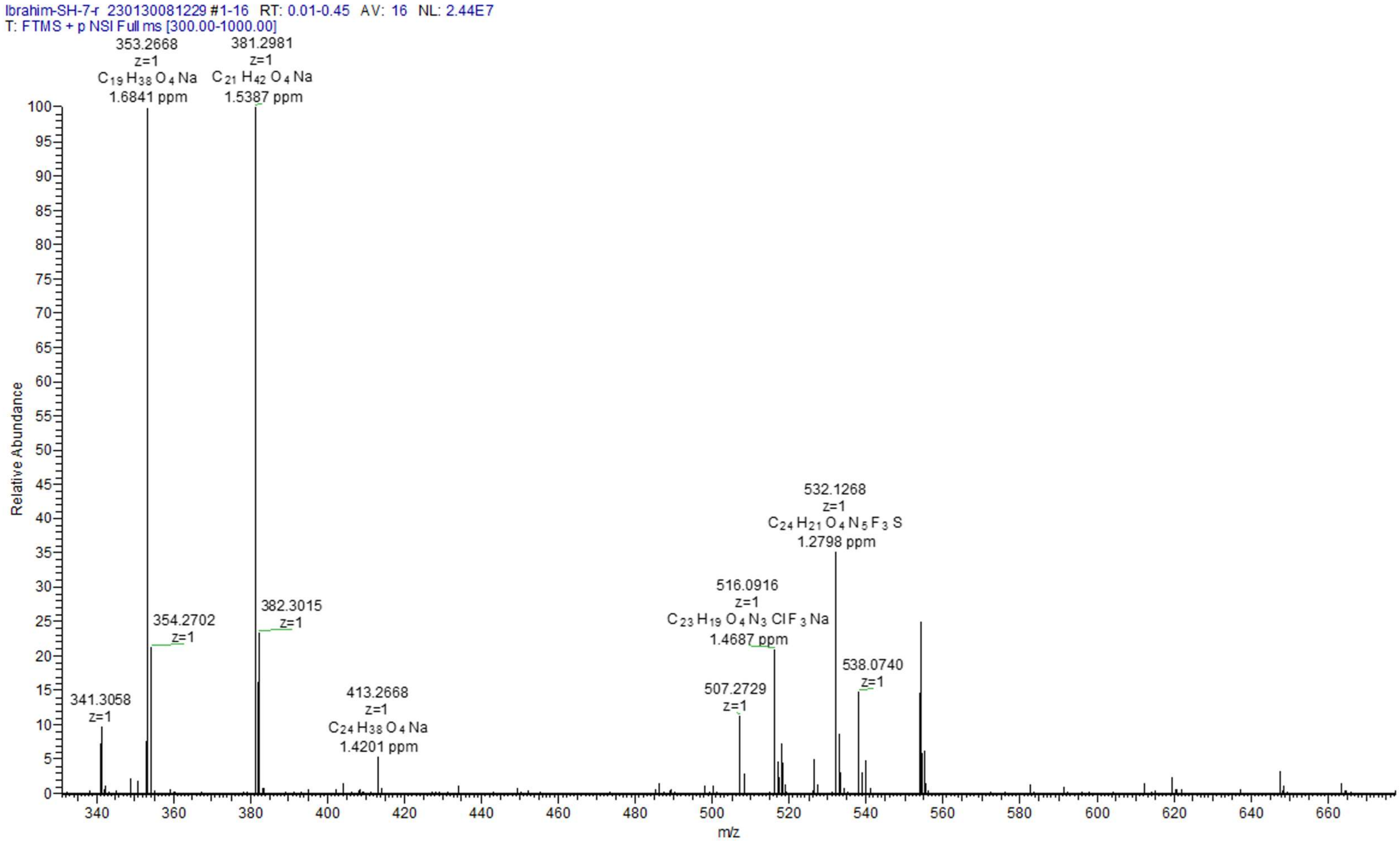

Figure S69. HRMS spectrum of compound **7r.**

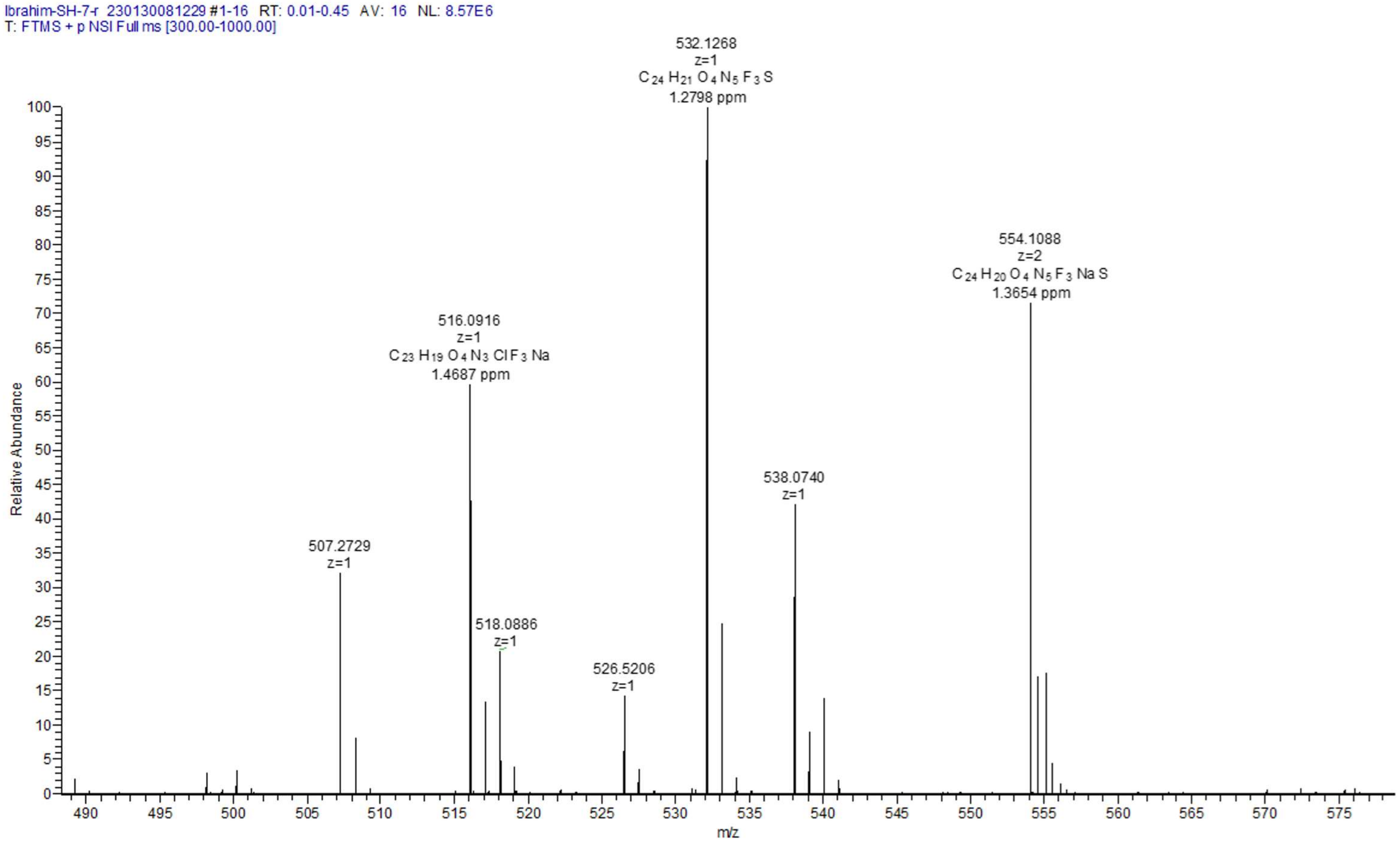

Figure S70. HRMS spectrum of compound **7r**

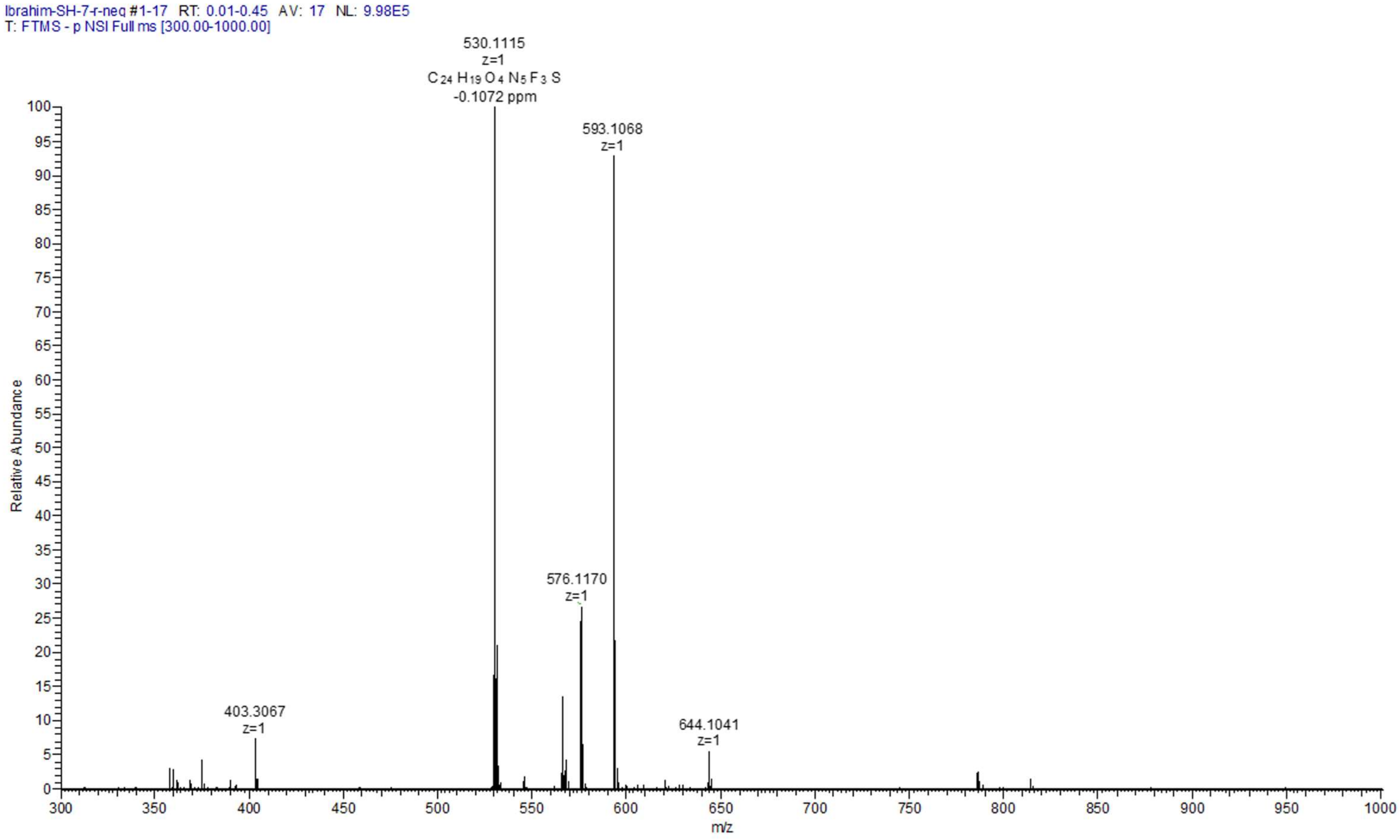

Figure S71. HRMS spectrum of compound **7r.**

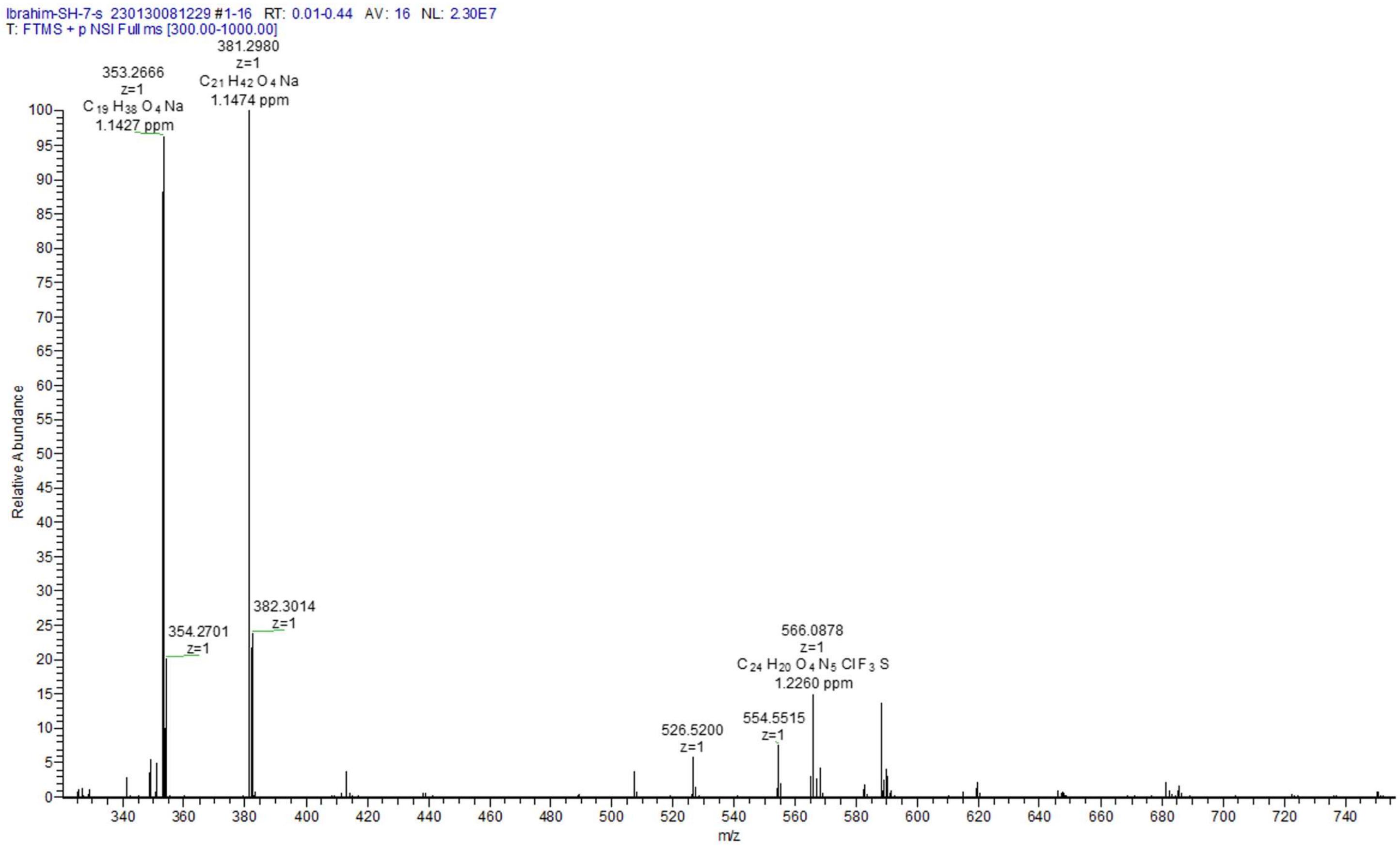

Figure S72. HRMS spectrum of compound **7s.**

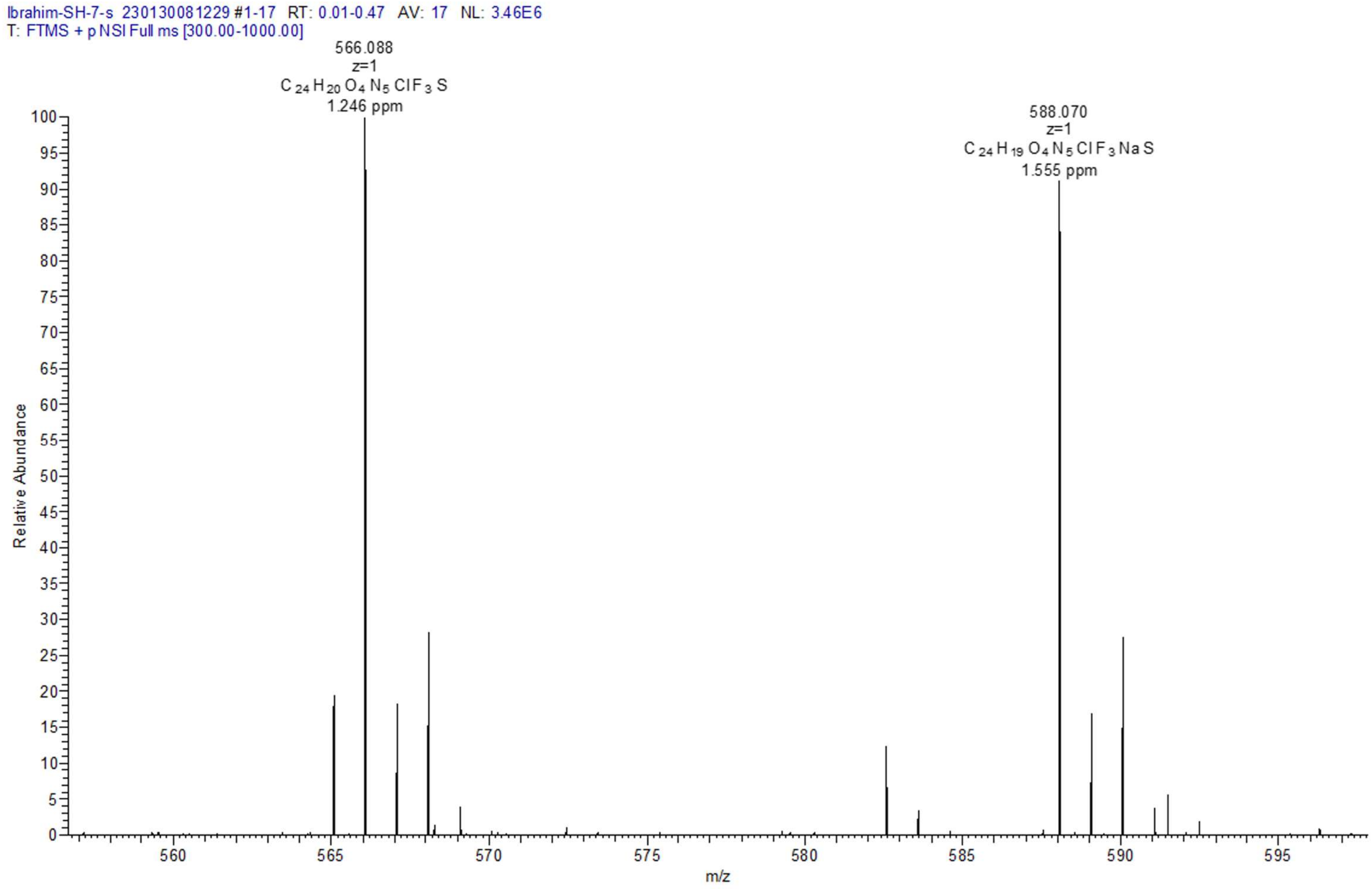

Figure S73. HRMS spectrum of compound **7s.**

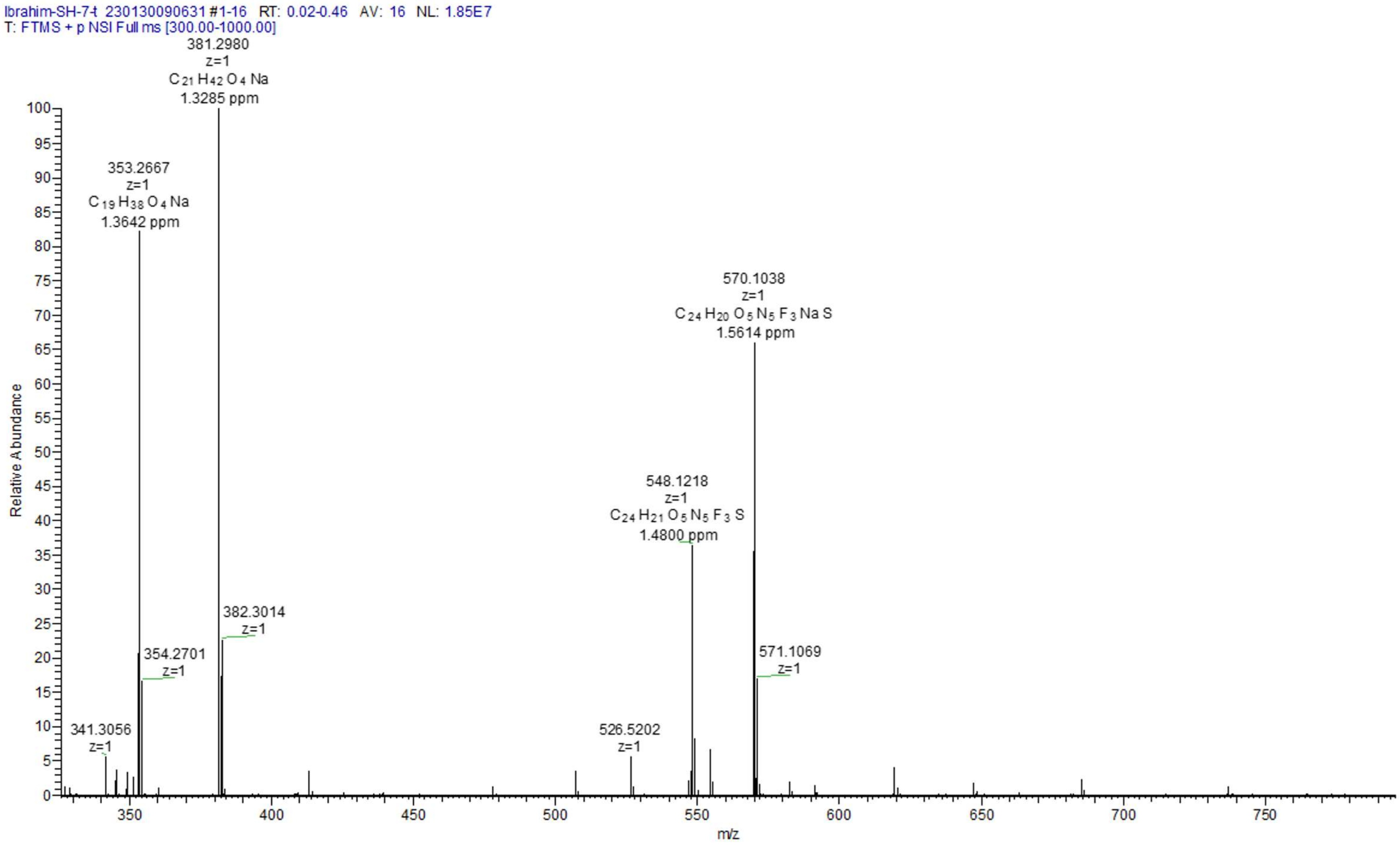

Figure S74. HRMS spectrum of compound **7t.**

HPLC Purity Charts

Analysenreport

Sample Information

Sample Name	:
Tray#	: 1
Vial#	: 66
Injection Volume	: 5
Data File	: SH-7a-1.lcd
Method File	: MSP5-95_30min_1.0.lcm
Batch File	: B_120123.lcb
Report Format File	: Reportformat2.lsr
Date Acquired	: 12.01.2023 11:07:26
Date Processed	: 12.01.2023 11:37:28
Comment	: MeOH/H2O/TFA 0,05%

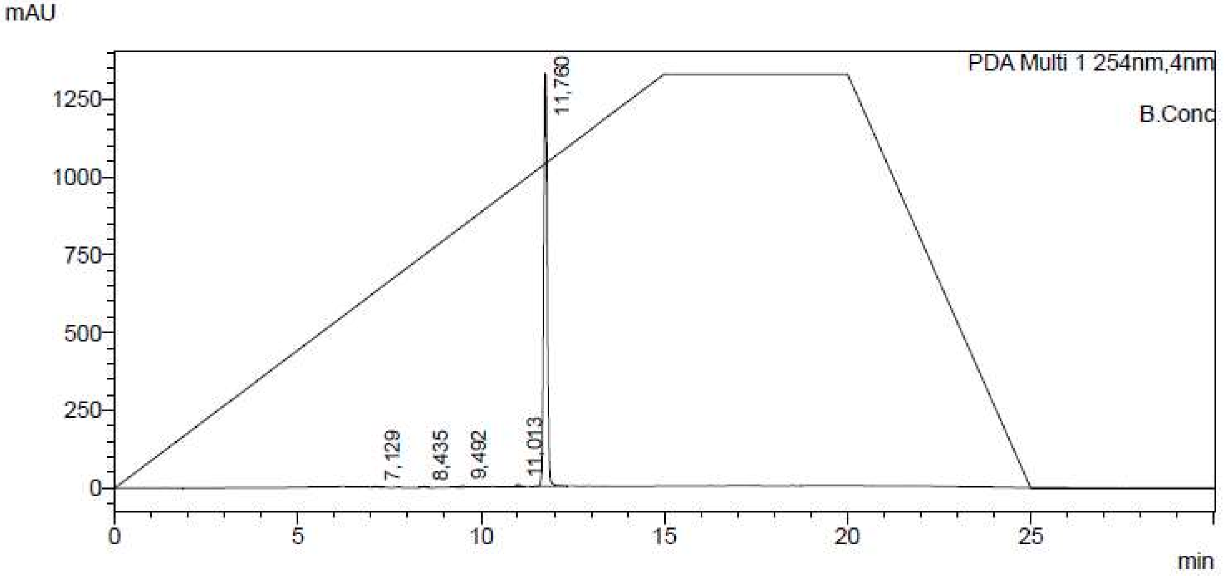

PDA Ch1 254nm

Peak#	Ret. Time	Area	Height	Area%
1	7,129	19817	3426	0,215
2	8,435	19304	3379	0,210
3	9,492	21738	3857	0,236
4	11,013	45085	7521	0,489
5	11,760	9106740	1324080	98,850
Total		9212685	1342262	100,000

Figure S76. HPLC chromatogram of compound **7a**

Analysenreport

Sample Information

Sample Name	:
Tray#	: 1
Vial#	: 67
Injection Volume	: 2
Data File	: SH-7b.lcd
Method File	: MSP5-95_30min_1.0.lcm
Batch File	: B_120123.lcb
Report Format File	: Reportformat2.lsr
Date Acquired	: 12.01.2023 11:37:56
Date Processed	: 12.01.2023 12:08:01
Comment	: MeOH/H2O/TFA 0,05%

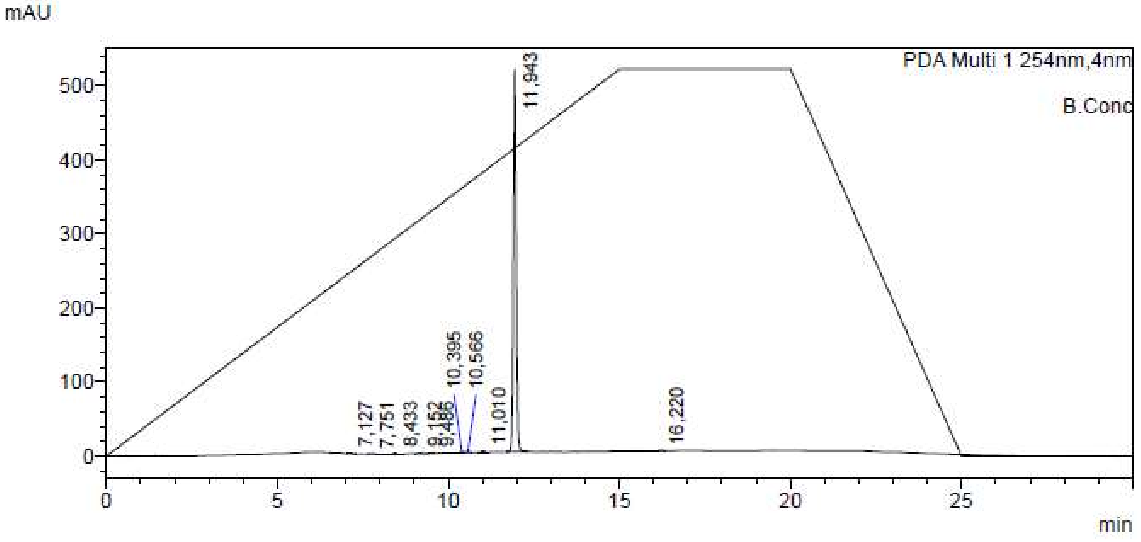

PDA Ch1 254nm

Peak#	Ret. Time	Area	Height	Area%
1	7,127	8694	1549	0,279
2	7,751	6151	926	0,197
3	8,433	9645	1682	0,309
4	9,152	6847	872	0,219
5	9,486	3820	744	0,122
6	10,395	9015	1569	0,289
7	10,566	5498	979	0,176
8	11,010	11217	2009	0,360
9	11,943	3054506	516052	97,917
10	16,220	4095	810	0,131
Total		3119489	527191	100,000

Figure S77. HPLC chromatogram of compound **7b**

Analysenreport

Sample Information

Sample Name	:
Tray#	: 1
Vial#	: 68
Injection Volume	: 2
Data File	: SH-7c.lcd
Method File	: MSP5-95_30min_1.0.lcm
Batch File	: B_120123.lcb
Report Format File	: Reportformat2.lsr
Date Acquired	: 12.01.2023 12:08:29
Date Processed	: 12.01.2023 12:38:32
Comment	: MeOH/H2O/TFA 0,05%

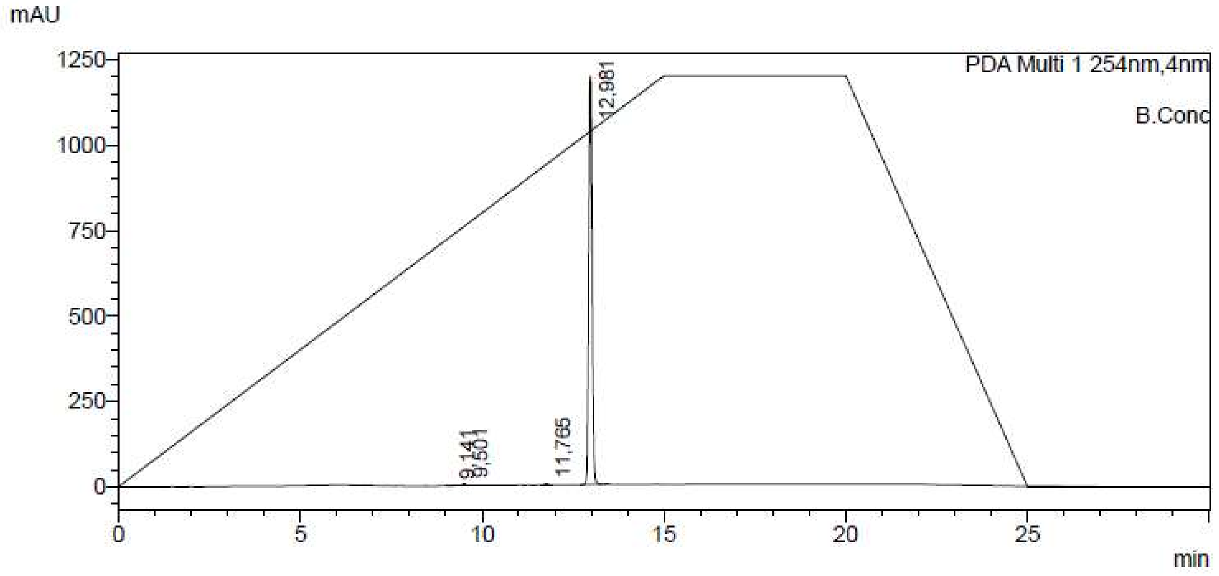

PDA Ch1 254nm

Peak#	Ret. Time	Area	Height	Area%
1	9,141	8456	979	0,110
2	9,501	32758	5784	0,425
3	11,765	21915	3789	0,284
4	12,981	7640076	1196863	99,180
Total		7703206	1207415	100,000

Figure S78. HPLC chromatogram of compound **7c**

Analysenreport

Sample Information

Sample Name	:
Tray#	: 1
Vial#	: 69
Injection Volume	: 2
Data File	: SH-7d-1.lcd
Method File	: MSP5-95_30min_1.0.lcm
Batch File	: B_120123.lcb
Report Format File	: Reportformat2.lsr
Date Acquired	: 12.01.2023 14:41:05
Date Processed	: 12.01.2023 15:11:07
Comment	: MeOH/H2O/TFA 0,05%

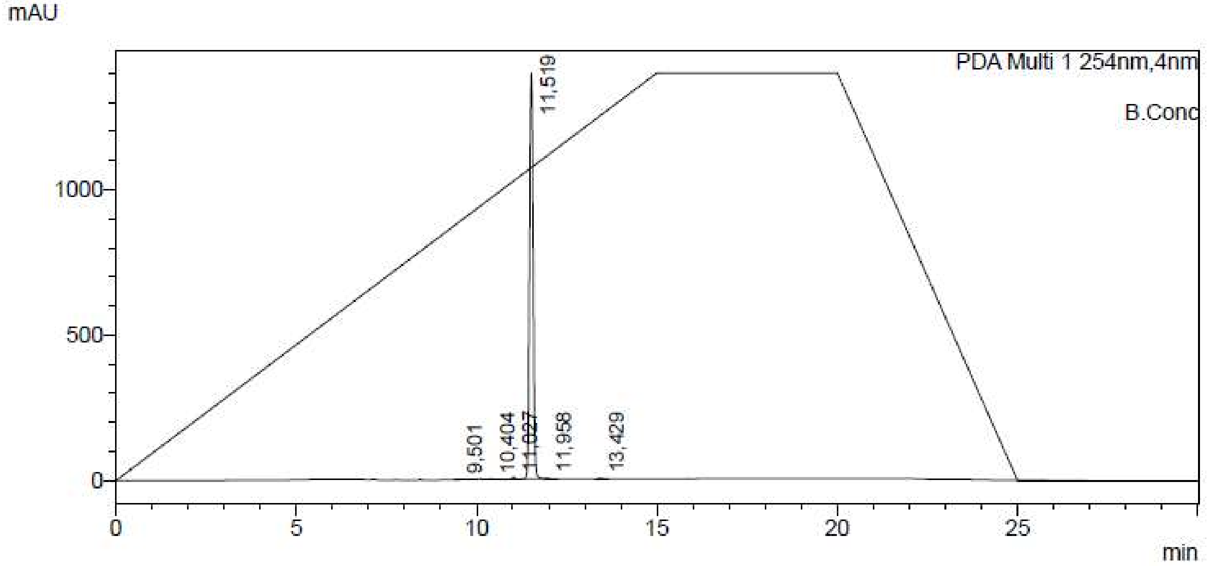

PDA Ch1 254nm

Peak#	Ret. Time	Area	Height	Area%
1	9,501	11045	2029	0,108
2	10,404	15556	2832	0,152
3	11,027	39293	6980	0,384
4	11,519	10112541	1395591	98,915
5	11,958	24411	3344	0,239
6	13,429	20642	2726	0,202
Total		10223487	1413503	100,000

Figure S79. HPLC chromatogram of compound **7d**

Analysenreport

Sample Information

Sample Name	:
Tray#	: 1
Vial#	: 70
Injection Volume	: 2
Data File	: SH-7e.lcd
Method File	: MSP5-95_30min_1.0.lcm
Batch File	: B_120123.lcb
Report Format File	: Reportformat2.lsr
Date Acquired	: 12.01.2023 13:09:31
Date Processed	: 12.01.2023 13:39:34
Comment	: MeOH/H2O/TFA 0,05%

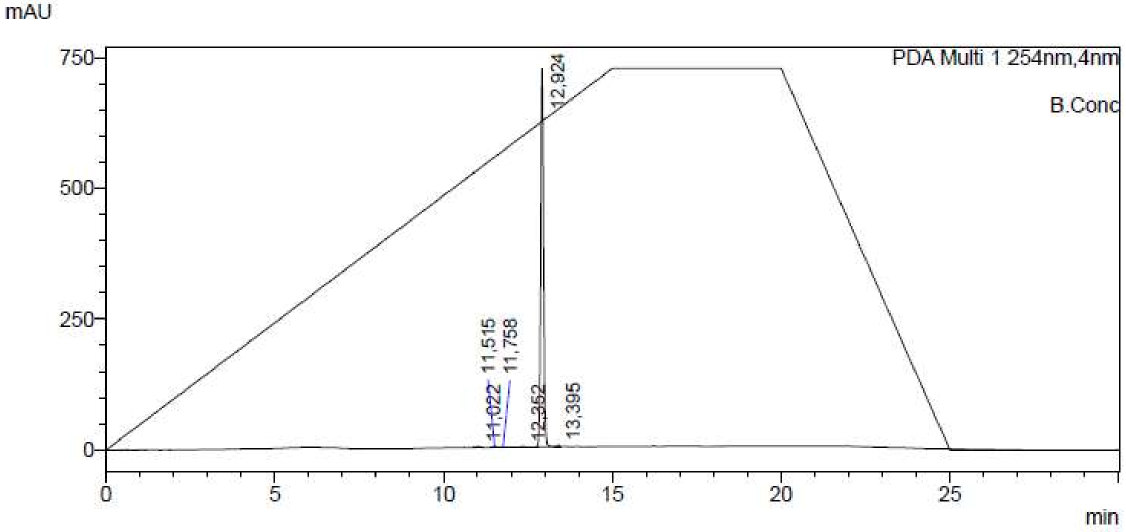

PDA Ch1 254nm

Peak#	Ret. Time	Area	Height	Area%
1	11,022	9503	1674	0,217
2	11,515	12385	1723	0,283
3	11,758	4366	757	0,100
4	12,352	6082	883	0,139
5	12,924	4320765	723011	98,872
6	13,395	16971	3215	0,388
Total		4370072	731262	100,000

Figure S80. HPLC chromatogram of compound **7e**

Analysenreport

Sample Information

Sample Name	:
Tray#	: 1
Vial#	: 71
Injection Volume	: 2
Data File	: SH-7f.lcd
Method File	: MSP5-95_30min_1.0.lcm
Batch File	: B_120123.lcb
Report Format File	: Reportformat2.lsr
Date Acquired	: 12.01.2023 13:40:02
Date Processed	: 12.01.2023 14:10:04
Comment	: MeOH/H2O/TFA 0,05%

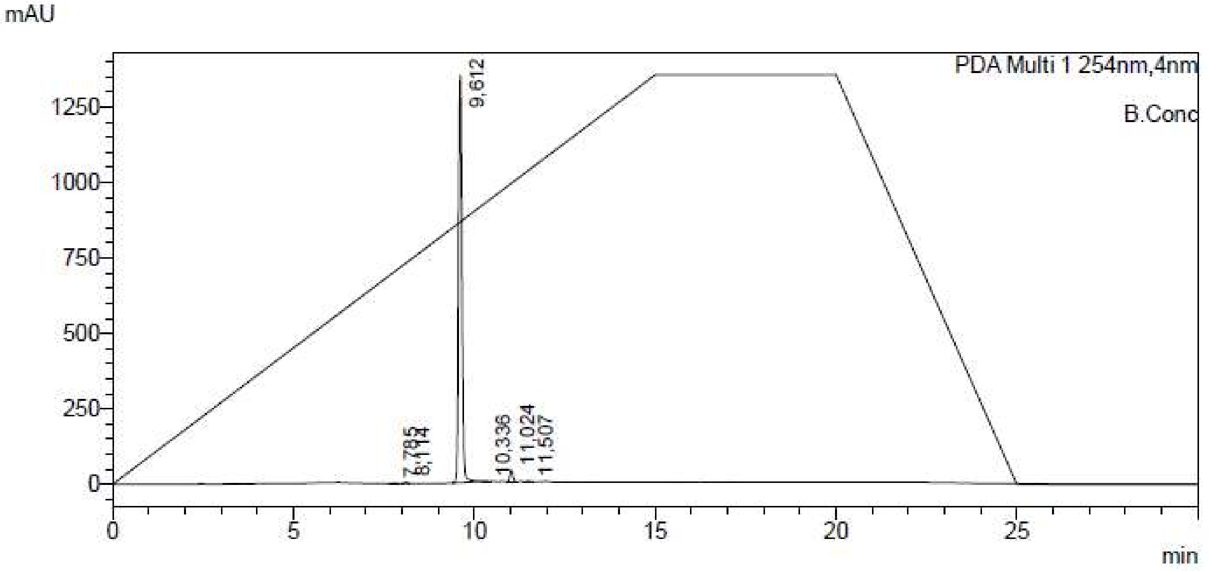

PDA Ch1 254nm

Peak#	Ret. Time	Area	Height	Area%
1	7,785	11111	1858	0,118
2	8,114	20234	3811	0,216
3	9,612	9105957	1351121	97,057
4	10,336	13964	2002	0,149
5	11,024	214310	37341	2,284
6	11,507	16538	2646	0,176
Total		9382114	1398778	100,000

Figure S81. HPLC chromatogram of compound **7f**

Analysenreport

Sample Information

Sample Name	:
Tray#	: 1
Vial#	: 72
Injection Volume	: 2
Data File	: SH-7g.lcd
Method File	: MSP5-95_30min_1.0.lcm
Batch File	: B_120123.lcb
Report Format File	: Reportformat2.lsr
Date Acquired	: 12.01.2023 14:10:33
Date Processed	: 12.01.2023 14:40:37
Comment	: MeOH/H2O/TFA 0,05%

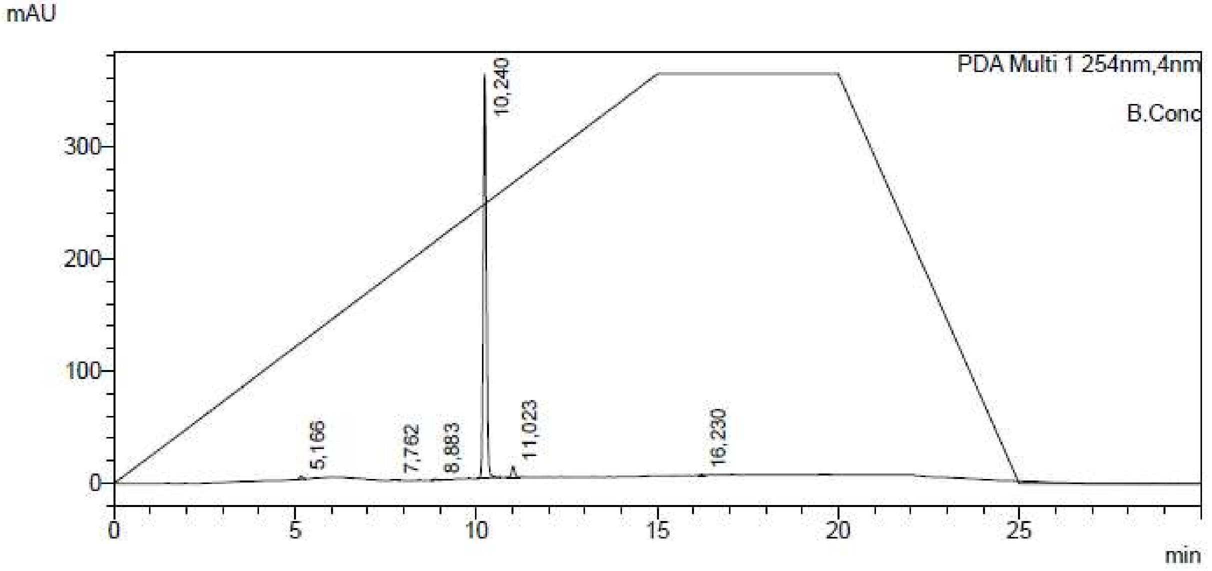

PDA Ch1 254nm

Peak#	Ret. Time	Area	Height	Area%
1	5,166	16473	2649	0,747
2	7,762	3624	666	0,164
3	8,883	7562	1262	0,343
4	10,240	2117421	359213	96,029
5	11,023	56085	9762	2,544
6	16,230	3812	802	0,173
Total		2204977	374354	100,000

Figure S82. HPLC chromatogram of compound **7g**

Analysenreport

Sample Information

Sample Name	:
Tray#	: 1
Vial#	: 73
Injection Volume	: 2
Data File	: SH-7h.lcd
Method File	: MSP5-95_30min_1.0.lcm
Batch File	: B_120123.lcb
Report Format File	: Reportformat2.lsr
Date Acquired	: 12.01.2023 15:11:35
Date Processed	: 12.01.2023 15:41:38
Comment	: MeOH/H2O/TFA 0,05%

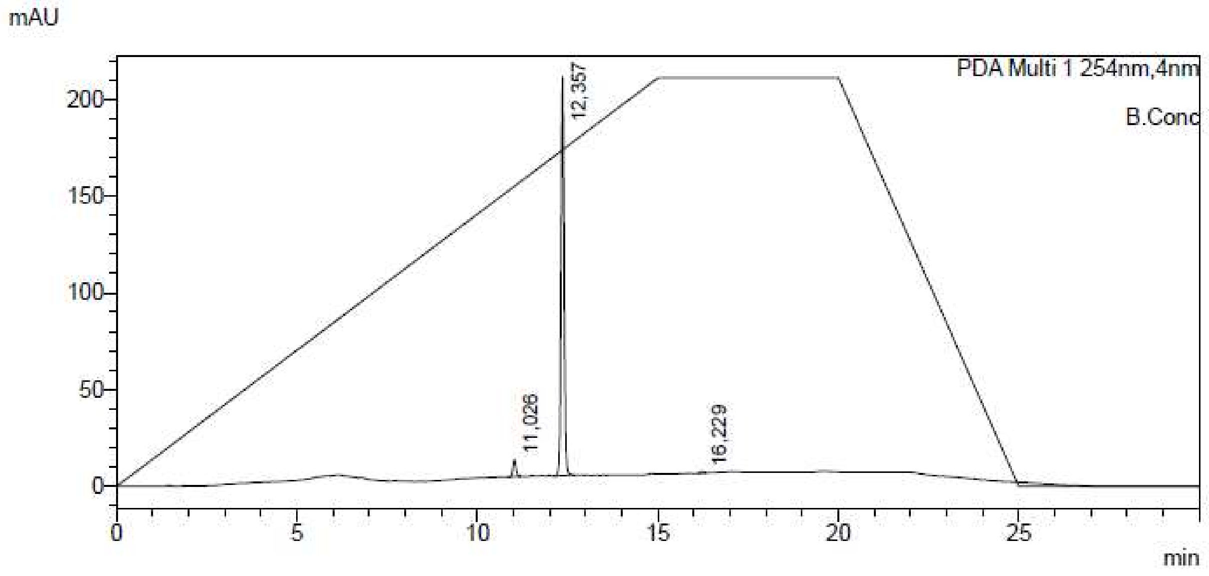

PDA Ch1 254nm

Peak#	Ret. Time	Area	Height	Area%
1	11,026	51169	8737	3,975
2	12,357	1232148	205526	95,722
3	16,229	3894	790	0,303
Total		1287211	215053	100,000

Figure S83. HPLC chromatogram of compound **7h**

Analysenreport

Sample Information

Sample Name	:
Tray#	: 1
Vial#	: 74
Injection Volume	: 2
Data File	: SH-7i.lcd
Method File	: MSP5-95_30min_1.0.lcm
Batch File	: B_120123.lcb
Report Format File	: Reportformat2.lsr
Date Acquired	: 12.01.2023 15:42:07
Date Processed	: 12.01.2023 16:12:09
Comment	: MeOH/H2O/TFA 0,05%

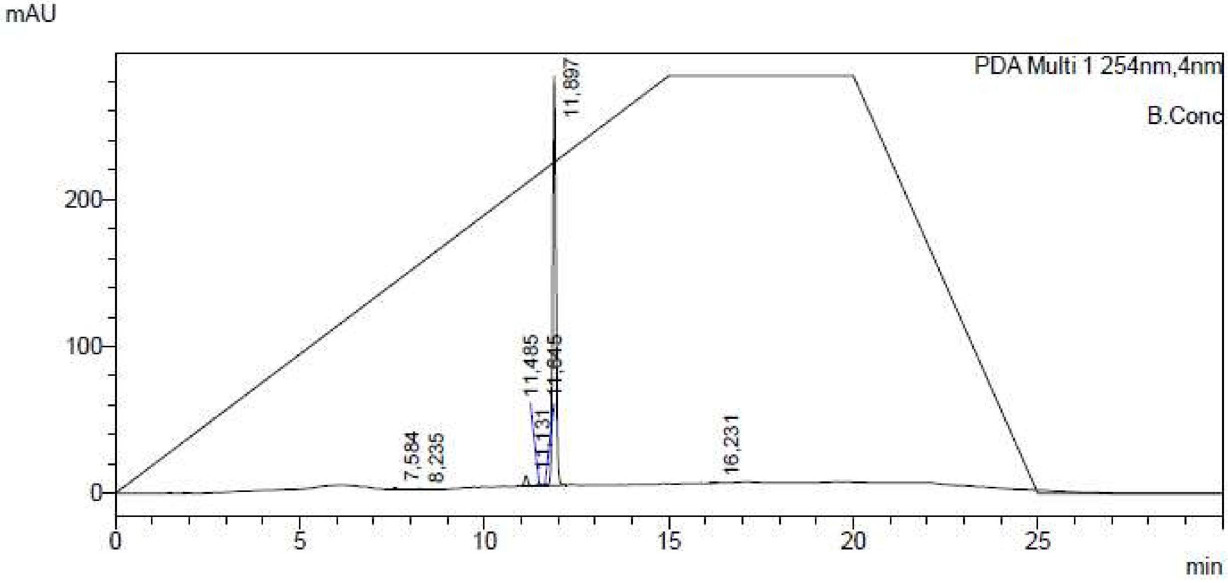

PDA Ch1 254nm

Peak#	Ret. Time	Area	Height	Area%
1	7,584	7836	1358	0,445
2	8,235	4165	747	0,236
3	11,131	38920	6819	2,208
4	11,485	10530	1808	0,597
5	11,645	6681	1115	0,379
6	11,897	1690843	278421	95,919
7	16,231	3802	755	0,216
Total		1762778	291023	100,000

Figure S84. HPLC chromatogram of compound **7i**

Analysenreport

Sample Information

Sample Name	:
Tray#	: 1
Vial#	: 75
Injection Volume	: 2
Data File	: SH-7j.lcd
Method File	: MSP5-95_30min_1.0.lcm
Batch File	: B_120123.lcb
Report Format File	: Reportformat2.lsr
Date Acquired	: 12.01.2023 16:12:39
Date Processed	: 12.01.2023 16:42:42
Comment	: MeOH/H2O/TFA 0,05%

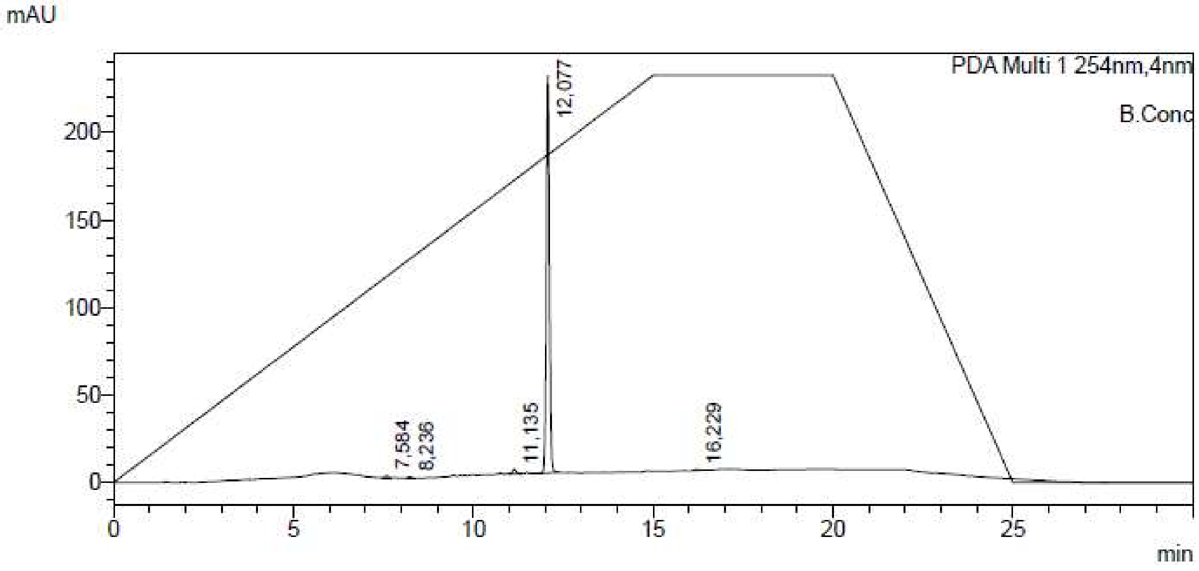

PDA Ch1 254nm

Peak#	Ret. Time	Area	Height	Area%
1	7,584	8448	1400	0,608
2	8,236	4460	803	0,321
3	11,135	18431	2771	1,326
4	12,077	1355132	227307	97,488
5	16,229	3584	739	0,258
Total		1390054	233019	100,000

Figure S85. HPLC chromatogram of compound **7j**

Analysenreport

Sample Information

Sample Name	:
Tray#	: 0
Vial#	: 1
Injection Volume	: 20
Data File	: SH-7k-WDH_1.lcd
Method File	: MSP5-95_30min_1.0.lcm
Batch File	: B_130123.lcb
Report Format File	: Reportformat2.lsr
Date Acquired	: 13.01.2023 08:45:16
Date Processed	: 13.01.2023 09:15:19
Comment	: MeOH/H2O/TFA 0,05%

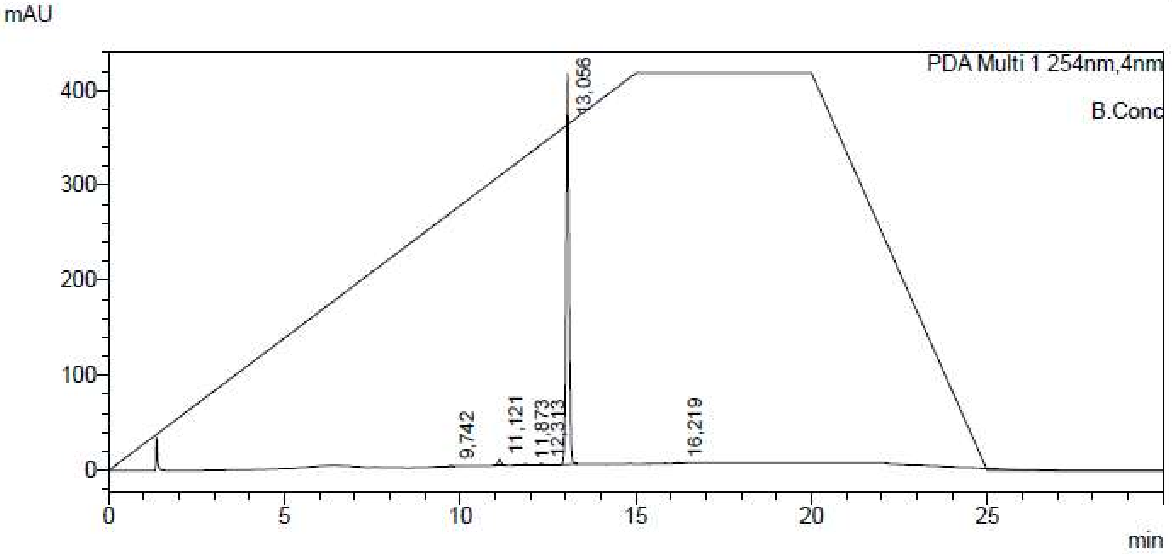

PDA Ch1 254nm

Peak#	Ret. Time	Area	Height	Area%
1	9,742	5752	1047	0,226
2	11,121	37942	6380	1,493
3	11,873	8020	1373	0,316
4	12,313	8283	1294	0,326
5	13,056	2477436	411640	97,507
6	16,219	3356	646	0,132
Total		2540789	422380	100,000

Figure S86. HPLC chromatogram of compound **7k**

Analysenreport

Sample Information

Sample Name	:
Tray#	: 1
Vial#	: 77
Injection Volume	: 2
Data File	: SH-7l.lcd
Method File	: MSP5-95_30min_1.0.lcm
Batch File	: B_120123.lcb
Report Format File	: Reportformat2.lsr
Date Acquired	: 12.01.2023 17:13:41
Date Processed	: 12.01.2023 17:43:44
Comment	: MeOH/H2O/TFA 0,05%

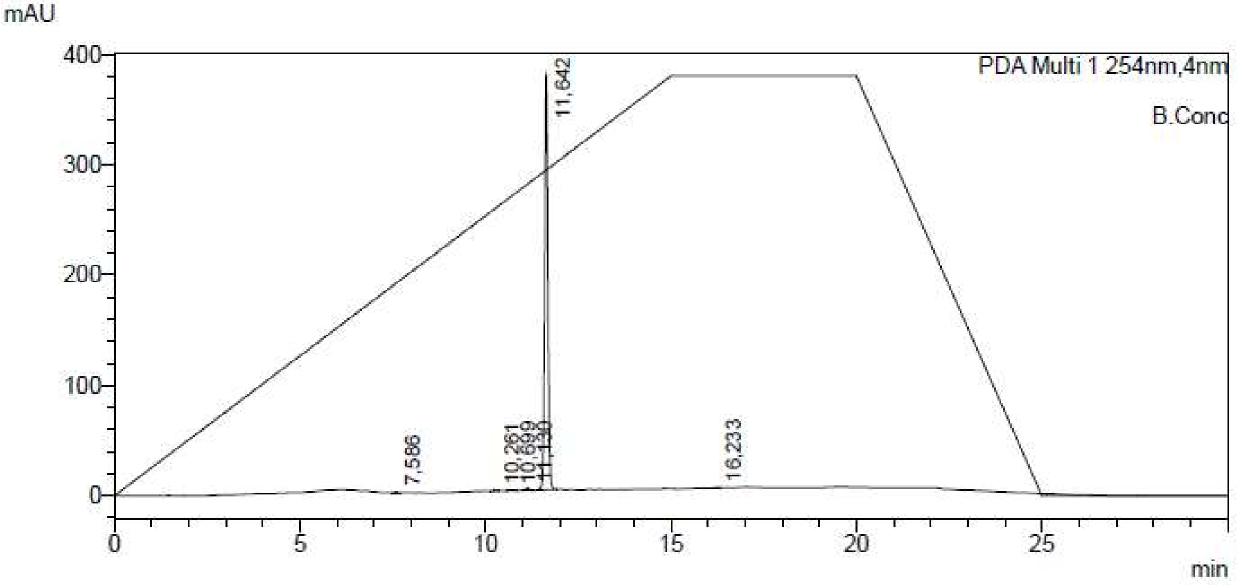

PDA Ch1 254nm

Peak#	Ret. Time	Area	Height	Area%
1	7,586	5311	917	0,229
2	10,261	4434	769	0,191
3	10,699	6927	913	0,298
4	11,130	12561	2118	0,541
5	11,642	2287718	374861	98,578
6	16,233	3766	769	0,162
Total		2320718	380346	100,000

Figure S87. HPLC chromatogram of compound **7l**

Analysenreport

Sample Information

Sample Name	:
Tray#	: 1
Vial#	: 79
Injection Volume	: 2
Data File	: SH-7n.lcd
Method File	: MSP5-95_30min_1.0.lcm
Batch File	: B_120123.lcb
Report Format File	: Reportformat2.lsr
Date Acquired	: 12.01.2023 18:14:43
Date Processed	: 12.01.2023 18:44:46
Comment	: MeOH/H2O/TFA 0,05%

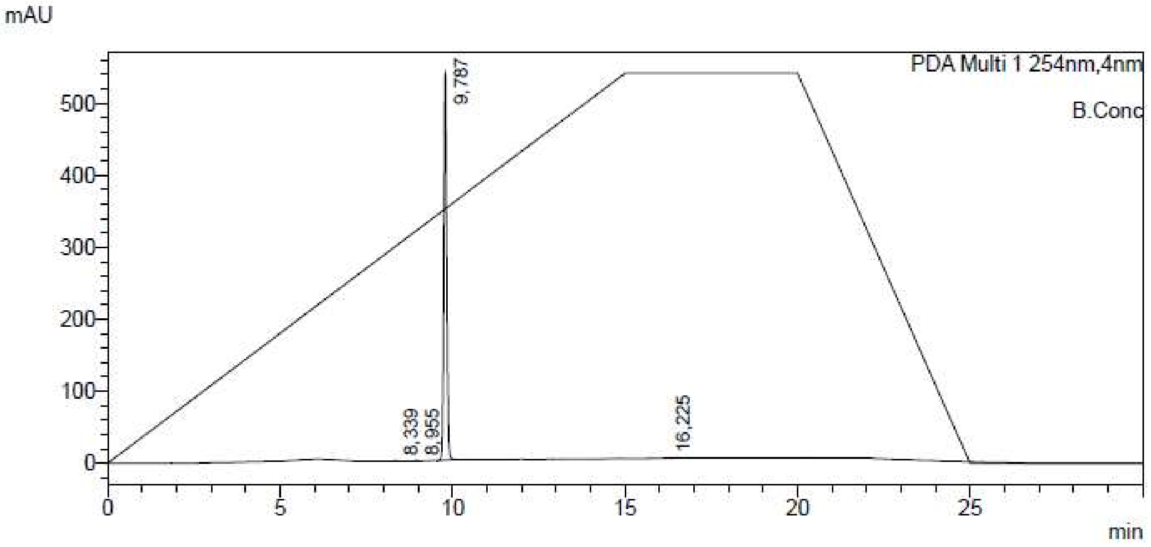

PDA Ch1 254nm

Peak#	Ret. Time	Area	Height	Area%
1	8,339	7946	1283	0,251
2	8,955	6571	1005	0,207
3	9,787	3149626	537701	99,420
4	16,225	3852	777	0,122
Total		3167995	540765	100,000

Figure S88. HPLC chromatogram of compound **7n**

Analysenreport

Sample Information

Sample Name	:
Tray#	: 1
Vial#	: 80
Injection Volume	: 2
Data File	: SH-7o.lcd
Method File	: MSP5-95_30min_1.0.lcm
Batch File	: B_120123.lcb
Report Format File	: Reportformat2.lsr
Date Acquired	: 12.01.2023 18:45:15
Date Processed	: 12.01.2023 19:15:17
Comment	: MeOH/H2O/TFA 0,05%

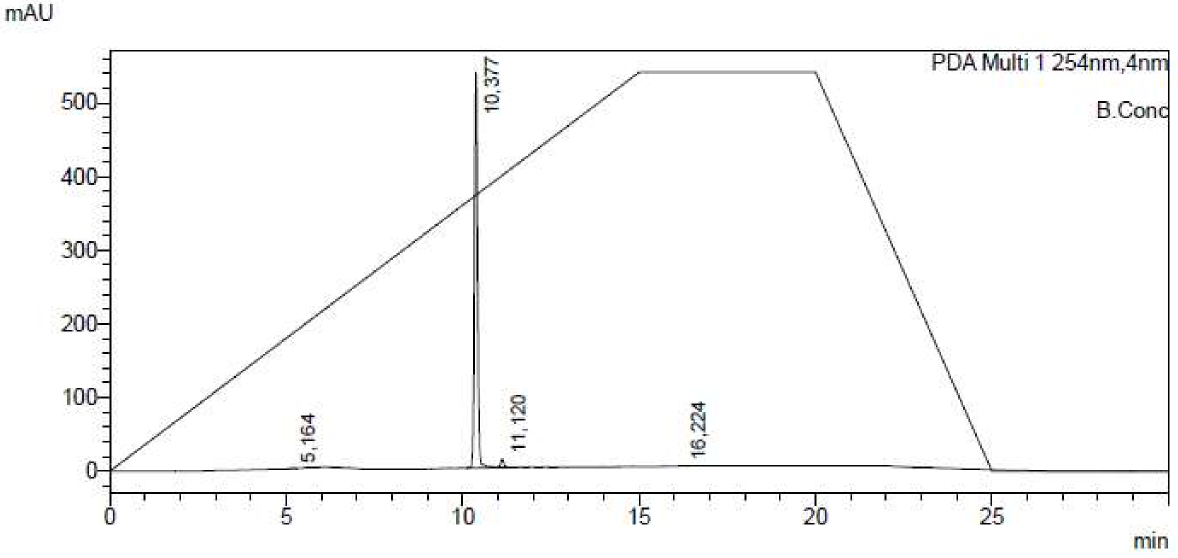

PDA Ch1 254nm

Peak#	Ret. Time	Area	Height	Area%
1	5,164	5840	952	0,175
2	10,377	3254441	537225	97,520
3	11,120	73227	11280	2,194
4	16,224	3687	752	0,110
Total		3337195	550209	100,000

Figure S89. HPLC chromatogram of compound **7o.**

Analysenreport

Sample Information

Sample Name	:
Tray#	: 1
Vial#	: 84
Injection Volume	: 2
Data File	: SH-7s.lcd
Method File	: MSP5-95_30min_1.0.lcm
Batch File	: B_120123.lcb
Report Format File	: Reportformat2.lsr
Date Acquired	: 12.01.2023 20:47:20
Date Processed	: 12.01.2023 21:17:22
Comment	: MeOH/H2O/TFA 0,05%

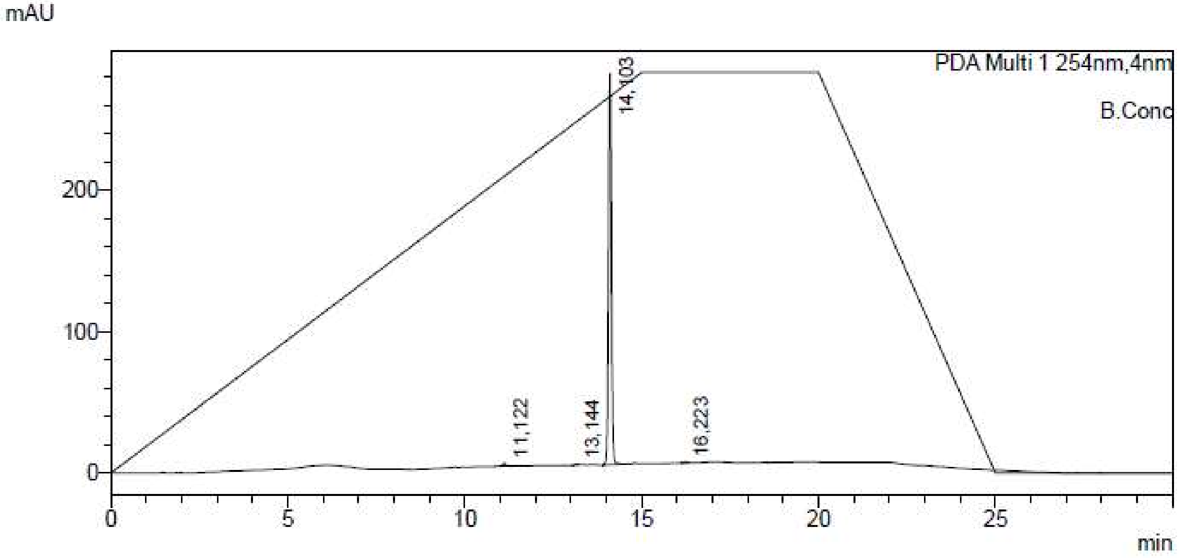

PDA Ch1 254nm

Peak#	Ret. Time	Area	Height	Area%
1	11,122	12872	2172	0,814
2	13,144	3565	709	0,226
3	14,103	1560708	277091	98,717
4	16,223	3854	791	0,244
Total		1580999	280762	100,000

Figure S90. HPLC chromatogram of compound **7s.**

Analysenreport

Sample Information

Sample Name	:
Tray#	: 1
Vial#	: 85
Injection Volume	: 2
Data File	: SH-7t.lcd
Method File	: MSP5-95_30min_1.0.lcm
Batch File	: B_120123.lcb
Report Format File	: Reportformat2.lsr
Date Acquired	: 12.01.2023 21:17:51
Date Processed	: 12.01.2023 21:47:52
Comment	: MeOH/H2O/TFA 0,05%

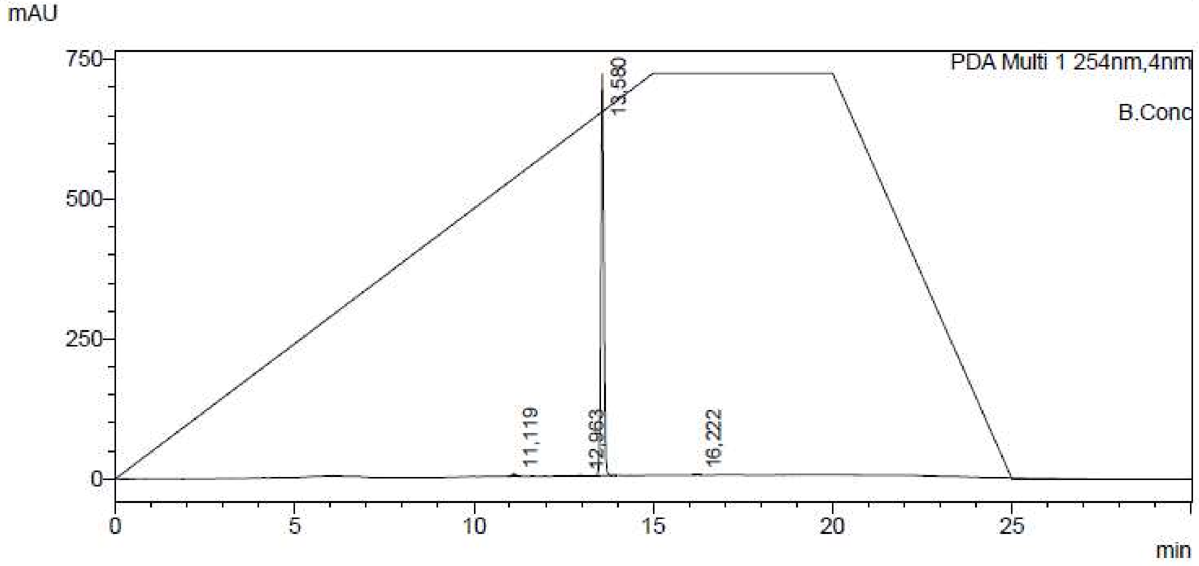

PDA Ch1 254nm

Peak#	Ret. Time	Area	Height	Area%
1	11,119	29456	4960	0,702
2	12,963	6494	1153	0,155
3	13,580	4158991	719503	99,053
4	16,222	3830	769	0,091
Total		4198770	726385	100,000

Figure S91. HPLC chromatogram of compound **7t.**

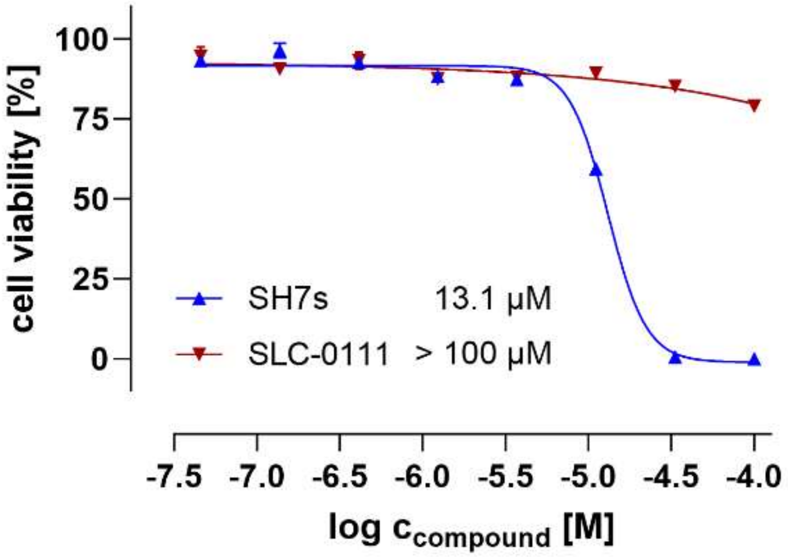

Figure S92. Dose-response curves of the anti-proliferative/cytotoxic effects of the derivative SH7s and the CA reference inhibitor SLC-0111 on human HCT-116 colorectal cancer cells, as measured after 48 h treatment by using a fluorometric resazurin-based cell viability assays. The data represent two biological replicates, each comprising technical triplicates. The given values are relative IC_{50} values. The standard errors are expressed as ±SEM. Data analysis was done by using GraphPad Prism 10.1 software.

Table S1. Kinase Assay profiling results for compound **SH7s**.

Kinases	% Enzyme Activity (relative to DMSO controls)	
	SH7s (20μM)	
	Data 1	Data 2
ABL1 (E255K)	103.34	99.50
ABL1 (E255V)	96.59	96.13
ABL1 (F317I)	101.71	101.68
ABL1 (F317L)	105.15	103.45
ABL1 (G250E)	102.03	101.59
ABL1 (H396P)	105.70	105.57
ABL1 (M351T)	105.35	105.32
ABL1 (Q252H)	88.44	87.29
ABL1 (T315I)	99.66	99.04
ABL1 (V299L)	104.67	102.20
ABL1 (Y253F)	105.85	105.41
ABL1 (Y253H)	109.39	107.47
AKT1 (E17K)	110.82	106.15
AKT2 (E17K)	97.43	94.60
AKT3 (E17K)	112.63	110.30
AKT3 (G171R)	101.53	98.51
ALK (C1156Y)	88.52	86.90
ALK (F1174L)	101.78	98.20
ALK (F1174L)-EML4	92.09	89.33
ALK (F1174L)-NPM1	99.66	95.52
ALK (F1174S)	110.50	109.74
ALK (G1202R)	106.81	106.41
ALK (G1269A)	107.86	107.71
ALK (G1269S)	98.79	98.21
ALK (L1152R)	103.21	101.95
ALK (L1196M)	103.69	101.08
ALK (R1275Q)	98.94	98.41
ALK (S1206R)	110.89	110.61
ALK (T1151-L1152insT)	105.99	104.66
ALK (T1151M)	97.18	96.60
ALK2 (Q207D)	33.35	29.45
ALK2 (R206H)	55	54.91
ALK-KIF5B (Kex24Aex20)	91.83	90.86
ALK-KLC1 (Kex8Aex20)	106.79	104.22
ALK-NPM1	97.94	97.84
ALK-TFG	100.53	98.57
ALK-TFG (Tex4Aex20)	96.23	95.80
ALK-TPM1	104.91	102.80
ALK-TPM3	104.30	104.27
Aurora B (G160L)	110.71	104.14
AXL (R499C)	109.1	107.15

**BRAF (d485-489/P490Y)**	87.36	86.84
**BRAF (G464V)**	90.70	87.35
**BRAF (G469A)**	96.73	96.42
**BRAF (K601E)**	95.05	92.03
**BRAF (L597V)**	108.13	105.16
**BRAF (R506_K507insVLR)**	98.49	97.88
**BRAF (T599_V600insT)**	104.34	102.34
**BRAF (V599E)**	88.51	87.62
**BRAF (V600A)**	98.86	97.52
**BRAF (V600D)**	89.07	87.73
**BRAF (V600K)**	104.17	102.51
**BRAF-FAM131B (Fex2Bex9)**	108.76	107.89
**BRAF-KIAA1549 (Kex15Bex9)**	96.08	95.90
**BRAF-KIAA1549 (Kex16Bex9)**	88.74	87.78
**BRAF-SRGAP3 (Sex12Bex9)**	104.40	98.00
**BTK (C481S)**	107.17	106.91
**BTK (E41K)**	106.95	105.30
**BTK (P190K)**	111.94	109.14
**CHK2 (I157T)**	104.42	103.28
**CK1epsilon (R178C)**	93.21	92.66
**c-Kit (A829P)**	113.02	112.63
**c-Kit (d557-558)**	93.73	93.26
**c-Kit (D816E)**	96.08	95.39
**c-Kit (D816F)**	107.49	106.53
**c-Kit (D816H)**	99.46	98.43
**c-Kit (D816I)**	98.51	96.27
**c-Kit (D816V)**	73.20	70.47
**c-Kit (D816Y)**	108.36	107.00
**c-Kit (D820E)**	96.04	94.69
**c-Kit (D820Y)**	98.07	93.10
**c-Kit (K642E)**	89.24	86.09
**c-Kit (T670I)**	83.21	82.98
**c-Kit (V559A)**	97.74	96.13
**c-Kit (V559D)**	104.02	103.46
**c-Kit (V559D/T670I)**	100.49	100.14
**c-Kit (V559D/V654A)**	95.19	93.96
**c-Kit (V560G)**	91.89	91.57
**c-Kit (V560G/D816V)**	110.05	107.99
**c-Kit (V560G/N822K)**	107.51	106.26
**c-Kit (V654A)**	97.00	84.95
**c-Kit (Y823D)**	98.22	97.82
**c-MER (A708S)**	82.73	82.70
**c-MET (D1228H)**	112.62	106.79
**c-MET (D1228N)**	110.82	107.33
**c-MET (F1200I)**	107.15	105.35
**c-MET (K1244R)**	109.69	109.22
**c-MET (M1250I)**	117.20	112.66

**c-MET (M1250T)**	102.03	99.96
**c-MET (P991S)**	129.53	128.41
**c-MET (T1173I)**	110.18	109.13
**c-MET (T992I)**	103.76	103.69
**c-MET (V1092I)**	110.24	109.10
**c-MET (Y1230A)**	107.15	105.34
**c-MET (Y1230C)**	114.24	113.96
**c-MET (Y1230D)**	96.45	95.97
**c-MET (Y1230H)**	116.25	115.88
**c-MET (Y1230S)**	101.42	100.26
**c-MET (Y1235D)**	92.99	85.03
**c-MET-KIF5B (Kex24Mex14)**	100.54	98.79
**c-MET-TFG (Tex5Mex15)**	92.07	90.58
**c-Src (T341M)**	109.68	104.17
**DDR2 (N456S)**	95.54	95.03
**DDR2 (T654M)**	100.08	97.02
**EGFR (A763_Y764insFHEA)**	108.98	108.71
**EGFR (A763_Y764insFQEA)**	103.56	102.02
**EGFR (C775S/T790M/L858R)**	142.60	140.61
**EGFR (C797A)**	94.19	91.03
**EGFR (C797S)**	101.61	101.56
**EGFR (C797S/L858R)**	101.40	100.42
**EGFR (d746)**	95.02	93.01
**EGFR (d746-750)**	96.82	93.55
**EGFR (d746-750/C775S/T790M/L858R)**	99.62	98.28
**EGFR (d746-750/C797A)**	105.74	104.88
**EGFR (d746-750/C797S)**	101.81	99.43
**EGFR (d746-750/T790M)**	95.74	95.53
**EGFR (d746-750/T790M/C797S)**	104.12	102.30
**EGFR (d747-749)**	100.48	99.65
**EGFR (d747-749/A750P)**	104.28	101.42
**EGFR (d747-752/P753S)**	108.50	108.14
**EGFR (d752-759)**	85.43	74.93
**EGFR (D761Y)**	100.77	99.65
**EGFR (D770_N771insNPG)**	99.62	98.35
**EGFR (D770_N771insNPG/T790M)**	99.89	99.62
**EGFR (D770GY)**	100.07	99.46
**EGFR (G719C)**	106.29	105.03
**EGFR (G719D)**	105.90	105.67
**EGFR (G719S)**	98.33	95.15
**EGFR (K716A)**	106.99	104.67
**EGFR (K716A/C797S/L858R)**	109.62	108.47
**EGFR (K716Q/L718Q)**	104.68	103.86
**EGFR (K728A)**	97.43	96.14
**EGFR (L718Q)**	93.29	91.36
**EGFR (L747S)**	104.46	102.65

**EGFR (L792F)**	107.60	107.47
**EGFR (L792F/L858R)**	92.47	92.18
**EGFR (L792H/C797S/L858R)**	99.41	96.84
**EGFR (L858R)**	90.73	88.97
**EGFR (L858R, T790M)**	101.05	98.56
**EGFR (L861Q)**	106.89	100.01
**EGFR (T790M)**	100.74	99.07
**EGFR (T790M/C797S)**	102.01	97.76
**EGFR (T790M/C797S/L858R)**	100.39	100.22
**EGFR (T790M/L792F/C797S/L858R)**	100.82	99.54
**EGFR (T790M/L792F/L858R)**	102.40	101.66
**EGFR (T790M/L792H/C797S/L858R)**	119.48	115.17
**EGFR (T790M/L792H/L858R)**	107.68	105.07
**ERBB2 (D769H)**	104.97	103.68
**ERBB2 (D769Y)**	102.59	100.17
**ERBB2 (P1170A)**	99.11	94.9
**ERBB2 (P780_Y781insGSP)**	102.67	101.67
**ERBB2 (R896C)**	103.28	103.06
**ERBB2 (V777_G778insCG)**	103.69	102.35
**ERBB2 (V777L)**	107.71	106.76
**FGFR1 (V561M)**	95.81	93.40
**FGFR1OP-FGFR1**	98.23	97.11
**FGFR2 (C491A)**	95.17	93.41
**FGFR2 (C491F)**	93.41	92.99
**FGFR2 (C491S)**	109.50	108.23
**FGFR2 (E565G)**	107.52	105.13
**FGFR2 (K526E)**	95.36	94.51
**FGFR2 (K641R)**	105.94	102.91
**FGFR2 (K659N)**	102.79	102.25
**FGFR2 (N549H)**	108.63	108.02
**FGFR2 (R612T)**	96.18	94.76
**FGFR2 (V564F)**	102.85	102.27
**FGFR3 (G697C)**	102.97	96.38
**FGFR3 (K650E)**	105.98	103.84
**FGFR3 (K650M)**	103.98	101.79
**FGFR3 (K650Q)**	92.33	90.69
**FGFR3 (V555M)**	107.98	105.77
**FGFR4 (N535K)**	103.23	98.99
**FGFR4 (V550E)**	101.62	99.79
**FGFR4 (V550L)**	117.62	117.20
**FGFR4 (V550M)**	96.00	93.38
**FLT3 (D835Y)**	92.76	92.42
**FLT3 (F594_R595insR)**	89.45	86.53
**FLT3 (F594_R595insREY)**	96.00	92.70
**FLT3 (ITD)**	82.84	81.57
**FLT3 (ITD)-NPOS**	99.13	97.61
**FLT3 (ITD)-W51**	86.12	84.81

**FLT3 (R595_E596insEY**	90.33	89.98
**FLT3 (Y591-V592insVDFREYEYD)**	107.53	105.89
**FYN (Y531F)**	100.18	98.84
**JAK2 (V617F)**	107.01	106.82
**KSR1 (A635F)**	108.04	105.41
**KSR1 (L639F)**	97.15	94.41
**KSR2 (R676S)**	93.31	90.78
**LRRK2 (G2019S)**	94.26	92.91
**LRRK2 (I2020T)**	112.16	110.60
**LRRK2 (R1441C)**	93.71	91.90
**MEK1 (P124L)**	95.01	94.01
**MELK (T460M)**	94.54	94.33
**P38a (T106M)**	104.08	102.27
**PDGFRa (D842V)**	103.38	102.24
**PDGFRa (T674I)**	116.00	110.27
**PDGFRa (V561D)**	103.33	102.70
**PDGFRa-FIP1L1**	89.65	89.52
**PDGFRb-TPM3**	98.26	97.97
**PKD2 (G870E)**	106.30	105.12
**PKMzeta**	95.20	92.91
**PKN1-TECR (Tex1Pex10)**	105.39	105.39
**RET (A883F)**	105.17	104.71
**RET (E762Q)**	100.70	98.80
**RET (G691S)**	95.85	95.27
**RET (L790F)**	107.89	102.98
**RET (M918T)**	105.81	104.22
**RET (R749T)**	107.01	105.36
**RET (R813Q)**	101.46	101.22
**RET (R912P)**	99.06	98.29
**RET (S891A)**	107.95	103.94
**RET (S904A)**	106.75	105.15
**RET (S904F)**	99.76	98.74
**RET (V778I)**	101.75	100.56
**RET (V804E)**	107.07	106.17
**RET (V804L)**	104.76	102.96
**RET (V804L)-KIF5B**	106.39	104.83
**RET (V804M)**	107.57	106.78
**RET (V804M)-KIF5B**	99.83	97.12
**RET (Y791F)**	112.48	112.43
**RET (Y806H)**	120.25	118.47
**RET-BCR**	114.49	113.51
**RET-CCDC6 (PTC1)**	105.16	104.93
**RET-KIF5B (Kex15Rex14)**	97.65	95.25
**RET-NCOA4 (PTC3)**	102.16	102.09
**RET-PRKAR1A (PTC2)**	104.28	104.25
**ROS1 (G2032R)**	104.93	100.37
**ROS1-GOPC**	106.67	106.20

**ROS1-TPM3**	100.51	100.21
**RSK2 (I416V)**	100.70	99.50
**RSK2 (L608F)**	108.98	104.33
**TIE2 (A1124V)**	82.22	78.48
**TIE2 (P883A)**	83.15	82.89
**TIE2 (R849W)**	102.46	100.85
**TIE2 (Y1108F)**	96.83	94.28
**TIE2 (Y897C)**	87.71	87.05
**TIE2 (Y897S)**	95.86	86.79
**TRKA (A608D)**	67.50	67.43
**TRKA (F589L)**	89.98	86.75
**TRKA (G595R)**	96.28	95.50
**TRKA (G595R/A608D)**	55.83	55.67
**TRKA (G595R/G667A)**	99.02	93.75
**TRKA (G595R/G667C)**	94.64	89.52
**TRKA (G595R/G667S)**	106.34	99.84
**TRKA (G595R/L657M)**	86.43	82.13
**TRKA (G667C)**	64.97	64.31
**TRKA (G667S)**	92.60	90.59
**TRKA (L657M)**	90.35	88.09
**TRKA-TFG (TRK-T3)**	121.72	113.42
**TRKA-TPM3**	72.59	71.21
**TRKA-TPR**	91.4	90.43
**TRKC (G623E)**	92.13	91.63
**TRKC (G623R)**	71.93	70.77
**TRKC (G623R/L686M)**	97.08	96.74
**TRKC (G696A)**	75.32	72.70
**TRKC (L686M)**	69.31	68.86
**YES1 (T348I)**	117.43	108.02
**ZAP70 (Y319F)**	99.26	98.22

NCI charts for cellular assay

Developmental Therapeutics Program One Dose Mean Graph	NSC: D-795331 / 1	Conc: 1.00E-5 Molar	Test Date: Jan 17, 2017
	Experiment ID: 1701OS42		Report Date: Feb 01, 2017

Panel/Cell Line	Growth Percent
**Leukemia**	
CCRF-CEM	55.02
HL-60(TB)	49.87
K-562	20.77
MOLT-4	35.96
RPMI-8226	51.07
SR	57.82
**Non-Small Cell Lung Cancer**	
A549/ATCC	85.40
EKVX	101.00
HOP-62	92.44
HOP-92	73.27
NCI-H226	98.87
NCI-H23	97.83
NCI-H322M	81.34
NCI-H460	81.76
NCI-H522	81.38
**Colon Cancer**	
COLO 205	94.11
HCC-2998	98.66
HCT-116	77.44
HCT-15	113.34
HT29	80.83
KM12	91.43
SW-620	95.42
**CNS Cancer**	
SF-268	79.71
SF-295	97.53
SF-539	85.74
SNB-19	77.50
SNB-75	70.97
U251	68.10
**Melanoma**	
LOX IMVI	88.37
MALME-3M	96.70
M14	82.02
MDA-MB-435	85.36
SK-MEL-2	90.07
SK-MEL-28	95.80
SK-MEL-5	79.43
UACC-257	86.01
UACC-62	83.41
**Ovarian Cancer**	
IGROV1	77.80
OVCAR-3	78.76
OVCAR-4	87.80
OVCAR-5	107.67
OVCAR-8	77.46
NCI/ADR-RES	99.16
SK-OV-3	104.74
**Renal Cancer**	
786-0	92.50
A498	71.88
ACHN	93.77
RXF 393	70.26
SN12C	89.10
TK-10	89.66
UO-31	70.24
**Prostate Cancer**	
PC-3	51.31
DU-145	94.27
**Breast Cancer**	
MCF7	58.43
MDA-MB-231/ATCC	89.06
HS 578T	76.26
BT-549	90.28
T-47D	63.11
MDA-MB-468	78.10
Mean	81.24
Delta	60.47
Range	92.57

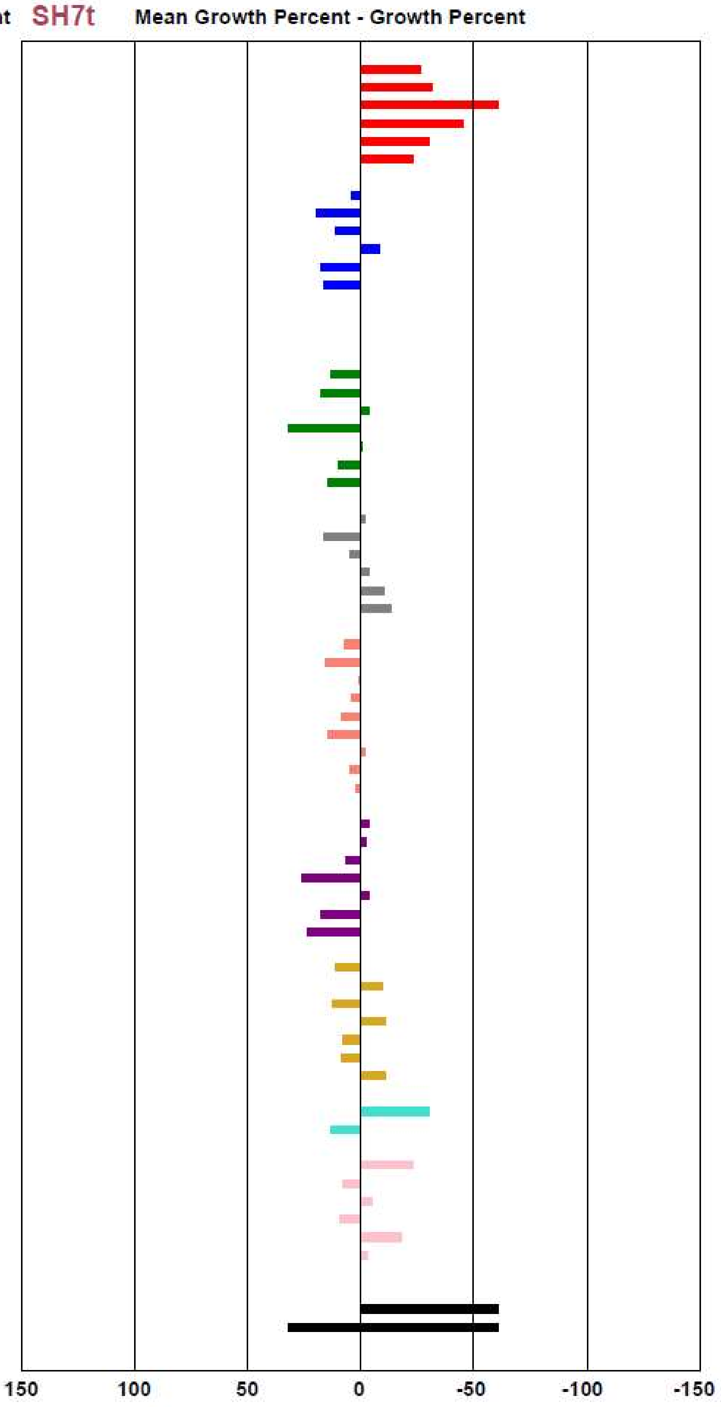

Figure S93. Single dose assay of compound **7t** a panel of 60 cancer cell lines.

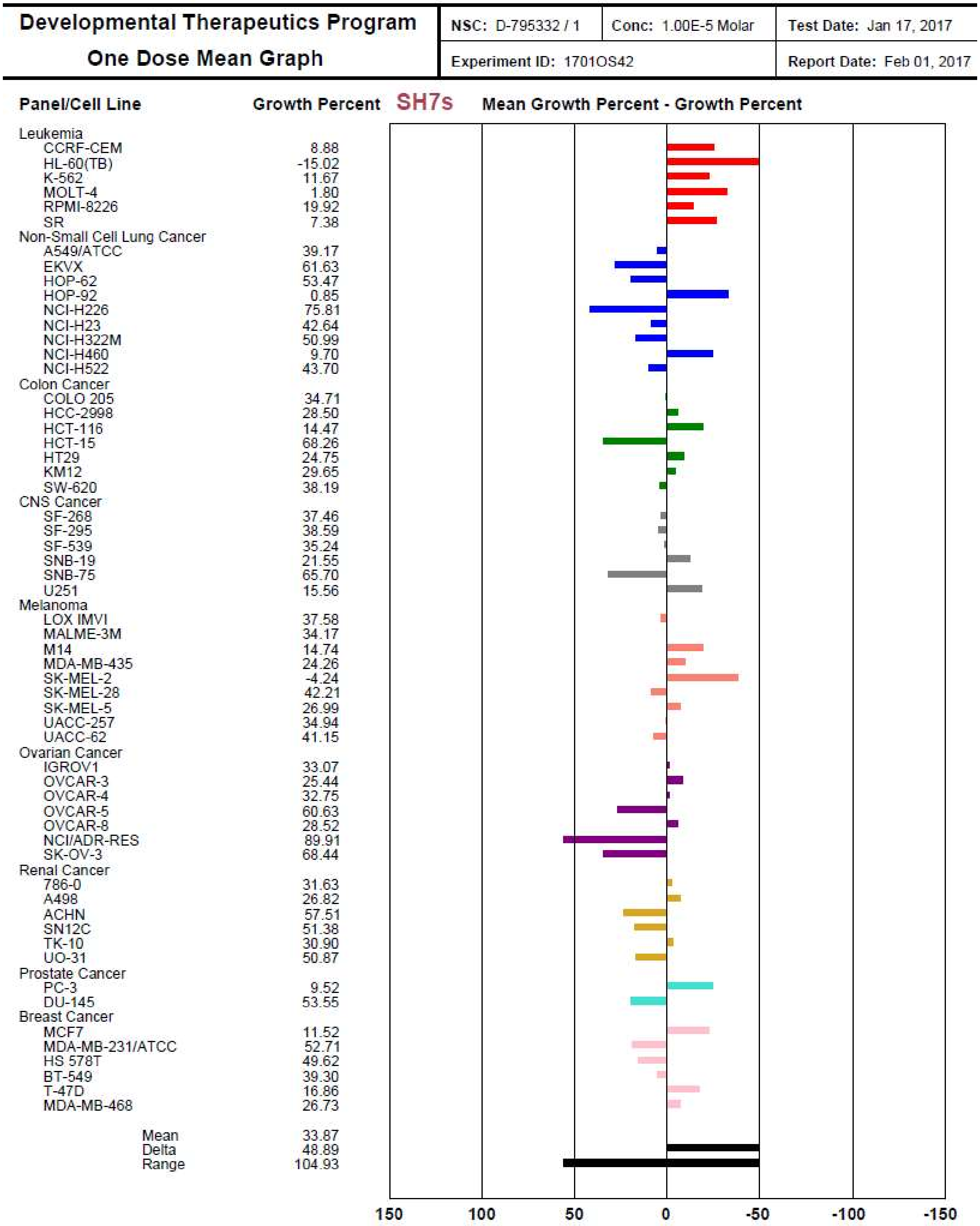

Figure S94. Single dose assay of compound **7s** a panel of 60 cancer cell lines**.**

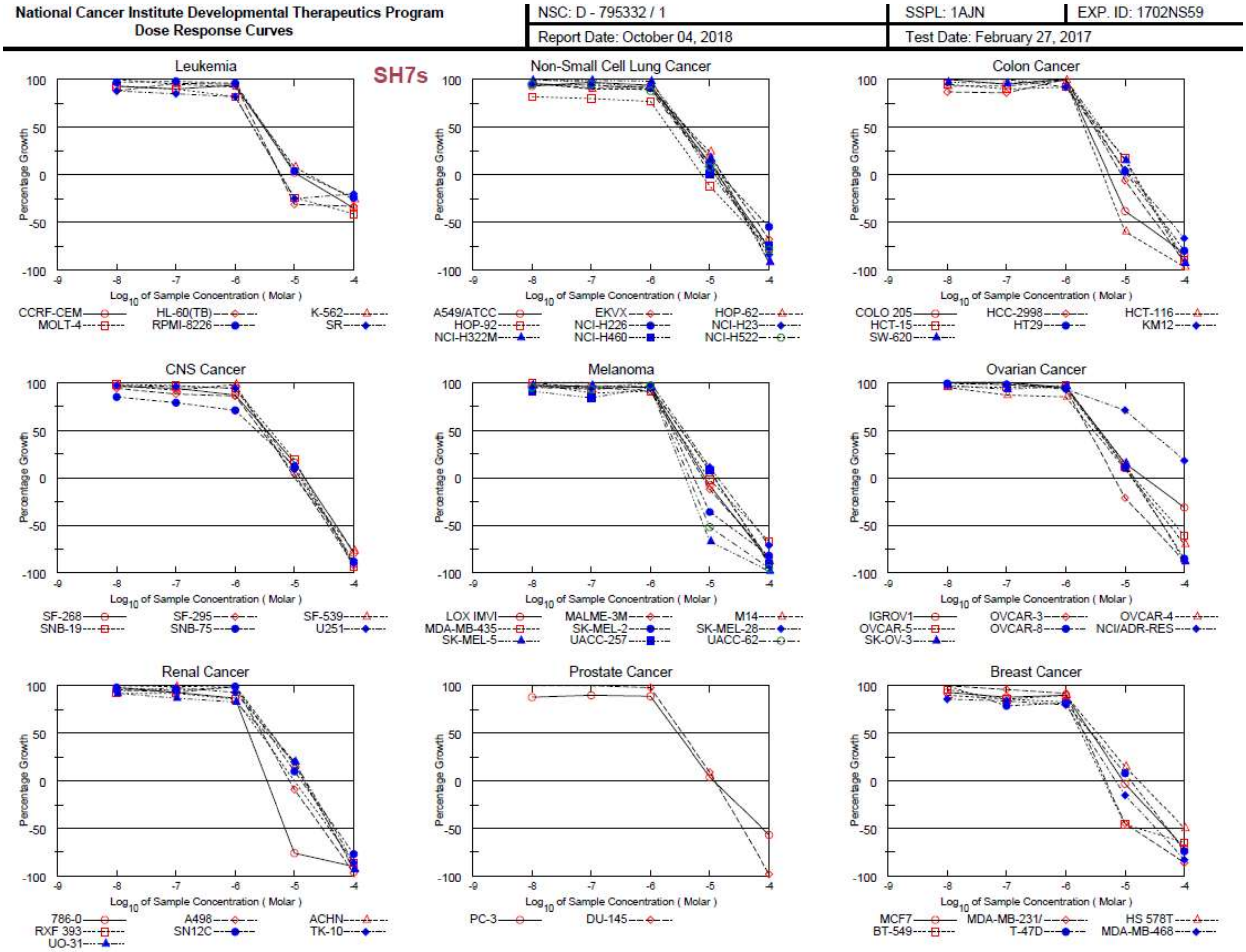

Figure S95. Five dose assay of compound **7s** a panel of 60 cancer cell lines**.**

National Cancer Institute Developmental Therapeutics Program
In-Vitro Testing Results

NSC : D - 795332 / 1	Experiment ID : 1702NS59	Test Type : 08	Units : Molar
Report Date : October 04, 2018	Test Date : February 27, 2017	QNS :	MC :
COMI : SH7s	Stain Reagent : SRB Dual-Pass Related	SSPL : 1AJN	

			Log10 Concentration												
	Time		Mean Optical Densities					Percent Growth							
Panel/Cell Line	Zero	Ctrl	-8.0	-7.0	-6.0	-5.0	-4.0	-8.0	-7.0	-6.0	-5.0	-4.0	GI50	TGI	LC50
Leukemia															
CCRF-CEM	0.560	3.030	2.857	2.780	2.881	0.607	0.366	93	90	94	2	-35	3.00E-6	1.13E-5	> 1.00E-4
HL-60(TB)	0.894	2.942	2.692	2.860	2.839	0.617	0.595	88	96	95	-31	-33	2.27E-6	5.67E-6	> 1.00E-4
K-562	0.149	1.482	1.482	1.412	1.370	0.251	0.111	100	95	92	8	-26	3.13E-6	1.69E-5	> 1.00E-4
MOLT-4	0.784	2.908	2.753	2.704	2.526	0.599	0.465	93	90	82	-24	-41	2.01E-6	5.97E-6	> 1.00E-4
RPMI-8226	0.926	3.055	2.996	3.007	2.965	1.003	0.703	97	98	96	4	-24	3.14E-6	1.35E-5	> 1.00E-4
SR	0.329	1.338	1.217	1.184	1.157	0.247	0.264	88	85	82	-25	-20	1.99E-6	5.83E-6	> 1.00E-4
Non-Small Cell Lung Cancer															
A549/ATCC	0.226	1.346	1.268	1.314	1.284	0.361	0.051	93	97	94	12	-77	3.46E-6	1.36E-5	4.94E-5
EKVX	0.632	2.022	1.961	1.890	1.922	0.890	0.201	96	91	93	19	-68	3.77E-6	1.64E-5	6.17E-5
HOP-62	0.593	1.598	1.562	1.500	1.500	0.836	0.046	96	90	90	24	-92	4.07E-6	1.61E-5	4.33E-5
HOP-92	1.333	1.992	1.871	1.860	1.842	1.172	0.293	82	80	77	-12	-78	2.02E-6	7.32E-6	3.75E-5
NCI-H226	0.900	2.325	2.254	2.234	2.191	1.056	0.405	95	94	91	11	-55	3.23E-6	1.46E-5	8.40E-5
NCI-H23	0.771	2.302	2.298	2.250	2.163	0.901	0.125	100	97	91	8	-84	3.14E-6	1.24E-5	4.30E-5
NCI-H322M	0.737	1.767	1.760	1.753	1.745	0.912	0.060	99	99	98	17	-92	3.91E-6	1.43E-5	4.12E-5
NCI-H460	0.245	2.405	2.486	2.411	2.583	0.270	0.062	104	100	108	1	-75	3.50E-6	1.04E-5	4.73E-5
NCI-H522	0.925	2.166	2.103	2.109	2.020	1.021	0.193	95	95	88	8	-79	2.98E-6	1.23E-5	4.61E-5
Colon Cancer															
COLO 205	0.417	1.604	1.669	1.541	1.608	0.259	0.069	105	95	100	-38	-83	2.31E-6	5.31E-6	1.84E-5
HCC-2998	0.779	2.377	2.162	2.160	2.456	0.733	0.061	87	86	105	-6	-92	3.13E-6	8.85E-6	3.24E-5
HCT-116	0.203	1.696	1.597	1.586	1.685	0.082	0.006	93	93	99	-60	-97	2.04E-6	4.21E-6	8.70E-6
HCT-15	0.357	2.360	2.260	2.160	2.198	0.698	0.036	95	90	92	17	-90	3.63E-6	1.44E-5	4.23E-5
HT29	0.269	1.773	1.802	1.767	1.902	0.315	0.055	102	100	109	3	-80	3.59E-6	1.09E-5	4.39E-5
KM12	0.400	2.436	2.438	2.466	2.282	0.493	0.132	100	101	92	5	-67	3.04E-6	1.16E-5	5.79E-5
SW-620	0.236	1.637	1.599	1.582	1.631	0.447	0.016	97	96	100	15	-93	3.86E-6	1.38E-5	3.98E-5
CNS Cancer															
SF-268	0.654	2.276	2.206	2.180	2.072	0.913	0.137	96	94	87	16	-79	3.34E-6	1.47E-5	4.94E-5
SF-295	0.647	2.094	2.011	1.922	1.886	0.700	0.052	94	88	86	4	-92	2.72E-6	1.09E-5	3.64E-5
SF-539	0.942	2.627	2.579	2.490	2.588	0.938	0.215	97	92	98	.	-77	3.06E-6	9.90E-6	4.42E-5
SNB-19	0.568	1.879	1.858	1.838	1.797	0.817	0.037	98	97	94	19	-93	3.85E-6	1.47E-5	4.10E-5
SNB-75	0.867	1.785	1.647	1.588	1.522	0.981	0.103	85	79	71	12	-88	2.30E-6	1.33E-5	4.18E-5
U251	0.451	2.116	2.072	2.048	2.022	0.599	0.045	97	96	94	9	-90	3.30E-6	1.23E-5	3.93E-5
Melanoma															
LOX IMVI	0.430	2.503	2.440	2.425	2.409	0.396	0.054	97	96	95	-8	-88	2.75E-6	8.36E-6	3.37E-5
MALME-3M	0.642	1.164	1.139	1.139	1.117	0.568	0.104	95	95	91	-12	-84	2.51E-6	7.72E-6	3.40E-5
M14	0.475	1.896	1.849	1.795	1.842	0.581	0.056	97	93	96	7	-88	3.32E-6	1.20E-5	3.99E-5
MDA-MB-435	0.393	2.320	2.299	2.102	2.147	0.387	0.131	99	89	91	-2	-67	2.77E-6	9.60E-6	5.54E-5
SK-MEL-2	1.160	2.267	2.377	2.329	2.307	0.742	0.204	110	106	104	-36	-82	2.42E-6	5.52E-6	2.00E-5
SK-MEL-28	0.680	1.947	1.952	1.866	1.971	0.822	0.195	100	94	102	11	-71	3.73E-6	1.37E-5	5.51E-5
SK-MEL-5	0.719	3.025	2.959	2.946	2.915	0.235	0.017	97	97	95	-67	-98	1.90E-6	3.85E-6	7.82E-6
UACC-257	0.881	1.983	1.886	1.809	1.932	0.966	0.076	91	84	95	8	-91	3.29E-6	1.20E-5	3.82E-5
UACC-62	1.005	2.939	2.838	2.810	2.889	0.478	0.048	95	93	97	-52	-95	2.07E-6	4.47E-6	9.63E-6
Ovarian Cancer															
IGROV1	0.470	1.711	1.721	1.725	1.646	0.660	0.323	101	101	95	15	-31	3.66E-6	2.13E-5	> 1.00E-4
OVCAR-3	0.440	1.643	1.683	1.643	1.568	0.347	0.055	103	100	94	-21	-88	2.40E-6	6.53E-6	2.72E-5
OVCAR-4	0.805	1.738	1.692	1.618	1.597	0.893	0.242	95	87	85	9	-70	2.90E-6	1.31E-5	5.60E-5
OVCAR-5	0.530	1.726	1.677	1.666	1.691	0.667	0.208	96	95	97	11	-61	3.54E-6	1.44E-5	7.08E-5
OVCAR-8	0.232	1.162	1.152	1.143	1.105	0.335	0.035	99	98	94	11	-85	3.39E-6	1.30E-5	4.31E-5
NCI/ADR-RES	0.539	1.898	1.904	1.871	1.799	1.503	0.779	100	98	93	71	18	2.47E-5	> 1.00E-4	> 1.00E-4
SK-OV-3	0.808	2.011	1.972	1.923	1.962	0.989	0.099	97	93	96	15	-88	3.69E-6	1.40E-5	4.29E-5
Renal Cancer															
786-0	0.790	2.612	2.568	2.482	2.371	0.191	0.081	98	93	87	-76	-90	1.68E-6	3.42E-6	6.94E-6
A498	1.338	2.046	2.015	1.994	2.037	1.221	0.038	96	93	99	-9	-97	2.84E-6	8.28E-6	2.93E-5
ACHN	0.359	1.617	1.679	1.600	1.617	0.567	0.029	105	99	100	17	-92	3.97E-6	1.42E-5	4.10E-5
RXF 393	0.794	1.439	1.387	1.390	1.349	0.789	0.111	92	92	86	.	-86	2.60E-6	9.83E-6	3.78E-5
SN12C	0.464	1.826	1.796	1.758	1.814	0.606	0.106	98	95	99	10	-77	3.58E-6	1.31E-5	4.89E-5
TK-10	0.839	1.965	1.902	1.947	1.884	1.061	0.114	94	98	93	20	-86	3.85E-6	1.53E-5	4.53E-5
UO-31	0.587	1.646	1.558	1.505	1.471	0.803	0.042	92	87	83	20	-93	3.39E-6	1.51E-5	4.18E-5
Prostate Cancer															
PC-3	0.615	1.675	1.545	1.569	1.561	0.653	0.266	88	90	89	4	-57	2.87E-6	1.14E-5	7.71E-5
DU-145	0.411	1.771	1.779	1.801	1.743	0.533	0.009	101	102	98	9	-98	3.46E-6	1.21E-5	3.56E-5
Breast Cancer															
MCF7	0.665	2.775	2.624	2.519	2.559	0.643	0.194	93	88	90	-3	-71	2.67E-6	9.21E-6	4.91E-5
MDA-MB-231/ATCC	0.443	1.164	1.169	1.135	1.109	0.245	0.061	101	96	92	-45	-86	2.04E-6	4.72E-6	1.34E-5
HS 578T	1.333	2.321	2.221	2.179	2.227	1.477	0.673	90	86	90	15	-50	3.41E-6	1.69E-5	> 1.00E-4
BT-549	0.747	1.486	1.456	1.386	1.363	0.403	0.263	96	86	83	-46	-65	1.81E-6	4.40E-6	1.61E-5
T-47D	0.754	1.677	1.682	1.484	1.509	0.825	0.199	100	79	82	8	-74	2.69E-6	1.24E-5	5.12E-5
MDA-MB-468	0.980	1.933	1.800	1.783	1.739	0.829	0.165	86	84	80	-15	-83	2.05E-6	6.88E-6	3.23E-5

Figure S96. Five dose assay of compound **7s** a panel of 60 cancer cell lines.
